# Translation, cross-cultural adaptation and content validation of the Global Trigger Tool surgical module

**DOI:** 10.1590/0034-7167-2021-0859

**Published:** 2022-07-18

**Authors:** Francine Taporosky Alpendre, Elaine Drehmer de Almeida Cruz, Josemar Batista, Eliane Cristina Sanches Maziero, Marilise Borges Brandão

**Affiliations:** I Universidade Federal do Paraná. Curitiba, Paraná, Brazil

**Keywords:** Validation Study, Quality Indicators, Health Care, Surgicenters, Medical Errors, Patient Safety., Estudio de Validación, Indicadores de Calidad de la Atención de Salud, Centros Quirúrgicos, Errores Médicos, Seguridad del Paciente., Estudo de Validação, Indicadores de Qualidade em Assistência à, Saúde, Centros Cirúrgicos, Erros Médicos, Segurança do Paciente.

## Abstract

**Objective:**

to translate, cross-culturally adapt and validate the Global Trigger Tool surgical module content for Brazil.

**Method:**

this is methodological research, carried out between March/2018 and February/2019, following the steps of translation, synthesis, back-translation, validation by the Delphi technique, pre-test and presentation to developers. Two translators, two back-translators, six professionals participated in the expert committee. A pre-test was carried out with a retrospective analysis of 244 medical records of adult patients. The content validity index and Cronbach’s alpha were determined for data analysis.

**Results:**

the translation and cross-cultural adaptation allowed adjustments of items for use in Brazil. The mean Content Validity Index was 1.38, and the degree of agreement among experts was 92.4%. Cronbach’s alpha was 0.83 for the 11 surgical triggers and their guidelines.

**Conclusion:**

the module was translated, cross-culturally adapted for Brazil, with high reliability to identify surgical adverse events.

## RESUMO

**Objetivo:**

traduzir, adaptar transculturalmente e validar o conteúdo do módulo cirúrgico do *Global Trigger Tool* para o Brasil.

**Método:**

pesquisa metodológica, realizada entre março/2018 e fevereiro/2019, seguindo os passos de tradução, síntese, retrotradução, validação pela técnica *Delphi*, pré-teste e apresentação para os desenvolvedores. Participaram dois tradutores, dois retrotradutores, seis profissionais para o comitê de especialistas. Realizou-se o pré-teste com análise retrospectiva de 244 prontuários de pacientes adultos. Determinou-se o índice de validade de conteúdo e alfa de Cronbach para análise dos dados.

**Resultados:**

a tradução e a adaptação transcultural permitiram ajustes dos itens para uso no Brasil. O Índice de Validade de Conteúdo médio foi 1,38, e grau de concordância entre os especialistas, 92,4 %. O alfa de Cronbach foi 0,83 para os 11 *triggers* cirúrgicos e respectivas orientações.

**Conclusão:**

o módulo foi traduzido e adaptado transculturalmente para o Brasil, com alta confiabilidade para identificar eventos adversos cirúrgicos.

## RESUMEN

**Objetivo:**

traducir, adaptar transculturalmente y validar el contenido del módulo quirúrgico Global Trigger Tool para Brasil.

**Método:**

investigación metodológica, realizada entre marzo/2018 y febrero/2019, siguiendo las etapas de traducción, síntesis, retrotraducción, validación por la técnica Delphi, pre-test y presentación a desarrolladores. En el comité de expertos participaron dos traductores, dos retrotraductores, seis profesionales. El pretest se realizó con un análisis retrospectivo de 244 historias clínicas de pacientes adultos. Para el análisis de los datos se determinó el índice de validez de contenido y el alfa de Cronbach.

**Resultados:**

la traducción y la adaptación transcultural permitieron ajustes de los ítems para uso en Brasil. El Índice de Validez de Contenido medio fue de 1,38 y el grado de acuerdo entre expertos fue del 92,4%. El alfa de Cronbach fue de 0,83 para los 11 disparadores quirúrgicos y sus orientaciones.

**Conclusión:**

el módulo fue traducido y adaptado transculturalmente para Brasil, con alta confiabilidad para identificar eventos adversos quirúrgicos.

## INTRODUCTION

In 1999, a publication by the Institute of Medicine, *To err is human: Building a Safer Health System*, revealed an alarming scenario of health errors and deaths resulting from inadequate care, becoming a historic milestone in patient safety^(1)^. Since then, there has been investment and concern by health organizations in issues related to safe practice promotion, such as adequacy of human resources, correct use of technological equipment and driving improvements in work processes that aim to advance the quality of care provided. However, there is a clear need for new approaches and strategies in order to significantly reduce the impacts arising from incidents^(2)^, especially those related to surgical care.

Adverse events (AEs) are conceptualized as incidents that result in harm to the patient. The World Health Organization (WHO) estimates that 234 million surgeries are performed worldwide, with the occurrence of seven million AEs, half of which are preventable. In developed countries, serious complications occur in about 3 to 16% and mortality rates are 0.4 to 0.8% among hospitalized patients^(3)^.

Systematic review analyzing events that should never happen, defined as never events, detected an error in the surgical site in 1/100,000 and retained surgical items in 1/10,000^(4)^. In Brazil, a pioneering study that aimed to retrospectively investigate 1,103 hospitalizations that occurred in three hospitals in the Southeast region, in 2003, identified an incidence of patients with AEs of 7.6%; 66.7% of cases were preventable and 34.7% occurred in the operating room^(5)^. Despite some advances in patient safety, more recent investigations conducted in Brazilian general hospitals revealed the magnitude and persistence of the problem at the national level, revealing a prevalence of AEs between 21.8%^(6)^ and an incidence of 33.7%^(7)^. It was estimated, respectively, that of the 60 and 266 cases identified, 90% and 58.3% of the events were preventable^(6-7)^.

The investigations^(6-7)^ applied the method proposed by the Canadian Adverse Event Study (CAES) protocol and used a potential AE tracking instrument translated and adapted for use in Brazil. However, it is observed that there is a scarcity of epidemiological data about these diseases in the country, possibly due to the constant use of traditional methods for measurement such as the use of voluntary notification, auditing and ombudsman systems. It is assumed the lack of precise methodologies to detect these events in the different sectors, specialties and health services^(8)^. When considering the growing need for surgical care^(9)^, proportional are the risks for the occurrence of AEs associated with interdisciplinarity, dependence on individual performance and complex care, demanding the development of reliable methods for assessing patient safety^(10)^.

In this context, it becomes relevant to develop and/or provide different validated tools to help health professionals, researchers and management teams in the identification and temporal monitoring of AEs. The Global Trigger Tool (GTT) methodology, developed in 2000 by the Institute of Healthcare Improvement (IHI) in the United States, proposes a retrospective review of a random sample of medical records, using a form that contains triggers, such as trackers of potential AEs^(11)^. This is widely used to investigate the occurrence of these conditions in hospital care, including the surgical context^(12-13)^.

The instrument consists of six modules (Care; Medication; Surgery; Intensive Care; Perinatal; and Emergency), each with their respective triggers, descriptions and guidelines. For instance, the surgical module trigger, named S3, refers to “Admission to Intensive Care Post-Operatively”. Guidelines on this trigger explains that ICU admission after major cardiac surgery is expected, but ICU admission after elective surgery, such as total knee replacement, would be unexpected. In other words, unexpected hospitalizations are often related to potential surgical AEs^(11)^.

Thus, considering the global problem regarding the individual and systemic factors that impact surgical patient safety promotion^(3)^, having a translated, adapted and validated instrument to track AEs in the Brazilian context means rich and productive learning, especially when recognizing that in the trajectory of professionals there is a difference between work reality and academic learning in surgical care. In the academic modality, an instrument that can be used as a tool to track possible AEs is learning that must be understood and developed for its efficient and effective use. However, in professional practice, the dynamic, complex and unique action will provide dexterity and objectivity to investigate the occurrence of these events, contributing to improving the quality of work processes in health and nursing, and in favor of positive construction of a safety culture so desired by high reliability organizations.

## OBJECTIVE

To translate, cross-culturally adapt and validate the Global Trigger Tool surgical module content for Brazil.

## METHODS

### Ethical aspects

This study followed the norms of Resolution 466/12 of the Brazilian National Health Council (*Conselho Nacional de Saúde*) and was approved by the Research Ethics Committee of the hospital where this stage of this project was developed. The IHI was previously consulted, obtaining authorization to use the instrument. All participants signed the Informed Consent Form.

### Study design, period, and place

This is methodological research, carried out from March 2018 to February 2019 for translation, cross-cultural adaptation and content validation of the IHI GTT surgical module into Brazilian Portuguese, after formal authorization by the authors of the material. The instrument consists of 11 triggers, identified by the letters S1 to S11, with their respective guidelines, which correspond to the triggers of potential surgical AEs to be used during a retrospective investigation of medical records.

The transcultural translation and adaptation process was based on international guidelines, with the following steps: 1 - Translation; 2 - Translation synthesis; 3- Back-translation; 4 - Content validation by an expert committee; 5 - Pre-test; 6 - Assessment by the authors of the instrument^(14)^. A pre-test of the validated version was carried out between January and February 2019, during a retrospective assessment of medical records to identify potential surgical AEs. Due to the data collection modality, patients were not approached directly.

### Population or sample; inclusion and exclusion criteria

The population and the criteria adopted were different according to each step. In Steps 1 and 2, two native Brazilian and English/Portuguese bilingual translators, aged over 18 years old, participated. One of them was a health professional, necessarily according to the method used. In Step 3, two independent bilingual translators, aged over 18 years, who were not previously informed about the concepts that would be assessed and did not even have access to the original version, were included.

In Step 4, instrument assessment and validation by an expert committee, a professional with knowledge in the health area, methodology, linguistics and English and Portuguese languages were included. The six experts were selected by consulting their resumes on the *Plataforma Lattes*, observing whether they had Brazilian nationality, knowledge of English language, Master’s or PhD in nursing or medicine, being an expert in at least one of the following areas: patient safety, scientific methodology, surgical care, translation and validation of research instruments. Invitation and instructions were sent electronically.

For Step 5 (pre-test), the target population was identified through the availability of a general list containing 11,021 records of hospitalizations of patients submitted, between June 2016 and May 2017, to surgery at a large teaching hospital in southern Brazil. First surgical procedure in the index hospitalization, performed by surgical specialty (general and digestive surgery, orthopedics and traumatology, neurosurgery, plastic surgery and liver transplantation), in patients aged ≥18 years and with a minimum hospital stay of 24 hours were considered inclusion criteria. The sample size calculation considered an incidence of surgical complications of 16%^(3)^, a maximum sampling error of 5% and a significance level of 5%, resulting in a random sample of 244 medical records.

The sample was excluded in the presence of any factor that limited the investigation of records, psychiatric patient records and when the information recorded electronically was unavailable, and such records were replaced according to the general list sequence.

### Study protocol

In Step 1, the original instrument was translated from English into Portuguese. Translation synthesis, by agreement among translators and researchers, was carried out in Step 2. Inconsistencies or doubts were clarified, resulting in the translation synthesis version. The synthesis version back-translation from Portuguese to English was performed in Step 3; then, the synthesis version was prepared by the researchers.

Step 4 was directed to content validation using the online Delphi technique, by six experts, to obtain consensus. The data collection instrument was named Expert Questionnaire, composed of 27 questions that were divided into two chunks with space for experts to record their observations and respective suggestions. The first chunk contained 22 questions corresponding to items that made up the synthesis version prepared in Step 3, for content assessment, fluidity, understanding of the wording of translated items and verification of the need for adaptation, inclusion and/or exclusion of items. The second chunk consisted of five questions to assess the semantic, idiomatic, conceptual, cultural and content equivalence^(14)^ of the translated instrument. Experts individually assigned a score for agreement to each question, which was later used to calculate the Content Validity Index (CVI).

Step 5 consisted of pre-test of the validated version, through a retrospective analysis of medical records. The IHI methodology stipulates a time limit of 20 minutes for the assessment of each selected medical record^(11)^. The medical records that met the tracking criteria for identifying potential AEs, i.e., that were positive for one or more triggers of the instrument, went on to the second investigation phase, which was carried out by two expert nurses in patient safety and a doctor specializing in the area of health risk assessment and quality management, who was responsible for confirming, or not, the cases of surgical AEs.

**Figure 1:**
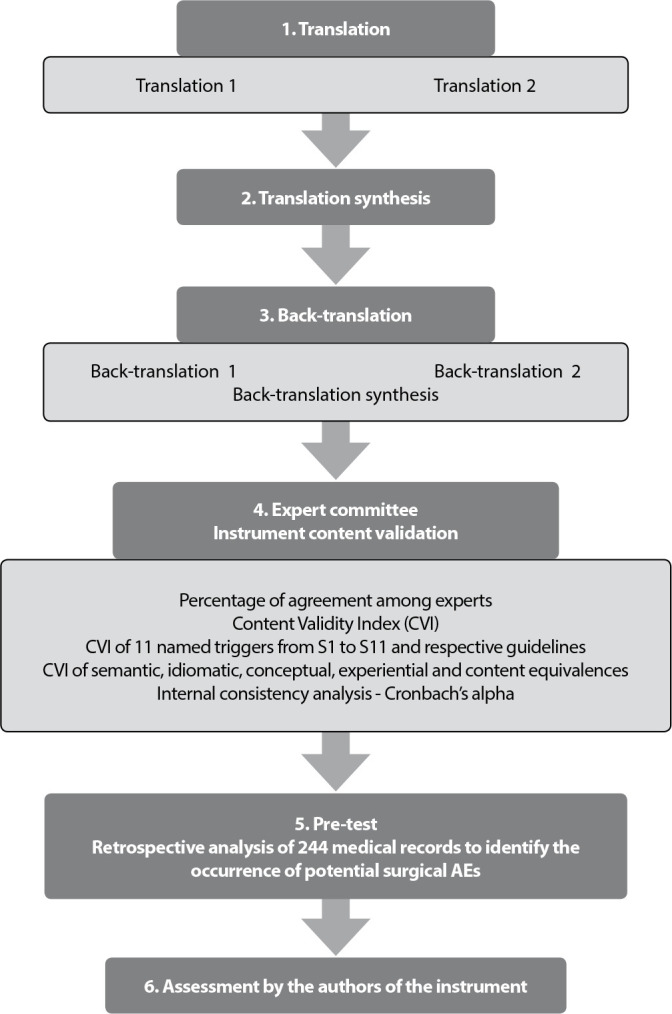
Flowchart of translation, cross-cultural adaptation and validation steps of the Global Trigger Tool surgical module for use in Brazil

The final version (Step 6) was sent to the authors of the original instrument for science and assessment, and was approved. Figure 1 summarizes the steps taken for instrument translation, cross-cultural adaptation and validation.

### Analysis of results, and statistics

The CVI calculation was used to assess consensus among experts and institute judgment rounds until a CVI ≥ 0.8 was obtained^(15)^. The percentage of agreement among experts in each domain was obtained by dividing the number of experts who attributed agreement to items and the total number of participants multiplied by 100. The CVI was calculated by the sum of the frequencies of the responses, multiplied by the score assigned to each Likert response and its weighting factor (-2) Strongly disagree, (-1) Disagree, (0) Indifferent, (+1) Agree and (+ 2) Totally agree, divided by the sum of the frequencies of each answer, using the weighted mean of the frequencies^(16)^. For this analysis, the items Totally disagree and Disagree (-1 and -2) and Agree and Totally agree (+1 and +2) were grouped. To assess reliability, Cronbach’s alpha was calculated. Values ≥ 0.7 were considered acceptable^(17)^.

## RESULTS

Translation and cross-cultural adaptation allowed adjustments so that the items were objective, understandable and suitable for use in Brazil. All discrepancies found were related to terms with similar meanings (e.g., *sala operatória* and *sala de cirurgia; morte* and *óbito; admissão* and *acesso; reparo and tratamento; operação* and *cirurgia*). Each translator produced an independent version named V1 and V2. It was decided on the terms considered more common in the Brazilian context, elaborating the translation synthesis version called V3. The back translations (V4 and V5) were equivalent. There are changes in tense in some descriptions of guidelines on triggers, however, differences in back translations were considered synonymous words. Back-translation synthesis by the researchers resulted in the V6 version.

Four nurses, a doctor and a bilingual professor majored in Literature are the expert committee members. This step was performed in a single assessment round and showed an agreement level of 92.4% and a mean CVI equal to 1.38. Table 1 presents agreement among experts and CVI of the 11 triggers and their respective guidelines.

**Table 1:** Agreement among experts and Content Validity Index for validating the Institute of Healthcare Improvement Global Trigger Tool surgical module (n=6), Curitiba, Paraná, Brazil, 2019

**Trigger*/*** **Guidelines** **S1 - S11**	**Agree** **%**	**Neither agree nor disagree** **%**	**Disagree** **%**	**Content Validity** **Index**
*Trigger* S1	83	0	17	1.17
Guidelines S1	83	0	17	1.17
*Trigger* S2	100	0	0	1.50
Guidelines S2	100	0	0	1.50
*Trigger* S3	83	17	0	1.33
Guidelines S3	83	17	0	1.33
*Trigger* S4	100	0	0	1.67
Guidelines S4	100	0	0	1.67
*Trigger* S5	100	0	0	1.67
Guidelines S5	100	0	0	1.67
*Trigger* S6	100	0	0	1.50
Guidelines S6	100	0	0	1.50
*Trigger* S7	83	0	17	1.17
Guidelines S7	83	0	17	1.17
*Trigger* S8	83	0	17	1.17
Guidelines S8	83	0	17	1.00
*Trigger* S9	100	0	0	1.50
Guidelines S9	100	0	0	1.50
*Trigge*r S10	100	0	0	1.50
Guidelines S10	100	0	0	1.67
*Trigge*r S11	83	0	17	1.00
Guidelines S11	83	0	17	1.00
Mean	92.4	-	-	1.38

Table 2 shows the mean of percentages of agreement and the CVI of assessments of semantic, idiomatic, conceptual, cultural and content equivalences. The mean degree of agreement among experts was 97.2%, and the mean CVI was 1.17.

**Table 2:** Agreement among experts and Content Validity Index for validating semantic, idiomatic, conceptual, cultural and content equivalences (n=6), Curitiba, Paraná, Brazil, 2019

**Equivalence**	**Agree** **%**	**Neither agree nor disagree** **%**	**Disagree** **%**	**Content Validity Index**
Semantic	83	0	17	1.00
Idiomatic	100	0	0	1.33
Conceptual	100	0	0	1.33
Cultural	100	0	0	1.33
Content	83	0	17	1.00
Mean	97.2	-	-	1.17

Cronbach’s alpha coefficient presented an overall value of 0.83 for the 11 surgical triggers and their respective orientations. For semantic, idiomatic, conceptual, cultural and content equivalences, Cronbach’s alpha was 0.77.

Experts’ suggestions and qualitative assessments were analyzed by the researchers, and the items considered relevant were accepted and modified in the instrument, as detailed below. In trigger S1, *retorno à cirurgia* was raplaced by *retorno à sala cirúrgica;* in trigger S3, *admissão ao cuidado intensivo no pós-operatório* was raplaced by *admissão na unidade de terapia intensiva no pós-operatório*; in trigger S3 guideline, *revisor precisa verificar o porquê a internação na unidade de cuidado intensivo ocorreu* was raplaced by *o revisor precisa verificar o motivo da internação na Unidade de Terapia Intensiva*; in trigger S6, *morte* was raplaced by *óbito*; in trigger S9, *aumento nos níveis de troponina superior a 1,5 ng/ml no pós operatório* was raplaced by *aumento nos níveis de troponina no pós-operatório pode indicar evento cardíaco;* in trigger S11, *decúbito was changed to lesão por pressão,* and *isso se refere a qualquer dentre inúmeras complicações* was replaced by *trata-se de qualquer uma entre várias complicações.* Overall, the following terms have been modified: *operatório* by *cirúrgico, operação* by *cirurgia,* and *unidade de cuidados pós anestesia* by *unidade de recuperação pós-anestésica.*

Based on the adjustments presented above, resulting in V7, in Step 5, a pre-test was carried out by the researchers through a retrospective analysis of 244 medical records. A positive tracking for the occurrence of potential AEs was found in 40 patients. In the second phase of investigation, in 31 cases, there was diagnostic confirmation of occurrence of surgical AEs, with a prevalence of 12.7%. Among the criteria proposed in the GTT surgical module, 90 positive trigger tools were identified as shown in Table 3.

**Table 3:** Positive criteria for tracking potential surgical adverse events according to the Institute of Healthcare Improvement Global Trigger Tool surgical module (n=90), Curitiba, Paraná, Brazil, 2019

**Global Trigger Tool - Institute of Healthcare Improvement**	n^**^	**%**
*S1- Retorno à sala cirúrgica*	20	22.22
*S2- Mudança no procedimento*	-	-
*S3- Admissão na Unidade de Terapia Intensiva no pós-operatório*	3	3.33
S4- Intubação ou reintubação ou uso de BiPap^*^ na Unidade de Recuperação Pós-Anestésica	1	1.11
*S5- Raio X Intraoperatório ou em Unidade de Recuperação Pós-Anestésica*	1	1.11
*S6- Óbito intra ou pós-operatório*	5	5.56
*S7- Ventilação mecânica superior a 24 horas no pós-operatório*	12	13.33
*S8- Administração intra-operatória de Epinefrina, Norepinefrina, Naloxona ou Romazicon*	2	2.22
*S9 - Aumento nos níveis de troponina superior a 1,5 ng/ml no pós-operatório*	-	-
*S10- Lesão, reparo ou remoção de órgão durante o procedimento cirúrgico*	16	17.79
*S11- Ocorrência de qualquer complicação cirúrgica*	30	33.33
Total	90	100

* BiPap = Bilevel Positive Pressure Airway;

** A medical record/patient could have more than one trigger

In step 6, the final version, called V8, was completed, which was forwarded to the authors of the original instrument. Chart 1 presents the final translated, cross-culturally adapted and validated version of the surgical module, and respective guidelines for use in Brazil.

**Chart 1:** Surgical triggers and their guidelines translated, adapted and validated for measuring adverse events in Brazil, Curitiba, Paraná, Brazil, 2019

**MÓDULO CIRÚRGICO E ORIENTAÇÕES DO *GLOBAL TRIGGER TOOL* DO *INSTITUTE FOR HEALTHCARE IMPROVEMENT* PARA MENSURAÇÃO DE EVENTOS ADVERSOS - VERSÃO BRASILEIRA**
***S1*** *- Retorno à sala cirúrgica.* ***Orientação*** *- O retorno à sala cirúrgica pode ser planejado ou não planejado, e ambos podem ser resultados de evento adverso. Exemplo de evento adverso seria paciente que teve hemorragia interna após a primeira cirurgia e necessitou de segunda cirurgia para explorar a causa e cessar o sangramento. Mesmo que a segunda cirurgia seja exploratória e não indique intercorrência, isto deveria ser considerado evento adverso.*
***S2-*** *Mudança no procedimento.* ***Orientação*** *- Quando o procedimento registrado nas anotações pós-operatórias for diferente em relação ao planejado nas anotações ou documentos pré-operatórios, constantes no consentimento cirúrgico, o revisor deve procurar por detalhes que justifiquem a alteração. Mudança inesperada no procedimento devido a complicações ou falha no dispositivo ou equipamento deve ser considerada evento adverso, particularmente se houver aumento no tempo de permanência ou ocorrência de lesão evidente.*
***S3-*** *Admissão na Unidade de Terapia Intensiva no pós-operatório.* ***Orientação*** *- A admissão em Unidade de Terapia Intensiva pode ser indicação pós-operatória normal ou pode ser inesperada. As internações inesperadas frequentemente estão relacionadas às ocorrências de eventos adversos cirúrgicos. Por exemplo, a admissão na Unidade de Terapia Intensiva após o reparo de aneurisma aórtico pode ser esperada, mas a admissão após artroplastia de joelho seria incomum. O revisor precisa verificar o motivo da internação na Unidade de Terapia Intensiva.*
***S4-*** Intubação ou reintubação ou uso de BiPap* na Unidade de Recuperação Pós-Anestésica. ***Orientação*** - Anestésicos, sedativos ou medicamentos para dor podem resultar em depressão respiratória, exigindo o uso de BiPap* ou reintubação pós-operatória, o que seria evento adverso. *O termo BiPap significa Bilevel Positive Pressure Airway (equipamento de ventilação não invasiva que oferece dois níveis de pressão inspiratória e expiratória, administrado por intermédio de máscara nasal ou facial).
***S5-*** *Raio X Intraoperatório ou em Unidade de Recuperação Pós-Anestésica.* ***Orientação*** *- Qualquer imagem que não seja rotineira ao procedimento requer investigação. Raio-x realizado devido à suspeita de itens retidos ou contagem incorreta de instrumento ou compressa é considerado gatilho positivo. A identificação de item retido que necessite novo procedimento é considerada evento adverso. Se o item retido for identificado e removido, sem qualquer evidência adicional de dano ou reoperação do paciente, não se considera evento adverso.*
***S6-*** *Óbito intra ou pós-operatório.* ***Orientação*** *- Todas os óbitos que ocorrem no intra-operatório são considerados eventos adversos, a menos que o óbito seja claramente esperado e a cirurgia tenha sido de indicação extrema. Óbitos pós-operatórios exigem revisão nos registros em busca de especificidades, mas em geral todos serão considerados eventos adversos.*
***S7-*** *Ventilação mecânica superior a 24 Horas no pós-operatório.* ***Orientação*** *- A ventilação mecânica de curto prazo está planejada no pós-operatório de procedimentos cardíacos, torácicos grandes e certos procedimentos abdominais. Se o paciente necessitar de ventilação mecânica superior a 24 horas, um evento adverso intra-operatório ou pós-operatório deve ser considerado. Pacientes com doença pulmonar ou muscular preexistente podem ter mais dificuldade para sair rapidamente da ventilação mecânica no pós-operatório, mas isso não deve excluir automaticamente a possibilidade de ser evento adverso. Os revisores devem usar o julgamento clínico para determinar se os cuidados intra-operatórios e pós-operatórios eram necessários ou parte do processo da doença.*
***S8-*** *Administração intra-operatória de Epinefrina, Norepinefrina, Naloxona ou Romazicon. Esses medicamentos não são administrados rotineiramente no intra-operatório.* ***Orientação*** *- Revisar anotações da anestesia e da cirurgia para determinar o motivo da administração. Hipotensão causada por sangramento ou sedação excessiva são exemplos de eventos adversos que podem ser tratados com estes medicamentos.*
***S9-*** *Aumento nos níveis de troponina superior a 1,5 ng/ml no pós-operatório.* ***Orientação*** *- Aumento nos níveis de troponina no pós-operatório pode indicar evento cardíaco. Revisores precisarão utilizar julgamento clínico para determinar se um evento cardíaco ocorreu.*
***S10-*** *Lesão, reparo ou remoção de órgão durante o procedimento cirúrgico.* ***Orientação*** *- Revisar as anotações cirúrgicas e pós-operatórias em busca de evidências de que o procedimento incluía reparo ou remoção de algum órgão. A remoção ou reparo deve fazer parte de procedimento planejado ou este é evento adverso e, provavelmente, resultado de intercorrência cirúrgica como lesão acidental.*
***S11-*** *Ocorrência de qualquer complicação cirúrgica.* ***Orientação*** *- Trata-se de qualquer uma entre várias complicações, incluindo, mas não se limitando, a Embolia Pulmonar, Trombose Venosa Profunda, Lesão por Pressão, Isquemia do Miocárdio, Falência Renal, etc.*

## DISCUSSION

To improve the selection of measurement instruments used in research and clinical practice, the Consensus-based Standards Group for the Selection of Health Measurement Instruments, composed of an international multidisciplinary team, proposes that the translated version undergo a review by an expert committee and pre-test^(18)^. It is noteworthy that all the steps proposed in the methodology of this study were followed, analyzed and documented to achieve the objective proposed in this research.

During translation, synthesis and back-translation of the GTT surgical module, grammatical changes were made, adaptations of terms more suitable for use in Brazil and correct choice of words were carried out to ensure better understanding and clarity in the translated version, supporting another methodological study whose objective was to translate, adapt and validate GTT for use in medical-surgical departments in Portugal. This study considered the international recommendations of Intercultural Adaptation Protocol (CCAP), revealing that translation and back-translation presented insignificant differences, requiring minor modifications^(8)^.

Translated versions of the IHI GTT are available for use in Danish, German and Swedish. The British version has been adapted to reflect the local UK context, which was last revised in September 2008, with no changes made to the surgical module^(11)^. In this research, no item from the surgical module was excluded, similar to what was found in the translated and cross-culturally adapted version for use in Italian hospitals, which kept all items from the original instrument^(19)^.

In Step 4, for validating the instrument content, version V6 was assessed, which corresponds to the fusion of translations and back-translations made in the previous steps, in addition to assessment of semantic, idiomatic, conceptual, cultural and content equivalences. According to the online Delphi technique, consensus was determined among experts. The property of an instrument measuring exactly what it proposes corresponds to content validity, and cross-cultural validity concerns the extent to which evidence supports the inference that the original instrument and a culturally adapted one are equivalent^(20)^.

To ensure assessment quality, a careful selection of professionals was carried out and it was evidenced that consensus was reached in the first assessment round. There was an mean degree of agreement among experts of 92.4% and an mean CVI of 1.38 for the surgical trigger tools and respective guidelines (Expert Questionnaire first chunk), and mean degree of agreement of 97.2% and mean CVI of 1.17 for semantic, idiomatic, conceptual, cultural and content equivalences (Expert Questionnaire second chunk). Such results demonstrate that the instrument translated into Brazil was validated in its content, as studies describe that, in order to verify instrument validity, a minimum agreement percentage of 80% among experts and a CVI between 0.5 and 0.8 are necessary^(21-22)^.

These indexes measure the proportion of experts who agree on the items that make up the instrument, and allow each item to be analyzed individually and in full^(21-22)^. An expert committee must be formed between five and 10 expert judges with proven knowledge in the area of the instrument to carry out content assessment. Agreement ≥90%, as verified in this research, means that the domains are adequate and are corroborated with the CVI results found in the instrument validation for the Portuguese version, which were considered excellent^(8)^.

To obtain item semantic, conceptual and functional equivalence, the Portuguese version of the IHI GTT was improved through a focus group consisting of doctors, nurses and pharmacists, totaling 15 judges/experts. The changes implemented increased the predictive value of the instrument, which showed high internal consistency, with a Cronbach’s alpha of 0.83^(8)^. This is identical to that found in the present research, which identified global internal consistency of 0.83 for the 11 triggers and their respective orientations, as well as an overall value of 0.77 for semantic, idiomatic, conceptual, cultural and content equivalences. Cronbach’s alpha coefficient was considered satisfactory, evidencing that the instrument has high reliability. To ensure quality of results of scientific studies, validity and reliability verification is necessary, as they are measurement properties necessary to determine that the instruments are reliable and valid^(23)^. In this research, both the content validity and reliability of the instrument translated and adapted for Brazil were performed and achieved satisfactory results.

In Step 5, a pre-test was carried out, which, according to the methodology used, must be carried out in a sample of 30-40 individuals in order to verify if the items that make up the instrument are understandable for the purpose for which it is intended^(14,21)^. A pre-test was performed using the V7 version by the researchers during the analysis of a retrospective sample of 244 medical records of surgical patients, with signaling of 90 trigger tools in 40 patients in the first phase of investigation. These findings showed that the instrument was understandable, easy to apply in investigative practice and that it enabled the identification and measurement of potential surgical AEs. In the area of patient safety, triggers tools are used to guide the identification of events in medical records and other records used in the health area. It is recommended that, when there is such signaling, the case is analyzed to confirm, or not, the occurrence and severity of the damage caused to a patient and what were the factors that contributed to this aggravation^(24)^, as done in the present study, with assessment of potential events by three experts on the subject, for diagnostic confirmation, obtaining a prevalence of AEs of 12.7%.

Thus, it was found that the instrument used in the pre-test is understandable for researchers to use in the Brazilian context, meets the attributes of simplicity, applicability and possibility of measurement and identification^(3)^ of potential surgical AEs. However, the prevalence found was lower than that shown in a cross-sectional and retrospective study according to a random sample of 90 medical records to validate the general GTT, which showed a prevalence of 36%. Of the 142 AEs identified, 98% were found due to the presence of triggers, which shows that it is advantageous to use the GTT methodology regularly to identify and characterize the most frequent types of AEs, especially as it is a valid, sensitive and reproducible tool for surgical services^(8)^.

In Step 6, the final version (V8) was prepared, which was translated and cross-culturally adapted into Brazilian Portuguese. The use of a consistent method for instrument translation, cross-cultural adaptation and validation, in addition to the description of the process steps, show the readers that the research was carried out seriously. The reliability verified in instruments that underwent cross-cultural adaptation allows their use in professional practice^(21)^. In this way, the GTT methodology is considered a tool that has feasibility and utility to detect AEs, providing essential information to quality management professionals, in order to carry out prevention strategies in favor of continuous improvement and promotion of patient safety^(25-26)^.

### Study limitations

As limitations of this research, it appears that the IHI GTT surgical module was translated and adapted cross-culturally in few countries, which minimizes the processes for comparison and discussion of results. Not having performed the overall validity of psychometric properties (sensitivity, specificity, positive and negative predictive value) and by triggers to detect AEs is also a limitation.

### Contributions to nursing, health, and public policies

The use of an instrument validated in different contexts in Brazil will make it possible to generalize the use of this methodology to identify the occurrence of surgical AEs. The GTT methodology can be used by students, researchers and assistant nurses, area supervisors and managers, as well as other health professionals interested in the assessment and measurement of surgical AEs, through the use of a valid and reliable instrument for teaching, risk management, continuous improvement of the quality of processes and care provided in favor of patient safety.

## CONCLUSIONS

The instrument was translated, cross-culturally adapted and validated for Brazilian Portuguese, with assessment of semantic, idiomatic, conceptual, experimental and content equivalences. According to experts’ analysis, the results proved sufficient content and cross-cultural validity, as well as high instrument reliability. Thus, this can be considered a reliable, valid instrument with potential application in professional practice for risk management, in studies aimed at improving patient safety and the quality of services, applicable in investigations to identify, monitor, measure and assess the occurrence of surgical AEs as well as in academic activities.

## SUPPLEMENTARY MATERIAL

https://acervodigital.ufpr.br/handle/1884/66356.

0034-7167-reben-75-06-e20210859-sup01

## References

[1] Pierre MS, Grawe P, Bergstrom J, Neuhaus C (2022). 20 years after To Err Is Human: a bibliometric analysis of ‘the
IOM report’s’ impact on research on patient safety. Safe Sci.

[2] Godambe AS, Shah RK, Shah RK, Godambe SA (2021). Introduction: a case-based approach to quality
improvement. Patient Safety and Quality Improvement in Healthcare.

[3] World Health Organization (WHO) (2008). World alliance for patient safety. The second Global Patient safety
challenge: safe surgery saves lives[Internet].

[4] Hempel S, Maggard-Gibbons M, Nguyen DK, Dawes AJ, Miake-Lye I, Beroes JM (2015). Wrong-Site surgery, retained surgical items, and surgical fires:
a systematic review of surgical never events. JAMA Surg.

[5] Mendes W, Martins M, Rozenfeld S, Travassos C (2009). The assessment of adverse events in hospitals in
Brazil. Int J Qual Health Care.

[6] Batista J, Cruz EDA, Alpendre FT, Rocha DJM, Brandão MB, Maziero ECS (2019). Prevalence and avoidability of surgical adverse events in a
teaching hospital in Brazil. Rev Latino-Am Enfermagem.

[7] Zanetti ACB, Dias BM, Bernardes A, Capucho HC, Balsanelli AP, Moura AA (2021). Incidence and preventability of adverse events in adult patients
admitted to a Brazilian teaching hospital. Plos One.

[8] Pierdevara L, Porcel-Gálvez AM, Ferreira da Silva AM, Barrientos Trigo S, Eiras M (2020). Translation, Cross-Cultural Adaptation, and Measurement
Properties of the Portuguese Version of the Global Trigger Tool for Adverse
Events. Ther Clin Risk Manag.

[9] Shrime MG, Bickler SW, Alkire BC, Mock C (2015). Global burden of surgical disease: an estimation from the
provider perspective. Lancet Glob Health.

[10] Venneri F, Brown LB, Cammelli F, Haut ER, Donaldson L, Ricciardi W, Sheridan S, Tartaglia R (2021). Safe Surgery Saves Lives. Textbook of Patient Safety and Clinical Risk Management.

[11] Griffin FA, Resar RK (2009). IHI Innovation Series white paper[Internet].

[12] Parrinello V, Grasso E, Saglimbeni G, Patanè G, Scalia A, Murolo G (2019). Assessing the development and implementation of the Global
Trigger Tool method across a large health system in Sicily. F1000Res.

[13] Brösterhaus M, Hammer A, Kalina S, Grau S, Roeth AA, Ashmawy H (2020). Applying the Global Trigger Tool in German Hospitals: a pilot in
surgery and neurosurgery. J Patient Saf.

[14] Beaton DE, Bombadier C, Guilemin F, Ferraz MB (2000). Guidelines for the Process of Cross-Cultural Adaptation of
Self-Report Measures. Spine.

[15] Yusoff MSB (2019). ABC of content validation and content validity index
calculation. Educ Med J.

[16] Alexandre NMC, Coluci MZO (2011). Validade de conteúdo nos processos de construção e adaptação de
instrumentos de medida. Ciên Saúde Coletiva.

[17] Eisinga R, Grotenhuis M, Pelzer B (2013). The reliability of a two-item scale: Pearson, Cronbach, or
Spearman-Brown?. Int J Public Health.

[18] Mokkink LB, Prinsen CAC, Bouter LM, Vet HCW, Terwee CB (2016). The Consensus-based Standards for the selection of health
Measurement Instruments (COSMIN) and how to select an outcome measurement
instrument. Braz J Phys Ther.

[19] Mortaro A, Moretti F, Pascu D, Tessari L, Tardivo S, Pancheri S (2017). Adverse Events Detection Through Global Trigger Tool Methodology:
results From a 5-Year Study in an Italian Hospital and Opportunities to
Improve Interrater Reliability. J Patient Saf.

[20] Polit DF (2015). Assessing measurement in health: beyond reliability and
validity. Int J Nurs Stud.

[21] Lino CRM, Brüggemann OM, Souza ML, Barbosa SFF, Santos EKA (2017). The cross-cultural adaptation of research instruments conducted
by nurses in Brazil: an integrative review. Texto contexto-enferm.

[22] Revorêdo LS, Dantas MMC, Maia RS, Torres GV, Maia EMC (2016). Content validation of an instrument for identifying violence
against children. Acta Paul Enferm.

[23] Souza ACD, Costa Alexandre NM, Guirardello EB (2017). Psychometric properties in instruments evaluation of reliability
and validity. Epidemiol Serv Saude.

[24] Corrêa CDTSO, Mendes W (2017). Proposal of a trigger tool to assess adverse events in dental
care. Cad Saúde Pública.

[25] Pierdevara L, Ventura IM, Eiras M, Gracias AMB (2017). Trigger Tool na Segurança do Doente: uma revisão sistemática de
literatura. Port J Public Health.

[26] Hu Q, Wu B, Zhan M, Jia W, Huang Y, Xu T (2019). Adverse events identified by the global trigger tool at a
university hospital: a retrospective medical record review. J Evid Based.

