# Supplementary material for: Translation, cross-cultural adaptation and content validation of the Global Trigger Tool surgical module
Source: Rev Bras Enferm. 2022 Jul 18;75(6):e20210859. doi: 10.1590/0034-7167-2021-0859 (PMC9728827; doi:10.1590/0034-7167-2021-0859)
Supplement: 0034-7167-reben-75-06-e20210859-sup01 [file 0034-7167-reben-75-06-e20210859-sup01.pdf]

UNIVERSIDADE FEDERAL DO PARANÁ

FRANCINE TAPOROSKY ALPENDRE

AVALIAÇÃO DA CULTURA DE SEGURANÇA E EVENTOS ADVERSOS CIRÚRGICOS EM HOSPITAIS BRASILEIROS

CURITIBA

2019

FRANCINE TAPOROSKY ALPENDRE

AVALIAÇÃO DA CULTURA DE SEGURANÇA E EVENTOS ADVERSOS CIRÚRGICOS EM HOSPITAIS BRASILEIROS

Tese apresentada ao Curso de Doutorado do Programa de Pós-Graduação em Enfermagem, Departamento de Enfermagem, Setor de Ciências da Saúde, Universidade Federal do Paraná, para obtenção do título de Doutora em Enfermagem.Linha de Pesquisa: Processo de Cuidar em Saúde e Enfermagem, Políticas e Práticas de Educação, Saúde e Enfermagem

Grupo de Pesquisa GEMSA/UFPR

Orientadora: Profª Drª Elaine Drehmer de Almeida Cruz

CURITIBA

2019

Ficha catalográfica

Alpendre, Francine Taporosky
Avaliação da cultura de segurança e eventos adversos cirúrgicos em hospitais brasileiros [recurso eletrônico] / Francine Taporosky Alpendre – Curitiba, 2019.

Tese (doutorado) – Programa de Pós-Graduação em Enfermagem. Setor de Ciências da Saúde, Universidade Federal do Paraná, 2019.

Orientadora: Professora Dra. Elaine Drehmer de Almeida Cruz

1. Segurança do paciente. 2. Cultura organizacional. 3. Complicações pós-operatórias. 4. Estudos de validação. 5. Qualidade da assistência à saúde.
I. Cruz, Elaine Drehmer de Almeida. II. Universidade Federal do Paraná. III. Título.

CDD 362.10425

Maria da Conceição Kury da Silva CRB 9/1275

TERMO DE APROVAÇÃO

Os membros da Banca Examinadora designada pelo Colegiado do Programa de Pós-Graduação em ENFERMAGEM da Universidade Federal do Paraná foram convocados para realizar a arguição da Tese de Doutorado de **FRANCINE TAPOROSKY ALPENDRE**, intitulada: **AVALIAÇÃO DA CULTURA DE SEGURANÇA E EVENTOS ADVERSOS CIRÚRGICOS EM HOSPITAIS BRASILEIROS**, sob orientação da Profa. Dra. ELAINE DREHMER DE ALMEIDA CRUZ, após terem inquirido a aluna e realizado a avaliação do trabalho, são de parecer pela sua APROVAÇÃO no rito de defesa.

A outorga do título de Doutor está sujeita à homologação pelo colegiado, ao atendimento de todas as indicações e correções solicitadas pela banca e ao pleno atendimento das demandas regimentais do Programa de Pós-Graduação.

Curitiba, 28 de Novembro de 2019.

ELAINE DREHMER DE ALMEIDA CRUZ
Presidente da Banca Examinadora

MARIA DE FATIMA MANTOVANI
Avaliador Interno (UNIVERSIDADE FEDERAL DO PARANÁ)

JOÃO LUIZ VIEIRA DA SILVA
Avaliador Externo (UNIVERSIDADE FEDERAL DO PARANÁ)

ANA ELISA BAUER DE CAMARGO SILVA
Avaliador Externo (UNIVERSIDADE FEDERAL DE GOIÁS)

DEDICATÓRIA

Dedico esse trabalho para minha família. Ao meu esposo Ricardo, amor da minha vida, pela paciência, compreensão e por me apoiar em todos os momentos dessa caminhada. Aos meus filhos Ricardo Filho e Rafael, que tornam os meus dias mais felizes e representam o motivo da minha vontade de aprender a cada dia e ser melhor.

Ao meu irmão Eliézer, minha cunhada Marcella e meu sobrinho Leonardo, que me encorajam a superar todos os obstáculos que a vida apresenta. Aos meus pais Josué e Maria dos Anjos por todo amor, apoio incondicional, e ensinamentos sobre retidão, caráter, justiça, respeito as pessoas e amor a Deus.

AGRADECIMENTOS

Aos meus pais **Josué** e **Maria dos Anjos**; meu esposo **Ricardo** e meus filhos **Ricardo Filho** e **Rafael;** meu irmão **Eliézer**, cunhada **Marcella** e meu sobrinho **Leonardo**, que são meu porto seguro. Amo vocês!

À minha orientadora, **Profª. Drª. Elaine Drehmer de Almeida Cruz**, pelos ensinamentos, ideias, correções, competência, profissionalismo e amizade. Devido ao seu exemplo, a temática Segurança do Paciente faz parte da minha luta. Suas orientações e palavras de incentivo me conduziram a concretizar mais este projeto de vida. Você me fez ir além do que eu achava possível. Obrigada por acreditar em mim!

Aos membros da banca, Professoras Doutoras **Maria de Fátima Mantovani**, **Ana Elisa Bauer de Camargo e Silva**, **Professor Doutor João Luiz Vieira da Silva** e doutorando **Josemar Batista** pela disponibilidade e valiosas contribuições. Profª Fátima faz parte da minha caminhada desde o início da vida acadêmica, saiba que aprendo com você até hoje. Profª Ana Elisa só tenho a agradecer por estar presente mais uma vez neste momento tão especial, por ser exemplo de profissional atuante na Segurança do Paciente. Prof. Dr. João Luiz, que conheço desde a época de doutorando, tornou-se professor e médico cirurgião ortopedista, além de priorizar à excelência da técnica cirúrgica e atendimento humanizado aos pacientes. Josemar, pela amizade, troca de experiências, conhecimento e competência.

À **Dra. Marilise Borges Brandão** que voluntariamente aceitou participar e colaborar com a realização desta pesquisa. Seus conhecimentos na gestão de risco e segurança do paciente foram fundamentais neste processo.

À **Enfermeira Thaize** pela receptividade e profissionalismo durante a coleta de dados em sua instituição. Profissionais como você fazem a diferença!

Ao **Programa de Pós-Graduação em Enfermagem da Universidade Federal do Paraná** pela oportunidade de desenvolver esta pesquisa e a todos os professores pelos ensinamentos. Sinto-me honrada de fazer parte desta universidade deste o período da graduação.

Ao **Grupo de Estudos Multiprofissional em Saúde do Adulto – GEMSA**, pelo qual tenho carinho mais que especial, por ter participado desde o início de sua

formação. Agradeço aos colegas e professores pela parceria e ensinamentos. Este grupo de pesquisa representa o desenvolvimento do conhecimento científico na área da pesquisa em saúde e da enfermagem em prol do cuidado qualificado e seguro ao paciente.

Às colegas da turma do Doutorado pela convivência e aprendizado. Desejo sucesso a todas!

À amiga **Eliane Cristina Sanches Maziero**, pelo apoio, competência profissional, por me ouvir e me ajudar até o final desta pesquisa. Como aprendemos nesta jornada! Desejo tudo de melhor para você!

Aos **profissionais tradutores, retrotradutores e especialistas** por aceitarem o convite em participar desta pesquisa e por tornarem possível a realização da tradução, adaptação e validação de instrumento para o contexto brasileiro em prol da identificação e mensuração de eventos adversos cirúrgicos.

A todos que de alguma forma fizeram parte dessa caminhada, pelas palavras de incentivo, amizade e orações.

Principalmente a **Deus**, razão da minha existência, por me ajudar deste o princípio desta caminhada, pela sabedoria e por renovar minhas forças em todos os momentos durante o desenvolvimento desta pesquisa. Afinal, Ele é o alfa e o ômega, e sua fidelidade dura para sempre.

EPÍGRAFE

"Pode parecer um princípio estranho anunciar como requisito básico, em um hospital, que não se deve causar dano ao paciente".
(NIGHTINGALE, 1863).

RESUMO

Introdução: a segurança do paciente cirúrgico é componente crítico na qualidade do cuidado em saúde e danos evitáveis persistem, inaceitavelmente, frequentes. Objetivo: avaliar a cultura de segurança do paciente e identificar eventos adversos cirúrgicos. Metodologia: estudo analítico, transversal, de abordagem quantitativa, desenvolvido nas unidades de centro cirúrgico e de internação cirúrgica de três hospitais de ensino da região sul do Brasil, nominados A – hospital federal; B- hospital privado e C – hospital estadual. Entre maio de 2017 e abril de 2019 foram coletados os dados do estudo, com simultânea pesquisa metodológica para tradução, adaptação e validação, para língua portuguesa do Brasil, do módulo cirúrgico *Global Trigger Tool do Institute of Healthcare Improvement*, empregado para rastrear os eventos adversos, juntamente com o instrumento do *Canadian Adverse Event Study*. Os potenciais eventos adversos foram identificados em amostra aleatória simples, com 678 prontuários analisados; os confirmados por especialistas foram classificados por tipo, grau de dano e potencial de evitabilidade. Para avaliar a cultura de segurança foi usado o formulário, autoaplicável, *Hospital Survey on Patient Safety Culture* com 381 profissionais das equipes de enfermagem e médica, por amostra não probabilística intencional; dimensões com índices ≥75% foram consideradas fortalecidas. Para a análise dos dados utilizou-se os seguintes testes: para a tradução o índice de validade de conteúdo para concordância entre especialistas e coeficiente Alfa de Cronbach; na associação entre os hospitais utilizou-se modelo de análise da variância ANOVA com um fator ou o teste não-paramétrico de Kruskal-Wallis e Fisher ou teste de Qui-quadrado; valores de $p<0,05$ indicaram significância estatística para eventos adversos. Na análise da cultura de segurança utilizou-se o teste Alfa de Cronbach para confiabilidade do instrumento, o modelo da variância com um fator ou o teste não-paramétrico de Kruskal-Wallis para variáveis quantitativas e teste de Qui-quadrado para variáveis categóricas; valores de $p<0,05$ indicaram significância estatística. Resultados: A cultura de segurança apresentou-se fragilizada (≤50%), nos três hospitais, nas dimensões Apoio da gestão para a segurança do paciente, Percepção geral da segurança do paciente, Retorno da informação e comunicação sobre o erro, Adequação de profissionais, Passagem de plantão/turnos e transferências e Respostas não punitivas ao erro. Trabalho em equipe dentro das unidades foi identificada como fortalecida no hospital privado; o Alfa de *Cronbach* geral mostrou confiabilidade do instrumento. O módulo cirúrgico traduzido foi considerado válido, confiável e com equivalências semântica, idiomática, conceitual, cultural e de conteúdo satisfatórias. A prevalência média dos eventos foi de 21%, 14,2% e 12,7% (p=0,044), e de readmissões de 9,8%; 29,4% e 45,2% (p=0,003), respectivamente, nos Hospitais A, B e C, evidenciando diferença estatística. A maioria dos eventos foi considerada evitável, resultando em danos leves e/ou moderados; os eventos prevalentes foram Infecção de Sítio Cirúrgico, Septicemia/Choque Séptico, Hematoma/Seroma, Deiscência de Sutura, Fístulas e Infecção do Trato Urinário. Conclusão: as dimensões frágeis da cultura de segurança são expressivas e mostram prioridades ao seu fortalecimento. A tradução e adaptação do módulo cirúrgico contribuiu para o rastreamento de eventos adversos e constitui instrumento aplicável no Brasil; o método de rastreamento evidenciou alta prevalência de eventos adversos cirúrgicos, apontando a importância do tema e a relevância de estratégias em prol da promoção da segurança cirúrgica e qualidade assistencial hospitalar.

Palavras-chaves: Segurança do Paciente; Cultura Organizacional; Complicações Pós-Operatórias; Estudos de Validação; Qualidade da Assistência à Saúde.

ABSTRACT

Introduction: Surgical patient safety is a critical component in the quality of health care where the preventable injuries persist, being unacceptably frequent. Objective: To evaluate patient safety culture and identify surgical adverse events. Methodology: An analytical, cross-sectional study with a quantitative approach, developed in the surgery room and inpatient units of three teaching hospitals in southern Brazil, referred to as A: federal hospital; B: private hospital and C: state hospital. The study's data were collected from May 2017 to April 2019, with simultaneous methodological research for translating, adapting and validating, for Brazilian Portuguese, the Global Trigger Tool surgical module of the Institute of Healthcare Improvement, employed to track adverse events, together with the Canadian Adverse Event Study instrument. Potential adverse events were identified in a simple random sample, with 678 analyzed medical records; those confirmed by experts were classified by type, degree of damage and avoidability potential. To assess the safety culture, the self-administered form, Hospital Survey on Patient Safety Culture with 381 professionals from the nursing and medical teams, by an intentional non-probabilistic sample; dimensions with indexes ≥75% were considered strengthened. For data analysis the following tests were used: for the translation, the content validity index for agreement among experts and Cronbach's alpha coefficient; in the association between the hospitals, the one-way ANOVA variance analysis model or the Kruskal-Wallis and Fisher non-parametric test or the Chi-square test were used; p values <0.05 indicated statistical significance for adverse events. Cronbach's alpha test for reliability of the safety culture, the one-way variance model or the non-parametric Kruskal-Wallis test for quantitative variables and the chi-square test for categorical variables were used; p values <0.05 indicated statistical significance. Results: Safety culture was fragile (⩽50%) in the three hospitals, in the dimensions Management support for patient safety; General perception of patient safety; Return of information and communication about the error; Adequacy of professionals; Change of duty/shifts and transfers and Non-punitive error responses. The teamwork within the units was identified as strengthened in the private hospital; the general Cronbach's Alpha shoed the reliability of the instrument. The translated surgical module was considered valid, reliable and with satisfactory semantic, idiomatic, conceptual, cultural and content equivalences. The mean prevalence of the events was 21%, 14.2% and 12.7% (p=0.044), and for the readmissions, 9.8%; 29.4% and 45.2% (p=0.003), respectively, in Hospitals A, B and C, showing statistical difference. Most of the events were considered preventable, resulting in mild and/or moderate damage; Surgical Site Infection, Septicemia/Septic Shock, Hematoma/Septic Shock, Suture Dehiscence, Fistulas and Urinary Tract Infection were prevalent. Conclusion: The fragile dimensions of the safety culture are expressive and show priorities for its strengthening. The translation and adaptation of the surgical module contributed to the tracking of adverse events and is an applicable instrument in Brazil; the screening method evidenced a high prevalence of surgical adverse events, pointing out the importance of the theme and the relevance of strategies to promote surgical safety and hospital care quality.

Key-words: Patient Safety; Organizational Culture; Postoperative Complications; Validation Studies; Quality of Health Care.

LISTA DE FIGURAS

FIGURA 1 – MODELO ADAPTADO DO QUEIJO SUIÇO DE JAMES REASON DE COMO DEFESAS E BARREIRAS PODEM SER PENETRADAS POR UMA TRAJETÓRIA DE ERRO..37
FIGURA 2 – FÓRMULA PERCENTUAL DE RESPOSTAS POSITIVAS PARA AS DIMENSÕES..73
FIGURA 3 – FÓRMULA PERCENTUAL DE RESPOSTAS POSITIVAS PARA OS ITENS DAS DIMENSÕES..73
FIGURA 4 – FÓRMULA PARA O CÁLCULO AMOSTRAL 75
FIGURA 5 – FÓRMULA PARA O CÁLCULO DO ÍNDICE DE VALIDADE DE CONTEÚDO...88

LISTA DE FLUXOGRAMAS

FLUXOGRAMA 1 – METODOLOGIA PARA AVALIAÇÃO DA CULTURA DE SEGURANÇA ORGANIZACIONAL – GRUPO 1...77

FLUXOGRAMA 2 – METODOLOGIA PARA AVALIAÇÃO DE EVENTOS ADVERSOS CIRÚRGICOS – GRUPO 2..81

FLUXOGRAMA 3 – ETAPAS METODOLÓGICAS DO PROCESSO DE TRADUÇÃO, ADAPTAÇÃO TRANSCULTURAL E VALIDAÇÃO DE CONTEÚDO DO MÓDULO CIRÚRGICO DO *GLOBAL TRIGGER TOOL* PARA USO NO BRASIL.....................90

FLUXOGRAMA 4 – PREVALÊNCIA E EVITABILIDADE DE EVENTOS ADVERSOS EM TRÊS HOSPITAIS BRASILEIROS. 106

LISTA DE GRÁFICOS

GRÁFICO 1 – FREQUÊNCIA DAS AVALIAÇÕES NA DIMENSÃO RESPOSTAS NÃO PUNITIVAS AO ERRO. 99
GRÁFICO 2 – NOTA GERAL DE SEGURANÇA DO PACIENTE. 101
GRÁFICO 3 – NÚMERO DE EVENTOS NOTIFICADOS NOS TRÊS HOSPITAIS. 103
GRÁFICO 4 – EVENTOS ADVERSOS NO HOSPITAL A. 123
GRÁFICO 5 – EVENTOS ADVERSOS NO HOSPITAL B. 124
GRÁFICO 6 – EVENTOS ADVERSOS NO HOSPITAL C. 125

LISTA DE QUADROS

QUADRO 1 – TRAGETÓRIA HISTÓRICA DA SEGURANÇA DO PACIENTE NO BRASIL E NO MUNDO. 33
QUADRO 2 – DIMENSÕES DA CULTURA ORGANIZACIONAL. 44
QUADRO 3 – DOMÍNIOS DA CULTURA DE SEGURANÇA NAS ORGANIZAÇÕES 47
QUADRO 4 – DIMENSÕES DA CULTURA DE SEGURANÇA CONFORME *HSOPSC* VERSÃO BRASILEIRA. 60
QUADRO 5 – INFORMAÇÕES GERAIS DOS HOSPITAIS PARTICIPANTES DA PESQUISA. 67
QUADRO 6 – MÓDULO CIRÚRGICO *GLOBAL TRIGGER TOOL* TRADUZIDO E ADAPTADO PARA O PORTUGUÊS DO BRASIL PARA MENSURAÇÃO DE EVENTOS ADVERSOS. 132
QUADRO 7 – ORIENTAÇÕES DO MÓDULO CIRÚRGICO *GLOBAL TRIGGER TOOL* PARA O PORTUGUÊS DO BRASIL PARA MENSURAÇÃO DE EVENTOS ADVERSOS. 133

LISTA DE TABELAS

TABELA 1 – DISTRIBUIÇÃO DAS VARIÁVEIS SÓCIODEMOGRÁFICAS E LABORAIS DOS PROFISSIONAIS DE SAÚDE. CURITIBA–PARANÁ, 2017-2018 92
TABELA 2 – DISTRIBUIÇÃO DAS FREQUÊNCIAS DAS RESPOSTAS POR GRUPO PROFISSIONAL EM RELAÇÃO ÀS DIMENSÕES DA CULTURA DE SEGURANÇA ORGANIZACIONAL. CURITIBA–PARANÁ, 2017-2018 94
TABELA 3 – DIMENSÕES DE CULTURA DE SEGURANÇA E O PERCENTUAL DE RESPOSTA POSITIVA POR GRUPO PROFISSIONAL. CURITIBA–PARANÁ, 2017-2018 96
TABELA 4 – ESCORES DA DIMENSÃO DAS RESPOSTAS NÃO PUNITIVAS AO ERRO. CURITIBA–PARANÁ, 2017-2018 98
TABELA 5 – ASSOCIAÇÃO DA NOTA DE SEGURANÇA DO PACIENTE NOS HOSPITAIS E CATEGORIAS PROFISSIONAIS. CURITIBA–PARANÁ, 2017-2018 100
TABELA 6 – ASSOCIAÇÃO DO NÚMERO DE EVENTOS NOTIFICADOS NOS TRÊS HOSPITAIS POR GRUPO PROFISSIONAL. CURITIBA–PARANÁ, 2017-2018 102
TABELA 7 – DIMENSÕES DA CULTURA DE SEGURANÇA ORGANIZACIONAL E OS VALORES DO COEFICIENTE *ALFA DE CRONBACH*. CURITIBA–PARANÁ, 2017-2018 104
TABELA 8 – CRITÉRIOS POSITIVOS PARA RASTREAMENTO DE POTENCIAIS EVENTOS ADVERSOS CONFORME PROTOCOLO *CANADIAN ADVERSE EVENTS STUDY*. CURITIBA–PARANÁ, 2019......108
TABELA 9 – CRITÉRIOS POSITIVOS PARA RASTREAMENTO DE POTENCIAIS EVENTOS ADVERSOS CONFORME PROTOCOLO *GLOBAL TRIGGER TOOL*. CURITIBA–PARANÁ, 2019 110
TABELA 10 – DISTRIBUIÇÃO DO NÚMERO DE EVENTOS ADVERSOS POR PACIENTE, GRAU DE DANO E EVITABILIDADE. CURITIBA–PARANÁ, 2019.....111
TABELA 11 – PERFIL DEMOGRÁFICO E CLÍNICO DOS PACIENTES COM EVENTOS ADVERSOS. CURITIBA–PARANÁ, 2019 113
TABELA 12 – DISTRIBUIÇÃO DAS VARIÁVEIS RELATIVAS À INTERNAÇÃO DOS PACIENTES COM EVENTOS ADVERSOS. CURITIBA–PARANÁ, 2019 114

TABELA 13 – DISTRIBUIÇÃO DAS VARIÁVEIS RELATIVAS AO PROCEDIMENTO CIRÚRGICO E ANESTÉSICO DOS PACIENTES COM EVENTOS ADVERSOS. CURITIBA–PARANÁ, 2019 116
TABELA 14 – DISTRIBUIÇÃO DA FREQUÊNCIA DOS PROCEDIMENTOS CIRÚRGICOS NO HOSPITAL A. CURITIBA–PARANÁ, 2019 118
TABELA 15 – DISTRIBUIÇÃO DA FREQUÊNCIA DOS PROCEDIMENTOS CIRÚRGICOS NO HOSPITAL B. CURITIBA–PARANÁ, 2019 119
TABELA 16 – DISTRIBUIÇÃO DA FREQUÊNCIA DOS PROCEDIMENTOS CIRÚRGICOS NO HOSPITAL C. CURITIBA–PARANÁ, 2019 121
TABELA 17 – CONCORDÂNCIA ENTRE OS ESPECIALISTAS (N=6) E IVC OBTIDO NO PROCESSO DE VALIDAÇÃO DO PROCESSO DE TRADUÇÃO E ADAPTAÇÃO TRANSCULTURAL DO MÓDULO CIRÚRGICO DO GTT DO IHI. CURITIBA–PARANÁ, 2019 130
TABELA 18 – CONCORDÂNCIA ENTRE OS ESPECIALISTAS (N=6) E IVC OBTIDO NO PROCESSO DE VALIDAÇÃO DAS EQUIVALÊNCIAS SEMÂNTICA, IDIOMÁTICA, CONCEITUAL, CULTURAL E CONTEÚDO. CURITIBA–PARANÁ, 2019 131

LISTA DE SIGLAS

AHRQ - *Agency for Healthcare Research and Quality* (Agência para Pesquisa e Qualidade do Cuidado à Saúde)
ANVISA - Agência Nacional de Vigilância Sanitária
BVS - Biblioteca Virtual de Saúde
CAES - *Canadian Adverse Event Study* (Estudo Canadense de Eventos Adversos)
CEP - Comitê de Ética em Pesquisa
COREN/SP - Conselho Regional de Enfermagem / São Paulo
COFEN - Conselho Federal de Enfermagem
DATASUS - Departamento de Informatica do Sistema Único de Saúde
DVE - Derivação ventricular externa
EA - Evento Adverso
GTT - *Global Trigger Tool* (Ferramenta de Gatilho Global)
HC/UFPR - Hospital de Clínicas da Universidade Federal do Paraná
HSOPSC - *Hospital Survey on Patient Safety Culture* (Pesquisa Hospitalar sobre Cultura de Segurança do Paciente)
ICPS -*International Classification for Patient Safety* (Classificação Internacional de Segurança de Paciente)
IHI - *Institute for Healthcare Improvement* (Instituto para Melhoria da Saúde)
IOM - Institute of Medicine (Instituto de Medicina)
IRAS - Infecções Relacionadas à Assistência à Saúde
ISC - Infecção de Sítio Cirúrgico
JCAHO -*Joint Comission for the Accreditation of Hospital Organizations* (Comissão Conjunta para Acreditação de Organizações Hospitalares)
JCI - *Joint Comission International* (Comissão Conjunta Internacional)
OMS - Organização Mundial da Saúde
OPAS - Organização Pan-Americana da Saúde
PCSSV - Programa Cirurgia Segura Salvam Vidas
PPGENF - Programa de Pós-Graduação em Enfermagem
OMS - Organização Mundial da Saúde
PNSP - Programa Nacional de Segurança do Paciente

LISTA DE SIGLAS

REBRAENSP- Rede Brasileira de Enfermagem e Segurança do Paciente
RIENSP - Rede Internacional de Enfermagem e Segurança do Paciente
TEV - Tromboembolismo Venoso
SAS - Secretaria de Atenção à Saúde
SciELO - *Scientific Electronic Library Online* (Biblioteca Eletrônica Científica Online)
SP - Segurança do Paciente
SUS - Sistema Único de Saúde
TCLE - Termo de Consentimento Livre e Esclarecido

SUMÁRIO

1 INTRODUÇÃO ... 22
2. OBJETIVOS ... 29
2.1 OBJETIVO GERAL ... 29
2.2 OBJETIVOS ESPECÍFICOS ... 29
3 REVISÃO DE LITERATURA ... 30
3.1 SEGURANÇA DO PACIENTE ... 30
3.1.1 Erros, incidentes e eventos adversos ... 36
3.2 CULTURA DE SEGURANÇA ... 41
3.2.1 Cultura Organizacional ... 42
3.2.2 Cultura de segurança do paciente ... 45
3.3 EVENTOS ADVERSOS CIRÚRGICOS ... 53
3.4 METODOLOGIAS E INSTRUMENTOS PARA AVALIAR A CULTURA DE SEGURANÇA E EVENTOS ADVERSOS CIRÚRGICOS ... 60
3.4.1 *Hospital Survey on Patient Safety Culture - HSOPSC* ... 61
3.4.2 Revisão retrospectiva de prontuários ... 64
3.4.3 Metodologia *Global Trigger Tool* ... 65
3.4.4 Protocolo *Canadian Adverse Events Study (CAES)* ... 65
4 METODOLOGIA ... 67
4.1 CARACTERIZAÇÃO DA PESQUISA ... 67
4.2 LOCAL DO ESTUDO ... 68
4.3 CULTURA DE SEGURANÇA – GRUPO 1 ... 73
4.3.1 População do estudo ... 73
4.3.2 Critérios de inclusão e exclusão ... 73
4.3.3 Coleta de dados ... 74
4.3.4 Análise dos dados ... 75
4.4 EVENTOS ADVERSOS CIRÚRGICOS – GRUPO 2 ... 78
4.4.1 População do estudo e Amostra ... 78
4.4.2 Critérios de inclusão e exclusão ... 79
4.4.3 Coleta de Dados ... 79
4.4.4 Análise de Dados ... 81
4.4.5 Análises estatísticas ... 82

4.5 TRADUÇÃO PARA O PORTUGUÊS DO BRASIL E ADAPTAÇÃO TRANSCULTURAL DO MÓDULO CIRÚRGICO DO *GLOBAL TRIGGER TOOL DO INSTITUTE OF HEALTHCARE IMPROVEMENT*. 82
4.5.1 Tipo de pesquisa 82
4.5.2 Local e período de realização 83
4.5.3 Participantes..83
4.5.4 Coleta de dados 85
4.5.5 Procedimentos de análise de dados 87
4.6 ASPECTOS ÉTICOS...91
5 RESULTADOS.. 92
5.1 CULTURA DE SEGURANÇA..92
5.1.1 Características sóciodemográficas e laborais dos participantes 92
5.1.2 Dimensões e variáveis de resultado da cultura de segurança do paciente nos hospitais participantes 93
5.1.3 Associação dos hospitais em relação à nota de segurança e número de eventos notificados...100
5.1.4 Propriedades psicométricas do instrumento de pesquisa 103
5.2 EVENTOS ADVERSOS CIRÚRGICOS...105
5.2.1 Prevalência, evitabilidade, grau de dano e local de ocorrência dos eventos adversos cirúrgicos 105
5.2.2 Rastreamento de potenciais eventos adversos da Fase 1. 106
5.2.3 Confirmação de eventos adversos da Fase 2 111
5.2.4 Características demográficas e clínicas dos pacientes com eventos adversos cirúrgicos 112
5.2.5 Características da internação e procedimento cirúrgico-anestésico dos pacientes com eventos adversos cirúrgicos 114
5.2.6 Classificação dos eventos adversos cirúrgicos e tipo de complicação cirúrgica...122
5.3 TRADUÇÃO, ADAPTAÇÃO TRANSCULTURAL E VALIDAÇÃO DO MÓDULO CIRÚRGICO *GLOBAL TRIGGER TOLL* 128
6 DISCUSSÃO 134
6.1 CULTURA DE SEGURANÇA..134
6.2 EVENTOS ADVERSOS CIRÚRGICOS...155

6.3 TRADUÇÃO, ADAPTAÇÃO TRANSCULTURAL E VALIDAÇÃO DO MÓDULO CIRÚRGICO *GLOBAL TRIGGER TOOL DO INSTITUTE OF HEALTHCARE IMPROVEMENT*...195
7 CONCLUSÃO 202
REFERÊNCIAS.. 206
APÊNDICE 1 – TERMO DE CONSENTIMENTO LIVRE E ESCLARECIDO..... 257
APÊNDICE 2 – PERFIL DEMOGRÁFICO, CIRÚRGICO E ANESTÉSICO DOS PACIENTES.. 260
APÊNDICE 3 – SOLICITAÇÃO DE AUTORIZAÇÃO PARA TRADUÇÃO, ADAPTAÇÃO TRANSCULTURAL E VALIDAÇÃO 261
APÊNDICE 4 – QUESTIONÁRIO PARA AVALIAÇÃO E VALIDAÇÃO DE INSTRUMENTO - FORMULÁRIO PARA ESPECIALISTA............................... 262
APÊNDICE 5 – E-MAIL ENVIADO AOS DESENVOLVEDORES REFERENTE À FINALIZAÇÃO DO PROCESSO DE TRADUÇÃO, ADAPTAÇÃO E VALIDAÇÃO 265
APÊNDICE 6 – SUGESTÕES DOS ESPECIALISTAS SOBRE A TRADUÇÃO E ADAPTAÇÃO TRANSCULTURAL DOS TERMOS E FRASES DO MÓDULO CIRÚRGICO GLOBAL TRIGGER TOLL. 266
ANEXO A – QUESTIONÁRIO HOSPITAL SURVEY ON PATIENT SAFETY CULTURE – HSOPSC 267
ANEXO B – CRITÉRIOS DE RASTREAMENTO DE POTENCIAIS EVENTOS ADVERSOS CIRÚRGICOS 271
ANEXO C – CRITÉRIOS DE RASTREAMENTO EVENTOS ADVERSOS – MÓDULO CIRÚRGICO GLOBAL TRIGGER TOOL... 273
ANEXO D – FORMULÁRIO DE INVESTIGAÇÃO DE EVENTOS ADVERSOS 274
ANEXO E – FORMULÁRIO DE AVALIAÇÃO E EVITABILIDADE DE EVENTO ADVERSO CIRÚRGICO 276
ANEXO F – FORMULÁRIO DE AVALIAÇÃO DA CLASSIFICAÇÃO DO DANO DO EVENTO ADVERSO.. 277
ANEXO G – EMAIL DE AUTORIZAÇÃO PARA REALIZAÇÃO DO PROCESSO DE TRADUÇÃO, ADAPTAÇÃO TRANSCULTURAL E VALIDAÇÃO.................... 278
ANEXO H – E-MAIL RESPOSTA DO DESENVOLVEDOR DO INSTRUMENTO APÓS A FINALIZAÇÃO DO PROCESSO DE TRADUÇÃO, ADAPTAÇÃO TRANSCULTURAL E VALIDAÇÃO.. 279
ANEXO I – PARECER CONSUBSTANCIADO CEP HOSPITAL A. 280
ANEXO J – PARECER COMITÊ DE ÉTICA EM PESQUISA HOSPITAL B. 284
ANEXO K – PARECER COMITÊ DE ÉTICA EM PESQUISA HOSPITAL C..... 290

1 INTRODUÇÃO

O caminho em direção à segurança do paciente (SP) requer vigilância constante e não pode ser meramente uma prioridade estratégica, mas deve se tornar valor central na estrutura das organizações. A cultura de segurança requer envolvimento e comprometimento de toda a equipe de profissionais, reconhecimento dos erros que levam às mudanças positivas e sustentáveis, comportamentos respeitosos e valorização da segurança física e psicológica dos pacientes e da equipe de trabalho (AMERICAN COLLEGE OF HEALTHCARE EXECUTIVES (ACHE); INSTITUTE HEALTHCARE IMPROVEMENT (IHI); NATIONAL PATIENT SAFETY FOUNDATION (NPSF); LUCIAN LEAPE INSTITUTE, 2017).

A cultura de segurança é compreendida como valores, atitudes, competências e padrões de comportamento individuais e coletivos que determinam o compromisso, o estilo e a proficiência da gestão para o desenvolvimento de organização segura. Apesar dos avanços históricos, os danos evitáveis aos pacientes continuam, inaceitavelmente, frequentes, e a SP persiste como desafio para gestores e profissionais de saúde (ORGANIZAÇÃO MUNDIAL DA SAÚDE OMS, 2009), bem como permanece como importante problema de saúde pública (NPSF, 2015) representado por custos, mensuráveis e imensuráveis.

Eventos Adversos (EA) são erros que ocorrem durante a prestação do cuidado à saúde e que resultam em dano ao paciente, o qual pode ser físico, social e psicológico, além de incluir todo tipo de lesão, sofrimento, incapacidade e morte (WORLD HEALTH ORGANIZATION (WHO), 2009a; BRASIL, 2013a). Incidentes que resultam em danos, não relacionados à evolução natural da doença de base do paciente, são reconhecidos como agravos não intencionais e podem ocasionar lesões mensuráveis nos pacientes e/ou prolongamento do tempo de atendimento e internação hospitalar, com probabilidade de prognóstico desfavorável (BRASIL, 2013a). Para o monitoramento e a investigação de EA é necessário o estabelecimento da cultura de segurança nos serviços de saúde (BRASIL, 2017a).

Após a publicação do relatório *To err is human* publicado pelo *Institute of Medicine (*1999), os erros ocorridos nas instituições de saúde ganharam relevância, pela divulgação da morte de um milhão de pacientes que sofrem algum tipo de dano, além da ocorrência de 44.000 e 98.000 mortes/ano resultantes de assistência inadequada. Verificou-se a necessidade do fortalecimento de cultura de segurança

em termos organizacionais, como medida fundamental ao processo de melhoria da SP no contexto hospitalar (KOHN; CORRIGAN; DONALDSON, 2000).

Estudo que discute as conclusões do relatório da *National Patient Safety Foundation,* o qual investiga a SP nos 15 anos após a publicação deste relatório descreve que a análise da cultura de segurança persiste como fundamental para a segurança do sistema em organizações de saúde. Lideranças devem desenvolver ações, tais como: estabelecimento e manutenção de cultura de segurança nos cuidados assistenciais, incentivo à honestidade, e promoção da aprendizagem, responsabilidade individual e organizacional (GANDHI; BERWICK; SHOJANIA, 2016).

Deste modo, acredita-se que para garantir a SP e a qualidade da assistência é fundamental que ocorram mudanças na cultura do cuidado em saúde. Os erros não devem ser percebidos como o resultado da incompetência do profissional, mas, sobretudo, como oportunidades para melhorar o sistema. (REIS; LAGUARDI; MARTINS, 2012).

As características da cultura de segurança sólida incluem o reconhecimento da inevitabilidade do erro, a identificação proativa das ameaças latentes e a incorporação de sistema não punitivo para relato e análise dos EA (URBANETTO; GERHARDT, 2013). A cultura de segurança positiva, portanto, favorece práticas seguras, por meio das melhorias na comunicação, trabalho em equipe e troca de conhecimentos. No Brasil, não existe diagnóstico amplo sobre os problemas de SP em hospitais, e o aumento dos EA evitáveis demonstra a necessidade do fortalecimento da cultura de segurança entre os profissionais (REIS; MARTINS; LAGUARDIA, 2013a).

O estímulo à cultura de segurança do paciente e a implantação de metas para a prevenção de EA tornaram-se grande desafio com vistas ao aprimoramento da qualidade na saúde, e são necessárias para melhoria dos sistemas (COSTA; RAMOS; GABRIEL et al., 2018). Dessa forma, os serviços devem estruturar o sistema de forma segura e desenvolver estratégias para detectar a ocorrência de EA para identificar as possíveis causas que induzem à ocorrência de falhas, classificar a intensidade e a ocorrência do dano ao paciente e à instituição e, consequentemente, implementar ações corretivas para sua prevenção (SILVA; ALVES; SANCHES et al, 2016).

Faltam informações sobre EA e seus fatores causais, fato que dificulta o conhecimento, avaliação e discussão sobre as consequências destes eventos para os profissionais, usuários e familiares (SILVA, 2010). Neste sentido, a avaliação da cultura de segurança em hospitais é considerada como indicador estrutural básico que viabiliza iniciativas para reduzir os riscos e EA (SILVA; ROSA, 2016).

Em consonância com os estudos apresentados, acredita-se que a falta de informações prejudica a realização de planejamento e desenvolvimento de estratégias organizacionais voltadas ao fortalecimento da cultura de segurança, minimização de EA e promoção da qualidade da assistência. Compreender o cenário da organização de saúde, a partir da avaliação da cultura de segurança, é ponto de partida para traçar ações em prol de mudanças na redução da incidência de EA e garantia de cuidados de saúde seguros (TIMM; RODRIGUES, 2016).

Em estudo de Revisão Integrativa de Literatura, com o objetivo de identificar as principais temáticas abordadas em ambiente hospitalar para a SP, de 2009 até 2015, verificou-se que 22,22% dos artigos estavam relacionados à ocorrência de EA. Nesse contexto, apesar dos avanços na área de saúde, a SP é influenciada pelos profissionais de saúde por meio da ocorrência de erros que se refletem, diretamente, na qualidade de vida dos pacientes. Os pesquisadores sugerem a execução de outros estudos para promover a qualidade da assistência prestada, por meio do controle e prevenção de EA (SILVA; ALVES; SANCHES et al., 2016).

Em 2018 foi publicado estudo multicêntrico brasileiro sobre EA relacionados à assistência hospitalar, com análise de 445.671 pacientes e 182 hospitais, distribuídos em 13 estados. Os principais resultados mostram que a mortalidade, associada aos EA graves, foi de 37% entre pacientes do Sistema Único de Saúde (SUS) e de 28,8% em saúde suplementar. Destaca-se que entre 30 a 36% dos óbitos poderiam ter sido evitados (COUTO; PEDROSA; ROBERTO et al., 2018).

Em relação aos EA cirúrgicos, dados da OMS pressupõem que sejam realizadas 234 milhões de cirurgias no mundo, que causam seis milhões de óbitos e sete milhões de EA, mas 50% desses seriam evitáveis. Estima-se que entre as cirurgias de alta complexidade, realizadas em países desenvolvidos, cerca de 3 a 16% apresentam complicações cirúrgicas graves, o que indica sete milhões de situações incapacitantes, e com índices de mortalidade entre 0,4 a 0,8%. Neste contexto, a SP

cirúrgico é considerada componente crítico da qualidade do cuidado em saúde. (ORGANIZAÇÃO MUNDIAL DA SAÚDE (OMS), 2009).

Portanto, falhas na segurança dos processos cirúrgicos causam EA sérios, como cirurgia realizada em local ou lado inadequado, em paciente errado, retenção de objeto estranho no organismo do paciente e morte no intraoperatório ou pós-operatório imediato, ocorrências que justificam a importância da implantação de protocolos para sua prevenção (ORGANIZAÇÃO MUNDIAL DA SAÚDE (OMS), 2009).

Os estudos na área da SP possibilitam o reconhecimento de fatores relacionados ao processo de cuidado, desenvolvimento de estratégias e adoção de práticas seguras que minimizam os riscos e a ocorrência de EA nos pacientes cirúrgicos. Essas, atingidas por meio do envolvimento das instituições de saúde no monitoramento das ocorrências e tratamento das informações, além de tornar eficaz a notificação aos órgãos governamentais. (CAPUCHO; CASIANNI, 2013).

No Brasil, para a notificação dos incidentes, EA e queixas técnicas relacionadas ao uso de produtos e serviços de assistência à saúde, foi criado o Sistema Nacional de Notificações para Vigilância Sanitária, denominado NOTIVISA, ancorado no portal da Agência Nacional de Vigilância Sanitária (ANVISA). Trata-se de sistema informatizado na plataforma web, previsto pela Portaria nº 1660 de 22 de julho de 2009, Portaria nº 529 de 1º de abril de 2013 do Ministério da Saúde e pela RDC nº 36 de 25 de julho de 2013 da ANVISA. Desde junho de 2014 as notificações tornaram-se obrigatórias e devem ser registradas. (BRASIL, 2019).

Essa obrigatoriedade se aplica aos profissionais de serviços de saúde (hospitais, clínicas, hemocentros, laboratórios, Núcleos de Segurança do Paciente, entre outros); profissionais/técnicos da ANVISA, das Vigilâncias Sanitárias Estaduais e Municipais, das Secretarias Estaduais e Municipais de Saúde; profissionais que atuam em drogarias e farmácias; Laboratórios de Saúde Pública, Univesidades/Centros de pesquisa; empresas detentoras de registro de produtos sob vigilância sanitária (fabricantes, importadores e distribuidores) e profissionais de saúde liberais (BRASIL, 2019).

No período de janeiro a dezembro de 2016 foram notificados 53.997 (100%) incidentes relacionados à assistência à saúde ao NOTIVISA, 50.735 (94%) ocorreram em hospitais. Destes, 26.977 (53,2%) ocorreram em setores de internação e 2.028 (4%) em Centros Cirúrgicos. Os incidentes classificados como

"falhas durante o procedimento cirúrgico" somaram 197 casos e os classificados como "outros", mas que envolveram cirurgias, atingiram 375 pacientes (BRASIL, 2018a).

Neste contexto, em relação aos EA cirúrgicos, outros dados do Departamento de Informática do Sistema Único de Saúde (DATASUS)[1], registrados de janeiro a dezembro de 2017, demonstram 4.512.917 internações para a realização de procedimentos cirúrgicos. Do total de EA notificados, verifica-se que 9.928 ocorreram em centros cirúrgicos e o número de *never events* (eventos que nunca poderiam ocorrer na assistência à saúde) representaram 17 procedimentos cirúrgicos realizados em local errado, 21 no lado errado do corpo, 4 no paciente errado, 16 cirurgias erradas em um paciente; 119 retenções não intencionais de corpo estranho e 28 óbitos intra−operatórios, ou imediatamente pós−operatório em paciente classificado como ASA 1[2]. (BRASIL, 2018b).

Estudo brasileiro realizado em três hospitais do Rio de Janeiro demostrou que os procedimentos cirúrgicos estão no *ranking* das ocorrências de EA (MENDES; MARTINS; ROZENFELD et al., 2009). Identificou-se incidência de 3,5% de EA cirúrgicos e 68,3% foram considerados evitáveis (MOURA; MENDES, 2012). Outro estudo evidenciou que 20% dos EA corresponderam à complicações cirúrgicas e/ou anestésicas (MENDES; PAVÃO; MARTINS et al., 2013).

Para desenvolvimento e execução de metodologia para investigação de incidentes relacionados aos cuidados em saúde é necessário desenvolvimento de lógica sistemática e os dados devem ser baseados em evidências. Podem ser utilizados como fontes de informações: entrevistas, prontuários médicos e outros históricos de intervenções; as ferramentas utilizadas devem responder às seguintes perguntas: o que aconteceu; quando aconteceu e onde aconteceu (BRASIL, 2017a).

Particularmente, para esta pesquisa, há interesse na SP cirúrgico, considerado de alto risco para a ocorrência de EA, pois centros cirúrgicos são locais cujos pacientes estão extremamente suscetíveis a erros e à ocorrência de grande parte de EA, em virtude da complexidade e riscos de complicações intra e pós-operatórias, inclusive de morte (SECANELL; ORREGO; VILA et al., 2016). Prestar

---

[1] DATASUS surgiu em 1991 e tem como responsabilidade prover os órgãos do SUS de sistemas de informação e suporte de informática, necessários ao processo de planejamento, operação e controle. (BRASIL, 2018b).

[2] ASA 1 refere-se a paciente saudável, conforme classificação do estado fisiológico do paciente relacionado ao risco cirúrgico. (DOYLE; GARMON, 2017).

assistência segura e de qualidade nesse ambiente é um desafio pois, as atividades são complexas, interdisciplinares, com forte dependência da atuação individual e desenvolvidas em ambiente considerado de alto risco para a ocorrência de EA (KELLICUT; KUNCIR; WILLIAMSON et al., 2014).

Estudo realizado em três hospitais na Região Nordeste do Brasil, para avaliar a cultura de segurança, sugere a realização de novas pesquisas que englobem hospitais de outras regiões do país, com a finalidade de melhorar a representatividade quantitativa e qualitativa dos achados (ANDRADE; LOPES; SOUZA FILHO et al., 2018). Neste sentido, o Ministério da Saúde brasileiro aponta como relevante a avaliação da cultura de segurança por meio de instrumentos reconhecidos e validados, com vistas à elaboração de um plano para SP que possa contribuir para a redução dos incidentes e EA, incluindo os relacionados à cirurgia (BRASIL, 2014a).

Portanto, esta pesquisa se propôs a avaliar dados relacionados à percepção dos profissionais de saúde sobre a cultura de segurança e investigar a prevalência de EA cirúrgicos, com utilização de metodologia de pesquisa específica. Tais dados são imprescindíveis para o diagnóstico sólido do cuidado prestado, promoção de melhorias nos processos de trabalho no desenvolvimento de cultura de segurança do paciente positiva, que podem nortear ações que priorizam práticas seguras na prevenção de EA em hospitais (EL-JARDALI; SHEIKH; GARCIA et al., 2014).

A realização deste estudo, pois acredita-se que o desenvolvimento de metodologia de pesquisa específica, que adapta instrumentos cientificamente validados, pode mostrar caminhos para a investigação sobre a cultura de segurança em hospitais, e a incidência de EA cirúrgicos. E, desse modo, ter a finalidade do mapeamento real dos problemas, avaliação das causas, sob a perspectiva dos profissionais de saúde, e mensuração das taxas de ocorrência, potencial de evitabilidade e gravidade destes eventos.

Infere-se que compreender estes fenômenos pode ser importante passo para o desenvolvimento de cultura de segurança positiva nos hospitais, bem como, na elaboração de estratégias para prevenção de EA cirúrgicos, fortalecimento de políticas públicas e desenvolvimento de ações institucionais em prol da melhoria da qualidade dos processos assistenciais cirúrgicos.

Diante deste cenário, urge a necessidade da difusão de conhecimentos e ações sobre as questões que envolvem a SP nos serviços de saúde. Por este

motivo, esta pesquisa pretende somar-se aos resultados de estudos nacionais e internacionais, por meio da análise de três diferentes realidades institucionais hospitalares, em relação à avaliação da cultura de segurança e prevalência de EA cirúrgicos. Deste modo, pretende-se contribuir para a sensibilização de novos pesquisadores e profissionais de saúde sobre a importância do desenvolvimento da cultura de segurança e redução dos riscos e EA em pacientes submetidos a procedimentos cirúrgicos.

A partir destas considerações, a hipótese desta pesquisa é: a investigação da cultura de segurança e ocorrência de eventos adversos cirúrgicos oportuniza a identificação de fragilidades na segurança do paciente e subsidia o planejamento de ações para a sua promoção.

2. OBJETIVOS

2.1 OBJETIVO GERAL

- Avaliar a cultura de segurança do paciente e eventos adversos cirúrgicos em hospitais de ensino da região sul do Brasil.

2.2 OBJETIVOS ESPECÍFICOS

- Analisar as dimensões da cultura de segurança do paciente na perspectiva dos profissionais de saúde que prestam assistência aos pacientes cirúrgicos.
- Traduzir, adaptar transculturalmente e validar o módulo cirúrgico *Global Trigger Tool do Institute of Healthcare Improvement* para o português do Brasil.
- Identificar a prevalência de eventos adversos cirúrgicos e o potencial de evitabilidade e gravidade.

3 REVISÃO DE LITERATURA

Este capítulo apresenta estudos nacionais e internacionais sobre mensuração da cultura de segurança e prevalência de EA cirúrgicos e que orientaram a elaboração do desenho de metodologia específica para esta pesquisa. Realizou-se revisão da literatura que abordam os seguintes temas: Segurança do Paciente (conceitos, evolução histórica, erros, incidentes e eventos adversos resultantes do cuidado em saúde), Clima e Cultura de Segurança (conceitos e suas dimensões) e EA decorrentes da assistência cirúrgica.

Foram realizadas buscas sobre resoluções e portarias pertinentes à temática, além de livros, dissertações e teses. As informações foram obtidas por intermédio de artigos científicos publicados nas bases de dados Biblioteca Virtual de Saúde (BVS), *Scientific Electronic Library Online (SciELO), PubMed, Web of Science, Scopus e pelo “Google Scholar”* utilizando-se dos seguintes descritores: “segurança do paciente”, “clima de segurança”, “cultura de segurança”, “cultura organizacional” e “rastreador de eventos adversos”.

3.1 SEGURANÇA DO PACIENTE

A discussão sobre a ocorrência de erros é considerada alicerce do movimento em prol da SP. Neste contexto, os profissionais têm sido estimulados a comunicar e discutir com outros profissionais, e com a própria instituição, de maneira que cada evento seja analisado e que as recorrências sejam prevenidas. A incidência e as características de EA no âmbito nacional podem ser ótimos indicadores para políticas de melhoria de desempenho na assistência à saúde (BRASIL, 2017a; MENDES; PAVÃO; MARTINS et al., 2013).

Historicamente, desde o século XIX havia preocupação com a SP. Em 1855 Florence Nightingale, com base em dados de mortalidade das tropas britânicas, propôs mudanças organizacionais e na higiene dos hospitais, ações que resultaram na redução do número de óbitos dos pacientes internados (NEUHAUSER, 2003). Em 1858 escreveu *notes on matters affecting the health, efficiency, and hospital administration of the British Army* e registrou o número de homens que morreram devido às condições sanitárias inadequadas encontradas em hospitas em compara-

ração com os que morreram por feridas de batalha. Usou dados estatísticos para revelar a natureza da infecção nos hospitais e no campo de batalha. Além disso, organizou dados e evidências para estabelecer diretrizes para a reforma dos cuidados de saúde (ARAVIND; CHUNG, 2010).

Alguns anos depois, em 1859, Florence Nightingale escreveu *Notes on Hospitals* e relatou o pensamento hipocrático *primum non nocere*, que significa não cause dano e descreveu as condições sanitárias do hospital de Renkioi na cidade de Istambul, onde atuou durante a Guerra da Criméia. Nesta obra ela discorre sobre como deveria ser a construção e funcionamento seguro de hospitais para o cuidado aos pacientes. Em 1860, escreveu *Notes on Nursing: what it is, and what it is not,* e relatou os princípios básicos dos cuidados de enfermagem aos pacientes no ambiente hospitalar, redigido sobre a forma de manual com conhecimentos específicos para à profissão e com detalhamento das competências que as enfermeiras precisam desenvolver (FIGUEIREDO; PERES, 2019).

Porém, a questão da segurança foi evidenciada, mundialmente, a partir do ano 2000, com a publicação do relatório *To Err is Human: building a safer health care system* pelo *Institute of Medicine (IOM)*, que demonstrou números elevados de danos e mortes em decorrência de assistência inadequada (KOHN; CORRIGAN; DONALDSON, 2000). Esse relatório focou nas recomendações sobre como alterar a cultura hospitalar e o termo cultura de segurança, utilizado na energia nuclear por 30 anos, começa a ser discutido no campo da saúde (LONGO; HEWETT; GE et al., 2005). Um ano depois, o referido Instituto publicou o relatório *Crossing the Quality Chasm*, que priorizou o sistema de saúde e seis domínios: segurança, efetividade, foco no paciente, otimização, eficiência e equidade (BRASIL, 2013a).

Em âmbito nacional, as propostas da ANVISA têm como objetivo a melhoria da qualidade no atendimento hospitalar e adesão do Brasil à Aliança Mundial em prol da SP (WHO; 2008a; BRASIL, 2016). Esta agência desenvolve suas atividades no campo de serviços de saúde em parceria com o Ministério da Saúde e a Organização Pan-Americana da Saúde. Do mesmo modo, práticas de vigilância e monitoramento sobre o uso de sangue, saneantes, materiais, dispositivos, equipamentos e medicamentos, aliam-se à prevenção, vigilância e controle de incidentes, em busca de uma atenção qualificada à saúde (BRASIL, 2016).

A seguir, para descrever brevemente a trajetória histórica das ações em prol da SP no Brasil e no mundo, apresentam-se os principais acontecimentos em relação ao tema, conforme QUADRO 1.

QUADRO 1 – TRAGETÓRIA HISTÓRICA DA SEGURANÇA DO PACIENTE NO BRASIL E NO MUNDO.

(continua)

**ANO**	**HISTÓRICO SOBRE A SEGURANÇA DO PACIENTE NO BRASIL E NO MUNDO**
1911	Investigações científicas relacionadas à Segurança do Paciente iniciaram-se com o estudo do médico cirurgião Ernest Codman sobre as falhas ocorridas no tratamento de pacientes. (WACHTER, 2013).
1918	Criação da *Joint Commission on Accreditation of Healthcare Organizations*, pelo Colégio Americano de Cirurgiões e publicação do estudo *Diseases of Medical Progress*, que demonstrou a prevalência e a evitabilidade de doenças iatrogênicas, identificadas como desfecho não natural do diagnóstico inicial do paciente. (WACHTER, 2013).
1996	Criação da *National Patient Safety Foundation* pela *American Medical Association* com o objetivo de garantir a oferta de cuidado seguro aos pacientes. (KOHN; CORRIGAN; DONALDSON; 2000).
2000	Publicação do relatório *To Err is Human: building a safer health care system* pelo Institute of Medicine (IOM), marco importante sobre a ocorrência de EA com visibilidade no cenário mundial. (KOHN; CORRIGAN; DONALDSON; 2000).
2001	Criação da Rede Brasileira de Hospitais Sentinela, pela Agência Nacional de Vigilância Sanitária do Brasil, visando à fortalecer a gestão de risco, por meio de ações direcionadas pela busca ativa de eventos adversos, estimular sua notificação e o registro de queixas técnicas relacionadas à tecnovigilância, farmacovigilância e hemovigilância (BRASIL, 2014; DUARTE; STIPP MAC; SILVA et al., 2015).
2004	Criação da *The World Alliance for Patient Safety*, pela Organização Mundial de Saúde durante a 55ª Assembleia Mundial da Saúde. O propósito dessa iniciativa foi desafiar a comunidade científica na definição e identificação de pontos críticos na assistência à saúde, com o objetivo de desenvolver políticas mundiais para a prevenção de danos aos pacientes, promoção da qualidade dos serviços e Segurança do Paciente (WHO, 2008a).
2004	Desenvolvimento da International Classification for Patient Safety, com 48 conceitos-chaves, nas versões em inglês, espanhol e português. (WHO, 2009a). A versão em português foi traduzida e validada em Portugal como Estrutura Conceitual da Classificação Internacional sobre Segurança do Doente. (OMS, 2011).
2005	Designação da *Joint Commission on Accreditation of Healthcare Organizations* pela Organização Mundial da Saúde como primeiro centro colaborador dedicado à Segurança do Paciente e na certificação da qualidade em serviços de saúde. (JOINT COMITION INTERNATIONAL CENTER FOR PATIENT SAFETY, 2008).
2005 - 2006	Lançamento do Primeiro Desafio Global pela Organização Mundial da Saúde - Programa Cuidado Limpo é Cuidado Seguro, focando a prevenção das Infecções Relacionadas à Assistência à Saúde. (OMS, 2009a).
2005	Criação da Rede Internacional de Enfermagem e Segurança do Paciente , em Concepción – Chile pela Organização Pan-Americana da Saúde em 2005, com o objetivo de traçar tendências e prioridades no desenvolvimento da enfermagem na área da Segurança do Paciente, discutir a cooperação e o intercâmbio de informações entre os países e a necessidade de fortalecimento do cuidado de enfermagem, a partir de evidências científicas (CALDANA; GUIRARDELLO; URBANETTO et al., 2015).
2007	Lançamento das nove soluções para a Segurança do Paciente, são estratégias que contribuem para diminuição da ocorrência de eventos adversos: Identificação de medicamentos; Identificação de pacientes; Comunicação intersetorial; Procedimento correto; Controle de soluções com eletrólitos de alta concentração; Precisão da medicação; Prevenção de erros na conexão de cateteres e

QUADRO 1 – TRAGETÓRIA HISTÓRICA DA SEGURANÇA DO PACIENTE NO BRASIL E NO MUNDO.

.

(conclusão)

ANO	HISTÓRICO SOBRE A SEGURANÇA DO PACIENTE NO BRASIL E NO MUNDO
2007	tubos; Uso único de dispositivos para administração de drogas; Melhoria na adesão à higienização das mãos (WHO, 2007).
2007	Criação da Rede Nacional de Monitoramento da Resistência Microbiana, uma parceria da Agência Nacional de Vigilância Sanitária do Brasil com a Organização Pan-Americana de Saúde, com o objetivo de tornar a assistência à saúde mais efetiva por meio do uso racional de antimicrobianos e para detecção, prevenção e controle a resistência microbiana em serviços de saúde (BRASIL, 2017b).
2007 - 2008	Lançamento do Segundo Desafio Global pela Organização Mundial da Saúde- Programa Cirurgias Seguras Salvam Vidas visando a desenvolver práticas seguras para a realização de cirurgias seguras (OMS, 2009a; WHO, 2009b).
2008	Criação da Rede Brasileira de Enfermagem e Segurança do Paciente para desenvolvimento de estratégias, articulação e cooperação técnica entre instituições diretas e indiretamente ligadas à saúde e educação de profissionais, com o objetivo de fortalecer a assistência de enfermagem segura e com qualidade. (REDE BRASILEIRA DE ENFERMAGEM E SEGURANÇA DO PACIENTE (REBRAENSP, 2013).
2010	Apresentação no Brasil do Programa Cirurgias Seguras Salvam Vidas, pela Secretaria de Atenção à Saúde do Ministério da Saúde, com o objetivo de diminuir a morbimortalidade causada pelas intervenções cirúrgicas e elevar os padrões de qualidade em serviços de assistência à saúde. A estratégia consistiu em definir um conjunto básico de normas de segurança dirigidas à prevenção das infecções pós-cirúrgicas, a segurança dos procedimentos anestésicos e das equipes cirúrgicas e a mensuração dos indicadores cirúrgicos, semelhantemente ao Programa da Organização Mundial da Saúde (OMS, 2009; BRASIL, 2013a).
2011	Lançamento das seis metas internacionais para a Segurança do Paciente pela Organização Mundial da Saúde, em parceria com a *Joint Commission International*: identificar os pacientes corretamente; melhorar a comunicação entre os profissionais; melhorar a segurança de medicações de alta vigilância; assegurar a realização de cirurgias em local de intervenção, procedimento correto e paciente correto; reduzir o risco de infecções associados aos cuidados de saúde; reduzir o risco de lesões aos pacientes decorrentes de queda (BRASIL, 2014).
2013	Instituição, pelo governo brasileiro, da Portaria Ministerial nº 529 e da RDC nº 36 que dispõem sobre objetivos, estratégias e ações para a segurança e melhoria da qualidade nos serviços de saúde, que culminou na instituição do Programa Nacional de Segurança do Paciente. (BRASIL, 2013b, BRASIL, 2013c).O Programa define os conceitos relevantes e as principais estratégias para promoção e sistematização de ações em prol da Segurança do paciente no país: suporte à implementação de práticas seguras nos hospitais, criação de um sistema de notificação de incidentes, elaboração de protocolos, promoção de processos de capacitação, bem como determina a criação do Núcleo de Segurança do Paciente e desenvolvimento de planos estratégicos para ações de gestão de risco e estabelecimento da notificação dos eventos adversos pelas organizações de saúde (BRASIL, 2013c).
2017	Lançamento do Terceiro Desafio Global pela Organização Mundial da - Medicação Sem Danos, durante a segunda Cúpula Ministerial em Bonn, na Alemanha. O objetivo é conscientizar, propor soluções para garantir a segurança no uso de medicação, alcançar o engajamento e o compromisso dos estados membros e das instituições de saúde para reduzir os danos graves evitáveis associados à medicação em 50% globalmente nos próximos cinco anos (WHO, 2017).

FONTE: A autora (2019).

Os riscos aos pacientes são uma realidade constante e cabe às equipes envolvidas neste processo de trabalho em saúde propor estratégias e proporcionar barreiras de proteção em prol da SP (ROSCANI; FERRAZ; OLIVEIRA FILHO et al., 2015). A assistência de enfermagem é fundamental para melhoria da SP nas instituições brasileiras (SILVA; ALVES; SANCHES et al., 2016). Esses profissionais são responsáveis por grande parte das ações assistenciais e, portanto, encontram-se em posição privilegiada para reduzir a possibilidade de incidentes que atingem o paciente, além de detectar as complicações precocemente e realizar as condutas necessárias para minimizar os danos (PEDREIRA; 2009a; PEDREIRA, 2009b).

O cuidado é considerado a essência do trabalho da enfermagem e, como tal, precisa ser realizado sem causar dano, de modo a atender o cliente de maneira integral. Portanto, é responsabilidade dos profissionais e da instituição de saúde, garantir um cuidado seguro e de qualidade (FASSINI; HAHN, 2012). Deste modo, a prática de enfermagem em hospitais é afetada por vários elementos associados aos erros na assistência, tais como gravidade e complexidade das doenças, período de internação, número de atividades delegadas pelos enfermeiros aos técnicos e auxiliares de enfermagem, déficit de recursos humanos da equipe de enfermagem, sobrecarga de atividades, elevada rotatividade de pessoal e longas horas de trabalho (SIQUEIRA; CALIRI; KALISCH et al., 2013).

Tal fato é comprovado por estudo de revisão bibliográfica realizado para analisar a contribuição da enfermagem para a SP no Brasil, que verificou que a assistência de enfermagem é essencial para o processo de melhoria da SP. No entanto, este mesmo estudo identificou baixo conhecimento dos profissionais de saúde sobre ocorrência de EA e a forma como devem ser notificados, sentimentos como medo em relação aos erros durante o cuidado em saúde devido a política de punição e baixa adesão na higienização das mãos (SILVA; ALVES; SANCHES et al., 2016).

A influência da equipe de enfermagem para prevenção da ocorrência de EA cirúrgicos ao paciente se reflete no processo de trabalho por ações como verificação da identidade dos pacientes; confirmação das assinaturas nos termos de consentimento cirúrgico e anestésico; conferência do local, cirurgia e lateralidade do procedimento; verificação e monitoramento dos sinais vitais; manutenção de acesso venoso permeável para a prevenção de riscos potenciais, como perda sanguínea,

dificuldade de vias aéreas, reações alérgicas e complicações no pós-operatório (GRIGOLETO; GIMENES; AVELAR, 2011).

Neste sentido, outro estudo que analisou a associação entre a equipe de enfermagem e mortalidade de pacientes em hospitais ingleses concluiu que o número de enfermeiros por enfermaria está associado com a redução da mortalidade de pacientes clínicos. Estudos assim direcionaram políticas relacionadas á quantidade mínima obrigatória de enfermeiro por paciente em alguns estados norte americanos e australianos. As evidências apontam que quanto maior o nível de formação profissional e maior a força de trabalho de enfermagem altamente qualificada, menor será o número de ocorrência de EA (GRIFFITHS; BALL; MURRELLS et al., 2016).

Os erros ocasionados durante o atendimento prestado ao paciente apresentam repercussões para o indivíduo, instituições e sociedade. Deste modo, os órgãos de saúde estabelecem estratégias para a melhoria do cuidado, com a finalidade de prevenir e reduzir a incidência de EA, por meio da implementação de políticas públicas e iniciativas privadas em prol da SP (BRASIL, 2013b).

3.1.1 Erros, incidentes e eventos adversos

Conforme a *International Classification for Patient Safety (ICPS)* ou a versão portuguesa traduzida e validada, denominada Estrutura Conceitual da Classificação Internacional sobre Segurança do Doente (CISD), os incidentes relacionados ao cuidado podem ser divididos em *near miss* (não atingiu o paciente); incidente sem dano (atingiu o paciente, mas não causou dano) e incidente com dano (resulta em dano ao paciente), o qual é considerado EA (WHO, 2009a).

Com base nesta classificação, o erro consiste em falha na execução de uma ação planejada de acordo com o desejado, ou mesmo o desenvolvimento incorreto de um plano, e pode ocorrer por prática de ação errada ou por não se conseguir praticar a ação certa, quer seja na fase de planejamento ou de execução de qualquer procedimento (WHO, 2009a; OMS, 2011). O termo dano refere-se ao prejuízo na estrutura ou funções do corpo e/ou qualquer efeito pernicioso resultante que inclui doença, lesão, sofrimento, incapacidade ou morte (OMS, 2011).

Estudos sobre o erro humano procuram desvendar os motivos desencadeantes, com vistas à criação de estratégias para sua minimização. Para

compreender a perspectiva da ocorrência do erro, o modelo teórico de *James Reason* é o mais utilizado, conforme apresentado na FIGURA 1.

Os sistemas complexos possuem várias barreiras que atuam como fatores de proteção e, a referida Teoria faz analogia ao queijo suíço, onde as barreiras de segurança funcionam como as fatias do queijo suíço, porém estas possuem muitos buracos. A presença de um espaço, não necessariamente permite que o evento aconteça, mas o erro será resultado de falhas simultâneas nas diversas barreiras do sistema (REASON, 2000).

FIGURA 1 – MODELO ADAPTADO DO QUEIJO SUIÇO DE JAMES REASON DE COMO DEFESAS E BARREIRAS PODEM SER PENETRADAS POR UMA TRAJETÓRIA DE ERRO

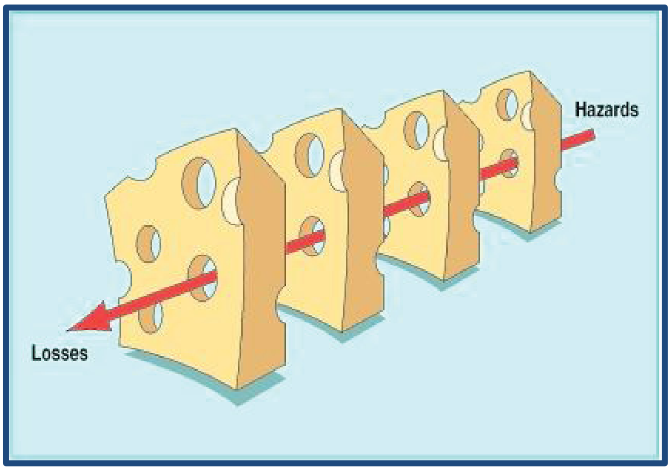

FONTE: REASON (2000).

O EA acontece quando os muitos “buracos do queijo suíço” operam continuamente na mesma linha, ficam momentaneamente alinhados, e permitem a trajetória do erro. O vetor representa que o risco não encontrou barreira e atingiu o paciente. Neste processo, o importante não é quem cometeu o erro, mas sim como e o porquê as defesas falharam. Existem barreiras de proteção no desenvolvimento dos processos de trabalho, mas quando as barreiras apresentam muitos furos, os sistemas se apresentam frágeis, os “furos” se alinham e geram o EA (REASON, 2000).

Os erros cometidos pelos profissionais de saúde, que podem originar danos graves em pacientes hospitalizados, são alvo de estudos para a criação de modelos que visam à eliminação do erro ou redução de suas consequências. Estes estudos auxiliam na compreensão das causas e fatores desencadeantes. Neste sentido, dois grupos de abordagem estão sendo desenvolvidos: o pessoal e o sistêmico. O

modelo pessoal verifica a causa do erro com foco na atuação individual, ou seja, procura identificar a ocorrência de negligência, desatenção, descuido, falta de conhecimento, falta de experiência, desmotivação. O modelo sistêmico compreende que existem causas sistêmicas relacionadas à ocorrência dos erros, reconhece a limitação humana e tem como objetivo antecipar-se ao erro e suas consequências (REASON, 2000).

O modelo sistêmico baseia-se nos conceitos de condições de falha latente e de falhas ativas. As falhas ativas são as ações inseguras cometidas pelos profissionais da linha de frente durante o cuidado (cansaço físico e mental, falta de atenção, baixa satisfação, falta de cuidado, negligência). As falhas latentes envolvem um desenho inadequado no sistema que afeta as condições de trabalho, como inexperiência dos profissionais, instalações inapropriadas, falta de manutenção dos equipamentos, más decisões gerenciais e organizações mal estruturadas. Neste sentido, nenhuma instituição de saúde está imune à ocorrência de EA, mas as organizações que utilizam o método sistêmico de abordagem do erro conseguem prevenir problemas (REASON, 2000).

Em consonância com os trabalhos do pesquisador James Reason, a OMS propõe trabalhar os erros com base nos modelos de barreiras, para impedir que estes erros atinjam o paciente. Cabe ao sistema organizar as barreiras de modo que as ações adotadas previnam a ocorrência de EA e promovam ações para melhoria dos processos que buscam à SP (OMS, 2009).

O cuidado em saúde é desenvolvido num ambiente dinâmico, envolve alta tecnologia, decisões individuais e julgamentos por parte dos profissionais de saúde. Porém, a maioria dos EA não é resultado da imprudência individual, mas sim a consequência de processos e sistemas defeituosos, que levam as pessoas a cometer erros, ou não agir corretamente para impedir sua ocorrência (KOHN; CORRIGAN; DONALDSON, 2000).

Existe diferença entre o erro e o evento adverso. O erro é um desvio de um procedimento que deveria ter sido correto e o evento adverso consiste em qualquer lesão provocada, ocorrida durante o cuidado de saúde, que causou dano ao paciente. Ambos ocorrem por deficiência de estrutura, por péssimas condições de trabalho nas instituições, por falta de medicações, treinamento inadequado de recursos humanos ou pela ausência de recursos necessários ao financiamento do tratamento. (BRASIL, 2013a).

No entanto, fatores relacionados ao ambiente, ao paciente e ao próprio profissional podem ter implicações na ocorrência de danos decorrentes de imperícia, negligência e imprudência. Neste contexto, pesquisa bibliográfica realizada para conhecimento de erros no exercício da enfermagem, que caracterizam imperícia, negligência e imprudência verificou que erros como contenção exagerada de paciente agitado, administração errônea de medicamentos, documentação incompleta das ocorrências durante a jornada de trabalho pode gerar danos remediáveis até óbito (CARBONI; REPPETTO; NOGUEIRA, 2018).

Neste sentido, imperícia refere-se a falta de perícia, incompetência e inexperiência; negligência é falta de atenção, menosprezo, desleixo, qualidade de negligente; imprudência refere-se a falta de cautela e prudência, que por sua vez é virtude que faz a pessoa conseguir o que deseja, evitando todos os perigos (DICIONÁRIO PRIBERAN DA LÍNGUA PORTUGUESA, 2019).

Conforme a Resolução COFEN nº 564/2017, que aprovou o novo Código de Ética dos Profissionais de Enfermagem (CEPE), no capítulo II, Artigo 45 diz que é dever dos profissionais de enfermagem: “Prestar assistência de Enfermagem livre de danos decorrentes de imperícia, negligência ou imprudência” (COFEN, 2017, p.157).

No Artigo 119, a mesma resolução referida acima descreve que a pena de cassação do direito ao exercício profissional é aplicável nos casos de infrações ao que está estabelecido no Artigo 45, entre outros (COFEN, 2017).

Estudo realizado pela *The Joint Comission* aponta as dez causas mais comuns de eventos: fatores humanos (68%); liderança (62%); comunicação (59%); avaliação das condutas (53%); gestão das informações (22%); infraestrutura (17%); continuidade do cuidado entre as unidades hospitalares (10%); plano do cuidado operatório (10%); e processos para utilização de medicação (9%). No entanto, destaca-se que este número representa uma pequena proporção dos acontecimentos que realmente acontecem na prática e que nem sempre são notificados (THE JOINT COMISSION, 2013).

Pesquisa conduzida a partir de estudos observacionais, cujo objetivo foi analisar o cuidado de saúde em ambientes hospitalares de diferentes países, estimou a ocorrência anual de aproximadamente 42,7 milhões de EA em todo o mundo, tendo como base 421 milhões de internações (JHA; LARIZGOITIA; AUDERA-LOPEZ et al., 2013).

No Brasil, conforme a tendência mundial em adotar ações de saúde para fortalecer à SP, desenvolvem-se inúmeras iniciativas como as investigações científicas relacionadas à ocorrência de EA (CARVALHO; CASSIANI, 2000). Como os pacientes estão expostos, continuamente, à riscos durante a prestação do cuidado em saúde, cabe às equipes envolvidas neste processo elaborar estratégias e proporcionar barreiras de proteção ao risco de dano aos pacientes (ROSCANI; FERRAZ; OLIVEIRA FILHO et al., 2015).

O cotidiano das instituições de saúde durante à prestação dos cuidados é complexo e, apesar da exigência dos elevados padrões de qualidade, verifica-se a ocorrência de incidentes e EA diariamente. Os autores sugerem que as instituições de saúde trabalhem em busca da Cultura de Segurança, considerada como ponto de partida para o planejamento de ações para a promoção da SP (LORENZINI; SANTI; BÁO, 2014).

Estudo realizado para descrever os EA relacionados com a assistência à saúde resultantes em óbito, conforme dados notificados no NOTIVISA, concluiu que os óbitos ocorreram principalmente em hospitais e que houve falhas nos registros dos atendimentos. A promoção da cultura de segurança deve motivar o aprendizado a partir das notificações, pois estas são importantes ferramentas para a vigilância sanitária e gestão dos serviços de saúde (MAIA; FREITAS; GALLO et al., 2018).

A comunicação e a informação são fundamentais para a gestão dos riscos, pois o conhecimento sobre a ocorrência dos EA, infecciosos ou não, possibilita a identificação das fontes do risco que fazem parte da cadeia de formação do dano ao paciente. (GOMES, 2012). O estabelecimento de cultura de segurança está intimamente ligado à gestão de risco, que está relacionada ao aprendizado com as falhas e à prevenção de novos incidentes (BRASIL, 2017a).

Importante estudo realizado em hospitais palestinos comprovou que as organizações que apresentaram cultura de segurança mais positiva tiveram menor taxa de EA (NAJJAR; NAFOURI; VANHAECHT et al., 2015). Neste contexto, todas as organizações que se preocupam com a SP devem analisar os dados sobre EA para conhecer, gradativamente, a realidade local, e propor estratégias direcionadas para a e assistência segura, bem como, desenvolver a cultura de segurança positiva. (BRASIL, 2013a; BRASIL, 2013b).

3.2 CULTURA DE SEGURANÇA

Os avanços tecnológicos, a diversidade de sistemas e processos organizacionais e a complexidade dos cuidados de saúde aumentam o potencial para a ocorrência de incidentes, erros ou falhas. A cultura de segurança reflete o comprometimento da organização na promoção contínua de ambiente seguro, bem como, influência comportamentos para o alcance de resultados positivos para profissionais e pacientes (SOUZA; MENDES, 2019).

A busca pelo fortalecimento da cultura de segurança emerge como um dos requisitos essenciais para melhoria do cuidado de saúde. E as organizações têm dado especial atenção ao tema, pois os cuidados de saúde, cada vez mais complexos, elevam o potencial da ocorrência de incidentes, erros ou falhas em hospitais (REIS; MARTINS; LAGUARDIA 2013a). Neste contexto, é importante o desenvolvimento de cultura avaliativa nas instituições de saúde pois, conhecer a cultura de segurança do cenário em que se pretende atuar é um aspecto imprescindível para efetivar melhorias (CARVALHO; GÖTTEMS; PIRES et al., 2015), uma vez que essa tem por objetivo promover ações e comportamentos adequados para um ambiente seguro (VICENT, 2010).

A promoção da cultura de segurança é um dos sustentáculos do movimento em prol da SP que permite reconhecer os fatores organizacionais que podem influenciar e causar danos ao paciente. Estratégias e estudos para avaliação da cultura de segurança são estimulados para auxiliar na melhoria da qualidade dos processos que envolvem os cuidados prestados aos pacientes (PETERLINI; SAES, 2014).

A formação de ambiente seguro para pacientes e profissionais necessita de plano estratégico, liderança e trabalho em equipe (TOBIAS; BEZERRA; BRANQUINHO et al., 2014). Neste contexto, a cultura de segurança pode ser compreendida por meio do comportamento individual e compartilhado dos membros da organização de saúde, de acordo com o comprometimento e responsabilidade na oferta de cuidado qualificado e seguro, independentemente de sua posição hierárquica (BRASIL, 2013c). Logo, a organização hospitalar para ser considerada segura precisa de esforço e cooperação coletiva, em todos os níveis hierárquicos (BRASIL, 2013a).

Para compreender o significado de cultura de segurança é necessário compreender os significados de cultura, que se refere aos costumes e rituais que as sociedades desenvolvem ao longo de sua história (CAMPOS; RÉDUA; ALVARELI, 2011). Cultura é considerado termo abstrato e complexo que envolve desde aspectos do aprendizado de determinada população até os epistemológicos e o desenvolvimento da intelectualidade do ser humano. Trata-se de fenômeno que ocorre no cotidiano e com características que passam despercebidas pelos sujeitos (MATOS; RODRIGUES; BORGES et al., 2016).

Nas abordagens sobre cultura existem duas grandes vertentes de metodologias, bem definidas e distintas. Numa delas a cultura define-se como algo que a instituição possui, assim como possui outros atributos, como estrutura e estratégia. Deste modo, a cultura torna-se variável independente e pode ser manipulada por intermédio de medidas que visam ao alcance dos objetivos organizacionais. São utilizados instrumentos que avaliam a correlação entre esses atributos e os resultados assistenciais (SCOTT; MANNION; DAVIES et al., 2003). Em contrapartida, na outra vertente está a abordagem antropológica, que define a cultura como um conjunto de valores, ideias, conceitos e regras de comportamento implícitas e explícitas, que são compartilhadas e permitem que um grupo social funcione e se perpetue (HUDELSON, 2004).

O termo cultura tem suas raízes na área de antropologia, no entanto, a aplicação dessas ideias deixou de ser focada na população indígena e passou a ser discutida no período pós-guerra, no contexto das organizações dos Estados Unidos, e tornou-se popular na década de 1980. Durante este período, livros na área de administração demonstravam que a cultura organizacional é crucial no gerenciamento dos serviços (DAVIES; NUTLEY; MANNION, 2000).

A seguir, partindo-se da fundamentação teórica sobre o termo cultura, descreve-se as terminologias frequentemente utilizadas, com suas respectivas especificidades e definições: cultura organizacional e clima / cultura de segurança.

3.2.1 Cultura Organizacional

A cultura organizacional refere-se às normas, valores, crenças e suposições profundamente incorporados pelos membros de uma organização. Estes evoluem ao

longo do tempo e são difíceis de mudar. O clima organizacional, em contraste, refere-se às percepções compartilhadas em um determinado ponto no tempo em relação às práticas organizacionais, como tomada de decisão, oportunidades de avanço e assim por diante. Estes são mais suscetíveis a mudanças (GERSHON; KARKASHIAN; GROSCH et al., 2000).

Neste sentido, a cultura organizacional é definida como um conjunto de pressupostos básicos considerados válidos e ensinados aos novos membros como a forma correta de perceber, pensar e sentir em relação aos problemas (SCHEIN, 1991). Envolve padrões de pressupostos, valores, símbolos e rituais que são inventados ou desenvolvidos por membros de uma empresa, para que lidem, de acordo com estes princípios, com os problemas de integração interna e adaptação externa (SCHEIN, 2010). Na cultura organizacional o líder desempenha o papel do modelo a ser seguido, influencia positivamente o desenvolvimento de comportamentos, valores e crenças que possibilitam seu fortalecimento (YAFANG, 2011).

Existem inúmeras definições na literatura sobre a cultura organizacional, porém, algumas características são frequentemente referenciadas: é uma abstração e não um fenômeno concreto, portanto, é de difícil definição e operacionalização; é relativamente estável ao longo do tempo; seu conceito é multidimensional e é partilhada por grupos de pessoas; pode conter vários aspectos que coexistem dentro de uma organização, por exemplo, a cultura de segurança; leva à práticas ostensivas e atende a uma proposta funcional (GULDENMUND, 2000).

Na concepção de outro autor, as principais características da cultura organizacional são: o grau em que os funcionários recebem estímulos para inovar; o grau em que se exige que os funcionários sejam precisos e atentos; o grau em que líderes e gestores valorizam os resultados; o grau em que as decisões são pautadas no bem estar dos funcionários; o grau em que as equipes são consideradas na tomada de decisão; o grau em que as pessoas são estimuladas pela organização a serem agressivas e competitivas e o grau em que a organização prioriza a estabilidade (ROBBINS; JUDGE; SOBRAL, 2010).

Existem seis dimensões que podem descrever a cultura de uma organização, conforme QUADRO 2:

QUADRO 2 – DIMENSÕES DA CULTURA ORGANIZACIONAL.

DIMENSÕES	DESCRIÇÕES
Primeira dimensão - orientada para o processo ou resultado	Em relação à valorização do processo quer dizer que a organização valoriza rotinas, técnicas burocráticas, e o modo como o trabalho é desenvolvido; em contrapartida, a valorização do resultado valoriza resultados, eficácia e solução de problemas.
Segunda dimensão - orientada para o trabalho ou para o empregado	A cultura orientada para o trabalho exerce pressão sobre os funcionários, a orientada para os funcionários prioriza o seu bem-estar.
Terceira dimensão - cultura profissional ou paroquial	Na cultura profissional as empresas estimulam as pessoas a separarem a vida pessoal da profissional e cada um é responsável pelo seu desenvolvimento dentro ou fora da empresa;<br>No sentido contrário, a cultura paroquial incentiva os indivíduos a não separarem o profissional da vida pessoal e a empresa se responsabiliza pelo seu desenvolvimento.
Quarta dimensão - sistemas abertos ou fechados	Relacionada ao tipo de comunicação que ocorre na organização. Nos sistemas abertos a sensação de fazer parte ocorre de modo mais rápido.
Quinta dimensão - controle apertado ou controle solto	Reflete o nível de formalidade e pontualidade da organização. Na cultura de controle apertado os horários são exigidos e respeitados; na cultura de controle solto os horários são flexíveis.
Sexta dimensão - pragmática ou normativa	Descreve a forma como a organização lida com os clientes. A cultura pragmática é flexível e a normativa prioriza aplicação de regras e leis.

FONTE: HOFSTEDE (2011).

A cultura orienta a maneira de fazer as coisas por pressuposições não escritas e nem sequer faladas. Apresentam-se em três diferentes dimensões: artefatos, valores compartilhados e pressupostos básicos. Os artefatos constituem as coisas concretas que cada pessoa vê, ouve e sente quando se depara com uma organização e abrangem os produtos, serviços ou departamentos, padrões de comportamento dos membros da organização e se expressam no modo como as pessoas se vestem, comportam-se e sobre o que conversam (SCHEIN, 1991).

Porém os valores compartilhados são valores importantes para as pessoas e definem as razões pelas quais elas fazem o que fazem. As pressuposições básicas constituem a dimensão mais íntima da cultura organizacional, que são as crenças inconscientes, percepções, sentimentos e pressupostos nos quais as pessoas creem (SCHEIN, 1991).

Para ocorrer uma mudança organizacional deve haver uma mudança cultural para depois transformar processos e recursos. Quando as pessoas começam a com-

partilhar ideias a respeito de assuntos que consideram importantes, cria-se uma cultura de aprendizado, transformando o conhecimento no principal ativo da organização. A base do processo de mudança organizacional é o capital intelectual, o qual está relacionado às pessoas. Desta forma, a construção da transformação sustentável passa pelo envolvimento das pessoas que fazem parte da organização (RABELO; FERENHOF; RADOS et al., 2012).

O comportamento coletivo reflete nos valores operacionais da organização que podem ter efeito sobre a segurança (NATIONAL QUALITY FORUM, 2010). As organizações de cuidado em saúde não medem esforços para melhorar a assistência prestada e, por este motivo, se reconhece a importância da cultura de SP, que necessita do entendimento sobre os valores, crenças e normas das organizações; e de quais atitudes e comportamentos são suportados, recompensados e esperados em relação à prestação de cuidados seguros aos pacientes (PAESE; SASSO, 2013).

3.2.2 Cultura de segurança do paciente

Historicamente, os processos de segurança sofreram grande avanço e tiveram início com a instalação e funcionamento das usinas nucleares, depois dos acidentes de *Three Mile Island*, nos EUA e de Chernobyl, na antiga União Soviética (FERRAZ, 2009). O termo cultura de segurança tornou-se popular após este desastre nuclear de *Chernobyl*, na década de 1980, quando foi sugerido que as organizações poderiam reduzir acidentes e incidente com o desenvolvimento de cultura de segurança positiva (THE HEALTH FOUNDATION, 2011). Desde então, a ocorrência de erros nessa área levou à identificação de falhas organizacionais, análise dos erros e produção de relatórios que possibilitaram conceituar a cultura de segurança (FLIN; BURNS; MEARNS et al., 2006).

A agência internacional de energia atômica definiu cultura de segurança como o conjunto de características e atitudes que ocorrem nas organizações e o significado que os indivíduos estabelecem nas questões de proteção e segurança (INTERNATIONAL ATOMIC ENERGY AGENCY, 2015). Ao analisar o impacto econômico, social e moral de tais catástrofes, as organizações e sistemas que executavam operações, consideradas de alto risco, investiram em meios de desenvolver estratégias de segurança. Estas organizações conseguiram reduzir a

incidência de erros, apesar de operarem em sistemas complexos (HALLIGAN; ZECEVIC, 2011). Esses erros tornaram-se relevantes para os cuidados de saúde na linha do que ocorreu com a aviação civil (SOUZA; MENDES, 2014).

Neste sentido, cultura de segurança é definida como conjunto de crenças partilhadas que dão suporte a práticas seguras entre os profissionais que nelas trabalham (WHO, 2008b). Organizações onde a cultura é positiva caracterizam-se por comunicação pautada em confiança mútua, por percepções compartilhadas sobre a importância de prestar cuidados seguros e pela confiança na eficácia de medidas preventivas (NIEVA; SORRA, 2003; SORRA; NIEVA, 2004).

É dever de toda instituição de saúde partilhar cultura de segurança positiva como ação essencial para reduzir a probabilidade da ocorrência de danos aos pacientes, principalmente EA evitáveis. Isso emerge por meio do aprendizado proativo, a partir dos erros ocorridos e redesenho dos processos (HANDLER; CASTLE; STUDENTSKI et al., 2006).

A falta de trabalho em equipe eficaz, e falhas na comunicação, são consideradas as principais causas de erros e influenciam, fortemente a ocorrência de incidentes de segurança do paciente (GABRANI; KNIBB; PETRELA et al., 2016).

No guia denominado *Leading A Culture of Safety: a Blueprint for Succes*, a cultura de segurança das organizações de saúde deve envolver seis domínios: Visão; Confiança, Respeito e Inclusão; Engajamento do Conselho Administrativo; Desenvolvimento de Liderança; Cultura Justa; Expectativas de Comportamento (ACHE; IHI; NPSF; LUCIAN LEAPE INSTITUTE, 2017, tradução nossa).Tais domínios são melhor explorados conforme apresentado no QUADRO 3.

QUADRO 3 – DOMÍNIOS DA CULTURA DE SEGURANÇA NAS ORGANIZAÇÕES.

**DOMÍNIO**	**DESCRIÇÃO**
1.Estabelecer uma visão convincente para a segurança	A visão de uma organização reflete prioridades que, quando alinhadas a sua missão, estabelecem base sólida para o trabalho da organização. Ao incorporar uma visão para a segurança do paciente e da força de trabalho dentro da organização, os líderes da área de saúde demonstram que a segurança é um valor central.
2.Construção de confiança, respeito e inclusão	Estabelecer confiança, demonstrar respeito e promover a inclusão. Demonstrar esses princípios em toda a organização, e com os pacientes e famílias, é essencial para a capacidade de um líder criar e de sustentar uma cultura de segurança. A fim de alcançar o dano zero, os líderes devem garantir que suas ações sejam consistentes em todos os momentos e em todos os níveis da organização. Confiança, respeito e inclusão são padrões não negociáveis que devem abranger a sala de diretoria, os departamentos clínicos e toda a força de trabalho.
3.Seleção, desenvolvimento e envolvimento da diretoria	Os conselhos executivos-administrativos desempenham papel vital na criação e manutenção de culturas de segurança. Os conselhos administrativos são responsáveis por garantir a educação dos membros do conselho sobre a ciência da segurança básica, incluindo a importância e os processos para manter os pacientes e a força de trabalho seguros. As diretorias devem garantir que as métricas que avaliam, significativamente, a segurança organizacional e cultura de segurança sejam implementadas sistematicamente, revisadas, analisadas, e os resultados atestados.
4.Priorizar a segurança na seleção e desenvolvimento de líderes.	É responsabilidade do conselho executivo-administrativo, em colaboração com a diretoria, incluir a responsabilidade pela segurança como parte da estratégia de desenvolvimento de liderança da organização. Além disso, identificar médicos, enfermeiros e outros líderes clínicos como defensores da segurança é fundamental para diminuir a distância entre o desenvolvimento de liderança administrativa e clínica.
5.Liderar e recompensar uma cultura justa	Os líderes devem possuir compreensão completa dos princípios e comportamentos da cultura justa e estar comprometidos em ensinar. Numa estrutura de cultura justa, o foco é abordar os sistemas e questões que contribuem para erros e danos. Embora a força de trabalho seja responsabilizada por negligenciar ativamente protocolos e procedimentos, o relato de erros, lapsos, quase-acidentes e eventos adversos é incentivado. A força de trabalho é suportada quando os sistemas quebram e ocorrem erros. Na verdadeira cultura justa, todos os membros da força de trabalho, tanto clínicos quanto não-clínicos, estão empoderados e sem medo de expressar preocupações sobre as ameaças à segurança do paciente.
6.Estabelecer expectativas de comportamento organizacional	Os líderes seniores são responsáveis por estabelecer a atenção plena em segurança para todos os integrantes da força de trabalho e, talvez até mais importante, modelar esses comportamentos e ações. Esses comportamentos incluem, mas não se limitam a transparência, trabalho em equipe eficaz, comunicação ativa, civilidade e *feedback* direto e oportuno. Esses compromissos culturais devem ser entendidos universalmente e aplicar-se igualmente para toda a força de trabalho, independentemente da classificação, função ou departamento.

FONTE: *American College of Healthcare Executives (ACHE); National Patient Safety Foundation (NPSF); Institute for Healthcare Improvement (IHI); Lucian Leape Institute*, (2017).

Estudo transversal conduzido em unidades de urgência e emergência da Holanda identificou dimensões positivas da cultura associada com a segurança do paciente (VERBEEK-VAN NOORD; WAGNER; VAN DYCK et al., 2013). Outro estudo transversal realizado em 68 hospitais libaneses, com 6.807 profissionais de saúde, encontrou indícios significativos da relação da cultura positiva e a SP atendidos (EL-JARDALI; DIMASSI; JAMAL et al., 2011).

A cultura de segurança envolve o comprometimento da organização e de seus gestores para identificar a necessidade do desenvolvimento de ações seguras como eixo norteador, bem como, conhecer as dificuldades e desafios que o prestador de cuidado direto vivencia diariamente, de modo a criar canal de comunicação efetivo. Organizações confiáveis mantêm o compromisso com a segurança em todos os níveis, do operacional às lideranças e até a alta direção (PAESE; SASSO, 2013).

Pesquisa descritiva conduzida em sete hospitais da China para avaliar a relação entre cultura de segurança do paciente e ocorrência de EA confirmou a hipótese de que uma cultura positiva está relacionada à minimização na ocorrência desses eventos (WANG; LIU; YOU et al., 2014). Outra investigação retrospectiva realizada em dois hospitais palestinos, com o objetivo de analisar se os aspectos da cultura de segurança estavam associados com as taxas de EA, nos departamentos de cirurgia, obstetrícia, medicina interna e ortopedia, verificou que cultura de segurança do paciente mais positiva está associada com a redução de EA em hospitais (NAJJAR; NAFOURI; VANHAECHT et al., 2015).

No Brasil, estudo identificou publicações científicas em português, espanhol e inglês, no período de 2010 até 2014, sobre EA na assistência de enfermagem em pacientes hospitalizados. Os pesquisadores destacaram a importância da notificação, da compreensão e das causas das ocorrências e, apontaram medidas preventivas, como o reconhecimento das ocorrências e a busca por uma cultura de segurança organizacional (DUARTE; STIPP; SILVA et al., 2015).

Conhecer a cultura de segurança por meio dos profissionais que prestam cuidado em saúde tem implicação direta nas atividades de promoção da SP (CAUDURO; SARQUIS; CRUZ, 2015). Estudo realizado para analisar a contribuição da enfermagem para a SP no Brasil, por meio do método da revisão integrativa da literatura, demonstrou que as publicações evidenciaram a importância da identificação

do erro e da utilização de ferramentas para melhoria da cultura de segurança nas instituições brasileiras (SILVA; SILVA; SANTOS et al., 2016*).*

Em consonância, outro estudo revelou que as instituições devem repensar seus processos de notificação de EA por constituir ferramenta fundamental para promover a SP, pois fornece dados importantes para análises e avaliações, implementação de barreiras, revisões de processos assistenciais e gerenciais para melhoria da qualidade (COSTA; RAMOS; GABRIEL et al., 2018).

Em hospitais, a criação de sistema de notificação de incidentes é encorajada para reunir dados que contribuam para melhoria significativa na segurança e na qualidade do trabalho; e como um ponto de partida para iniciar o planejamento de ações que busquem mudanças na intenção de reduzir a incidência de eventos adversos (SOUZA; MENDES, 2019). É necessário repensar o processo de trabalho nas instituições hospitalares para superar as falhas e problemas, reconhecer que toda a atividade de assistência à saúde possui pontos frágeis que podem comprometer a SP. Deste modo, a chave para reduzir o risco é criar um ambiente sem a cultura da culpa, bem como o desenvolvimento da cultura de vigilância e cooperação (CAMERINI; SILVA, 2011).

Em virtude das recentes políticas públicas no Brasil, percebe-se o início da construção de novas percepções culturais sobre a SP. O sistema de saúde deve oportunizar estratégias, como a comunicação dos erros e da cultura não punitiva, pois deste modo é possível a identificação dos fatores causais e implementação de barreiras que podem diminuir as situações de risco (TOMAZONI; ROCHA; SOUZA et al., 2014).

Diversos autores também defendem o desenvolvimento da cultura de segurança com foco na não punição, e sugerem a detecção do incidente, promoção da implementação de recomendações voltadas para as melhores práticas, com a finalidade de contribuir com a SP e consequente redução do número de incidentes. (TEIXEIRA; CASSIANI, 2014). As características de cultura de segurança sólida incluem ações que estimulam o compromisso para discutir e aprender com os erros, reconhecimento de sua inevitabilidade, identificação proativa das ameaças latentes e incorporação de sistema não punitivo frente aos relatos e análises dos EA (PAESE; SASSO, 2013).

Desta maneira, espera-se que a cultura de segurança, nas organizações de saúde, englobe os seguintes componentes: cultura justa não punitiva frente à ocorrência de incidentes, mas que prioriza a identificação de suas causas; cultura de notificação de incidentes que inclui etapas como coleta/recolha, análise e divulgação das informações referentes à notificação de incidentes e quase incidentes, de forma a encorajar as pessoas a falar sobre os erros e notificá-los e, por fim, cultura de aprendizagem, pois a organização possui memória de eventos passados que pode direcionar o aprendizado (WHO, 2008b).

O cuidado seguro depende da construção de uma cultura de segurança definida como cultura na qual todos os trabalhadores, incluindo profissionais envolvidos no cuidado e gestores, assumem responsabilidade pela sua própria segurança, pela segurança de seus colegas, pacientes e familiares; cultura que prioriza a segurança acima de metas financeiras e operacionais e cultura que encoraja e recompensa a identificação, a notificação e a resolução dos problemas relacionados à segurança (BRASIL, 2013c). Outra definição de cultura de segurança nos serviços de assistência à saúde compreende o conjunto global das percepções de clima apresentado pelos profissionais em relação ao comprometimento da organização com as questões de segurança (THE JOINT COMMISSION, 2013).

O clima de segurança é definido como a medida das atitudes e percepções individuais das características da cultura de segurança entre os trabalhadores da organização, que pode variar dentro da instituição. Os autores relatam que a avaliação da cultura de segurança em uma instituição pode ser obtida por meio da percepção do clima de segurança relatado por seus profissionais (SINGER; VOGUS, 2013).

Deste modo, os termos clima e cultura de segurança são utilizados na literatura como sinônimos. No entanto, quando se utilizam questionários específicos para mensurar as percepções de um grupo de profissionais sobre diversos aspectos no seu local de trabalho, o termo mais apropriado é clima. A cultura de segurança pode ser conhecida a partir do clima de segurança que se constitui na avaliação temporal. No entanto, o termo percepções faz parte de ambas as definições (SEXTON; HELMREICH; NEILANDS et al., 2006).

Neste contexto, cultura e clima de segurança serão tratados nesta pesquisa como sinônimos, não interferindo na análise dos dados, permanecendo em

consonância com a literatura utilizada na área de SP. Infere-se que em processos ava-

liativos que utilizam instrumentos validados permitem o desenvolvimento de cultura de segurança do paciente positiva nas instituições de saúde, especificamente no Centro Cirúrgico, e pode contribuir para melhoria na qualidade da assistência e diminuição dos EA cirúrgicos e mortalidade.

A avaliação da cultura de segurança permite a mensuração das condições organizacionais que podem ocasionar possíveis danos aos pacientes nos serviços de saúde. (BRASIL, 2016). Neste contexto, é obrigação moral e ética de todas as pessoas envolvidas garantir a qualidade no atendimento, segurança e prevenção de danos evitáveis aos pacientes e seus familiares, para criar culturas sustentáveis de segurança. A taxa de erros e danos originários da assistência à saúde alerta para a necessidade de mudanças abrangentes em todo o sistema (ACHE; IHI; NPSF; LUCIAN LEAPE INSTITUTE, 2017).

Os resultados do estudo que avaliou as relações entre a pesquisa hospitalar de SP da *Agency for Healthcare Research and Quality's (AHRQ)* e as taxas de complicações intra-hospitalares e EA, que foram mensurados pelos indicadores de SP desta mesma Agência, indicaram que os hospitais com escores mais positivos de cultura de segurança do paciente apresentaram menores taxas de complicações intra-hospitalares ou EA. No entanto, os autores sugerem que mais pesquisas sejam realizadas em número maior de hospitais para verificar a relação causal entre cultura de segurança e os resultados clínicos verificados nos pacientes (MARDON; KHANNA; SORRA et al., 2010).

Portanto, a cultura de segurança em organizações de saúde desponta como um indicador estrutural facilitador de iniciativas para redução dos riscos de incidentes e de eventos adversos (AGENCY FOR HEALTHCARE RESEARCH AND QUALITY'S (AHRQ), 2013). Mensurar e compreender a cultura de segurança de uma organização é importante ferramenta de avaliação da qualidade dos cuidados prestados (NORDÉN-HÄGG; SEXTON; KÄLVEMARK-SPORRONG et al., 2010). Essas ações permitem a identificação e gerenciamento da SP e a prevenção de EA cirúrgicos (LOURENÇÃO; TRONCHIN, 2016).

3.3 EVENTOS ADVERSOS CIRÚRGICOS

Em virtude dos erros associados aos procedimentos cirúrgicos, a OMS lançou em 2008 o Programa Cirurgias Seguras Salvam vidas (PCSSV). Para que os objetivos

desta campanha sejam alcançados é necessário compreender que a segurança não deve estar restrita apenas ao ato cirúrgico, mas também à avaliação pré-operatória e cuidado pós-operatório adequado, além dos aspectos organizacionais fundamentais ao procedimento (OMS, 2009).

O respectivo programa apresenta 10 objetivos para serem alcançados para segurança do paciente cirúrgico:

1. A equipe operará o paciente certo e o local cirúrgico certo.
2. A equipe usará métodos conhecidos para impedir danos na administração de anestésicos, enquanto protege o paciente da dor.
3. A equipe reconhecerá e estará efetivamente preparada para perda de via aérea ou de função respiratória que ameacem a vida.
4. A equipe reconhecerá e estará efetivamente preparada para o risco de grandes perdas sanguíneas.
5. A equipe evitará a indução de reação adversa a drogas ou reação alérgica sabidamente de risco ao paciente.
6. A equipe usará de maneira sistemática, métodos conhecidos para minimizar o risco de infecção no sítio cirúrgico.
7. A equipe impedirá a retenção inadvertida de instrumentais ou compressas nas feridas cirúrgicas.
8. A equipe manterá seguros e identificará precisamente todos os espécimes cirúrgicos.
9. A equipe se comunicará efetivamente e trocará informações críticas para a condução segura da operação.
10. Os hospitais e os sistemas de saúde pública estabelecerão vigilância de rotina sobre a capacidade, volume e resultados cirúrgicos (OMS), 2009).

O PCSSV sugere a utilização de *checklist* cirúrgico com 19 perguntas distribuídas em três etapas: “antes da indução anestésica”, “antes da incisão cirúrgica” e “antes do paciente deixar a sala operatória. Esta lista foi aplicada em oito instituições pilotos no mundo e foram investigados 7.688 pacientes, sendo que 3.733 antes da utilização do *checklist* e 3.955 após o preenchimento do *checklist*. Os resultados evi

denciaram redução de 36% das complicações cirúrgicas, 47% na taxa de mortalidade, 50% nas taxas de infecção e 25% na necessidade de nova intervenção

cirúrgica. Assim sendo, estima-se que foram evitados danos a 250 pacientes e 27 mortes em um universo de 4.000 pacientes (OMS, 2009).

As falhas na segurança dos processos cirúrgicos causam EA sérios, como cirurgia realizada em local inadequado, em paciente errado, retenção de objeto estranho no organismo do paciente, e morte no intraoperatório ou pós-operatório imediato. Ocorrências essas que justificam a importância da implantação de protocolos para sua prevenção. Verifica-se que nem todos os pacientes com EA cirúrgicos evoluem para o óbito, contudo, frequentemente permanecem com sequelas/incapacidades e têm o tempo de internação prolongado entre três e 16% (OMS, 2009).

Na assistência perioperatória são verificados outros EA cirúrgicos, como erros relacionados à medicação e transfusões, quedas, queimaduras por uso inadequado de equipamentos, complicações associadas à anestesia, os quais demandam atenção especial da equipe envolvida no cuidado em saúde (WACHTER, 2013). Os EA cirúrgicos podem ser causados pela inexperiência do cirurgião, carga excessiva de trabalho e fadiga dos profissionais, deficiência na supervisão de estagiários e falhas na comunicação entre os profissionais (GAWANDE, 2011).

Em relação ao paciente cirúrgico, a AHRQ, dos Estados Unidos da América, definiu os seguintes indicadores a serem monitorados nos serviços de saúde: complicações na anestesia; mortalidade por doença pré-existente ou situações adversas de baixa mortalidade; úlcera de decúbito; falha de resgate; corpo estranho deixado no corpo durante a cirurgia; pneumotórax iatrogênico; infecções relacionadas à assistência à saúde; fratura de quadril pós-operatória; hemorragia ou hematoma; alterações fisiológicas ou metabólicas; disfunção respiratória; trombose venosa profunda ou embolia pulmonar; sepse; deiscência de sutura pós cirurgia abdominal e pélvica; punção acidental e laceração; reação transfusional; trauma de nascimento com dano ao neonato; trauma obstétrico em parto vaginal com instrumento, em parto vaginal sem instrumento e por cesariana (AHRQ, 2011).

Estudo sueco com análise de 960 prontuários identificou que cerca de 20% dos pacientes foram acometidos por EA; destes 26% eram cirúrgicos e sofreram pelo menos um evento (RUTBERG; RISBERG; SJODAH et al., 2014). Pesquisa conduzida

em oito países da África e no Oriente Médio identificou incidência de 18,4% de EA relacionados à assistência cirúrgica (WILSON; MICHEL; OLSEN et al., 2012).

Na Tunísia essas ocorrências representaram 55% do total dos casos entre pacientes cirúrgicos e submetidos a procedimentos invasivos (LETAIEF; EL-MHAMDI; EL-ASADY et al., 2010). Devido à complexidade e risco decorrente de cirurgia, estudos têm evidenciando a relação destes fatores com a ocorrência de EA. Revisão sistemática realizada com 14 estudos incorporando 16.424 pacientes cirúrgicos identificou taxa de EA cirúrgico de 14,4% (ANDERSON; DAVIS; HANNA et al., 2013).

Em relação às lesões de pele ao término dos procedimentos anestésicos-cirúrgicos, estudo demonstrou a ocorrência 74% dos pacientes; os fatores de risco identificados foram a falta do uso de protetores corporais, tempo cirúrgico prolongado, obesidade ou sobrepeso (BARBOSA; OLIVA; SOUZA NETO, 2011). Outro estudo realizado sobre a ocorrência de lesões de pele no período intraoperatório demonstrou que dos 154 pacientes avaliados, sete (4,5%) apresentaram 11 lesões de pele, predominantemente, no glúteo e no tórax; os principais fatores associados foram as variáveis tempo do procedimento cirúrgico, tipo de anestesia e comorbidades (BEZERRA; GALVÃO; VIEIRA et al., 2019).

Neste contexto, o grande desafio é identificar situações que possibilitam a ocorrência de possíveis erros durante os procedimentos cirúrgicos, como falhas nos equipamentos cirúrgicos e de anestesia, falta de pessoal capacitado, equipe cirúrgica que trabalha sob pressão, utilização de novas tecnologias sem o conhecimento necessário, entre outras. Este cenário incentiva a busca de novos conhecimentos em virtude dos riscos, erros e EA aos pacientes cirúrgicos (CONRARDY; BRENEK; MYERS, 2010).

Revisão sistemática que analisou a ocorrência em pacientes cirúrgicos norte-americanos de *never events,* ou seja, eventos que nunca deveriam ocorrer, estimou a ocorrência de cirurgia em local errado e itens cirúrgicos retidos de 1 EA /100.000 e 1 EA/10.000 procedimentos, respectivamente (HEMPEL; MAGGARD-GIBBONS; NGUYEN et al., 2015).

No Brasil, em 2004, investigação sobre ocorrência de erros médicos, conforme os processos éticos instaurados no Conselho Regional de Medicina no Eestado de São Paulo, verificou que as razões mais frequentes dos processos se referiam às especialidades cirúrgicas, aliadas às condutas ético profissionais, como negligência, imperícia e imprudência, principalmente nas especialidades de ortopedia, traumatologia e cirurgia geral (DUARTE; STIPP; SILVA et al., 2015).

Estudo conduzido em três hospitais de ensino da região sudeste do Brasil, com dados de 1.103 internações ocorridas no decorrer do ano de 2003, demonstrou incidência de EA de 7,6%. A maioria dos casos investigados foram atribuídos a procedimentos cirúrgicos (35,2%). Evidenciou-se que os cuidados de saúde causaram danos na proporção de 1/5 dos pacientes que apresentaram EA cirúrgico, pois estes evoluíram com incapacidade permanente ou morreram (MENDES; MARTINS; ROZENFELD et al., 2009).

Outro estudo realizado em hospital de ensino no sul do Brasil para verificar a ocorrência de EA notificados em pacientes internados em unidade cirúrgica observou incidência de 20 (7,9%) EA, sendo nove (3,6%) notificações de perda do acesso venoso, quatro (1,6%) notificações de infecção do acesso venoso e sete notificações de infecção do sítio cirúrgico (2,8%). Resultados demonstraram a necessidade de ações que estimulem a notificação de EA e a promoção da SP como educação permanente, quantitativo de profissionais adequado, condições adequadas de dispositivos e materiais, rotinas bem estabelecidas e de fácil acesso (SELL; AMANTE; MARTINS et.al., 2016).

Neste contexto, inúmeros fatores organizacionais e humanos são responsáveis para ocorrência de EA cirúrgicos (SANTIAGO; TURRINI, 2015). Tais eventos causam danos no paciente e podem originar inúmeras consequências, inclusive danos permanentes. Durante o ato cirúrgico, ocorrem em média 60 interrupções ou distrações pelo movimento de pessoas na sala de cirurgia. Este é um dos fatores humanos que influencia na ocorrência de erros, ou seja, o fator concentração (PEREIRA; PEREIRA; CORREIA et al., 2011). Por este motivo, o comportamento humano no cuidado em saúde torna-se um desafio constante para SP, devido ao valor atribuído pelo profissional ao paciente e às ações básicas de segurança (CRUZ, 2016).

Em pesquisa realizada em clínica de internação cirúrgica na região centro-oeste do Brasil verificou-se a ocorrência de 218 EA e sete resultaram em óbito. Os EA mais recorrentes foram dor aguda no pós-operatório, retirada de dispositivos tubulares não programada, falhas de procedimentos relacionados à falta de habilidade técnica do profissional que resultou em nova intervenção cirúrgica, prolongamento no tempo

de internação, complicações nos locais de punção, lesões por mau posicionamento da traqueostomia e óbito. Foram apontados problemas administrativos que

interferiram na qualidade do atendimento como suspensões cirúrgicas e internações indevidas, as quais resultaram no prolongamento da internação e novo agendamento do procedimento cirúrgico (PARANAGUA; BEZERRA; SILVA et al., 2013).

Estudo realizado para avaliar a prevalência dos incidentes ocorridos em centro cirúrgico de hospital de ensino da região centro-oeste do Brasil estimou a prevalência de 8,7 % de pacientes que foram expostos a algum tipo de incidente como: cirurgia suspensa (34,6%); perfuração de luva (27,0%); falhas técnicas envolvendo o paciente como dor durante cirurgia, reação do paciente, lesão uretral, perfuração de reto, bloqueio anestésico mal sucedido (19,2%); gerenciamento do serviço (19,2%). Esses evidenciaram falhas no processo de trabalho, como falha de organização e falta de material, exame suspenso com paciente em sala, perda de amostra de material (BEZERRA; BEZERRA; PARANAGUÁ et al., 2015).

Pesquisa desenvolvida em centro cirúrgico ambulatorial privado, na região sul do país, que analisou 250 notificações, verificou que as ocorrências mais frequentes foram relacionadas aos erros de processos pelo descumprimento de rotinas e padrões institucionais. As principais causas de EA, que envolveram diretamente o paciente, foram: queimaduras ocasionadas pelo uso indevido de bisturi elétrico, queda, erros de medicação (relacionados à prescrição e administração) e a ocorrência de úlcera por pressão após procedimento cirúrgico de longa duração, o que evidenciou mau posicionamento do paciente (SILVA; JUNIOR; OLIVEIRA et al., 2015).

Ao avaliar os estudos sobre a SP no período perioperatório, verifica-se que todo o processo fica comprometido por erros de montagem de sala cirúrgica; não implementação de todas as etapas do processo de enfermagem; ausência de diálogo entre o paciente e equipe a respeito dos medos, ansiedade e dúvidas; falta de entendi
mento entre a equipe multiprofissional; movimentação e ruídos excessivos na sala cirúrgica e a não realização do *checklist* recomendado pela OMS (HENRIQUES; COSTA; LACERDA, 2016).

Em relação à ocorrência de possíveis erros ou EA no período perioperatório, infere-se que o enfermeiro e a equipe técnica de enfermagem influenciam nos proces

sos relacionados a SP cirúrgicos. Isto se deve porque a enfermagem é a maior força de trabalho em saúde no Brasil, com 2.216.068 profissionais cadastrados nos Conselhos Regionais de Enfermagem (COFEN, 2019). O que remete a necessidade da relação direta da categoria com as estratégias de SP e prevenção de erros (DUARTE; STIPP; SILVA, 2015).

A assistência cirúrgica requer o exercício de atividades gerenciais do enfermeiro, como a agilidade de tomada de decisões, conhecimento técnico científico, organização e planejamento das atividades, habilidade no trabalho em equipe, flexibilidade e comunicação eficiente com os demais profissionais (MARTINS; DALL'AGNOL, 2016).

O preenchimento do *checklist* de segurança cirúrgico deve ser feito na primeira etapa com o envolvimento do paciente acordado e consciente, exceto em casos de emergência. Após, deve-se marcar o local ou os locais onde vai ser realizado o procedimento, com marcador de tinta permanente, pelo cirurgião ou outro membro da equipe, mas na presença dele. Realizar o *time out*, ou seja, pausa breve antes da intervenção cirúrgica para revisão da história clínica do paciente, estratégia cirúrgica, equipamentos e recursos necessários (SOUZA; MENDES, 2019).

Tais ações facilitam o compartilhamento de informações, desenvolvem a cooperação entre os membros da equipe (FLIN; PATEY; GLAVIN et al., 2010). Deste modo, desenvolve-se a cultura de segurança para atuação coordenada em prol de um objetivo comum que é a SP e a efetividade do ato cirúrgico para prevenção de EA (KRAUSE; HIDLEY, 2009).

3.4 METODOLOGIAS E INSTRUMENTOS PARA AVALIAR A CULTURA DE SEGURANÇA E EVENTOS ADVERSOS CIRÚRGICOS

Nas organizações de saúde, a maior parte dos estudos que avalia cultura de segurança utiliza questionários como instrumento de coleta de dados, os quais possuem uma combinação de dimensões e são considerados eficiente estratégia (FLIN; BURNS; MEARNS et al., 2006). A avaliação se dá utilizando-se escalas e questionários autoaplicáveis (RIGOBELLO; CARVALHO; CASSIANI et al., 2012).

Os questionários possibilitam a mensuração de múltiplas dimensões da cultura de segurança do paciente para verificar a opinião dos profissionais sobre

pontos como crenças, normas da organização, notificação de eventos adversos, comunicação, liderança e gestão (SOUZA; MENDES, 2019).

Em relação à ocorrência de EA, a mensuração pode ocorrer por meio da notificação voluntária do incidente, por uma série de indicadores de segurança e por revisão de prontuários isoladamente ou após o uso de uma ferramenta de rastreamento (WACHTER, 2013).

A OMS criou em 2002, durante a 55ª Assembléia Mundial da Saúde, um grupo de trabalho com o objetivo de estudar metodologias para avaliar os riscos para a SP nos serviços de saúde. O estudo focou a atenção em metodologias que avaliaram a
natureza e a magnitude do agravo causado pelos EA, e classificou os estudos em seis grupos (WHO, 2003):

1) Estudos de revisão retrospectiva de prontuários, inquéritos confidenciais, estudos de taxas de erros e dados de queixas e de ações litigiosas;
2) Estudos com dados colhidos da atividade de cuidados dos profissionais com revisão retrospectiva de prontuários e inquéritos transversais;
3) Estudos observacionais;
4) Análise de dados provenientes de encontros sobre morbimortalidade, autópsias, auditoria médica e seguros de saúde;
5) Sistemas de relatórios (externos ou internos) à organização e de especialistas;
6) Informação obtida em entrevista, por meio de informantes-chave ou grupos focais.
7) Questionário autoaplicável a profissionais.

Na prática, observa-se a ocorrência de erros que não são notificados nos relatórios de incidentes, por isso, a utilização de ferramentas de rastreamento surgiu como método de escolha para mensurar a incidência de EA associados ao cuidado em saúde (WACHTER, 2013). Dentre as metodologias, instrumentos e ferramentas utilizados em pesquisas na SP destacamos os que foram utilizados no desenvolvimento desta pesquisa:

3.4.1 *Hospital Survey on Patient Safety Culture - HSOPSC*

O instrumento *Hospital Survey on Patient Safety Culture (HSOPSC)* foi desenvolvido pela *Agency for Healthcare Research and Quality* (AHRQ) dos Estados Unidos em 2004, com o objetivo de avaliar múltiplas dimensões da cultura de segurança do paciente. (AHRQ, 2017; REIS; LAGUARDI; MARTINS, 2012; REIS; LAGUARDIA; VASCONCELOS, et al., 2016).

No Brasil, a versão do HSPOSC foi traduzida, adaptada transculturalmente para língua portuguesa e validada em dois hospitais do Rio de Janeiro, com nível de confiabilidade *Alfa de Crombach* de 0,91. Importante salientar que este instrumento já estava autorizado pela AHRQ para livre uso no país (REIS; LAGUARDIA; MARTINS, 2012; REIS, 2013; REIS; LAGUARDIA; VASCONCELOS, et al., 2016).

Trata-se de questionário autoaplicável constituído por nove seções, dispostas da letra A a I, somando-se 42 questões que mensuram a percepção dos profissionais de saúde sobre a cultura de segurança, as quais são agrupadas em 12 dimensões avaliadas a partir de uma escala de *Likert* de cinco pontos, com categorias de respostas em grau de concordância (REIS; LAGUARDIA; MARTINS, 2012; REIS; LAGUARDIA; VASCONCELOS, et al., 2016; SORRA; GRAY; STREAGLE et al., 2016).

As 12 dimensões do HSPOSC estão divididas em três níveis de avaliação: as três primeiras dimensões questionam sobre o ambiente hospitalar geral, as sete seguintes sobre os indicadores relacionados ao ambiente específico da unidade de trabalho e as duas últimas são variáveis de resultado (SORRA; NIEVA, 2004; REIS; LAGUARDIA; VASCONCELOS, et al., 2016; AHRQ, 2017). As respectivas descrições sobre cada dimensão encontram-se no QUADRO 4.

QUADRO 4 – DIMENSÕES DA CULTURA DE SEGURANÇA CONFORME *HSOPSC* VERSÃO BRASILEIRA.

**NÍVEL**	**DIMENSÃO**	**DESCRIÇÃO**
HOSPITALAR	Apoio da gestão para segurança do paciente	Avaliação da gestão hospitalar proporcionar um clima de trabalho que promova a segurança do paciente e mostrar que a segurança do paciente é um fator relevante na instituição
	Trabalho em equipe entre unidades	Investigação se as unidades de trabalho cooperam e coordenam-se entre si para melhorar o atendimento ao paciente
	Passagem de plantão/turno e transferências	Transmissão de informações importantes relacionadas ao tratamento do paciente entre as unidades hospitalares e durante as mudanças de turno
UNIDADE	Expectativas sobre seu supervisor/chefe e ações promotoras da segurança	Investigação se gestores consideram e valorizam as sugestões dos funcionários para melhorar a segurança do paciente
	Aprendizado organizacional, melhoria contínua	Mensuração da percepção de que os erros levaram a mudanças positivas
	Trabalho em equipe dentro das unidades	Identificação se há trabalho em equipe nas unidades, com apoio e respeito mútuo
	Abertura da comunicação	Disposição dos funcionários em comunicar algo que pode afetar negativamente um paciente e de não hesitar em questionar aqueles com maior autoridade
	Retorno da informação e comunicação sobre o erro	Informação aos funcionários sobre os erros que acontecem, com *feedback* sobre as mudanças implementadas após e discussão acerca de maneiras de evitar erros
	Respostas não punitivas ao erro	Investigação se funcionários sentem que seus erros e relatórios de erros não são usados contra eles e não são mantidos em seu arquivo pessoal
	Adequação de profissionais	Identificação da suficiência de pessoal para atender ao fluxo de trabalho com o melhor atendimento aos pacientes
RESULTADOS	Frequência de relatos de eventos	Relato de problemas de segurança do paciente e de eventos identificados ou erros antes que atinjam o paciente
	Percepção geral da segurança do paciente	Identificação se os sistemas e protocolos são suficientes na prevenção de erros

FONTE: REIS; MARTINS; LAGUARDIA (2016).

Além de mensurar as dimensões supracitadas, o HSOPSC visa à identificação de áreas em que a cultura necessita de melhorias, avaliação da efetividade das ações implementadas para o aperfeiçoamento da segurança, priorização dos esforços para fortalecimento da cultura de segurança e auxílio para organização de saúde na comparação de seus dados com os de outras organizações (SORRA; NIEVA, 2004; REIS, 2013). Na seção H, o instrumento avalia características profissionais e laborais dos participantes, como o tempo de trabalho no hospital; na unidade; carga horária semanal de trabalho; cargo; interação direta com os pacientes e tempo de trabalho na atual profissão. Na seção I, é possível

registrar comentários sobre a SP, erros ou comunicação de EA (SORRA; NIEVA, 2004).

Para garantir respostas consistentes, a AHRQ desenvolveu 18 perguntas formuladas negativamente identificadas pela letra “R” (A5 R, A7 R, A8 R, A10 R, A12 R, A14 R, A16 R, A17 R, B3 R, B4 R, C6 R, F2 R, F3 R, F5 R, F6 R, F7 R, F9 R, F11 R 9). Tais questões precisam escore reverso no cálculo das respostas, ou seja, quando o participante discordar do item estará respondendo positivamente (SORRA; NIEVA, 2004).

O HSPOC é um dos instrumentos mais utilizados mundialmente para mensurar a cultura de segurança do paciente nas unidades assistenciais, permite avaliar a opinião dos profissionais de saúde sobre questões relacionadas à SP, valores, crenças e normas da organização, relato de EA, comunicação, liderança e gestão. Este apresenta os melhores índices dos testes psicométricos, como confiabilidade, validade e eficiência, quando comparados com os demais, o que viabiliza a mensuração fiel e precisa do fenômeno a ser estudado (SORRA; NIEVA, 2004; REIS, 2013; REIS; LAGUARDIA; MARTINS, 2012; REIS; LAGUARDIA; VASCONCELOS, et al., 2016).

3.4.2 Revisão retrospectiva de prontuários

Os estudos que utilizam a técnica de revisão retrospectiva de prontuários para avaliação de incidência/prevalência de EA em hospitais tem o objetivo de chamar a atenção dos gerentes, profissionais e sociedade para identificação de áreas críticas, estabelecimento de prioridades, e desenvolvimento de estratégias e ações relacionadas à questão da segurança nos estabelecimentos de saúde (BRASIL, 2014).

Em estudo de revisão sistemática da literatura sobre avaliação da ocorrência de EA em hospitais de em nove países que utilizaram a metodologia de revisão retrospectiva de prontuários, verificou-se que os procedimentos cirúrgicos foram responsáveis pela maioria dos EA detectados. Os autores concluíram que entender o

fenômeno de ocorrência de EA na assistência à saúde é um importante passo para pensar em políticas que garantam a SP; e que a metodologia de revisão retrospectiva de prontuários pode ser útil em estudos em países que não possuem

informações eletronicamente disponíveis, como o Brasil (MENDES; PAVÃO; MARTINS et al., 2013).

Conforme indicação da OMS, a revisão retrospectiva de prontuários foi descrita no livro *To Err is Human: building a safer health care system* e é considerada um marco nas ações em prol da temática sobre SP (KOHN; CORRIGAN; DONALDSON, 2000) e a metodologia mais adequada para coleta de dados (*gold standard*) na avaliação de EA (OMS, 2009).

3.4.3 Metodologia *Global Trigger Tool*

Uma, dentre outras metodologias, utilizadas em vários países para monitorar e mensurar as taxas de EA e melhorar os processos de trabalho para segurança do paciente é denominada *Global Trigger Tool (GTT)*. Esta foi desenvolvida no ano 2000 pelo *Institute of Healthcare Improvement* (IHI) dos Estados Unidos e consiste na revisão retrospectiva de amostra aleatória de prontuários de pacientes, utilizando-se de formulário contendo gatilhos, denominados *triggers*, que servem como rastreadores para identificação de potenciais EA. O instrumento é composto por seis módulos identificado por letras: Cuidados (C) com 15 itens; Medicamentos (M) com 13 itens; Cirúrgico (S) com 11 itens; Cuidado Intensivo (I) com quatro itens; Assistência Perinatal (P) com oito itens; e Emergência (E) com dois itens, totalizando 53 itens (GRIFFIN; RESAR, 2009).

Rastrear os EA ao longo do tempo é metodologia útil para conhecimento das ações e mudanças que são realizadas nas instituições hospitalares. Neste contexto, foi elaborado o manual denominado *IHI Global Trigger Tool for Measuring Adverse Events* para fornecer informações sobre a metodologia, com descrições e orientações de cada gatilho e o que os revisores devem procurar nos prontuários dos pacientes para determinar a ocorrência de EA (GRIFFIN; RESAR, 2009).

3.4.4 Protocolo *Canadian Adverse Events Study (CAES)*

Outra metodologia semelhante ao proposto pelo IHI, para a identificação de EA por meio de revisão de prontuários e critérios de rastreamento implícitos e explícitos, é preconizada a partir do protocolo desenvolvido pelo *Canadian Adverse*

Events Study (CAES), com a utilização de instrumento de coleta de dados que contém 19 rastreadores (BAKER; NORTON; FLINTOFT et al., 2004). Os critérios foram traduzidos, adaptados e validados para o Brasil e estão relacionados à ocorrência de incidentes pertinentes ao cuidado clínico, cirúrgico, obstétrico, em caráter eletivo, urgência e emergência e intensivo (MENDES; TRAVASSOS; MARTINS et al., 2008)

O CAES foi o primeiro estudo canadense realizado para avaliar a ocorrência de EA em cuidados de saúde e, neste, o termo EA foi definido como uma lesão ou complicação involuntária causada pela gestão de cuidados em saúde, em vez da doença ou doença subjacente de um paciente, que pode originar complicações com sequelas, permanência hospitalar prolongada e morte (BAKER; NORTON; FLINTOFT et al., 2004).

Na revisão retrospectiva de 3700 prontuários, em 20 hospitais, dos 2,5 milhões de internações hospitalares, verificou-se que 185 mil estavam associados à ocorrência de EA. Tal estudo evidenciou que 7,5% dos pacientes internados em hospitais canadenses foram vítimas de EA, sendo que 37% destes foram considerados evitáveis e 20,08% ocasionaram mortes. Os pesquisadores sugerem que a melhoria da qualidade e SP depende de modificações no ambiente de trabalho dos profissionais de saúde, com a finalidade de originar melhores defesas para evitar os EA e minimizar seus efeitos (BAKER; NORTON; FLINTOFT et al., 2004).

No Brasil, os formulários de avaliação do *CAES*, contidos no *Manual for the Canadian Adverse Study*, é composto por cinco documentos e 340 páginas. Os instrumentos de avaliação, respectivamente denominados de Formulário de Rastreamento e Formulário de Avaliação, passaram por processo de tradução e adaptação, que foi realizado em quatro etapas: tradução do inglês para o português; painel de especialistas para julgamento e consenso dos critérios de rastreamento e solução das dúvidas na tradução; pré-teste e estudo piloto e retrotradução dos formulários. A adaptação dos instrumentos do CAES ao contexto dos hospitais brasileiros mostrou desempenho satisfatório na avaliação dos EA por meio da revisão retrospectiva de prontuários e pode ser empregado em associação com outros métodos na avaliação (MENDES; TRAVASSOS; MARTINS et al., 2008).

4 METODOLOGIA

4.1 CARACTERIZAÇÃO DA PESQUISA

Estudo analítico, com delineamento transversal, de abordagem quantitativa e coleta mista de dados: prospectiva para avaliação da cultura de segurança e retrospectiva para análise de EA cirúrgicos. Desenvolveu-se em duas fases principais: a primeira fase para avaliação da cultura de segurança em centros cirúrgicos e unidades de internação cirúrgicas. A segunda fase para tradução, adaptação e validação para língua portuguesa do Brasil, de instrumento utilizado na coleta de dados para rastreamento de EA cirúrgicos, por meio de pesquisa metodológica; e simultânea análise de prontuários.

O estudo analítico são aqueles delineados para examinar a existência de associação entre uma exposição e uma doença ou condição relacionada à saúde (HOCHMAN; NAHAS; OLIVEIRA FILHO et al, 2005), sem intervir diretamente na relação analisada e com tratamento estatístico dos dados coletados da pesquisa (ROUQUAYROL; SILVA, 2003). Este tipo de estudo tem como propósito a obtenção de medidas dos fatores de risco e dos efeitos ou a avaliação de procedimentos terapêuticos ou preventivos, para teste de hipóteses (PEREIRA, 2008).

Em relação à sequência temporal, o estudo é considerado transversal quando se faz o levantamento simultâneo da causa e do efeito, em único recorte de tempo. (PEREIRA, 2008); descreve uma situação ou fenômeno, presença de doença ou transtorno (HOCHMAN; NAHAS; OLIVEIRA FILHO et al., 2005). Apresenta-se como uma fotografia ou corte instantâneo que se faz numa população por meio de uma amostragem, examinando-se nos integrantes da casuística ou amostra, a presença ou ausência da exposição e a presença ou ausência do efeito ou doença (HADDAD, 2004). A condução de um estudo transversal envolve as seguintes etapas: definição de uma população de interesse, estudo de amostragem de parte da população e determinação da presença ou ausência de desfecho e da exposição para os indivíduos estudados (ROUQUAYROL; SILVA, 2003).

A abordagem quantitativa tem suas raízes no pensamento positivista lógico, tende a enfatizar o raciocínio dedutivo, as regras da lógica e os atributos mensuráveis da experiência humana. Este tipo de abordagem utiliza instrumentos formais para coleta de dados e é caracterizada pelo emprego da quantificação nas modalidades de coleta de dados e no seu tratamento, por meio de técnicas estatísticas e foco na dimensão mensurável dos fenômenos para traduzi-los em números, opiniões e informações (POLIT; BECK, 2011).

4.2 LOCAL DO ESTUDO

Esta pesquisa foi realizada em três hospitais gerais e de ensino nível II, de alta complexidade, localizados na região leste do Estado do Paraná, Brasil. As instituições foram nominadas como “Hospital A”, “Hospital B” e “Hospital C”. A seleção dos hospitais participantes, todos prestadores de serviços ao Sistema Único de Saúde, (SUS), foi por conveniência, com vistas a assegurar a investigação daqueles de maior porte, instalados no município do estudo.

As ações foram desenvolvidas nas unidades de centro cirúrgico geral e em cinco unidades de internações cirúrgicas das seguintes especialidades: Ortopedia e Traumatologia; Cirurgia Geral; Cirurgia do Aparelho Digestivo; Neurocirurgia, Cirurgia Plástica e Transplante Hepático. Estas especialidades cirúrgicas foram selecionadas por comporem a Unidade Gerencial Cirúrgica (UCIR) e representarem, aproximadamente, 37,8% do total de procedimentos cirúrgicos do “Hospital A”, local onde se iniciou esta pesquisa.

Os hospitais participantes são classificados como hospitais de ensino (HE). As regras em vigor para uma instituição hospitalar qualificar-se para este tipo de especialidade, segue as normas das portarias interministeriais 2.400/2007 e 285/2015. Para adquirir a certificação de HE, a organização deve atender alguns requisitos estruturais como: possuir instalações adequadas, disponibilizar um preceptor com carga horária de 20 horas semanais para cada doze alunos de internato, ou três residentes (BRASIL, 2007; BRASIL, 2015).

A portaria nº 285 de 24 de março de 2015 redefine o programa de certificação de HE e no seu artigo segundo considera-se:

> I - Hospitais de Ensino (HE): estabelecimentos de saúde que pertencem ou são conveniados a uma Instituição de Ensino Superior (IES), pública ou privada, que sirvam de campo para a prática de atividades de ensino na área da saúde e que sejam certificados conforme o estabelecido nesta Portaria (BRASIL, 2015, p.31).

Para ser certificado como HE nível I precisam atender aos seguintes critérios: possuir internato médico curricular obrigatório por área de atuação, ou no mínimo, 20 alunos respeitando a carga horária de até 35% do total de horas do curso de medicina; se houver programa de residência no município, ser cenário prático para atividades hospitalares de residência de medicina geral de família e comunidade; ofertar ao menos dois programas de residência médica, próprios ou conveniados, em áreas prioritárias para o SUS, com número mínimo de vagas anuais no percentual de 8% de seu número total de leitos operacionais; garantir acompanhamento diário por docente ou preceptor para os estudantes de graduação e para os programas de residência, respeitando um preceptor com carga horária de 20 horas semanais para cada três residentes, ou um preceptor com carga horária de 40 horas semanais para cada seis residentes, e um preceptor para cada 12 alunos de internato (BRASIL, 2015).

Como HE nível II, para receber certificação deverão cumprir, além do preconizado para certificação nível I, obrigações específicas como: desenvolver atividades curriculares de, no mínimo, três cursos de graduação na área da saúde, obrigatoriamente a enfermagem com carga horária de até 20% do total da carga horária do curso; oferecer programas de residência médica e multiprofissional, sendo pelo menos quatro programas de residência médica em áreas prioritárias para o SUS, um programa de residência multiprofissional e obrigar programa de graduação stricto sensu em área da saúde; dispor de UTI nível II ou III; possuir ao menos uma habilitação em alta complexidade para maternidade de referência para gestante ou recém-nascido de alto risco, oncologia, ortopedia, neurologia, cardiologia, transplantes e terapia renal substitutiva; e executar as ações previstas no Programa Nacional de Segurança do Paciente, com Núcleo de Segurança do Paciente em funcionamento, Planos de Segurança do Paciente implementados, e no mínimo de quatro protocolos de Segurança do Paciente (BRASIL, 2015).

O QUADRO 5 descreve informações gerais sobre os hospitais participantes desta pesquisa.

QUADRO 5 – INFORMAÇÕES GERAIS DOS HOSPITAIS PARTICIPANTES DA PESQUISA.

(continua)

CARACTERÍSTICAS	HOSPITAL A	HOSPITAL B	HOSPITAL C
Data de inauguração e classificação	Inaugurado em 1961 e constitui o maior hospital público do Paraná e o terceiro maior HE federal do país.	Inaugurado em 1947 e reconhecido como o maior hospital universitário particular e de caráter filantrópico do Estado do Paraná.	Inaugurado em 1959, instituição estadual caracterizada por gestão tripartite: participação do Governo Federal (UFPR e sua Fundação – Funpar), Governo Estadual (SESA – Paraná) e Governo Municipal (SMS – Curitiba.
Nº funcionários ano 2018.	- 584 médicos<br>747 Funcionários Fundação, (inclusive médicos)<br>- 1.645 Funcionários Ministério da Educação (inclusive médicos)<br>- 881 Funcionários EBSERH (inclusive médicos)<br>484 Funcionários Terceirizados<br>- 369 residentes médicos<br>- 118 residentes Multiprofissional<br><br>TOTAL: 3757 funcionários e 487 residentes	- 450 médicos<br>- 1.800 profissionais na área assistencial e administrativa<br>- 160 residentes médicos<br><br>TOTAL: 2250 funcionários e 160 residentes	- 359 médicos<br>- 530 profissionais de enfermagem (enfermeiros, técnicos e auxiliares) - 644 que pertencem aos serviços assistenciais e administrativos.<br>- 50 residentes médicos<br><br>TOTAL: 1533 funcionários e 50 residentes
Nº de leitos	632 leitos SUS	475 leitos (416 leitos SUS e 59 leitos Convênios/Particular)	222 leitos SUS
Tipos de atendimentos	Exclusivamente SUS*	SUS* (383 leitos) e privado/convênios (56 leitos)	Exclusivamente SUS*
	Pronto Atendimento (PA) para atendimento de urgência e emergência obstétrico e ginecológico e pediatria (não atende trauma).	Pronto Socorro 24 horas para atendimento de emergências com porta de entrada aberta (demanda espontânea ou referenciada via SIATE†/SAMU‡).	Pronto Socorro 24 horas para atendimento de emergências com porta de entrada aberta (demanda espontânea ou referenciada via SIATE†/SAMU‡).

QUADRO 5 – INFORMAÇÕES GERAIS DOS HOSPITAIS PARTICIPANTES DA PESQUISA.

(conclusão)

Tipo de atendimentos	- Atendimento de urgências (casos clínicos e cirúrgicos encaminhados pelas Unidades de Pronto Atendimento-UPAS)<br>- Atendimento obstétrico e ginecológico de urgência e emergência (encaminhamento da Unidade de Saúde 24h – Centro Municipal de Urgências Médicas - CMUM, Unidade Básica de Saúde ou Mãe Curitibana)<br>- Atendimento ambulatorial (usuários encaminhados pelas Unidades de Saúde)	- Atendimentos de urgência e emergência (trauma, acidentes em geral, vítimas de queimaduras, gestação de alto risco)<br><br>- Atendimento ambulatorial (retornos do Pronto Socorro e usuários encaminhados pelas Unidades de Saúde).	- Atendimentos de urgência e emergência (trauma, acidentes de trabalho e acidentes em geral)<br><br>- Atendimento ambulatorial geral (retornos do Pronto Socorro e usuários encaminhados pelas Unidades de Saúde);<br><br>- Atendimento Ambulatório de Saúde do Trabalhadorna especialidade de ortopedia (atender à demanda de pacientes do Centro Metropolitano de Apoio à Saúde do Trabalhador – CEMAST).
Nº cirurgias/mês<br>Nº de leitos cirúrgicos Nº salas cirúrgicas<br>(Ano 2018)	893 cirurgias/mês	1530 cirurgias/mês	1400 cirurgias/mês
	10 salas cirúrgicas e 220 leitos para clínica cirúrgica	16 salas cirúrgicas e 60 leitos para clínica cirúrgica	Nove salas cirúrgicas e 76 leitos para clínica cirúrgica
Núcleo de Segurança do Paciente	SIM (implantação desde 2010)	SIM (implantação desde 2012)	SIM (implantação desde 2013)

Dados obtidos nos sites oficiais dos hospitais participantes desta pesquisa, nos respectivos serviços de informática e no site do Cadastro Nacional dos estabelecimentos de Saúde (CNES) do Ministério da Saúde. (BRASIL, 2018c). *SUS = Sistema Único de Saúde; †SIATE = Serviço Integrado de Atendimento ao Trauma em Emergência; ‡SAMU = Serviço de Atendimento Móvel de Urgência. Dados da média de cirurgias ao ano (2018) - Hospital A=10.716 cirurgias/ano; Hospital B=18.361 cirurgias/ano; Hospital C=16.805 cirurgias/ano.
Fonte: A autora (2019).

Como nesta pesquisa realizou-se a avaliação da cultura de segurança, torna-se essencial descrever a missão, visão e valores dos hospitais participantes.

O Hospital A tem por missão ser referência de excelência na formação de profissionais de saúde, na atenção à saúde, na inovação tecnológica e sustentabilidade, atendendo com inserção e integração na rede de serviços de saúde e nas necessidades da população. Tem como visão ser o melhor complexo hospitalar público de ensino e assistência em saúde, em todos os níveis de atenção, do país, acreditado com excelência. Seus valores são: Comportamento ético, Compromisso com o Ensino e Pesquisa, Gestão participativa e Sustentável, Humanização, Qualidade e Segurança.

O Hospital B tem como missão contribuir para a qualidade de vida do cidadão e da comunidade, desenvolvendo, em nível de excelência, ações de saúde voltadas à prevenção, assistência, reabilitação, ensino e pesquisa, nas áreas de trauma e emergência, saúde do trabalhador, materno-infantil e infectologia. Tem como visão executar a gestão com qualidade, tornando o melhor Centro Público de Assistência Hospitalar à Traumatologia do país em 10 anos (2010-2020). Seus valores incluem acolhimento, ética profissional, comprometimento e foco no resultado, qualidade e segurança do paciente, desenvolvimento técnico-científico e respeito ao meio ambiente e responsabilidade social.

O Hospital C tem como missão prestar assistência médico-hospitalar a quem o procura e promover ensino e pesquisa na área da saúde, utilizando-se de profissionais capacitados e das melhores técnicas possíveis, para melhorar a qualidade de vida das pessoas, de acordo com os princípios cristãos. Este hospital foi criado e é mantido por uma sociedade que tem como valores a solidariedade, o exercício da misericórdia, do amor, da socialização, da espiritualidade, cuidado pastoral sem distinção social/político/religiosa.

A seguir, apresenta-se os participantes do estudo em dois grupos, conforme os dois principais objetos de investigação: cultura de segurança, que envolveu os profissionais de saúde que trabalham no centro cirúrgico e/ou nas unidades de internação cirúrgicas (participantes do grupo 1) e ocorrência de EA cirúrgicos, que foram avaliados em prontuários dos pacientes cirúrgicos (participantes do grupo 2).

4.3 CULTURA DE SEGURANÇA – GRUPO 1

4.3.1 População do estudo

O grupo 1 foi composto por profissionais de saúde que trabalham no centro cirúrgico e/ou nas unidades de internação cirúrgicas. Compreenderam os médicos cirurgiões e médicos anestesiologistas, médicos residentes em cirurgia e anestesiologia, enfermeiros, auxiliares e técnicos de enfermagem. Estes profissionais foram recrutados no próprio ambiente de trabalho e foram convidados a responder o instrumento *Hospital Survey on Patient Safety Culture (HSOPSC)* - versão brasileira (ANEXO A).

Os respectivos profissionais foram identificados por meio da disponibilização das listas fornecidas pelas chefias dos serviços participantes, para identificação por função e serviço de lotação, e determinação do número de profissionais envolvidos na pesquisa; todos os constantes das listas foram convidados a participar, caracterizando-se como amostra não probabilística intencional.

4.3.2 Critérios de inclusão e exclusão

Os critérios de inclusão dos participantes da pesquisa foram: profissionais lotados e atuantes nos centros cirúrgicos e/ou unidades cirúrgicas dos hospitais participantes, durante o período de coleta de dados; com carga horária mínima semanal de trabalho de 20 horas; contato direto ou interação com pacientes cirúrgicos, e aqueles sem contato direto com o paciente, mas com função de liderança, gerência ou supervisão (ROCKVILLE; SORRA; GRAY, et al., 2016).

Foram excluídos da análise os participantes cujos questionários tiveram menos de 50% de perguntas preenchidas e/ou pelo menos uma seção com todas as perguntas não preenchidas, e os com respostas iguais para todas as dimensões do instrumento (ROCKVILLE; SORRA; GRAY, et al., 2016).

4.3.3 Coleta de dados

A coleta de dados foi realizada pela pesquisadora e dois discentes do curso de mestrado em enfermagem, previamente capacitados pela professora orientadora do projeto; e ocorreu nos meses de maio de 2017 até março de 2018, por intermédio do preenchimento de questionário autoaplicável *HSOPSC* - versão brasileira (ANEXO A).

O questionário é constituído por 50 itens, 44 específicos para cultura de segurança e seis relacionados a informações pessoais, distribuídos em nove seções, dispostas da letra A à I. Os temas são agrupados em 12 dimensões, três relacionadas ao hospital, sete relacionadas à unidade de trabalho dentro do hospital e duas variáveis de resultados. A maioria das respostas é avaliada a partir de escala de *Likert* de cinco pontos, com categorias de respostas em grau de concordância (ROCKVILLE; SORRA; GRAY, et al., 2016).

O HSOPSC contém 18 itens formulados negativamente, identificados pela letra “R” (A5R, A7R, A8R, A10R, A12R, A14R, A16R, A17R, B3R, B4R, C6R, F2R, F3R, F5R, F6R, F7R, F9R, F11R) e quando o participante discorda do item está opinando de forma positiva (ROCKVILLE; SORRA; GRAY, et al., 2016).

Os dados foram coletados nas dependências dos centros cirúrgicos e unidades de internação cirúrgicas, em locais previamente designados pelas chefias dos serviços. O convite e esclarecimentos sobre a pesquisa realizaram-se de forma individual e/ou em grupo, e os pesquisadores informaram os participantes sobre o tempo estimado para preenchimento do instrumento, em média de 15 minutos (SORRA; GRAY; STREAGLE et al., 2016; REIS; LAGUARDIA; MARTINS, 2012; REIS; LAGUARDIA; VASCONCELOS., et al., 2016). Os participantes foram concentrados em duas categorias profissionais: equipe médica e equipe de enfermagem

O profissional que aceitou participar da pesquisa recebeu envelope com o HSOPSC versão brasileira (ANEXO A), duas vias do Termo de Consentimento Livre e Esclarecido- TCLE (APÊNDICE 1) e caneta esferográfica azul. Foram disponibilizados impressos do instrumento e, opcionalmente, o uso de dispositivo eletrônico móvel, sem conexão com a internet, com o HSOPSC - versão brasileira, na plataforma do aplicativo *QuickTapSurvey*

4.3.4 Análise dos dados

Os dados foram repassados para planilha no *Microsoft Office Excel* 2016®, por dupla digitação, para correção de inconsistências e posterior processamento e análise. Realizou-se a verificação de completude das respostas para identificação de questionários com respostas incompletas e inelegíveis, para posterior análise estatística descritiva em medidas de proporção absoluta e relativa das variáveis categóricas (ROCKVILLE; SORRA; GRAY, et al., 2016).

Em relação às variáveis quantitativas, para associação entre os três hospitais participantes, utilizou-se o modelo de análise da variância (ANOVA) com um fator ou o teste não-paramétrico de Kruskal-Wallis. Em relação às variáveis categóricas, as comparações foram feitas usando-se o teste de Qui-quadrado; valores de $p<0,05$ indicaram significância estatística. Os dados foram analisados com o programa computacional IBM SPSS *Statistics* v.20.0. Armonk, NY: IBM Corp.

Para a análise descritiva os itens foram agrupados em respostas positivas (opções 4 ou 5 - concordo/concordo totalmente ou quase sempre/sempre) para as sentenças formuladas de forma positiva ou, nas perguntas formuladas negativamente (opções 1 ou 2 - discordo/ discordo totalmente ou nunca/raramente. A opção 3 classifica as respostas neutras (nem discordo nem concordo ou às vezes) para qualquer pergunta. Respostas negativas foram aquelas assinaladas nas opções 1 ou 2 (discordo/discordo totalmente ou nunca/ raramente) para as perguntas formuladas de forma positiva, ou nas opções 4 ou 5 (concordo/concordo totalmente ou quase sempre/ sempre), nas sentenças formuladas negativamente (SORRA; GRAY; STREAGLE et al., 2016).

Considerou-se dimensão forte para a SP quando houve ≥75% de respostas positivas. Resultados ≤50% indicaram fragilidade e necessidade de melhorias nos processos de trabalho e realização de cuidados seguros. Respostas neutras foram consideradas como aquelas cujo percentual de resposta positivas variou entre 51 e 74% (SORRA; NIEVA, 2004).

Após a inversão dos itens reversos, o percentual de respostas positivas para cada dimensão e para cada item que compõe as dimensões do instrumento foram definidos utilizando-se as fórmulas propostas por Sorra, Gray, Streagle et al., (2016), conforme descritas nas FIGURAS 2 e 3:

FIGURA 2 –FÓRMULA PERCENTUAL DE RESPOSTAS POSITIVAS PARA AS DIMENSÕES

$$\text{\% de respostas positivas da dimensão X} = \frac{\text{número de respostas positivas da dimensão X}}{\text{número total de respostas válidas aos itens da dimensão X (positivas, neutras e negativas, excluindo-se os dados ausentes)}} \times 100$$

FONTE: SORRA, GRAY, STREAGLE et al., (2016).

FIGURA 3 – FÓRMULA PERCENTUAL DE RESPOSTAS POSITIVAS PARA OS ITENS DAS DIMENSÕES

$$\text{\% de respostas positivas ao item da dimensão X} = \frac{\text{número de respostas positivas ao item da dimensão X}}{\text{número total de respostas válidas ao item da dimensão X (positivas, neutras e negativas, excluindo-se os dados ausentes)}} \times 100$$

FONTE: SORRA, GRAY, STREAGLE et al., (2016).

Conforme recomendação da AHRQ, realizou-se a avaliação da consistência interna do questionário, com o cálculo do Alfa de *Cronbach*. Este coeficiente mede a correlação entre as respostas, quando as opções são escalonadas (Discordo Totalmente, Discordo, Indiferente, Concordo e Concordo Totalmente), com valores que variam de 0 a 1, estabelecendo a seguinte atribuição de confiabilidade: > 0,90 = excelente; > 0,80 = bom; > 0,70 = aceitável; > 0,60 = questionável; > 0,50 = pobre; < 0,50 = inaceitável. (GEORGE; MALLERY, 2003).

A seguir, o FLUXOGRAMA 1 demonstra as etapas metodológicas para avaliação da cultura de segurança nos três hospitais participantes da pesquisa.

FLUXOGRAMA 1 – METODOLOGIA PARA AVALIAÇÃO DA CULTURA DE SEGURANÇA ORGANIZACIONAL – GRUPO 1.

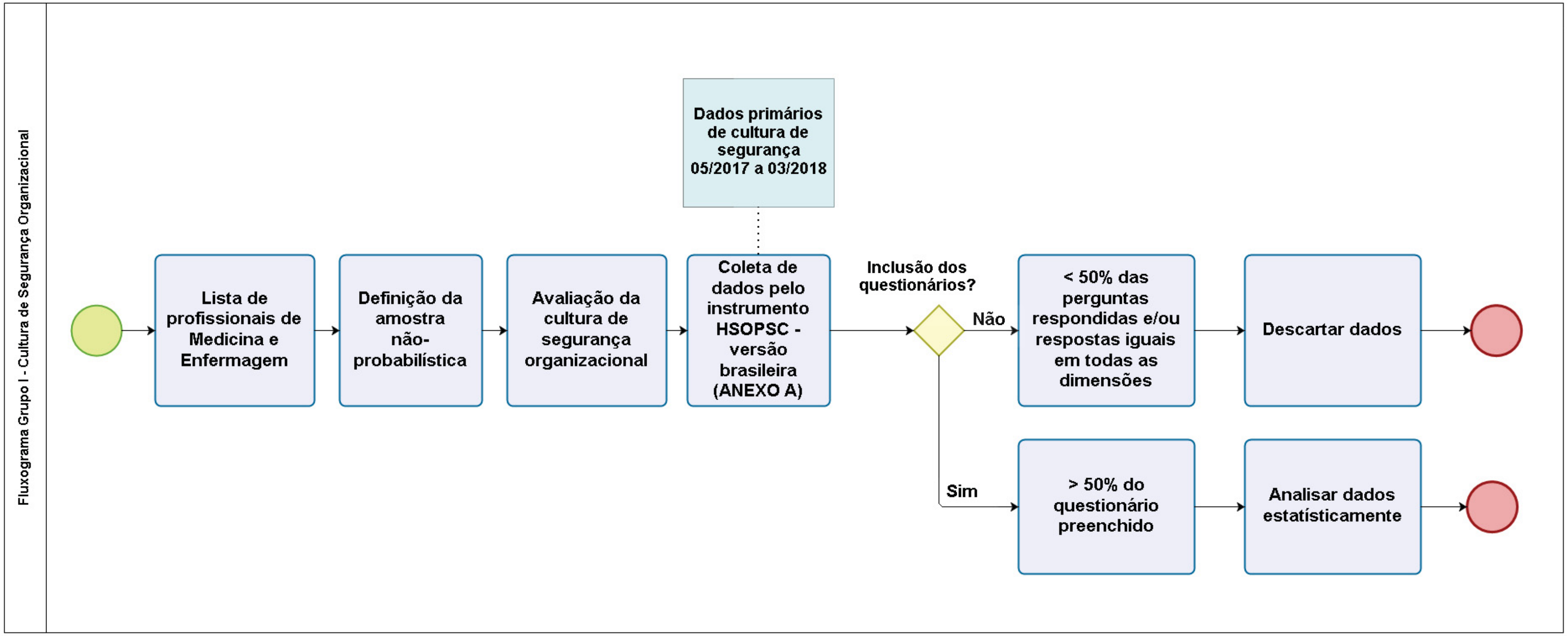

FONTE: A autora (2019).

4.4 EVENTOS ADVERSOS CIRÚRGICOS – GRUPO 2

4.4.1 População do estudo e Amostra

Em virtude da coleta retrospectiva de dados nos prontuários, os pacientes não foram abordados diretamente. Pela seleção de amostra aleatória/probabilística simples, foram analisados os prontuários selecionados a partir da lista do total de cirurgias/procedimentos cirúrgicos[3] realizadas entre junho de 2016 e maio de 2017 emitidas pelo serviço de informática dos hospitais participantes desta pesquisa.

Foi selecionada amostra aleatória/probabilística simples. Conforme estudo prévio realizado pela OMS (2009) para o cálculo amostral considerou-se a incidência de complicações cirúrgicas de 16%, erro amostral máximo de 5% e nível de significância de 5%, conforme FIGURA 4:

FIGURA 4 – FÓRMULA PARA O CÁLCULO AMOSTRAL

$$n = \frac{N.\ Z^2.\ p.(1-p)}{Z^2.\ p.\ (1-p) + e^2.\ (N-1)}$$

Onde:

n – amostra calculada

N – população

Z – variável normal padronizada associada ao nível de confiança

p – verdadeira probabilidade do evento

FONTE: BUSSAB; MORETTIN (2017).

---

[3] Os procedimentos cirúrgicos foram baseados na Portaria nº 2.848/GM/MS que consolida e detalha os Procedimentos, Medicamentos, Órteses, Próteses e Materiais Especiais do Sistema Único de Saúde - SUS, e 06/11/2007, grupo: 04 –procedimentos cirúrgicos e, no conceito de cirurgia maior adotado pela OMS como “qualquer procedimento na sala de operações envolvendo incisão, excisão, manipulação ou sutura de tecido que geralmente requer anestesia regional ou geral ou sedação profunda para controle da dor”. (OMS, 2009, p. 13).

O profissional estatístico realizou o cálculo do número da amostra de cada hospital e elaborou a lista dos pacientes, conforme amostra aleatória/probabilística simples. Os prontuários que foram sorteados aleatoriamente para compor a amostra e que se encontravam inelegíveis ou indisponíveis no serviço de arquivamento foram substituídos pelos prontuários/registros imediatamente subsequentes da lista geral de cirurgias/procedimentos cirúrgicos.

4.4.2 Critérios de inclusão e exclusão

Os dados da população de pacientes foram coletados por meio da revisão retrospectiva de prontuários, para verificação da ocorrência de EA cirúrgicos (grupo 2), e considerou-se os seguintes critérios de inclusão: primeiro procedimento cirúrgico na internação índice[4] realizado pela especialidade cirúrgica relativa aos serviços incluídos na pesquisa (cirurgia geral e do aparelho digestivo, ortopedia e traumatologia, neurocirurgia, cirurgia plástica e transplante hepático), em paciente com idade ≥18 anos e com tempo de internação mínima de 24 horas. Foram excluídos da amostra prontuários de pacientes psiquiátricos (MENDES; MARTINS; ROZENFELD et al., 2009).

4.4.3 Coleta de Dados

A revisão retrospectiva dos prontuários ocorreu entre os meses de julho à setembro de 2017 para análise dos dados no Hospital A; entre março à abril de 2018 no Hospital B e entre janeiro e fevereiro de 2019 no Hospital C, nas dependências do Serviço de Arquivo Médico e Estatística para avaliação de prontuários físicos (Hospital A) e Salas de Estudo destinadas para análise de prontuários eletrônicos (Hospitais B e C).

Foram considerados os dados que possibilitaram a avaliação da ocorrência de EA pela revisão retrospectiva de prontuários, conforme protocolo desenvolvido pelo *Canadian Adverse Events Study (CAES)* que estabelece duas fases de investigação: a fase 1 refere-se ao rastreamento dos potenciais EA (pEA) por meio de critérios explícitos, com a revisão dos prontuários por enfermeiros. A fase 2

[4] Internação relativa ao período no qual foi realizado o procedimento cirúrgico. Reoperações na internação índice não foram consideradas na amostra.

refere-se à confirrmação, ou não, dos EA, por revisão estruturada implícita realizada por profissional médico (MENDES; MARTINS; ROZENFELD et al., 2009).

Nesta pesquisa, a busca, identificação e registro de pistas de EA foram executados pela pesquisadora e um mestrando participante da pesquisa no Hospital A e, pela pesquisadora nos Hospitais B e C, utilizando-se o formulário de rastreamento de pEA preconizados pelo protocolo *Canadian Adverse Events Study – CAES* (BAKER; NORTON; FLINTOFT, et al., 2004), traduzido e adaptado transculturalmente para o contexto brasileiro. (MENDES; TRAVASSOS; MARTINS, et al., 2008). O protocolo foi desenvolvido por pesquisadores canadenses, contém 18 critérios relacionados a procedimentos cirúrgicos e anestésicos, medicamentos, diagnóstico, cuidados, tratamento não medicamentoso e incidentes obstétricos. (BAKER; NORTON; FLINTOFT, et al., 2004).

Durante o processo de tradução e adaptação realizado por Mendes, Travassos, Martins, et al., (2008) foram excluídos da versão original dois critérios relacionados à reinternações hospitalares, e incluído um critério de busca para identificar pacientes com injúrias renais, totalizando 17 rastreadores. Esta versão foi utilizada na presente pesquisa (ANEXO B). Cabe ressaltar que os rastreadores relacionados ao aborto, trabalho de parto e parto foram excluídos do anexo, considerando os critérios ora utilizados.

De forma a complementar, para a identificação de pEA, foi utilizado o módulo cirúrgico do GTT desenvolvido pelo IHI (ANEXO C). Este propõe 11 gatilhos denominados *triggers*, que auxiliam na identificação de pEA ocorridos no intra e pós-operatório. (GRIFFIN; RESAR, 2009). Justifica-se o uso deste instrumento para minimizar a subnotificação de dados pelos revisores na fase 1 e, por tratar itens específicos para rastreamento de EA em ambientes cirúrgicos, contribuindo para o processo de identificação de eventos na primeira fase de revisão de prontuários.

Os revisores da fase 1 preencheram o Formulário de Investigação de EA Cirúrgicos (ANEXO D), desenvolvido por Mendes (2008) e adaptado para esta pesquisa e o roteiro semiestruturado para coleta dos dados referente ao perfil demográfico, cirúrgico e anestésico dos pacientes (APÊNDICE 2).

Para a identificação dos pEA relacionados à infecção de sítio cirúrgico (ISC), ocorridos após a alta hospitalar, foram analisados dados contidos nas fichas de consultas ambulatoriais (registros). Considerou-se os critérios recomendados pela ANVISA e pelos *Centers for Disease Control and Prevention,* os quais definem ISC como aquela que ocorre em até 30 dias após o procedimento cirúrgico e/ou 90 dias após inserção de implante. (BRASIL, 2017c; CENTERS FOR DISEASE CONTROL AND PREVENTION (CDC, 2017a).

A Fase 2 do processo de investigação ocorreu entre outubro e novembro de 2017 no Hospital A, entre maio e junho de 2018 no Hospital B e entre março e abril de 2019 no Hospital C, por intermédio da participação de uma médica com tempo de atuação superior há 20 anos na área de gestão da qualidade e dois enfermeiros com expertise na área de SP. Este grupo formou o comitê de especialistas para julgar os pEA identificados na fase anterior. Neste contexto, utilizou-se o conceito de EA proposto pela OMS como o incidente que resultou em dano ao paciente para a análise dos casos (WHO, 2009).

Após a confirmação da ocorrência do EA em consenso do comitê de especialistas, foi realizada a segunda avaliação dos casos com a finalidade de identificar e registrar as evidências clínicas confirmatórias do caso. Para tal, foram utilizadas duas escalas: a primeira para julgar se a lesão ou dano foi causado pela assistência prestada ao paciente, e a segunda para avaliar seu grau de evitabilidade (ANEXO E). As escalas possuem seis pontos, e considerou-se como EA evitável quando a pontuação alcançou, no mínimo, quatro pontos (MENDES; MARTINS; ROZENFELD, et al., 2009). Por último, foi avaliado o grau de dano do EA (ANEXO F) de acordo com o preconizado pela OMS (WHO, 2009).

4.4.4 Análise de Dados

Em relação aos EA cirúrgicos foram analisados os critérios de rastreamento positivos encontrados nos prontuários; a prevalência de EA cirúrgicos por especialidade; o tipo de EA e reinternação hospitalar, ou não, em decorrência do EA.

As variáveis demográficas, clínicas, cirúrgicas e anestésicas, informadas nos registros do paciente e coletadas foram: sexo (masculino/feminino), idade (<60 anos; ≥60 anos), tempo de permanência hospitalar (1-2 dias; 3-5 dias; ≥6 dias); tempo de internamento pré-operatório (<24 horas; ≥24 horas); classificação da cirurgia (eletiva;

emergência); potencial de contaminação (limpa; potencialmente contaminada; contaminada; infectada); risco cirúrgico de acordo com classificação da *American Society of Anesthesiology* – ASA[5]; tipo de anestesia (geral; regional; combinada);

As medidas utilizadas foram: incidência de EA cirúrgico entre os pacientes internados [(número de pacientes com pelo menos um EA cirúrgico/número total de pacientes) x 100]; proporção de EA cirúrgicos evitáveis [(número de EA cirúrgicos evitáveis/número total de EA cirúrgicos) x 100].

Os EA cirúrgicos foram classificados nas categorias: administração clínica; processo clínico/procedimento; documentação; infecção relacionada à assistência à saúde (IRAS), medicação/fluidos endovenosos; sangue/hemoderivados; nutrição; oxigênio/gases/vapores; dispositivos/equipamentos médicos; comportamento; acidentes com o paciente; infraestrutura/local/instalações; e recursos/gestão organizacional (WHO, 2009). O grau de dano do EA foi agrupado em leve, moderado, grave e óbito (WHO, 2009).

Para a análise de evitabilidade dos EA cirúrgicos considerou-se a classificação: fortemente prevenível, potencialmente prevenível, potencialmente não prevenível e fortemente não prevenível, adaptado de Mendes Junior (2007).

4.4.5 Análises estatísticas

Resultados de variáveis quantitativas foram descritos por média, desvio padrão, mediana, valor mínimo e valor máximo. As variáveis qualitativas foram apresentadas

---

[5] O ASA foi desenvolvido para oferecer uma classificação simples do estado fisiológico do paciente, podendo ser útil na predição do risco cirúrgico. O estado físico do paciente pode estar classificado em uma das 6 categorias: ASA I – saudável; ASA II – doença sistêmica leve; ASA III – doença sistêmica grave sem risco de morte; ASA IV – doença sistêmica grave com risco de morte; ASA V – Paciente moribundo, com expectativa de sobrevida mínima, independente da cirurgia; ASA VI – doador de órgãos (morte cerebral). Acrescentar a letra "E" para indicar cirurgias de emergência. (DOYLE; GARMON, 2017).

em frequências absolutas e relativas. Para verificar a associação entre os três hospitais, em relação às variáveis quantitativas, utilizou-se modelo de análise da variância ANOVA com um fator ou o teste não-paramétrico de Kruskal-Wallis e Fisher ou teste de Qui-quadrado.

Os dados foram analisados por profissional estatístico com auxílio do programa computacional IBM SPSS *Statistics* v.20; valores de $p<0,05$ indicaram significância estatística. A seguir, a metodologia para avaliação dos EA cirúrgicos nos hospitais participantes está representada no FLUXOGRAMA 2.

FLUXOGRAMA 2 – METODOLOGIA PARA AVALIAÇÃO DE EVENTOS ADVERSOS CIRÚRGICOS – GRUPO 2.

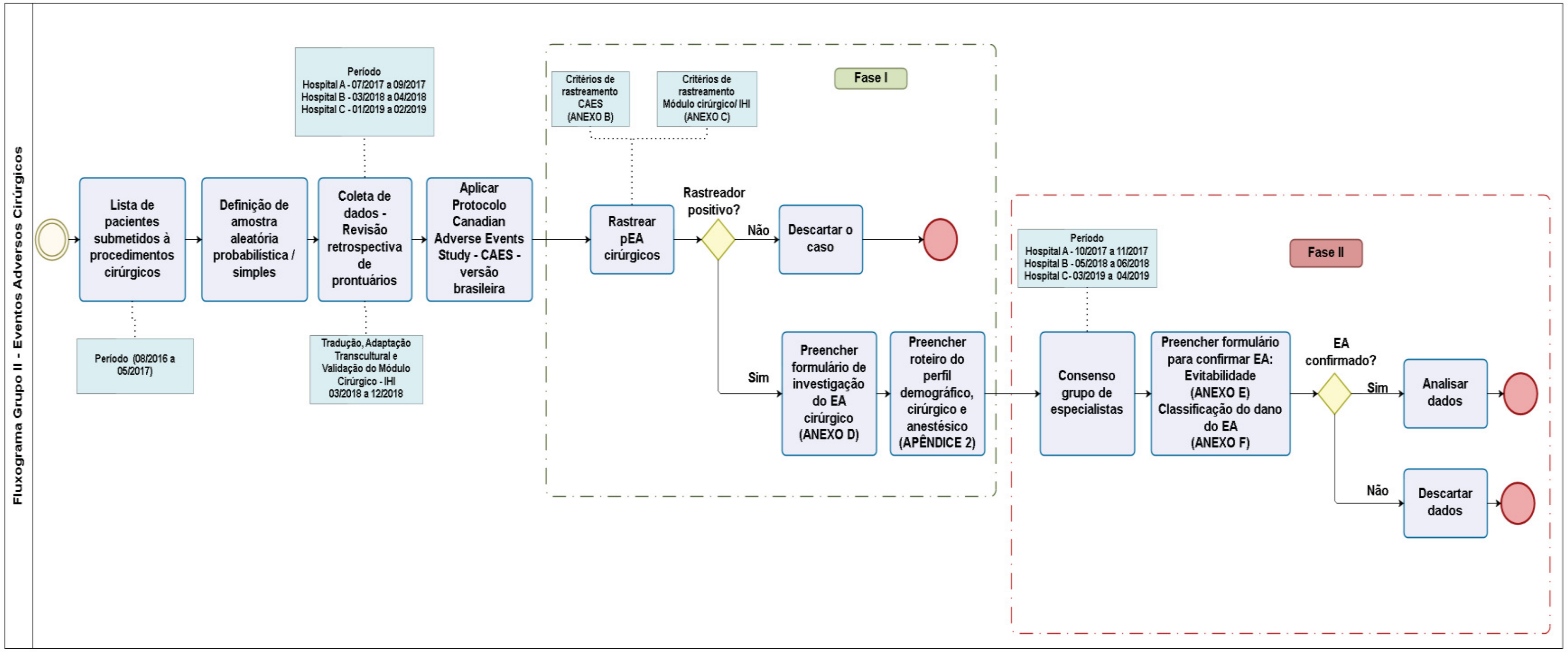

FONTE: A AUTORA (2019).

4.5 TRADUÇÃO PARA O PORTUGUÊS DO BRASIL E ADAPTAÇÃO TRANSCULTURAL DO MÓDULO CIRÚRGICO DO *GLOBAL TRIGGER TOOL DO INSTITUTE OF HEALTHCARE IMPROVEMENT*.

4.5.1 Tipo de pesquisa

Pesquisa metodológica para realização da tradução e adaptação transcultural do módulo cirúrgico do *Global Trigger Tool* (GTT) do *INSTITUTE OF HEALTHCARE IMPROVEMENT (IHI)* dos Estados Unidos (ANEXO C) Este é composto por 11 gatilhos que são identificados pela letra "S" (S1 a S11), com a finalidade de investigar os critérios de rastreamento para potenciais EA cirúrgicos, durante revisão retrospectiva de amostra aleatória de prontuários (GRIFFIN; RESAR, 2009).

Para realização do processo de tradução e adaptação transcultural foi utilizada a diretriz proposta por Beaton e colaboradores (2000), por ser a mais utilizada no Brasil e no mundo (TOMASCHEWSKI-BARLEM; LERCH, DEVOS et al., 2015; SCHARDOSIM RUSCHEL; CUNHA et al., 2014; FREITAS; ANTUNES; LOPES et al., 2014). Foram realizadas as seguintes etapas: 1. Tradução inicial, 2. Síntese das Traduções, 3. Retrotradução, 4. Comitê de especialistas, 5. Pré-teste, 6. Avaliação pelos autores do instrumento (BEATON; BOMBARDIER; GUILLEMIN et al., 2000). Deste modo, foi realizada a validação de conteúdo e adaptação transcultural por meio da técnica Delphi *online*.

A pesquisa metodológica refere-se às investigações dos métodos de obtenção, organização e análise de dados, discorrendo sobre a elaboração, validação e avaliação dos instrumentos e técnicas de pesquisa, objetivando a elaboração de instrumento confiável, preciso e utilizável que possa ser empregado por outros pesquisadores (POLIT; HUNGLER, 2004). É caracterizada pelos processos de testagem de instrumentos de coleta de dados e estabelece subsídios para construção e obtenção de instrumentos válidos, que sejam capazes de mensurar o que se espera por meio de procedimentos relacionados à elaboração, validação e avaliação de instrumentos de mensuração (PASQUALI, 2009). Consiste em processo dinâmico realizado a partir do julgamento de peritos para sua adequação, sendo desenvolvido com rigor metodológico, em passos previamente definidos (MATA; CARVALHO; NAPOLEÃO et al., 2011; DAL SASSO; SOUZA, 2011).

A técnica Delphi é metodologia sistematizada que possibilita o julgamento de informações, para obter consenso sobre determinado tema. Ocorre, por meio de validações articuladas em fases e ciclos, com feedback controlado das opiniões. (CASTRO; REZENDE, 2009). A técnica Delphi *online* é a adaptação do método Delphi Clássico em que são conservadas as mesmas características como o anonimato, o *feedback* e a representação estatística, porém, é realizada via *internet* de forma assíncrona. Os resultados são apresentados pela rede de computadores para que possam ser avaliados pelos pesquisadores, com exclusão da necessidade de utilização dos correios para envio dos questionários e materiais informativos (GIOVINAZZO, 2001).

4.5.2 Local e período de realização

Antes de iniciar o processo de tradução, adaptação transcultural e validação do módulo cirúrgico do GTT, foi solicitada e obtida autorização ao IHI (APÊNDICE 3).

Esta etapa da pesquisa foi desenvolvida no período de março a dezembro de 2018. As etapas para tradução inicial, síntese das traduções e retrotradução ocorreram via *online* e nas dependências do Campus Jardim Botânico, Bloco Didático II da Universidade Federal do Paraná. A etapa de validação de conteúdo e transcultural foi realizada via *online,* no período de outubro a dezembro de 2018. A etapa de pré-teste do instrumento foi realizada no Hospital C participante da pesquisa, nos meses de janeiro e fevereiro de 2019, durante as avaliações retrospectivas de prontuários em busca da ocorrência de pEA cirúrgicos.

4.5.3 Participantes

A seguir estão descritos os participantes e formas de recrutamento, conforme cada etapa proposta por Beaton e colaboradores (2000):

Etapa 1 - Tradução inicial: nesta primeira etapa ocorreram as traduções do instrumento original em inglês para o português do Brasil por dois tradutores nativos brasileiros e bilíngues (inglês e português). Um dos tradutores era da área da saúde,

conhecedor dos conceitos e do tema abordado pelo instrumento e o outro tradutor não era da área da saúde e não recebeu informações sobre o instrumento. O recrutamento dos tradutores ocorreu por meio de carta convite enviada via correio eletrônico e envio de cópia do módulo cirúrgico do GTT desenvolvido pelo IHI, na versão original a ser traduzido (ANEXO C). Depois do aceite de participação, um novo *e-mail* com informações detalhadas sobre o projeto, metodologia e demais recomendações foram enviadas aos participantes.

Etapa 2 - Síntese das Traduções: essa etapa foi realizada por meio de consenso entre os tradutores da etapa 1 e os pesquisadores para formular a redação apropriada e resolver discrepâncias observadas.

Etapa 3 - Retrotradução: fizeram parte desta etapa dois tradutores independentes bilíngues (inglês e português) que não participaram da etapa da tradução, não tiveram acesso à versão original do instrumento e não foram informados sobre os conceitos explorados. O recrutamento dos retrotradutores ocorreu por intermédio de carta convite enviada via e-mail e a versão síntese V3 elaborada na fase anterior.

Etapa 4 - Comitê de Especialistas: os profissionais foram selecionados por meio de consulta ao currículo na Plataforma Lattes do Conselho Nacional de Desenvolvimento Científico e Tecnológico, conforme os seguintes critérios: ter conhecimentos na área de saúde, metodologia, linguística e conhecer os dois idiomas envolvidos. (BEATON; BOMBARDIER; GUILLEMIN et al., 2000).

Dos seis profissionais integrantes do comitê de especialistas, quatro eram enfermeiros, uma médica e uma professora bilíngue formada em Letras. Em relação à última titulação dos especialistas, dois eram professores em enfermagem com título de doutor e especialistas em metodologia científica, segurança do paciente, tradução e adaptação de instrumentos; dois eram professores com título de mestrado em enfermagem e especialistas na área de segurança do paciente; uma médica especialista em gestão da qualidade e gestão de riscos em segurança do paciente e uma professora bilingue português-inglês.

Os especialistas receberam o email convite para participação da pesquisa e o Questionário para Especialista (APÊNDICE 4), que foram encaminhados por correio eletrônico, estabelecendo-se um prazo de dez dias para o retorno.

Etapa 5 Pré-teste: análise retrospectiva de prontuários de pacientes submetidos à cirurgia entre os meses de junho de 2016 a maio de 2017, no Hospital C participante da pesquisa. O critério de exclusão foi qualquer limitante à leitura dos prontuários, ou indisponibilidade destes no serviço de arquivamento ou no meio eletrônico, os quais foram substituídos pelos prontuários/registros imediatamente subsequentes da lista geral de cirurgias/procedimentos cirúrgicos.

4.5.4 Coleta de dados

As coletas de dados ocorreram conforme as seguintes etapas:

Etapa 1 - Tradução inicial: nesta etapa, cada tradutor foi responsável por traduzir o instrumento e suas respectivas orientações e produzir uma versão independente nominadas de **V1** e **V2**.

Etapa 2 - Síntese das Traduções: reunião entre os dois tradutores e as pesquisadoras para discussão e comparação; as inconsistências, ou dúvidas, foram esclarecidas e deram origem à versão síntese das traduções iniciais e nominada **V3**.

Etapa 3 - Retrotradução: nesta terceira etapa a versão síntese V3 foi retrotraduzida do português para o inglês. Cada retrotradutor elaborou uma nova versão, as quais foram nominadas **V4** e **V5**, com o objetivo de avaliar se o conteúdo da versão síntese era semelhante ao instrumento original. Realizou-se a síntese dessas retrotraduções, para formulação da versão **V6**, pelas pesquisadoras.

Etapa 4 - Comitê de Especialistas: nesta quarta etapa o comitê de especialistas realizou a validação de conteúdo do isntrumento, conforme a Técnica *Delphi on line* para elaboração da versão nominada **V7**. Para avaliar a concordância entre eles, utilizou-se o Índice de Validade de Conteúdo (IVC) e estabeleceu-se que seriam realizadas rodadas até que se obtivesse um IVC $\geq 0{,}8$.

O instrumento de coleta de dados foi nominado Questionário para Especialista (APÊNDICE 4) e foi composto por 27 questões, distribuídas em dois blocos, conforme escala *Likert* de 5 pontos. Ao final de cada item avaliado havia espaço para sugestões e observações dos especialistas.

No primeiro bloco, com 22 questões, a avaliação foi direcionada ao julgamento da versão V6 que correspondeu à síntese das traduções e retrotraduções realizadas nas etapas anteriores e que incluíram 11 triggers que compõem o módulo cirúrgico do instrumento e 11 orientações que constam no Manual do GTT do IHI. Foram avaliados fluidez, compreensão na redação dos itens traduzidos econteúdo dos *triggers* relacionados à metodologia para mensuração e identificação de EA cirúrgicos, a necessidade de adaptação, inclusão e/ou exclusão de itens.

O bloco dois do Questionário para Especialistas teve a finalidade de realizar a análise das equivalências semântica, idiomática, conceitual, cultural e de conteúdo. A equivalência semântica analisa a adaptação de palavras conforme gramática e vocabulário da língua para a qual realiza-se à adaptação; a equivalência idiomática refere-se às expressões coloquiais, informais ou gírias utilizadas no país de origem e que apresentam dificuldades de tradução. A equivalência conceitual consiste em verificar se as palavras estão adequadas ao contexto para uso em pesquisas no Brasil. A equivalência cultural ou experimental identifica se a versão expressa a experiência vivenciada no cotidiano dos serviços de saúde no contexto cultural do Brasil; e a equivalência de conteúdo avalia os itens do instrumento em relação à compreensão, clareza e redundâncias (BEATON; BOMBARDIER; GUILLEMIN et al., 2000).

Etapa 5 Pré-teste - nesta quinta etapa foi realizado o teste de campo da versão **V7** com análise retrospectiva de 244 prontuários, de pacientes submetidos à cirurgia entre os meses de junho de 2016 à maio de 2017, no Hospital C participante da pesquisa. Após a primeira fase, os pEA cirúrgicos que apresentaram critérios de rastreamento positivos foram reavaliados por dois enfermeiros especialistas em SP e uma médica especialista em gestão da qualidade e riscos em saúde, para confirmação diagnóstica da ocorrência dos EA cirúrgicos e sua caracterização.

Etapa 6 Avaliação pelos autores do instrumento: na sexta etapa, as pesquisadoras elaboraram a versão final do instrumento, nominada **V8**, a qual foi encaminhada para os autores do instrumento original para ciência, avaliação, aprovação e divulgação pelo site do IHI.

4.5.5 Procedimentos de análise de dados

Nas etapas de **Tradução e Retrotradução** foram analisadas qualitativamente as versões de consenso elaboradas em cada fase (**V3 e V6**).

Na etapa do **Comitê de especialistas**, após o recebimento das respostas dos questionários, as questões pontuadas por meio da escala *Likert* foram analisadas quantitativamente, mediante aplicação de testes psicométricos. Nas variáveis quantitativas, os resultados foram descritos por médias, medianas, valores mínimos, máximos e desvio padrão. As sugestões dos especialistas foram analisadas qualitativamente.

Para medir o grau de concordância entre os especialistas, foi aplicado o IVC. Os participantes puderam avaliar o instrumento traduzido de maneira quantitativa até que se atingisse o IVC ≥0,8. Deste modo, cada item do módulo cirúrgico GTT e suas respectivas orientações foram avaliados por meio de escala *Likert,* com variação de cinco pontos: (-2) “Discordo Totalmente”, (-1) “Discordo”, (0) “Indiferente”, (+1) “Concordo” e (+2) “Concordo Totalmente”. Após avaliar cada item, os especialistas fizeram avaliação qualitativa em campo apropriado para sugestões de alterações.

A Técnica Delphi permite o desenvolvimento de um processo estruturado, com a submissão de questionários em rodadas, para obtenção de opinião coletiva, qualificada e consensual por pessoas selecionadas (*experts*), conhecedoras potenciais de assuntos específicos (KEENEY; HASSON; MCKENNA, 2006; ESHER; SANTOS; MAGARINOS-TORRES et al., 2012). A metodologia de Likert utilizada nos questionários desta pesquisa é uma das mais usadas nos processos de investigação e preconiza escalas que permitem que pessoas com diferentes opiniões e pontos de vista respondam de forma diferenciada (LIKERT, 1932).

Estudos que utilizam a metodologia, ou escala de *Likert,* empregam instrumentos com número ímpar de classe de medida (cinco pontos) e o resultado é obtido pela soma dos valores dos itens (escore), os quais podem ser tratados estatisticamente como variáveis de tipo intervalar. O formato de medida, para ser denominado como escala Likert, tem que ser composto por número ímpar e o ponto central é um ponto neutro, cujos extremos devem ser opostos e simétricos (CURADO; TELES; MARÔCO, 2014).

O IVC mede a proporção ou porcentagem de *experts* que estão de acordo sobre determinados elementos do instrumento; com ele, é possível avaliar cada item individualmente e, posteriormente, o instrumento como um todo (ALEXANDRE; COLUCI, 2011; COLUCI; ALEXANDRE; MILANI, 2015).

O IVC foi calculado por meio da soma de concordância dos itens pelos *experts*, considerando concordante aquele que assinala (+1) “Concordo” e (+2) “Concordo Totalmente”. A fórmula para o cálculo constitui-se do número de *experts* que estavam de acordo com a questão, dividido pelo número de *experts* total participantes, conforme FIGURA 5. O valor de IVC ≥0,8 foi estabelecido baseando-se em revisão bibliográfica, a qual encontrou como níveis de consenso entre os especialistas, para este tipo de avaliação, valores entre 0,5 e 0,8 (CASTRO; RESENDE, 2009), porém, sabe-se que quanto maior o IVC, melhor a concordância (COLUCI; ALEXANDRE; MILANI, 2015).

FIGURA 5 – FÓRMULA PARA O CÁLCULO DO ÍNDICE DE VALIDADE DE CONTEÚDO

$$IVC = \frac{\text{Número de respostas } +1 \text{ ou } +2}{\text{Número total de Respostas}}$$

FONTE: Adaptado de ALEXANDRE; COLUCI (2011).

Os “Testes adicionais à versão adaptada” foram realizados por meio de análise do IVC das equivalências semântica, idiomática, de experiência e conceitual, utilizando-se a técnica Delphi *online*. (BEATON; BOMBARDIER; GUILLEMIN et al., 2000).

Para análise da consistência interna dos questionários preenchidos pelos especialistas, para validação de conteúdo e transcultural do instrumento, foi aplicado o coeficiente *Alfa* de *Cronbach* que mede a correlação entre as respostas. Estabeleceu-se, para esta pesquisa, a seguinte atribuição de confiabilidade, a qual mede a correlação entre as respostas escalonadas: Discordo Totalmente (-2), Discordo (-1), Indiferente (0), Concordo (+1) e Concordo Totalmente (+2). Foi atribuída a seguinte confiabilidade: >0,90 = excelente; a >0,80 = bom; a >0,70 = aceitável; a >0,60 = questionável; a >0,50 = pobre; a <0,50 = inaceitável. (GEORGE; MALLERY, 2003). Estabeleceu-se o valor mínimo de 0,7 por ser considerado valor aceitável na avaliação da confiabilidade.

Nas áreas de ciências sociais e da saúde, a consistência interna de um teste ou de um questionário é utilizada para verificar se as respostas são coerentes e confiáveis. Nesse sentido, a confiabilidade de um teste poderá indicar se as diferenças encontradas nas pontuações obtidas são decorrentes de variações reais ou apenas de erros casuais. Ela também se refere à estabilidade dos resultados de um teste, ou seja, ao grau de consistência e precisão dos escores (CRONBACH, 1951).

A confiabilidade é também denominada fidedignidade e se refere à consistência para medir determinado atributo e produzir resultados semelhantes em testagens repetidas. É necessário analisar a medição deste atributo, por intermédio da consistência interna, a fim de avaliar se os itens do instrumento medem o mesmo conceito ou característica (PASQUALI, 2009).

O coeficiente Alfa *de Cronbach* é considerado um dos principais parâmetros de avaliação de confiabilidade de instrumentos de medição, e largamente utilizado em diversas áreas do conhecimento, principalmente na área da saúde e enfermagem (MATTHIENSEN, 2011).

O FLUXOGRAMA 3 representa a metodologia utilizada para tradução e adaptação transcultural para o português do Brasil do módulo cirúrgico do GTT do IHI.

FLUXOGRAMA 3 – ETAPAS METODOLÓGICAS DO PROCESSO DE TRADUÇÃO, ADAPTAÇÃO TRANSCULTURAL E VALIDAÇÃO DE CONTEÚDO DO MÓDULO CIRÚRGICO DO *GLOBAL TRIGGER TOOL* PARA USO NO BRASIL.

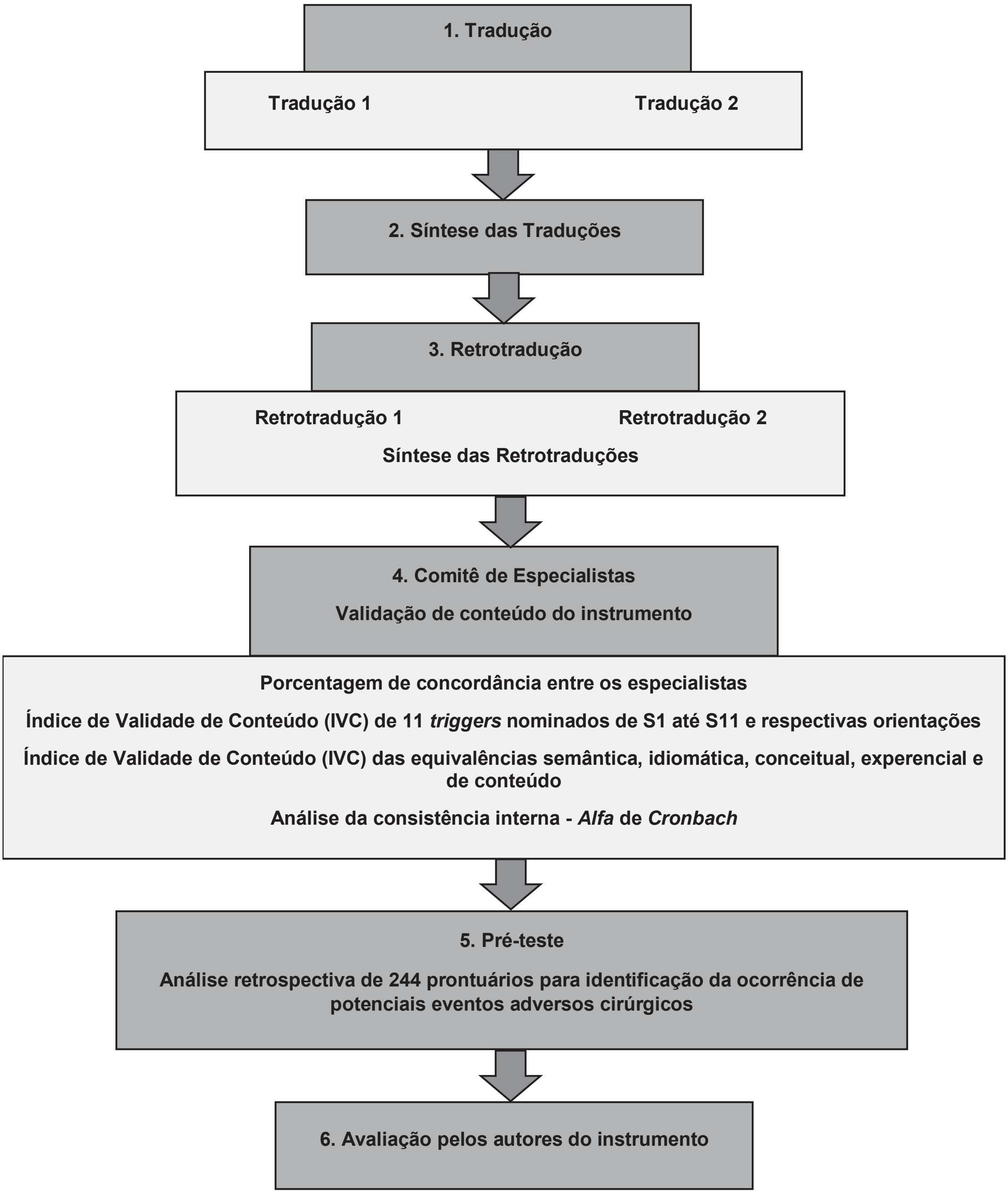

FONTE: A autora (2018).

4.6 ASPECTOS ÉTICOS

A pesquisa foi aprovada pelo Comitê de Ética e Pesquisa (CEP) do Hospital A sob o parecer número 1.990.760 (ANEXO H), recebeu anuência do Hospital B sob parecer número 2.325.448 (ANEXO I), e do Hospital C sob parecer número 2.367.592 (ANEXO J).

A etapa da pesquisa destinada à tradução, adaptação transcultural e validação do módulo cirúrgico *Global Trigger Tool* para uso no Brasil ocorreu após a autorização formal do *Institute of Healthcare Improvement* (ANEXO G), que elaborou e publicou o instrumento, com a condição de que lhe fosse atribuído o desenvolvimento do conteúdo original, seguido da informação de que foi adaptado, traduzido e validado para o português brasileiro, com a inclusão da seguinte linha de crédito no material: Fonte: *Adapted from the Institute for Healthcare Improvement Global Trigger Tool for Measuring Adverse Events*, seguido do endereço online do instrumento original.

O IHI solicitou o encaminhamento de carta sobre a finalização do processo de tradução, adaptação transcultural e validação do módulo cirúrgico GTT (APÊNDICE 5) e da versão final elaborada nesta pesquisa, para ser publicada no site da instituição, com objetivo de ser compartilhada com outros pesquisadores e profissionais de saúde interessados na pesquisa de EA cirúrgicos.

Todos os participantes foram informados dos objetivos e metodologia da pesquisa, observando o preconizado pela Resolução nº 466/2012 do Conselho Nacional de Pesquisa (BRASIL, 2012). Os participantes convidados que aceitaram participar da pesquisa assinaram o Termo de Consentimento Livre e Esclarecido - TCLE (APÊNDICE 1). Os dados serão utilizados apenas para fins científicos, garantindo o anonimato e confidencialidade das respostas individuais dos participantes e dos pacientes cujos prontuários foram investigados.

5 RESULTADOS

5.1 CULTURA DE SEGURANÇA

5.1.1 Características sóciodemográficas e laborais dos participantes

Os participantes do estudo foram 381 profissionais sendo que destes, 63,7% (n=158) foram do Hospital A; 59% (n=121) do Hospital B e 61% (n=102) do Hospital C. A TABELA 1 apresenta a distribuição dos profissionais segundo o perfil sociodemográfico e laboral.

TABELA 1 – DISTRIBUIÇÃO DAS VARIÁVEIS SÓCIODEMOGRÁFICAS E LABORAIS DOS PROFISSIONAIS DE SAÚDE. CURITIBA–PARANÁ, 2017-2018

(continua)

Variáveis	Hospital A		Hospital B		Hospital C	
	n	%	n	%	n	%
**Sexo**						
Feminino	91	57,6	67	55,4	59	57,8
Masculino	67	42,4	54	44,6	43	42,2
**Categoria profissional**						
Médico	41	25,9	23	19,0	15	14,7
Médico residente	31	19,6	57	47,1	37	36,3
Enfermeiro	15	9,5	15	12,4	15	14,7
Técnico de Enfermagem	24	15,2	18	14,9	25	24,5
Auxiliar de Enfermagem	47	29,7	08	6,6	10	9,8
**Grau de instrução**						
Ensino médio completo	26	16,4	25	20,7	34	33,3
Ensino superior incompleto	12	7,7	-	-	-	-
Ensino superior completo	35	22,2	32	26,4	23	22,5
Pós-graduação (nível especialização)	59	37,3	55	45,5	37	36,3
Pós-graduação (nível mestrado ou doutorado)	25	15,8	08	6,6	07	6,9
**Tempo de atuação no hospital (em anos)**						
Menos de 1	22	13,9	15	12,4	14	13,7
1-5	43	27,2	81	66,9	58	56,9
6-10	06	3,8	19	15,7	25	24,5
11-15	23	14,6	-	-	-	-
16-20	10	6,3	-	-	-	-
21 ou mais	54	34,2	06	5,0	05	4,9
**Tempo de atuação na unidade (em anos)**						
Menos de 1	31	19,6	21	17,4	24	23,5

TABELA 1 – DISTRIBUIÇÃO DAS VARIÁVEIS SÓCIODEMOGRÁFICAS E LABORAIS DOS PROFISSIONAIS DE SAÚDE. CURITIBA–PARANÁ, 2017-2018

(conclusão)

1-5	55	34,8	78	64,5	54	52,9
6-10	06	3,8	17	14,0	20	19,6
11-15	15	9,5	-	-	-	-
16-20	11	7,0	-	-	-	-
21 ou mais	40	25,3	05	4,1	04	3,9
**Carga horária semanal**						
20-39 horas	111	70,2	53	43,8	50	49,0
40-59 horas	17	10,8	04	3,3	15	14,7
60-79 horas	17	10,8	34	28,1	22	21,6
80-99 horas	07	4,4	26	21,5	10	9,8
100 horas ou mais	06	3,8	04	3,3	05	4,9
**Interação/Contato direto com o paciente**						
Sim	158	100	120	99,2	101	99,0
Não	-	-	01	0,8	01	1,0
**Total**	**158**	**100**	**121**	**100**	**102**	**100**

FONTE: A autora (2019).

Houve predominância do sexo feminino nos três locais de estudo e a média de idade dos participantes foi de 43 anos no hospital A (desvio padrão -DP±12,3), 32,2 no hospital B (DP±8,2) e 35 no hospital C (DP±9,7), com idade mínima de 22 anos e máxima de 71 anos. A média de atuação profissional (em anos) no Hospital A foi de 18 anos, no Hospital B de 4,7 anos e no Hospital C de 5,7 anos.

Em relação ao tempo de trabalho dos profissionais em cada hospital, verificou-se predominância da variável 21 anos ou mais no Hospital A (34,2%) e de 1-5 anos no Hospital B (66,9%) e Hospital C (56,9%). Quanto ao contato direto com o paciente, as características foram similares nas três instituições, 100% dos respondentes no hospital A e 99% nos hospitais B e C. Os dois respondentes que não tinham contato direto com o paciente tinham função de gerência.

5.1.2 Dimensões e variáveis de resultado da cultura de segurança do paciente nos hospitais participantes

Para cada uma das dimensões avaliadas, testou-se a hipótese nula de que as distribuições sobre as classificações de respostas (negativa, neutra e positiva) são iguais para os três hospitais, *versus* a hipótese alternativa de distribuições diferentes.

Na TABELA 2 são apresentados, para cada dimensão, os percentuais de respostas negativas, neutras e positivas para os três hospitais e os valores de p.

TABELA 2 – DISTRIBUIÇÃO DAS FREQUÊNCIAS DAS RESPOSTAS POR GRUPO PROFISSIONAL EM RELAÇÃO ÀS DIMENSÕES DA CULTURA DE SEGURANÇA ORGANIZACIONAL. CURITIBA–PARANÁ, 2017-2018

Dimensão	Hospital	Pecentual de Respostas			Valor de p*
		**Resposta Negativa**	**Resposta Neutra**	**Resposta Positiva**	
1. Trabalho em equipe dentro das unidades	Hospital A	23,4	20,6	56	**<0,001**
	Hospital B	6,6	15,5	77,8	
	Hospital C	13,8	20,4	65,8	
2. Expectativas sobre seu supervisor/chefe e ações promotoras de segurança	Hospital A	24,8	18,8	56,4	**<0,001**
	Hospital B	12,7	15,6	71,8	
	Hospital C	19,4	11	69,6	
3. Aprendizado organizacional-melhoria continua	Hospital A	20,3	20,3	59,5	
	Hospital B	8,6	18,8	72,6	
	Hospital C	14,1	17,8	68,1	
4. Apoio da gestão para segurança do paciente	Hospital A	36,8	32,5	30,8	**<0,001**
	Hospital B	32,6	18,9	48,5	
	Hospital C	30,5	27,5	42,0	
5. Percepção geral da segurança do paciente	Hospital A	35,4	18,9	45,8	**<0,001**
	Hospital B	56,6	15,3	28,1	
	Hospital C	45,1	18,1	36,8	
6. Retorno da informação e comunicação sobre o erro	Hospital A	30,4	31,8	37,8	0,010
	Hospital B	33,1	27,3	39,5	
	Hospital C	22,0	30,6	47,4	
7. Abertura da comunicação	Hospital A	26,1	23,1	50,8	
	Hospital B	17,9	24,8	57,3	
	Hospital C	20,6	29,1	50,3	
8. Frequência de relatos de eventos	Hospital A	31,9	22,8	45,3	0,055
	Hospital B	28,4	29,2	42,4	
	Hospital C	27,1	21,8	51,2	
9. Trabalho em equipe entre as unidades	Hospital A	42,4	26,8	30,7	**<0,001**
	Hospital B	23,9	18,4	57,7	
	Hospital C	34,3	20,7	44,9	
10. Adequação de profissionais	Hospital A	40,2	20,6	39,2	0,227
	Hospital B	41,0	23,9	35,1	
	Hospital C	45,0	22,1	32,9	
11. Passagem de plantão/turnos e transferências	Hospital A	41,5	26	32,5	**<0,001**
	Hospital B	30,9	21,9	47,2	
	Hospital C	39,0	22,5	38,5	
12. Respostas não punitivas ao erro	Hospital A	64,5	16,3	19,3	0,029
	Hospital B	75,1	11,6	13,3	
	Hospital C	68,5	14,8	16,7	

FONTE: A autora (2018).
*Teste de Qui-quadrado, p<0,05

Conforme apresentados na TABELA 2, verifica-se que a dimensão **Trabalho em equipe dentro das unidades** foi a única identificada como fortalecida no Hospital B (77,8%). Os profissionais deste hospital externaram, significativamente, percepção menos negativa em seis dimensões de cultura ($p<0,001$). Nos demais hospitais, nenhuma outra dimensão mostrou cultura fortalecida, ou seja, com valores equivalentes a ≥75%.

De modo geral, as dimensões mais próximas de escores positivos foram: **Expectativas sobre seu supervisor/chefe e ações promotoras de segurança**, com 56,4% no Hospital A, 71,8% no Hospital B e 69,6% no Hospital C; **Aprendizado organizacional - melhoria contínua**, com 59,5% no Hospital A, 72,6% no Hospital B e 68,1% no Hospital C.

Entre os escores negativos, o maior percentual foi identificado na dimensão **Respostas não punitivas ao erro**, com 64,5% no Hospital A, 75,1% no Hospital B e 68,5% no Hospital C. As dimensões **Apoio da gestão para a segurança do paciente**, **Percepção geral da segurança do paciente**, **Retorno da informação e comunicação sobre o erro**, **Adequação de profissionais**, **Passagem de plantão/turnos e transferências** apresentaram-se fragilizadas (≤50%) nos três hospitais.

A TABELA 3 demonstra que o grupo profissional da enfermagem, comparado aos profissionais médicos, apresentou percepção mais positiva nos três hospitais para as 10 dimensões: Trabalho em equipe dentro das unidades, Expectativas sobre seu supervisor/chefe e ações promotoras de segurança, Aprendizado organizacional- melhoria contínua, Apoio da gestão para a segurança do paciente, Retorno da informação e comunicação sobre o erro, Abertura da comunicação, Frequência de relatos de eventos, Trabalho em equipe entre as unidades, Adequação de profissionais e Passagem de plantão/turnos e transferências.

Na dimensão **Percepção geral da segurança do paciente**, a comparação entre os profissionais revelou que os médicos residentes apresentam percepção mais positiva (42,7%).

Na dimensão **Respostas não punitivas ao erro** foram identificados baixos percentuais de respostas positivas em todas as categorias profissionais, nos três hospitais.

TABELA 3 – DIMENSÕES DE CULTURA DE SEGURANÇA E O PERCENTUAL DE RESPOSTA POSITIVA POR GRUPO PROFISSIONAL. CURITIBA–PARANÁ, 2017-2018

(continua)

**Dimensão**	**Profissional**	**Hospital A**	**Hospital B**	**Hospital C**
Trabalho em equipe dentro das unidades	Médicos Assistentes	59,8	74,7	64,4
	Médicos Residentes	43,9	73,7	64,9
	Enfermagem	58,5	85,4	67
Expectativas sobre seu supervisor/chefe e ações promotoras de segurança	Médicos Assistentes	53,0	64,1	56,7
	Médicos Residentes	56,5	71,7	66,9
	Enfermagem	58,0	76,2	75,5
Aprendizado organizacional-melhoria contínua	Médicos Assistentes	51,2	69,1	65,9
	Médicos Residentes	60,9	70,0	61,8
	Enfermagem	63,0	78,0	73,3
Apoio da gestão para a segurança do paciente	Médicos Assistentes	23,6	44,9	37,8
	Médicos Residentes	25,8	43,1	32,7
	Enfermagem	36,1	57,7	50,0
Percepção geral da segurança do paciente	Médicos Assistentes	41,5	40,2	36,7
	Médicos Residentes	42,7	18	29,1
	Enfermagem	49,0	35,4	42,5
Retorno da informação e comunicação sobre o erro	Médicos Assistentes	26,8	35,3	40,9
	Médicos Residentes	31,5	28,1	36,9
	Enfermagem	45,3	57,7	57,0
Abertura da comunicação	Médicos Assistentes	55,7	60,9	53,3
	Médicos Residentes	36,6	45,0	36,0
	Enfermagem	53,7	72,4	60,0
Frequência de relatos de eventos	Médicos Assistentes	35,0	27,5	31,1
	Médicos Residentes	29,0	26,3	32,4
	Enfermagem	56,3	73,2	71,4
Trabalho em equipe entre as unidades	Médicos Assistentes	25,0	44,6	40,0
	Médicos Residentes	17,1	52,5	36,1
	Enfermagem	38,5	72,2	53,0

TABELA 3 – DIMENSÕES DE CULTURA DE SEGURANÇA E O PERCENTUAL DE RESPOSTA POSITIVA POR GRUPO PROFISSIONAL. CURITIBA–PARANÁ, 2017-2018

(conclusão)

Adequação de profissionais	Médicos Assistentes	37,8	33,7	28,3
	Médicos Residentes	39,0	24,8	26,5
	Enfermagem	39,9	50,3	39,0
Passagem de plantão/turnos e transferências	Médicos Assistentes	22,1	40,2	35,0
	Médicos Residentes	20,7	40,8	25,0
	Enfermagem	41,8	59,8	49,5
Respostas não punitivas ao erro	Médicos Assistentes	13,0	17,4	17,8
	Médicos Residentes	15,1	6,4	9,0
	Enfermagem	23,9	20,7	22,1

FONTE: A autora (2018).

A dimensão **Respostas não punitivas ao erro** é composta por três perguntas denominadas A8 – “os profissionais consideram que seus erros, enganos ou falhas podem ser usados contra eles”; A12 – “quando um evento é relatado, parece que o foco recai sobre a pessoa e não sobre o problema” e A 16 – “os profissionais se preocupam que seus erros, enganos ou falhas sejam registrados em suas fichas funcionais”. Estas três perguntas (A8, A12 e A16) fazem parte das questões formuladas negativamente e que precisam de escores reversos no cálculo, ou seja, quando os profissionais discordam estão respondendo positivamente (escores positivos) para a dimensão analisada. Deste modo, a TABELA 4 apresenta a frequência das respostas dos profissionais e escores de cada pergunta que compõem esta dimensão.

TABELA 4 – ESCORES DA DIMENSÃO DAS RESPOSTAS NÃO PUNITIVAS AO ERRO. CURITIBA–PARANÁ, 2017-2018

PERGUNTAS	ESCORE	Hospital A		Hospital B		Hospital C	
		n	%	n	%	n	%
A8 - Os profissionais consideram que seus erros, enganos ou falhas podem ser usados contra eles?	Negativo	115	74,2	101	64,7	85	54,4
	Neutro	20	12,9	23	14,7	33	21,2
	Positivo	20	12,9	32	20,5	38	24,4
A12 - Quando um evento é relatado, parece que o foco recai sobre a pessoa e não sobre o problema?	Negativo	95	78,5	92	76,0	84	70,6
	Neutro	17	14,0	8	6,6	17	14,3
	Positivo	9	7,4	21	17,4	18	15,1
A16 - Os profissionais se preocupam que seus erros, enganos ou falhas sejam registrados em suas fichas funcionais?	Negativo	79	77,5	64	62,7	66	65,3
	Neutro	14	13,7	14	13,7	17	16,8
	Positivo	9	8,8	24	23,5	18	17,8

FONTE: A autora (2018).

Foram identificados baixos percentuais de escores positivos em todas as questões (A8, A12 e A 16), o que demonstra que os profissionais discordam que nos três Hospitais existe cultura para respostas não punitivas ao erro, ou seja, concordam que há cultura punitiva ao erro.

Deste modo, na dimensão **Respostas não punitivas ao erro**, as maiores porcentagens concentraram-se nos escores negativos, conforme taxas evidenciadas nos três hospitais:

- Na questão A8, os escores negativos mostram que a maioria dos profissionais concordam que seus erros, enganos ou falhas podem ser usados contra eles (74,2%; 64,7% e 54,4%), respectivamente, nos Hospitais A, B e C.

- Na questão A12, os escores negativos evidenciam que a maioria dos profissionais concordam que quando um evento é relatado, parece que o foco recai sobre a pessoa e não sobre o problema (78,5%; 76,0% e 70,6%), respectivamente, nos Hospitais A, B e C.

- Na questão A 16, “os os escores negativos demonstram que os profissionais concordam e se preocupam que seus erros, enganos ou falhas sejam registrados em suas fichas funcionais (77,5%; 62,7% e 65,3%), respectivamente, nos Hospitais A, B e C.

O GRÁFICO 1 evidencia a frequência dos escores positivos, neutros e negativos na dimensão **Respostas não punitivas ao erro,** em cada hospital participante.

A análise dos resultados mostra baixos percentuais de escores positivos, o que indica que os profissionais discordam (escores reversos) que nos três hospitais existe cultura de respostas não punitivas ao erro, e demonstra fragilidade nesta dimensão, com escores positivos ≤50%.

GRÁFICO 1 – FREQUÊNCIA DAS AVALIAÇÕES NA DIMENSÃO RESPOSTAS NÃO PUNITIVAS AO ERRO.

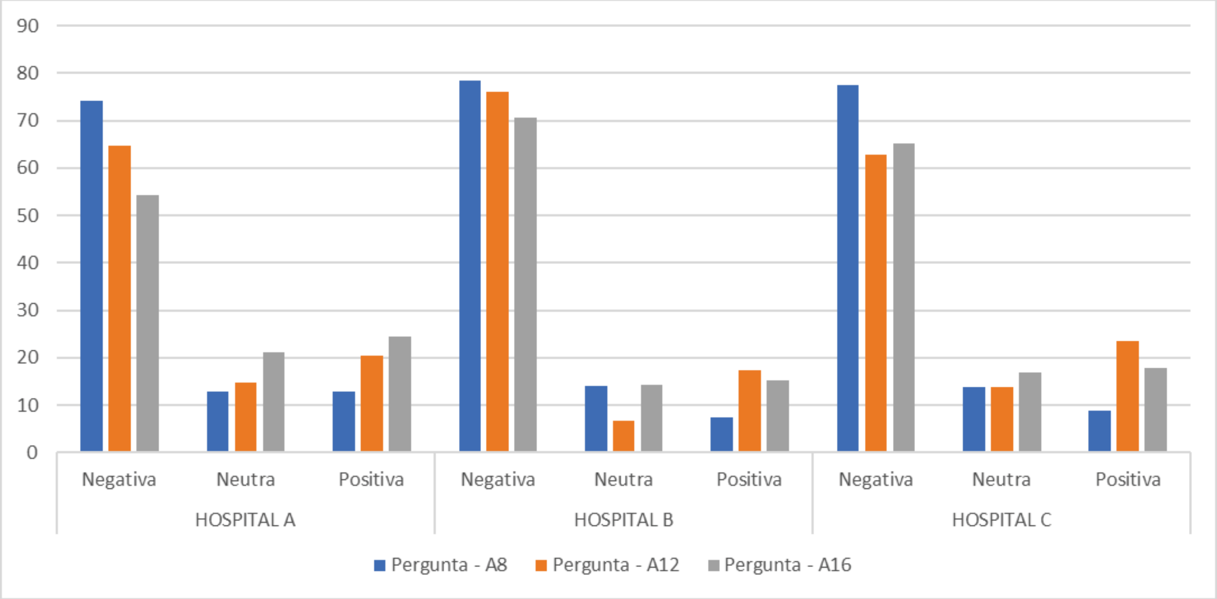

FONTE: A autora (2018).
LEGENDA: A8 - Os profissionais consideram que seus erros, enganos ou falhas podem ser usados contra eles? A12 - Quando um evento é relatado, parece que o foco recai sobre a pessoa e não sobre o problema?
A16 - Os profissionais se preocupam que seus erros, enganos ou falhas sejam registrados em suas fichas funcionais?

5.1.3 Associação dos hospitais em relação à nota de segurança e número de eventos notificados.

A TABELA 5 apresenta a associação da nota de segurança do paciente com as categorias profissionais e respectivos hospitais.

No Hospital A verifica-se que maior porcentagem de respostas foi expressa na categoria enfermagem, que atribuíram nota excelente/muito boa (70,9%). No Hospital B, a maior porcentagem de respostas foi verificada na categoria médicos residentes, com nota geral regular (64,9%). No Hospital C, a maior porcentagem de respostas foi atribuída pelos médicos assistentes com nota excelente/ muito boa (66,7%).

TABELA 5 – ASSOCIAÇÃO DA NOTA DE SEGURANÇA DO PACIENTE NOS HOSPITAIS E CATEGORIAS PROFISSIONAIS. CURITIBA–PARANÁ, 2017-2018

**Categoria profissional**	**Nota de segurança do paciente**	**Hospital A**		**Hospital B**		**Hospital C**		**Valor p**
		n	%	n	%	n	%	
**Médico Assistente**	Excelente/muito boa	24	58,5	13	56,5	10	66,7	0,3
	Regular	15	36,6	7	30,4	2	13,3	
	Ruim/muito ruim	2	4,9	3	13,0	3	20,0	
**Médico Residente**	Excelente/muito boa	18	58,1	19	33,0	16	43,2	Não aplicável
	Regular	13	41,9	37	64,9	19	51,4	
	Ruim/muito ruim	0	0,00	1	1,8	2	5,4	
**Enfermagem**	Excelente/muito boa	61	70,9	23	56,1	23	46,0	0,028
	Regular	22	25,6	18	43,9	25	50,0	
	Ruim/ muito ruim	3	3,5	0	0,0	2	4,0	

FONTE: A autora (2018).

Em relação à categoria enfermagem, o Hospital A apresentou o maior escore para a nota em relação à segurança do paciente, como excelente/muito boa (70,9%). No Hospital B, mais da metade da equipe de enfermagem também expressou nota excelente/muito boa (56,1%). Porém, no Hospital C, a maioria desta categoria profissional atribuiu nota regular para à segurança (50,0%).

A análise da categoria médicos assistentes demonstra que mais da metade dos profissionais atribuíram nota excelente/muito nos três hospitais, com maior destaque no Hospital C (66,7%). Os resultados mostram que na categoria médicos residentes, no Hospital A prevaleceu a nota excelente/muito boa (58,1%) para à segurança do paciente; diferente da avaliação realizada nos Hospitais B e C, que teve como prevalente a nota regular (64,9%; 51,4%).

A seguir, o GRÁFICO 2 apresenta os valores da nota geral de segurança do paciente nos três hospitais.

GRÁFICO 2 – NOTA GERAL DE SEGURANÇA DO PACIENTE.

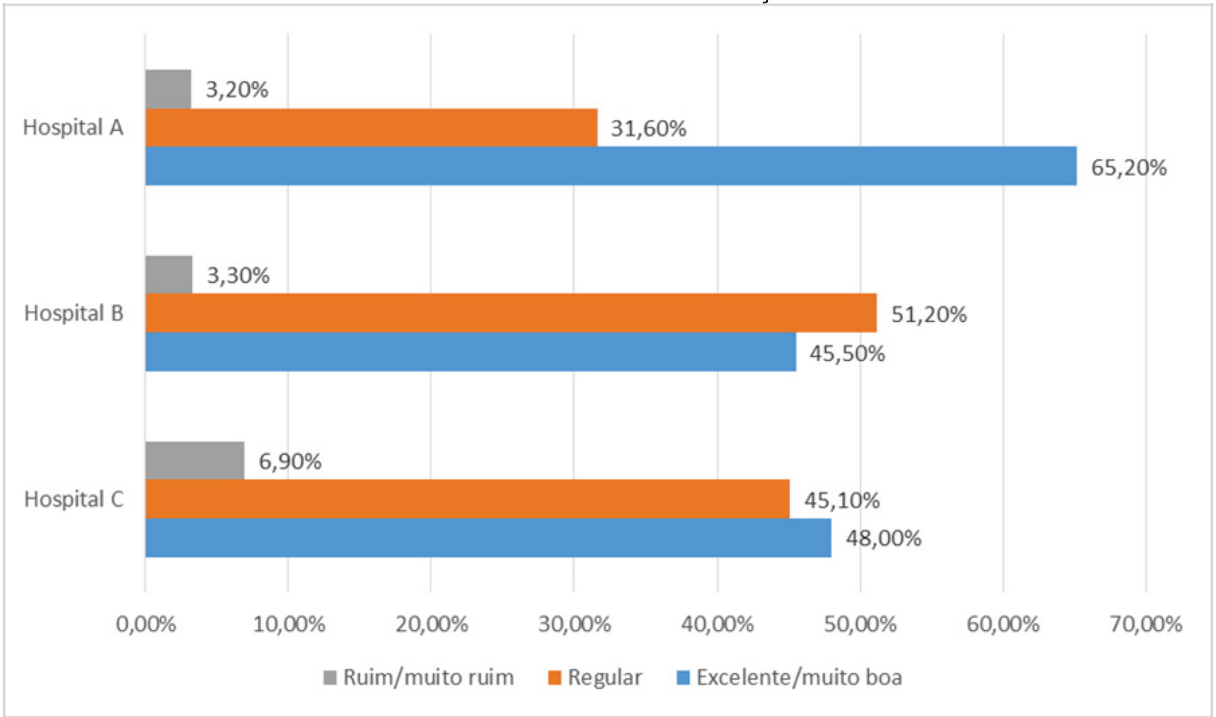

FONTE: A autora (2018).

A TABELA 6 demonstra a associação do número de eventos notificados nos últimos 12 meses com as categorias profissionais e respectivos hospitais.

TABELA 6 – ASSOCIAÇÃO DO NÚMERO DE EVENTOS NOTIFICADOS NOS TRÊS HOSPITAIS POR GRUPO PROFISSIONAL. CURITIBA–PARANÁ, 2017-2018

Categoria profissional	Número de eventos notificados	Hospital A		Hospital B		Hospital C		Valor p
		n	%	n	%	n	%	
**Médico Assistente**	Nenhum	17	41,5%	11	47,8%	8	53,3%	Não aplicável
	1-2	18	43,9%	10	43,5%	5	33,3%	
	3-5	5	12,2%	2	8,7%	2	13,3%	
	6 ou mais	1	2,4%	0	0,0%	0	0,0%	
**Médico Residente**	Nenhum	14	45,2%	36	63,2%	20	54,1%	Não aplicável
	1-2	11	35,5%	21	36,8%	17	45,9%	
	3-5	5	16,1%	0	0,0%	0	0,0%	
	6 ou mais	1	3,2%	0	0,0%	0	0,0%	
**Enfermagem**	Nenhum	55	64,7%	25	61,0%	30	61,2%	0,201
	1-2	13	15,3%	10	24,4%	12	24,5%	
	3-5	10	11,8%	6	14,6%	2	4,1%	
	6 ou mais	7	8,2%	0	0,0%	5	10,2%	

FONTE: A autora (2018).

De modo geral, destaca-se a prevalência de alta frequência para à ausência de notificações, em todas as categorias profissionais. Apenas 24% dos participantes fizeram 6 ou mais notificações.

Nas categorias profissionais de médicos residentes e enfermagem, as maiores porcentagens foram encontradas nas respostas que consideraram nenhum evento relatado nos últimos 12 meses.

Quanto à categoria de médicos assistentes, os maiores escores foram para 1-2 notificações de eventos (43,90%) nos últimos 12 meses, verificado no Hospital A, porém, não houve significância estatística. Nos demais hospitais, na avaliação dos médicos assistentes, prevaleceu a classificação de nenhum evento relatado (47,80% Hospital B; 53,30% Hospital C).

O GRÁFICO 3 mostra o número de eventos notificados nos hospitais nos últimos 12 meses, de acordo com a avaliação dos participantes.

GRÁFICO 3 – NÚMERO DE EVENTOS NOTIFICADOS NOS TRÊS HOSPITAIS.

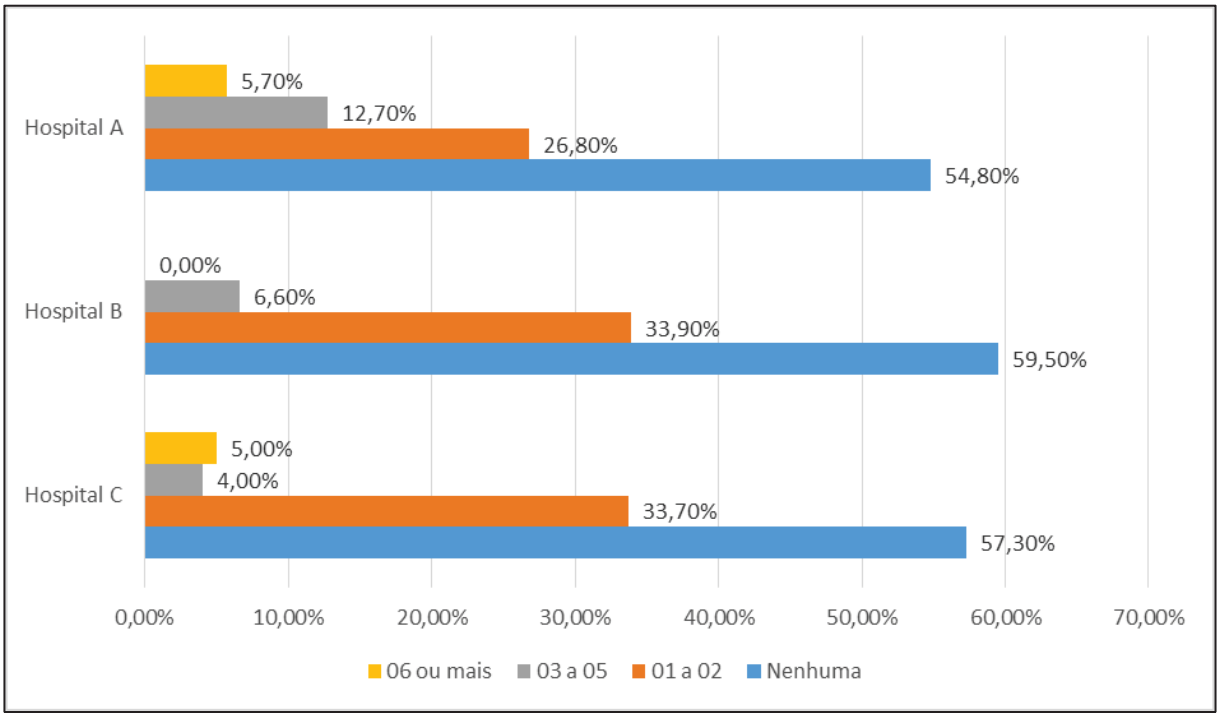

FONTE: A autora (2018).

O Gráfico 3 demonstra que, em relação ao número de EA notificados, os três hospitais apresentam predomínio de nenhum evento; seis ou mais eventos foram notificados no Hospital A (5,7%) e no Hospital C (5,0%) , mas no Hospital B, nenhum participante notificou seis ou mais eventos.

5.1.4 Propriedades psicométricas do instrumento de pesquisa

As perguntas que compõem cada dimensão do questionário foram submetidas à análise de confiabilidade, pelo cálculo do alfa de *Cronbach,* conforme demonstra a TABELA 7.

TABELA 7 – DIMENSÕES DA CULTURA DE SEGURANÇA ORGANIZACIONAL E OS VALORES DO COEFICIENTE *ALFA DE CRONBACH*. CURITIBA–PARANÁ, 2017-2018

**Dimensão***	**Coeficiente Alfa de *Cronbach*†**		
	**Hospital A**	**Hospital B**	**Hospital C**
1. Trabalho em equipe dentro das unidades	0,75	0,69	0,66
2. Expectativas sobre seu supervisor/chefe e ações promotoras de segurança	0,70	0,57	0,65
3. Aprendizado organizacional- melhoria contínua	0,59	0,50	0,56
4. Apoio da gestão para a segurança do paciente	0,70	0,50	0,62
5. Percepção geral da segurança do paciente	0,50	0,69	0,53
6. Retorno da informação e comunicação sobre o erro	0,76	0,76	0,58
7. Abertura de comunicação	0,55	0,58	0,67
8. Frequência de relato de eventos	0,82	0,89	0,86
9. Trabalho em equipe entre as unidades	0,74	0,74	0,66
10. Adequação de profissionais	0,26	0,47	0,46
11. Passagem de plantão/turnos e transferências	0,74	0,74	0,74
12. Respostas não punitivas ao erro	0,10	0,70	0,56

FONTE: A autora (2018).
NOTA: * Especificações sobre as dimensões. (REIS; MARTINS; LAGUARDIA, 2016).
†Teste de confiabilidade: > 0,90 - excelente; > 0,80 - bom; > 0,70 - aceitável; > 0,60 – questionável; > 0,50 - pobre; < 0,50 = inaceitável. (GEORGE; MALLERY, 2003).

O questionário obteve confiabilidade geral satisfatória, conforme os valores apresentados nos respectivos hospitais: Hospital A (0,89), Hospital B (0,90) e Hospital C (0,91). A dimensão oito avaliou a Frequência de relatos de eventos e apresentou a melhor consistência interna nos três hospitais.

A dimensão 10 analisou "Adequação de profissionais" e evidenciou-se

inconsistência inaceitável nos três hospitais participantes, conforme os repectivos valores de Alfa de *Cronbach*: Hospital A (0,26), Hospital B (0,47) e Hospital C (0,46). A dimensão 12 analisou “Respostas não punitivas ao erro”, apresentou Alfa de *Cronbach* igual a 0,10 no Hospital A e correspondeu ao menor valor de consistência interna entre as dimensões e hospitais avaliados.

5.2 EVENTOS ADVERSOS CIRÚRGICOS

Na Fase 1 da pesquisa, dos 678 prontuários analisados foram identificados pEA em 130 prontuários. Na Fase 2, foi confirmada a ocorrência de EA em 106 prontuários e, após análise dos EA, foram identificados 53 (Hospital A), 78 (Hospital B) e 55 (Hospital C), respectivamente, sendo que mais de 90% foram considerados evitáveis. Desse total de 186 EA, 15 resultaram em óbito (8%) e 39 (21%) foram classificados eventos graves.

5.2.1 Prevalência, evitabilidade, grau de dano e local de ocorrência dos eventos adversos cirúrgicos

Procedimentos cirúrgicos, amostra de prontuários analisados, pacientes com pEA e a prevalência e evitabilidade dos casos com EA cirúrgicos encontra-se na FLUXOGRAMA 4.

FLUXOGRAMA 4 – PREVALÊNCIA E EVITABILIDADE DE EVENTOS ADVERSOS EM TRÊS HOSPITAIS BRASILEIROS.

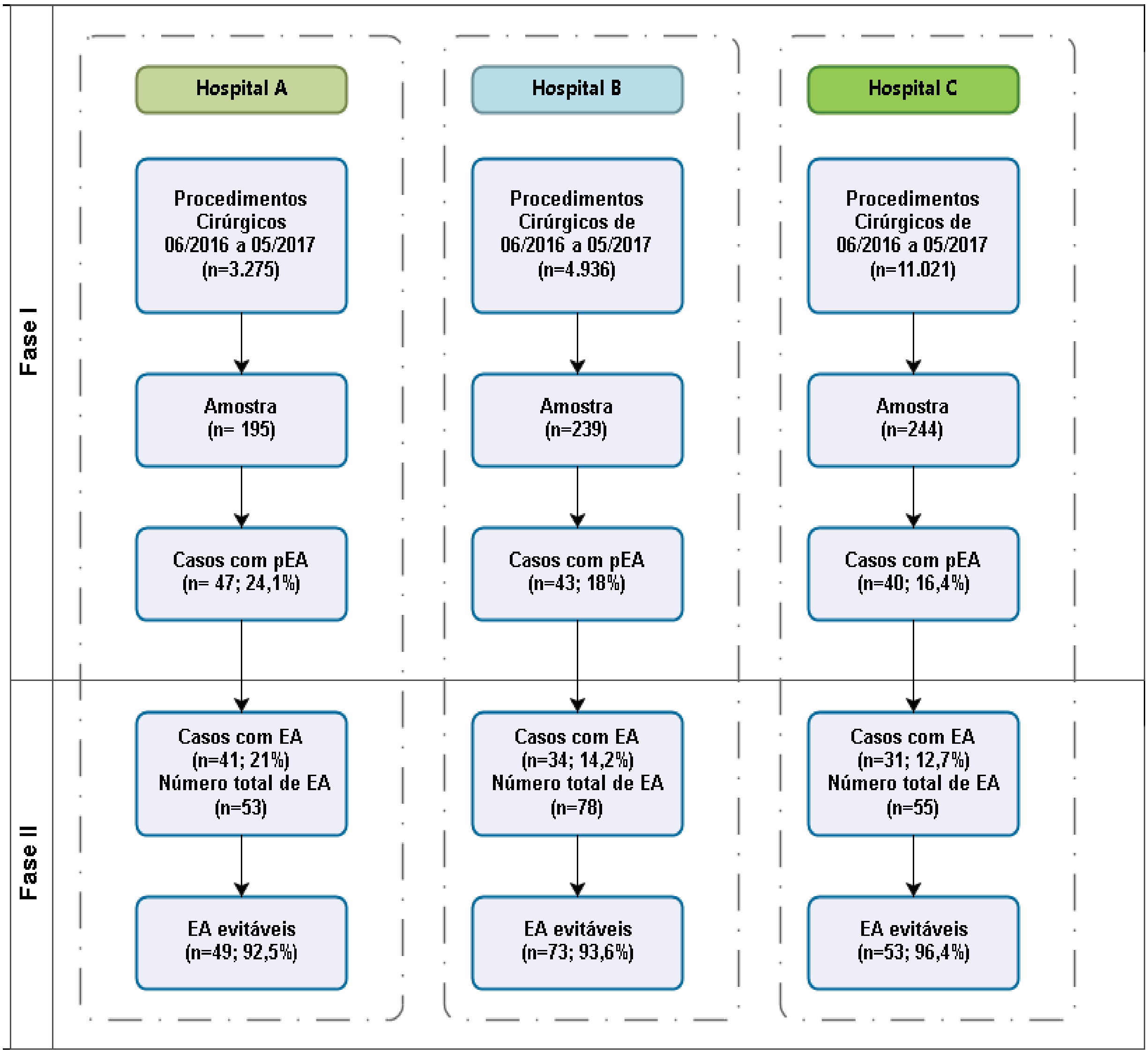

FONTE: A autora (2019).

5.2.2 Rastreamento de potenciais eventos adversos da Fase 1.

A frequência da identificação de critérios de rastreamento positivos para a ocorrência de potenciais EA (pEA), conforme os protocolos *Canadian Adverse Events Study* (CAES) e *Global Trigger Tool* (GTT) do *Institute of Healthcare Improvement* (IHI), estão descritos na TABELA 8 e TABELA 9, respectivamente.

Conforme Protocolo CAES, observa-se que no Hospital A não foram identificados os seguintes critérios de rastreamento nas amostras de prontuários avaliadas: 5. Retorno não planejado à sala de cirurgia, 8. Desenvolvimento de

alteração neurológica ausente na admissão, 10. Alta hospitalar inapropriada, 11. Parada cardiorrespiratória revertida, 13. Insatisfação com o cuidado recebido documentada no prontuário e 16. Quaisquer outros eventos indesejados.

No Hospital B não foram encontrados casos que necessitassem de 3. Transferência não planejada para a unidade de cuidados intensivos e semi-intensivos. No Hospital C não foram observados critérios de rastreamento relativos à 2. Reação adversa ao medicamento e 13. Insatisfação com o cuidado recebido documentada no prontuário.

Apenas o Hospital C apresentou 3 casos com rastreador positivo para o critério 13 - Insatisfação com o cuidado recebido documentada no prontuário, o que representou 2,34% do total de pacientes com pEA cirúrgico neste hospital.

Percentuais significativos foram verificados nos três hospitais em relação ao critério de rastreamento 7 - Outras complicações inesperadas ao tratamento: Hospital A (64,1%), Hospital B (17,19%) e Hospital C (15,88%). Comparado com os demais rastreadores e entre os hospitais, tal critério apresentou a maior porcentagem de rastreamento para pEA cirúrgicos no Hospital A.

Em relação ao rastreador 9 - óbito, constatou-se sua ocorrência nos três hospitais avaliados: Hospital A (3,8%), Hospital B (7,03%) e Hospital C (4,76%). O Hospital B destacou-se pelo maior número de pacientes cirúrgicos que foram à óbito (n=9), seguido do Hospital C (n=6) e Hospital A (n=2).

TABELA 8 – CRITÉRIOS POSITIVOS PARA RASTREAMENTO DE POTENCIAIS EVENTOS ADVERSOS CONFORME PROTOCOLO *CANADIAN ADVERSE EVENTS STUDY*. CURITIBA–PARANÁ, 2019

Protocolo Canadian Adverse Events Study – CAES	**HOSPITAL A**		**HOSPITAL B**		**HOSPITAL C**	
	**n**	**%**	**n**	**%**	**n**	**%**
1 - Ocorrência de lesão no paciente durante a internação	3	5,7	6	4,69	12	9,52
2 - Reação adversa ao medicamento	5	9,4	2	1,56	-	-
3 - Transferência não planejada para a unidade de cuidados intensivos e semi-intensivos	3	5,7	-	-	3	2,38
5 - Retorno não planejado à sala de cirurgia	-	-	21	16,41	18	14,29
6 - Remoção, lesão ou correção não planejada de um órgão ou estrutura durante cirurgia	2	3,8	12	9,38	18	14,29
7 - Outras complicações inesperadas ao tratamento	34	64,1	22	17,19	20	15,88
8 - Desenvolvimento de alteração neurológica ausente na admissão	-	-	2	1,56	3	2,38
9 – Óbito	2	3,8	9	7,03	6	4,76
10 – Alta hospitalar inapropriada	-	-	5	3,91	3	2,38
11 - Parada cardiorrespiratória revertida	-	-	3	2,34	4	3,17
12 - Infecção/ Septicemia	3	5,7	20	15,62	22	17,46
13 - Insatisfação com o cuidado recebido documentada no prontuário	-	-	3	2,34	-	-
15 - Duplicação do valor de creatinina normal na internação	1	1,8	1	0,78	6	4,76
16 - Quaisquer outros eventos indesejados (Embolia Pulmonar, Pneumotórax, Pneumoencéfalo, Pneumonia, Meningite, Trombose Venosa Profunda, Lesão por Pressão, Isquemia do Miocárdio, Infarto Agudo do Miocárdio, Falência Renal, Embolia Gasosa, Queda, Intoxicação por Opióide, Hipóxia, Pancreatite, Choque Séptico, Choque Hipovolêmcico, Hemorragia, Infecção do Trato Urinário, etc)	-	-	22	17,19	11	8,73
**TOTAL**	**53**	**100**	**128**	**100**	**126**	**100**

FONTE: A autora (2019).

De acordo com o protocolo GTT do IHI, verifica-se que nas amostras de prontuários analisadas do Hospital A não foram encontrados rastreadores positivos para ocorrência de pEA cirúrgicos quanto aos critérios: S1 - Retorno à sala cirúrgica, S2 – Mudança no procedimento e S7 – Ventilação mecânica superior a 24 horas no pós-operatório.

Observa-se que os Hospitais A e B foram semelhantes em não apresentarem rastreadores positivos para S4 – Intubação ou reintubação ou uso de *BiPap* na Unidade de Recuperação Pós-Anestésica e S5 – Raio X Intraoperatório ou em Unidade de Recuperação Pós-Anestésica. Tais rastreadores foram positivos apenas no Hospital C.

No Hospital C, o rastreador S2- Mudança no procedimento foi o único critério não identificado. Este mesmo rastreador foi encontrado apenas nas amostras de prontuários analisadas do Hospital B.

Destaca-se que o rastreador S8- Administração no intra-operatória de Epinefrina, Norepinefrina, Naloxona ou Ramazicon apresentou porcentagem maior no Hospital B (10,2%) quando comparado ao Hospital A (2,1%) e Hospital C (2,22%). Do mesmo modo, o rastreador S7- Ventilação mecânica superior a 24 horas no pós-operatório foi encontrado com valores semelhantes apenas no Hospital B (10,2%) e Hospital C (13,2%). E o rastreador S10- Lesão, reparo ou remoção de órgão durante o procedimento cirúrgico apresentou porcentagens superiores no Hospital B (12,2%) e Hospital C (19,8%), quando comparado ao Hospital A (4,2%).

Por fim, as maiores porcentagens para a ocorrência de pEA foram identificadas conforme o rastreador S11 - Ocorrência de qualquer complicação cirúrgica, que engloba diversas complicações clínicas que foram decorrentes da cirurgia como: Embolia Pulmonar, Trombose Venosa Profunda, Lesão por Pressão, Isquemia do Miocárdio, Falência Renal, etc. Os valores encontrados nos respectivos hospitais foram: Hospital A (83,3%), Hospital B (32,8%) e Hospital C (33,0%).

TABELA 9 – CRITÉRIOS POSITIVOS PARA RASTREAMENTO DE POTENCIAIS EVENTOS ADVERSOS CONFORME PROTOCOLO *GLOBAL TRIGGER TOOL*. CURITIBA–PARANÁ, 2019

**Global Trigger Tool - Institute of Healthcare Improvement**	**HOSPITAL A**		**HOSPITAL B**		**HOSPITAL C**	
	**n**	%	n	%	n	%
S1 - Retorno à sala cirúrgica	-	-	21	21,4	18	19,8
S2 – Mudança no procedimento	-	-	2	2,0	-	-
S3 – Admissão na Unidade de Terapia Intensiva no pós-operatório	3	6,2	2	2,0	3	3,2
S4 – Intubação ou reintubação ou uso de BiPap* na Unidade de Recuperação Pós-Anestésica	-	-	-	-	1	1,1
S5 – Raio X Intraoperatório ou em Unidade de Recuperação Pós-Anestésica	-	-	-	-	1	1,1
S6 - Óbito no intra ou pós-operatório	2	4,2	9	9,2	6	6,6
S7 – Ventilação mecânica superior a 24 horas no pós-operatório	-	-	10	10,2	12	13,2
S8 - Administração no intra-operatória de Epinefrina, Norepinefrina, Naloxona ou Ramazicon	1	2,1	10	10,2	2	2,2
S10 - Lesão, reparo ou remoção de órgão durante o procedimento cirúrgico	2	4,2	12	12,2	18	19,8
S11 - Ocorrência de qualquer complicação cirúrgica	40	83,3	32	32,8	30	33,0
**Total**	**48**	**100**	**98**	**100**	**91**	**100**

FONTE: A autora (2019).

5.2.3 Confirmação de eventos adversos da Fase 2

A prevalência de pacientes com EA cirúrgicos, número de EA por paciente grau de dano e evitabilidade estão apresentados na TABELA 10.

TABELA 10 – DISTRIBUIÇÃO DO NÚMERO DE EVENTOS ADVERSOS POR PACIENTE, GRAU DE DANO E EVITABILIDADE. CURITIBA–PARANÁ, 2019

Variável	HOSPITAL A		HOSPITAL B		HOSPITAL C		Valor de p*
	n	%	n	%	N	%	
**Prevalência**							
Não	154	79,0	205	85,8	213	87,3	
Sim	41	21,0	34	14,2	31	12,7	**0,044**
**Total**	**195**	**100**	**239**	**100**	**244**	**100**	
**Internação**							
Não	27	65,9	1	2,9	2	6,5	
Sim	14	34,1	33	97,1	29	93,5	**<0,001**
**Total**	**41**	**100**	**34**	**100**	**31**	**100**	
**Número**							
Um evento	31	75,6	14	41,2	17	54,8	
Dois eventos	8	19,5	12	35,3	6	19,4	
Três a nove eventos	2	4,9	8	23,5	8	25,8	**0,023**
**Total**	**41**	**100**	**34**	**100**	**31**	**100**	
**Grau de dano**							
Leve	24	45,3	16	20,5	4	7,3	
Moderado	23	43,4	37	47,5	28	50,9	
Grave	5	9,4	16	20,5	18	32,7	
Óbito	1	1,9	9	11,5	5	9,1	**<0,001**
**Total**	**53**	**100**	**78**	**100**	**55**	**100**	
**Evitabilidade**							
Fortemente prevenível	17	32,1	31	39,8	14	25,5	
Potencialmente prevenível	32	60,4	42	53,8	39	70,9	**0,365**
Potencialmente não prevenível	4	7,5	5	6,4	2	3,6	
**Total**	**53**	**100**	**78**	**100**	**55**	**100**	

FONTE: A autora (2019).
*Teste de Qui-quadrado, p<0,05

A detecção do EA cirúrgico durante a internação foi prevalente no Hospital B e Hospital C. O contrário foi observado no Hospital A, onde a maior parte dos EA foram detectados por meio dos registros realizados pela equipe médica no retorno ambulatorial do paciente. Esta variável apresentou significância estatística entre os hospitais (p<0,001).

A maioria dos pacientes avaliados nos três hospitais apresentaram um EA cirúrgico: Hospital A (75,6%), Hospital B (41,2%) e Hospital C (54,8%). Porém, destaca-se a ocorrência de dois EA cirúrgicos no Hospital B (35,3%), quando comparado aos demais hospitais.

Houve significância estatística nos Hospitais B e C quando associados ao Hospital A (p=0,017), em relação à ocorrência de três a nove eventos por paciente: dados semelhantes foram verificados em oito pacientes no Hospital B (23,5%) e Hospital C (25,8%), diferentemente da taxa verificada no Hospital A (4,9%), que ocorreu em dois pacientes.

Quanto ao grau de dano ocasionado pelos EA prevaleceu a classificação de danos moderados: Hospital A (43,4%), Hospital B (47,5%) e Hospital C (50,9%). No entanto, os danos graves no Hospital B merecem destaque (20,5%) e foi equivalente a taxa registrada de danos leves (20,5%).

A variável óbito apresentou significância estatística (p<0,001) entre os hospitais: Hospital A (1,9%), Hospital B (11,5%) e Hospital C (9,1%). A associação dos resultados mostra que o Hospital B apresentou maior taxa de óbitos.

Em relação a classificação da evitabilidade dos EA, as maiores porcentagens referem-se aos "potencialmente preveníveis", conforme as respectivas taxas: Hospital A (60,4%), Hospital B (53,8%) e Hospital C (70,9%), seguido da classificação "fortemente preveníveis": Hospital A (32,1%), Hospital B (39,8%) e Hospital C (25,5%).

5.2.4 Características demográficas e clínicas dos pacientes com eventos adversos cirúrgicos

O perfil demográfico e clínico dos pacientes que apresentaram evento adverso cirúrgico constam na TABELA 11.

TABELA 11 – PERFIL DEMOGRÁFICO E CLÍNICO DOS PACIENTES COM EVENTOS ADVERSOS. CURITIBA–PARANÁ, 2019

**Variável**	**HOSPITAL A**		**HOSPITAL B**		**HOSPITAL C**		**Valor de p***
	**n**	**%**	**n**	**%**	**n**	**%**	
**Sexo**							
Masculino	13	31,7	16	47,1	22	71,0	
Feminino	28	68,3	18	52,9	9	29,0	**0,004**
**Faixa etária**							
< 60 anos	31	75,6	24	72,7	23	74,2	
≥ 60 anos	10	24,4	9	27,3	8	25,8	0,961
**Total**	**41**	**100**	**34**	**100**	**31**	**100**	
**Comorbidades/ Fatores de risco**							
Hipertensão arterial severa	9,0	22,0	12	35,3	5	16,1	0,178
Tabagismo	10,0	24,4	1	2,9	3	9,7	**0,019**
Diabetes *Mellitus*	6,0	14,6	6	17,7	2	6,5	0,388
Obesidade	3,0	7,3	0	0	1	3,2	0,249
Hipotireoidismo	2,0	4,9	1	2,9	0	0,0	-
Dislipidemia	1,0	2,4	2	5,9	2	6,5	0,676
Doença de Chron	1,0	2,4	0	0,0	0	0,0	**-**
Outros†	6,0	14,6	14	41,2	11	35,5	**0,028**

FONTE: A autora (2019).
* Teste de Qui-quadrado, p<0,05
†Cardiopatia; Artrite; Osteoporose; Sarcoma; Metástase; Hepatite; Problema renal e Etilismo.

A média de idade dos pacientes foi de 49,3 (DP±15,1) no Hospital A, 47,6 (DP±18,5) no Hospital B e 48,0 no Hospital C (DP±20,1).

Pacientes do sexo feminino foram prevalentes nos Hospitais A e B. No Hospital C verificou-se a prevalência do sexo masculino.

A Hipertensão arterial e a variável "Outros" (Cardiopatia, Artrite, Osteoporose, Sarcoma, Metástase, Hepatite, Problema renal, Drogadicção e Etilismo) foram a comorbidade/fator de risco preponderantes entre os pacientes com EA cirúrgicos nos três hospitais. O tabagismo foi o fator de risco mais prevalente no Hospital A (24,4%), com significância estatística (p=0,019) em relação ao Hospital B (2,9%) e Hospital C (9,7%).

5.2.5 Características da internação e procedimento cirúrgico-anestésico dos pacientes com eventos adversos cirúrgicos

As característivas relativas à internação estão descritas na TABELA 12.

TABELA 12 – DISTRIBUIÇÃO DAS VARIÁVEIS RELATIVAS À INTERNAÇÃO DOS PACIENTES COM EVENTOS ADVERSOS. CURITIBA–PARANÁ, 2019

**Variável**	**HOSPITAL A**		**HOSPITAL B**		**HOSPITAL C**		**Valor de p***
	**n**	**%**	**n**	**%**	**n**	**%**	
**Mês do internamento**							
Janeiro a junho	23	56,1	17	50,0	16	51,6	
Julho a dezembro	18	43,9	17	50,0	15	48,4	0,859
**Dias de internamento**							
1 a 2 dias	11	26,8	4	11,8	4	12,9	
3 a 5 dias	15	36,6	7	20,6	6	19,4	
≥ 6 dias	15	36,6	23	67,6	21	67,7	**0,041**
**Tempo de internação pré-operatório**							
< 24 horas	35	85,4	19	55,9	26	83,9	
≥ 24 horas	6	14,6	15	44,1	5	16,1	**0,006**
**Readmissão**							
Não	37	90,2	24	70,6	17	54,8	
Sim	4	9,8	10	29,4	14	45,2	**0,003**
**Total**	**41**	**100**	**34**	**100**	**31**	**100**	

FONTE: A autora (2019).
*Teste não-paramétrico de Kruskal-Wallis; teste de Qui-quadrado, p<0,05

Em relação ao número de dias de internamento equivalente a ≥ 6 dias foi constatado a significância estatística entre os hospitais avaliados (p=0,041), pois o Hospital A apresentou taxa de incidência menor de pacientes neste período (36,6%), comparado ao Hospital B (67,6%) e Hospital C (67,7%), que apresentaram resultados semelhantes. Em relação ao tempo de internação pré-operatório dos pacientes, houve significância estatística entre os hospitais quanto ao período ≥ 24 horas (p=0,006), pois a porcentagem resultante no Hospital B (44,1%) foi superior a encontrada no Hospital A (14,6%) e Hospital C (16,1%). Os dados sobre episódios de readmissão

mostram significância estatística entre os hospitais (p=0,003): Hospital A (9,8%), Hospital B (29,4%) e Hospital C (45,2%). Deste modo, o Hospital C apresentou o maior número de re-internações em decorrência de EA cirúrgicos.

Outra variável que não consta na TABELA 12 e que está relacionada a média de internamento (em dias) dos pacientes com EA cirúrgico, desde o primeiro dia de internamento até a alta hospitalar, mas que passou por análise estatística evidenciou os seguintes resultados: 4 dias no Hospital A (1 – 98 dias), 12 dias no Hospital B (1-72 dias) e 17 dias no Hospital C (1-117 dias); Tais resultados apresentaram significância estatística (p=0,002), após análise com o Teste não-paramétrico de Kruskal-Wallis e teste de Qui-quadrado p<0,05, na associação entre os Hospitais: Hospitais A x B: p=0,004; A x C: p=0,001; B x C: 0,667.

A seguir, apresentam-se os resultados sobre as variáveis das cirurgias e anestesias realizadas nos pacientes que sofreram EA cirúrgico, conforme TABELA 13.

TABELA 13 – DISTRIBUIÇÃO DAS VARIÁVEIS RELATIVAS AO PROCEDIMENTO CIRÚRGICO E ANESTÉSICO DOS PACIENTES COM EVENTOS ADVERSOS. CURITIBA–PARANÁ, 2019

Variável	Classificação	HOSPITAL A		HOSPITAL B		HOSPITAL C		Valor de p*
		n=41	%	n=34	%	n=31	%	
Especialidade	Ortopedia /Traumatologia	6	14,6	5	14,7	16	51,6	
	Cx. Geral / Cx. Ap. Digestivo	30	73,2	22	64,7	9	29,0	
	Cx. Plástica e Tx. Hepático*	2	4,9	0	0	0	0	
	Neurocirurgia	3	7,3	7	20,6	6	19,4	
Classificação cirurgica	Eletiva	33	80,5	20	58,8	8	25,8	
	Emergência	8	19,5	8	23,5	15	48,4	
	Urgência	0	0	6	17,7	8	25,8	**<0,001**
Risco cirúrgico	ASA I	9	22,0	10	29,4	17	54,8	
	ASA II	22	53,7	5	14,7	9	29,0	
	ASA III	10	24,4	13	38,2	5	16,1	
	ASA IV	0	0	6	17,7	0	0	**<0,001**
Grau de contaminação	Limpa	22	53,6	22	64,8	18	58,1	
	Contaminada	13	31,7	8	23,5	8	25,8	
	Potencialmente contaminada	4	9,8	3	8,8	2	6,4	
	Infectada	2	4,9	1	2,9	3	9,7	**0,870**
Anestesia	Geral	16	39,0	27	79,4	15	48,4	
	Combinada	9	22,0	3	8,8	8	25,8	
	Raquidiana	14	34,2	0	0,0	2	6,5	
	Local	1	2,4	0	0,0	0	0,0	
	Peridural	1	2,4	3	8,8	6	19,4	
	Plexular	0	0	1	2,9	0	0,0	
Duração cirúrgica (minutos)	<120	18	43,9	22	64,7	15	48,4	
	>120	23	56,1	12	35,3	16	51,6	**0,179**

FONTE: A autora (2019).
*Teste de Qui-quadrado, p<0,05
* Tx. Hepático = exclusivo no Hospital A

Na análise sobre a prevalência de EA em relação à especialidade cirúrgica, observa-se que no Hospital A (73,2%) e Hospital B (64,7%) os eventos ocorreram com prevalência nos pacientes operados pela equipe médica da Cirurgia Geral / Cirurgia do Aparelho Digestivo e, no Hospital C (51,6%), os eventos ocorreram com prevalência nos pacientes operados pela equipe médica da Ortopedia.

Quanto à classificação cirúrgica, as cirurgias eletivas foram prevalentes no Hospital A (80,5%) e Hospital B (58,8%), porém, no Hospital C, destacaram-se as cirurgias de emergência (48,4%). Houve significância estatística entre os hospitais em relação às cirurgias de urgência ($p<0,001$), que ocorreram no Hospital B (17,7%) e Hospital C (25,8%). No Hospital A não foram registradas cirurgias neste tipo de classificação.

Os resultados em relação ao risco cirúrgico dos pacientes foram diferentes nos três hospitais analisados: no Hospital A prevaleceu ASA II (53,7%), Hospital B foi ASA III (38,2%) e no Hospital C foi ASA I (54,8%). Houve significância estatística nos pacientes com risco cirúrgico ASA IV ($p<0,001$) pois, foram identificados somente no Hospital B (17,7%).

Nos três hospitais observou-se a ocorrência de maiores porcentagens de cirurgias limpas e entre os procedimentos anestésicos, a anestesia geral foi prevalente.

As TABELAS 14, 15 e 16 representam a distribuição da frequência dos procedimentos cirúrgicos com evolução para EA identificados em cada hospital participante da pesquisa.

TABELA 14 – DISTRIBUIÇÃO DA FREQUÊNCIA DOS PROCEDIMENTOS CIRÚRGICOS NO HOSPITAL A. CURITIBA–PARANÁ, 2019

Cirurgias Hospital A	n	%
Hernioplastia incisional	5	12,2
Videocolescitectomia	4	9,8
Hernioplastia inguinal	2	4,9
Herniorrafia umbilical	2	4,9
Apendicectomia	2	4,9
Artroplastia total de joelho	2	4,9
Artroplastia total de quadril	2	4,9
Laparotomia exploradora	2	4,9
Colpoperineoplastia	1	2,4
Cranioplastia	1	2,4
Descompressão de fossa posterior	1	2,4
Enterectomia	1	2,4
Esfincteroplastia	1	2,4
Fistulotomia	1	2,4
Hepatectomia	1	2,4
Hernioplastia umbilical	1	2,4
Herniorrafia Incisional	1	2,4
Hipofisectomia transesfenoidal	1	2,4
Ileocolectomia	1	2,4
Jejunostomia	1	2,4
Mamoplastia	1	2,4
Pancreatectomia parcial	1	2,4
Reconstrução de períneo/retalho fasciocutâneo	1	2,4
Reposicionamento de ileostomia	1	2,4
Ressecção de lipossarcoma	1	2,4
Retirada de material de síntese	1	2,4
Sigmoidectomia	1	2,4
Tireoidectomia total	1	2,4
**Total**	**41**	**100**

FONTE: A autora (2019).

No Hospital A, as Hernioplastias, incisional (12,2%), inguinal (4,8%) e umbilical (2,4%) e, as Herniorrafias, incisional (2,4%) e umbilical (4,8%) foram as cirurgias mais prevalentes, com resultado equivalente a 26,84% (n=11). Em seguida destacaram-se as Artroplastias de joelho (4,8%) e quadril (4,8%) e as Videocolecistectomias (9,7%), que resultaram em 19,5% (n= 8) das cirurgias que evoluíram com a ocorrência de EA.

TABELA 15 – DISTRIBUIÇÃO DA FREQUÊNCIA DOS PROCEDIMENTOS CIRÚRGICOS NO HOSPITAL B. CURITIBA–PARANÁ, 2019

**CIRURGIAS HOSPITAL B**	**n**	**%**
Colecistectomia	8	12,1
Laparotomia Exploradora (LE)	6	9,1
Craniotomia	4	6,1
Drenagem de Abscesso	3	4,5
Colorrafia	2	3,0
Derivação Ventricular Externa	2	3,0
Enteroanastomose	2	3,0
Enterorrafia	2	3,0
Hepatectomia	2	3,0
Liberação de Aderências	2	3,0
Hernioplastia inguinal	2	3,0
Osteossíntese para Fixação de Fratura	2	3,0
Colédocolitíase	1	1,5
Colostomia	1	1,5
Craniectomia Descompressiva bi-frontal	1	1,5
Cranioplastia	1	1,5
Desbridamentos de Tecidos Desvitalizados e Curativo Cirúrgico	1	1,5
Drenagem de Coleções intra-abdominais	1	1,5
Drenagem Pleural	1	1,5
Duodenopancreatectomia	1	1,5
Enterectomia	1	1,5
Gastrorrafia	1	1,5
Hepatorrafia	1	1,5
Hernioplastia epigástrica	1	1,5
Herniorrafia inguinal	1	1,5
Herniorrafia umbilical	1	1,5
Ileostomia em Alça	1	1,5
Implante de cateter intraventricular (PIC)	1	1,5
Ligadura Segmento Entérico	1	1,5
Limpeza Cirúrgica	1	1,5
Microcirurgia para Ressecção de Tu de Hipófise	1	1,5
Microdissecção de Tu cerebral	1	1,5
Peritoneostomia com tela orgânica	1	1,5
Rafia da Veia Porta	1	1,5
Rafia do Duodeno na 2ª porção	1	1,5
Reabordagem Cirúrgica para Correção de fratura Membro Inferior	1	1,5
Ressecção de Órgãos	1	1,5
Ressecção de Tu Retroperitoneal	1	1,5
Retossigmoidectomia	1	1,5
Revisão de Artroplastia Total de Quadril	1	1,5
Tratamento de Peritonite	1	1,5
**Total**	**66**	**100**

FONTE: A autora (2019).

No Hospital B verificou-se que os pacientes durante a mesma abordagem cirúrgica eram submetidos a múltiplos procedimentos, o que mostra a diversidade de cirurgias realizadas.

As cirurgias de Colecistectomia (12,1%), Laparotomias Exploradoras (9,1%), Enterectomia (1,5%), Enteroanastomose (3,0%), Enterorrafia (3,0%) e Ligadura de Segmento Entérico (1,5%), em conjunto com as cirurgias neurológicas de Craniectomia Descompressiva bi-frontal (1,5%), Cranioplastia (1,5%), Craniotomia (6,1%) e Derivação Ventricular Externa (3,0%) e Microdissecção de Tumor Cerebral (1,5%) foram prevalentes e representaram o total de 43,8 % (n=29) dos procedimentos que evoluíram com a ocorrência de EA.

Em seguida, destacaram-se as Hernioplastias, epigástrica (1,5%) e inguinal (3,0%) e, as Herniorrafias, inguinal (1,5%) e umbilical (1,5%), bem como, Drenagens de abscesso (4,5%), de Coleções intra-abdominais (1,5%) e pleural (1,5%) que resultaram em 15% (n=10) das cirurgias que evoluíram com a ocorrência de EA.

.

TABELA 16 – DISTRIBUIÇÃO DA FREQUÊNCIA DOS PROCEDIMENTOS CIRÚRGICOS NO HOSPITAL C. CURITIBA–PARANÁ, 2019

**Cirurgias Hospita C**	**n**	**%**
Laparotomia exploradora	5	8,8
Redução aberta e fixação interna de fratura	5	8,8
Craniectomia	4	7,0
Instalação de fixador externo para estabilização de fratura óssea	4	7,0
Osteossíntese para fixação de fratura	3	5,3
Desbridamentos de tecidos desvitalizados e curativo cirúrgico	3	5,3
Gastrorrafia	2	3,5
Artroplastia parcial de quadril / Artroplastia total de quadril	2	3,5
Hernioplastia inguinal	2	3,5
Tenorrafia em tendão de aquiles	2	3,5
Toracostomia para drenagem de pneumotórax	2	3,5
Colecistectomia	1	1,8
Colorrafia de Intestino transverso	1	1,8
Correção cirúrgica de fratura	1	1,8
Correção de sindactilia	1	1,8
Craniotomia descompressiva	1	1,8
Drenagem de hematoma cerebelar	1	1,8
Enterorrafia	1	1,8
Esplenectomia	1	1,8
Fasciotomia em membro inferior	1	1,8
Fechamento de ileostomia	1	1,8
Frenorrafia	1	1,8
Hernioplastia epigástrica	1	1,8
Hernioplastia umbilical	1	1,8
Limpeza cirúrgica em membro inferior	1	1,8
Nefrorrafia	1	1,8
Rafia de bexiga	1	1,8
Reconstrução craniana	1	1,8
Reconstrução de trânsito	1	1,8
Redução incruenta de luxação de ombro	1	1,8
Retirada de material de síntese	1	1,8
Revisão de tratamento cirúrgico de fratura de patela	1	1,8
Tratamento cirúrgico de fístula liquórica	1	1,8
Tratamento cirúrgico de hematoma subdural agudo	1	1,8
**Total**	**57**	**100**

FONTE: A autora (2019).

No Hospital C, as cirurgias que evoluíram com a ocorrência de EA, foram prevalentes na especialidade de Ortopedia/Traumatologia: Artroplastia parcial de quadril / Artroplastia total de quadril (3,5%), Correção cirúrgica de fratura (1,8%), Correção de sindactilia (1,8%), Desbridamentos de tecidos desvitalizados pós

trauma e Curativo cirúrgico (5,3%), Instalação de fixador externo para estabilização de fratura óssea (7%), Osteossíntese para fixação de fratura (5,3%), Redução aberta e fixação interna (RAFI) de fratura (8,8%), Redução incruenta de luxação de ombro (1,8%), Retirada de material de síntese (1,8%), Revisão de tratamento cirúrgico de fratura de patela (1,8%) e Tenorrafia em tendão de aquiles (3,5%), o que representou um total de 42,4% (n= 24) dos procedimentos realizados.

Em seguida destacaram-se as cirurgias: Laparotomia exploradora (8,8%) e as Hernioplastias, epigástrica (1,8%), inguinal (3,5%) e umbilical (1,8%), em conjunto com cirurgias neurológicas, Craniectomia (7,0%), Craniotomia descompressiva (1,8%), Drenagem de hematoma cerebelar (1,8%), Reconstrução craniana (1,8%), Tratamento cirúrgico de fístula liquórica (1,8%) e de Hematoma subdural agudo (1,8%), que resultaram em 31,9% (n=18) das cirurgias que evoluíram com a ocorrência de EA.

5.2.6 Classificação dos eventos adversos cirúrgicos e tipo de complicação cirúrgica

Os GRÁFICOS 4, 5 e 6 representam os EA cirúrgicos conforme o tipo de complicação cirúrgica, respectivamente nos Hospitais A, B e C.

GRÁFICO 4 – EVENTOS ADVERSOS NO HOSPITAL A.

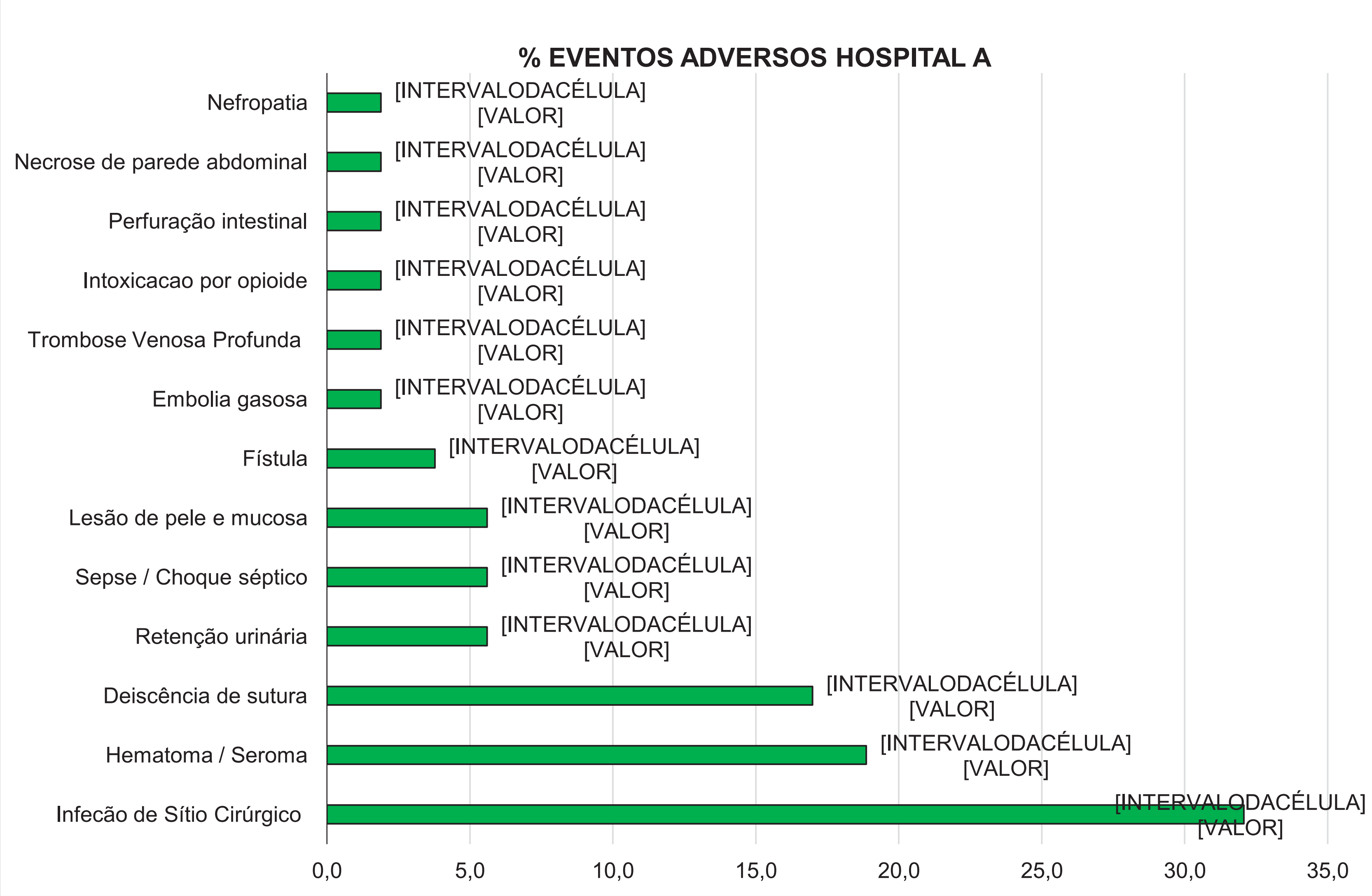

FONTE: A autora (2019)

GRÁFICO 5 – EVENTOS ADVERSOS NO HOSPITAL B.

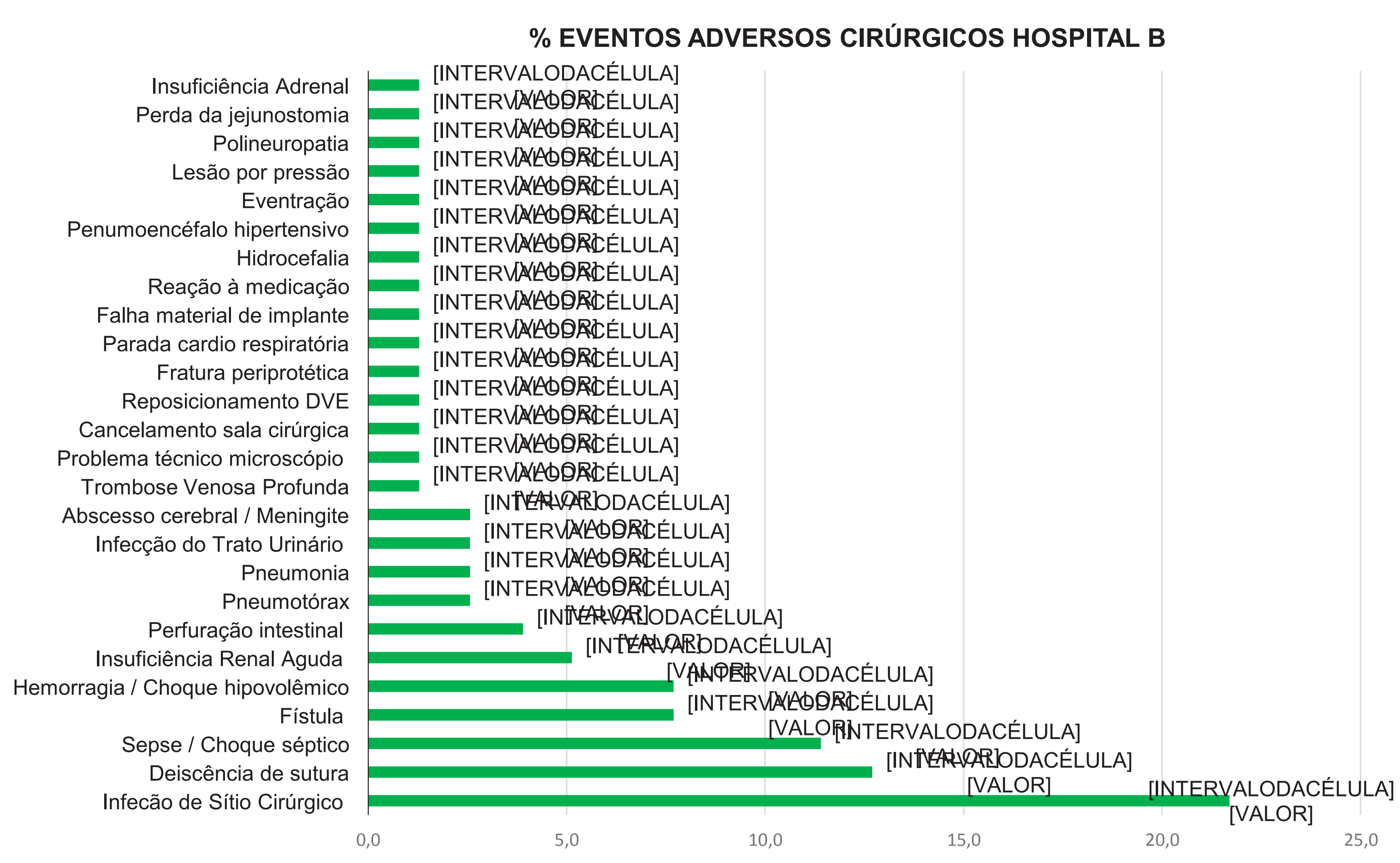

FONTE: A autora (2019).

GRÁFICO 6 – EVENTOS ADVERSOS NO HOSPITAL C.

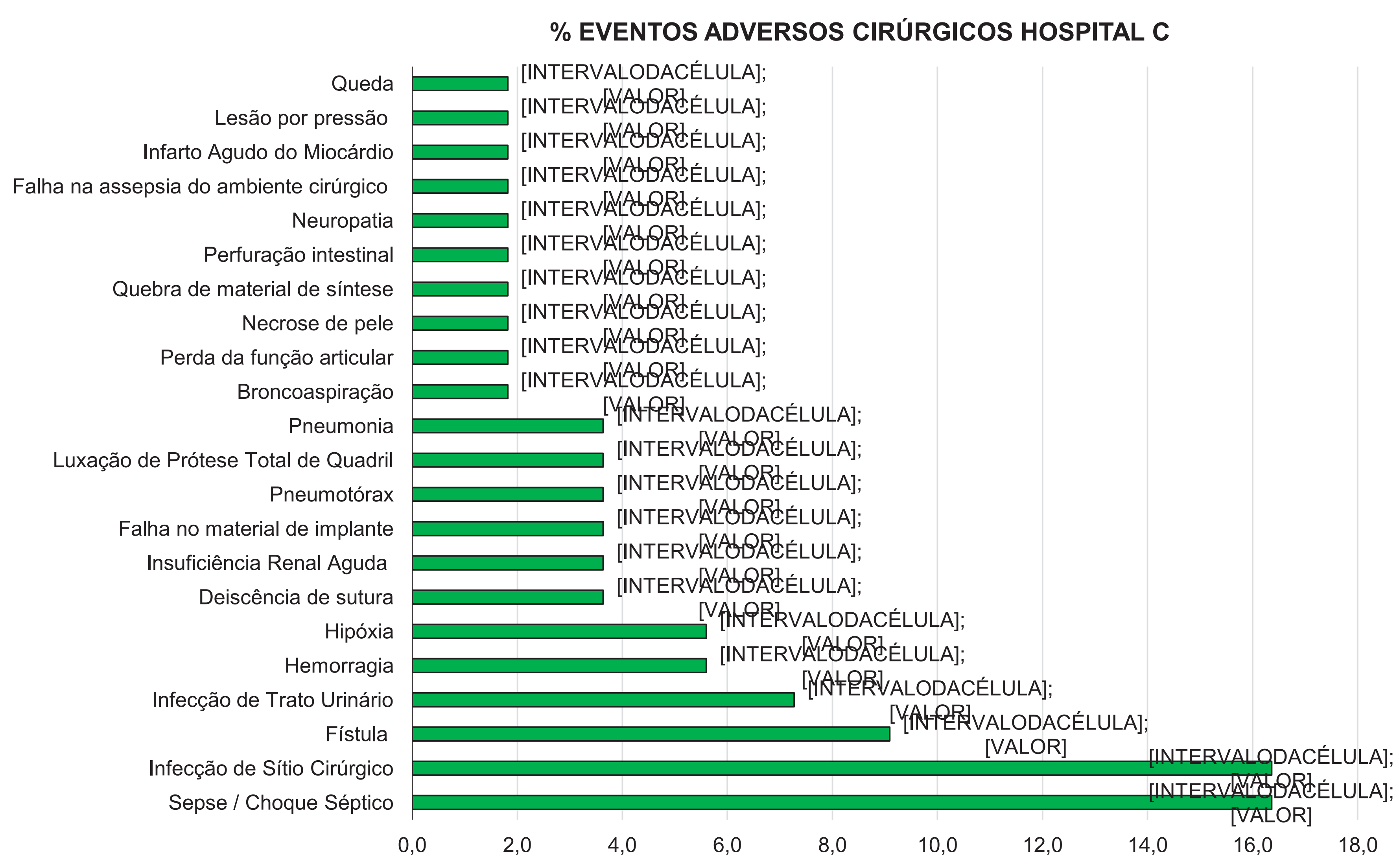

FONTE: A autora (2019).

De acordo com a GRÁFICO 4, no Hospital A identificou-se 53 EA cirúrgicos classificados em:

- Infecção Associada à Atenção de Saúde (IRAS) = Infecção de Sítio Cirúrgico (32,1%/n=17); Deiscência de sutura (17%/n=9) e Sepse/Choque Séptico (5,6%/n=3), totalizando 29 eventos (54,7%).

- Processo clínico / procedimentos = Hematoma/Seroma (18,9%/n=10); Retenção urinária (5,6%/n=3); Fístula pancreática (3,8%/n=2); Embolia gasosa (1,9%/n=1); Trombose Venosa Profunda (1,9%/n=1); Necrose de parede abdominal (1,9%/n=1) e Nefropatia (1,9%/n=1), totalizando 19 eventos (35,9%)

- Medicação/fluídos endovenosos = Intoxicação por opióide (1,9%/n=1), totalizando um evento (1,9%).

- Acidentes com o paciente = Lesão de pele e mucosas (5,6%/n=3) e Perfuração de cólon transverso (1,9%/n=1), totalizando quatro eventos (7,5%).

Conforme GRÁFICO 5, no Hospital B detectou-se 78 EA cirúrgicos classificados em:

Infecção Associada aos Cuidados de Saúde = Infecção de Sítio Cirúrgico (21,7%/n=17); Sepse/Choque séptico (11,3%/n=9); Pneumonia (2,6%/n=2); ITU (2,6%/n=2); Abscesso cerebral / Meningite (2,6%/n=2) e Deiscência de sutura (12,6%/n=10), totalizando 42 eventos (53,4%);

Processo clínico/procedimentos = Fístulas (7,7%/n=6); Hemorragia / Choque hipovolêmico (7,7%/ n=6); IRA (5,2%/n=4); Pneumotórax (2,6%/n=2); TVP (1,3%/n=1); Fratura periprotética (1,3%/n=1); Parada cardio respiratória (1,3%/n=1); Hidrocefalia (1,3%/n=1); Penumoencéfalo hipertensivo (1,3%/n=1); Eventração (1,3%/n=1); Polineuropatia (1,3%/n=1), totalizando 25 eventos (32,3%).

- Dispositivo/ equipamento médico = Problema técnico microscópio (1,3%/n=1); Falha material de implante (1,3%/n=1); Perda da jejunostomia (1,3%/n=1); Reposicionamento DVE (1,3%/n=1), totalizando quatro eventos (5,2%).

- Medicação / fluídos endovenosos = Insuficiência Adrenal (1,3%/n=1); Reação à medicação (1,3%/n=1), totalizando de 2 eventos (2,6%).

- Acidentes com o paciente= Perfuração intestinal (3,9%/n=3); Úlcera de pressão (1,3%/n=1), totalizando quatro eventos (5,2%).

- Recursos / gestão organizacional = Cancelamento sala cirúrgica (1,3%/n=1), totalizando um evento (1,3%).

De acordo com a GRÁFICO 6, no Hospital C evidenciou-se 55 EA cirúrgicos classificados em:

- Infecção Associada aos Cuidados de Saúde = Sepse / Choque Séptico (16,4%/n=9); Infecção de Sítio Cirúrgico (16,4%/n=9); Infecção de Trato Urinário (7,3%/n=4); Pneumonia (3,6%/n=2); e Deiscência de sutura (3,6%/n=2), totalizando 26 eventos (47,3%).

- Processo clínico/procedimentos = Fístulas (9,1%/n=5; Hemorragia (5,6%/n=3); Hipóxia (5,6%/n=3); Insuficiência Renal Aguda (3,6%/n=2); Pneumotórax (3,6%/n=2); Broncoaspiração (1,8%/n=1); Perda da função articular (1,8%/n=1); Necrose de pele (1,8%/n=1); Neuropatia (1,8%/n=1) e Infarto Agudo do Miocárdio (1,8%/n=1), totalizando 20 eventos (36,5%).

- Dispositivo / equipamento médico = Falha material de implante (3,6%/n=2); Luxação de Prótese Total de Quadril (3,6%/n=2) e Quebra de material de síntese (1,8%/n=1), totalizando cinco eventos (9%).

- Acidentes com o paciente = Perfuração de intestino delgado por cautério (1,8% (n=1); Lesão por pressão grau II (1,8%/n=1) e Queda (1,8%/n=1), totalizando três eventos (5,4%).

- Infraestrutura / local / instalações = Falha na assepsia do ambiente cirúrgico (1,8%/n=1), totalizando um evento (1,8%).

A análise geral dos dados dos três hospitais participantes demonstra que as maiores porcentagens de EA cirúrgicos foram classificados na categoria Infecção Associada aos Cuidados de Saúde: Hospital A (54,7%), Hospital B (53,5%) e Hospital C (47,3%).

5.3 TRADUÇÃO, ADAPTAÇÃO TRANSCULTURAL E VALIDAÇÃO DO MÓDULO CIRÚRGICO *GLOBAL TRIGGER TOLL*

O processo metodológico para tradução, adaptação transcultural para contexto brasileiro e validação do módulo cirúrgico do GTT do *Institute of Helthcare Improvement* foi realizado em todas as etapas propostas na metodologia. A finalidade foi adaptar instrumento para ser utilizado, durante as investigações para identificação e mensuração de EA cirúrgicos, por meio das avaliações retrospectivas de amostras aleatórias de prontuários.

Na primeira etapa foram elaboradas duas versões pelos tradutores participantes denominadas **V1** e **V2**; as discrepâncias mais frequentes foram relacionadas às palavras ou termos (ex.: sala operatória e sala de cirurgia; morte e óbito; admissão e acesso; reparo e tratamento; operação e cirurgia).

Na segunda etapa foi realizada a síntese das traduções (V1 e V2) para dar origem a versão **V3**. As discrepâncias encontradas na etapa anterior foram analisadas e optou-se pelo termos considerados mais usuais para o contexto brasileiro.

Na terceira etapa, as versões retrotraduzidas denominadas **V4** e **V5** foram equivalentes e, houve modificações no tempo verbal em algumas descrições das orientações sobre os *triggers.* As diferenças de retrotraduções foram consideradas palavras sinônimas. Deste modo, foi formulada a versão **V6** que correspondeu ao instrumento original.

Na quarta etapa, o comitê de especialistas realizou melhorias com alterações e adaptações transculturais relevantes para validação de conteúdo do instrumento e formulação da versão **V7,** que foi utilizada posteriormente no pré-teste.

A seguir, a TABELA 17 apresenta as avaliações de cada especialista e a média dos percentuais de concordância, considerando-se os 11 *triggers* (S1 até S11) e suas respectivas orientações, bem como o Índice da Validade de Conteúdo (IVC). Para esta pesquisa foi estabelecido o valor de IVC≥0,8 e foram agrupadas as opções de resposta discordo totalmente e discordo (-1 e -2) e as opções de resposta concordo e concordo totalmente (+1 e +2). Na primeira rodada Delphi de avaliação, o primeiro bloco do Questionário para Especialista (APÊNDICE 4) que contemplou a avaliação dos 11 *triggers* e das 11 respectivas orientações, obteve nível de concordância 92,4% e IVC médio igual a 1,38. Deste modo, o instrumento não foi submetido à nova rodada de validação pelos especialistas.

TABELA 17 – CONCORDÂNCIA ENTRE OS ESPECIALISTAS (N=6) E IVC OBTIDO NO PROCESSO DE VALIDAÇÃO DO PROCESSO DE TRADUÇÃO E ADAPTAÇÃO TRANSCULTURAL DO MÓDULO CIRÚRGICO DO GTT DO IHI. CURITIBA–PARANÁ, 2019

Trigger/Orientações S1 - S11	Concordam %	Indiferente %	Discordam %	Validade de Conteúdo (IVC)*
Trigger S1	83	0	17	1,17
Orientações S1	83	0	17	1,17
Trigger S2	100	0	0	1,50
Orientações S2	100	0	0	1,50
Trigger S3	83	17	0	1,33
Orientações S3	83	17	0	1,33
Trigger S4	100	0	0	1,67
Orientações S4	100	0	0	1,67
Trigger S5	100	0	0	1,67
Orientações S5	100	0	0	1,67
Trigger S6	100	0	0	1,50
Orientações S6	100	0	0	1,50
Trigger S7	83	0	17	1,17
Orientações S7	83	0	17	1,17
Trigger S8	83	0	17	1,17
Orientações S8	83	0	17	1,17
Trigger S9	100	0	0	1,50
Orientações S9	100	0	0	1,50
Trigger S10	100	0	0	1,50
Orientações S10	100	0	0	1,67
Trigger S11	83	0	17	1,17
Orientações S11	83	0	17	1,17
**Média**	92,4	-	-	1,38

FONTE: A autora (2019).
* IVC - Para consenso entre os especialistas recomeda-se os valores entre 0,5 e 0,8 (CASTRO; RESENDE, 2009).

O segundo bloco do Questionário para Especialista (APÊNDICE 4) possibilitou a avaliação das equivalências semântica, idiomática, conceitual, cultural e de conteúdo do instrumento. Deste modo, a Tabela 18 apresenta tais avaliações, a média dos percentuais de concordância e o IVC das avaliações, evidenciando que o instrumento foi validado na primeira rodada Delphi de avaliação pelos especialistas, pois obteve média de nível de concordância 97,2% e IVC médio igual a 1,17.

TABELA 18 – CONCORDÂNCIA ENTRE OS ESPECIALISTAS (N=6) E IVC OBTIDO NO PROCESSO DE VALIDAÇÃO DAS EQUIVALÊNCIAS SEMÂNTICA, IDIOMÁTICA, CONCEITUAL, CULTURAL E CONTEÚDO. CURITIBA–PARANÁ, 2019

Equivalência	Concordam %	Indiferente %	Discordam %	Índice de Validade de Conteúdo (IVC)*
Semântica	83	0	17	0,83
Idiomática	100	0	0	1,33
Conceitual	100	0	0	1,33
Cultural	100	0	0	1,33
Conteúdo	83	0	17	1,00
Média	97,2	-	-	1,17

FONTE: A autora (2019).
* IVC - Para consenso entre os especialistas recomeda-se os valores entre 0,5 e 0,8 (CASTRO; RESENDE, 2009).

As sugestões e avaliações qualitativas dos especialistas foram analisadas pelas pesquisadoras, e os itens considerados relevantes foram acatados e modificados no instrumento (APÊNDICE 6).

Para análise da confiabilidade das avaliações dos especialistas empregou-se o cálculo do coeficiente alfa de *Cronbach* para análise de cada *trigger* cirúrgico, suas respectivas orientações de uso durante as revisões retrospectivas de prontuários, e equivalências (Semântica, Idiomática, Conceitual, Cultural e de Conteúdo). O alfa de *Cronbach* apresentou valor geral de 0,83 para os 11 *triggers* cirúrgicos e 0,84 para as orientações, o que corresponde a nível alto de consistência interna. As avaliações dos especialistas, quanto às cinco equivalências analisadas, demonstraram que o alfa de *Cronbach* apresentou valor geral de 0,77; tal resultado representa bom nível de consistência interna do instrumento.

Na quinta etapa do processo metodológico a versão **V7** foi submetida a pré-teste durante análise retrospectiva de prontuários de pacientes cirúrgicos pelos investigadores. Dentre os 244 prontuários analisados, foram rastreados 40 pacientes com potenciais Eventos Adversos (pEA) e em 31 casos houve a confirmação diagnóstica da ocorrência dos EA cirúrgicos. Verificou-se a ocorrência de no mínimo um até no máximo 09 EA cirúrgicos no mesmo paciente. A versão V7 foi considerada de fácil compreensão e uso pelos investigadores. Deste modo, foi constatado sua aplicabilidade prática durante as análises dos prontuários para detecção de rastreadores positivos (*triggers*) na identificação da ocorrência de eventos cirúrgicos.

O QUADRO 6 apresenta a versão final traduzida, adaptada transculturalmente e validada do módulo cirúrgico do GTT do *Institute of Helthcare Improvement* para uso no contexto brasileiro.

QUADRO 6 – MÓDULO CIRÚRGICO *GLOBAL TRIGGER TOOL* TRADUZIDO E ADAPTADO PARA O PORTUGUÊS DO BRASIL PARA MENSURAÇÃO DE EVENTOS ADVERSOS.

**Módulo Cirúrgico Global Trigger Tool do Institute for Healthcare Improvement para Mensuração de Eventos Adversos**
**S1**- Retorno à sala cirúrgica.
**S2-** Mudança no procedimento.
**S3-** Admissão na Unidade de Terapia Intensiva no pós-operatório.
**S4-** Intubação ou reintubação ou uso de BiPap* na Unidade de Recuperação Pós-Anestésica.<br>*O termo BiPap significa *Bilevel Positive Pressure Airway* (equipamento de ventilação não invasiva que oferece dois níveis de pressão inspiratória e expiratória, administrado por intermédio de máscara nasal ou facial).
**S5-** Raio X Intraoperatório ou em Unidade de Recuperação Pós-Anestésica.
**S6-** Óbito intra ou pós-operatório.
**S7-** Ventilação mecânica superior a 24 Horas no pós-operatório.
**S8-** Administração intra-operatória de Epinefrina, Norepinefrina, Naloxona ou Romazicon. Esses medicamentos não são administrados rotineiramente no intra-operatório.
**S9-** Aumento nos níveis de troponina superior a 1,5 ng/ml no pós-operatório.
**S10-** Lesão, reparo ou remoção de órgão durante o procedimento cirúrgico.
**S11-** Ocorrência de qualquer complicação cirúrgica.

FONTE: A autora (2019).
Adapted from the Institute for Healthcare Improvement Global Trigger Tool for Measuring Adverse Events. http://www.ihi.org/resources/Pages/IHIWhitePapers/IHIGlobalTriggerToolWhitePaper.aspx

O QUADRO 7 apresenta as orientações sobre os *triggers* que estão disponíveis no Manual do *Institute for Healthcare Improvement Global Trigger Tool for Measuring Adverse Events* publicado por Griffin e Resar (2009) e que foram traduzidos, adaptados transculturalmente e validados para uso no contexto brasileiro.

QUADRO 7 – ORIENTAÇÕES DO MÓDULO CIRÚRGICO *GLOBAL TRIGGER TOOL* PARA O PORTUGUÊS DO BRASIL PARA MENSURAÇÃO DE EVENTOS ADVERSOS.

**Orientações sobre os *Triggers* Cirúrgicos**
**S1 Orientação** - O retorno à sala cirúrgica pode ser planejado ou não planejado, e ambos podem ser resultados de evento adverso. Exemplo de evento adverso seria paciente que teve hemorragia interna após a primeira cirurgia e necessitou de segunda cirurgia para explorar a causa e cessar o sangramento. Mesmo que a segunda cirurgia seja exploratória e não indique intercorrência, isto deveria ser considerado evento adverso.
**S2 Orientação** - Quando o procedimento registrado nas anotações pós-operatórias for diferente em relação ao planejado nas anotações ou documentos pré-operatórios, constantes no consentimento cirúrgico, o revisor deve procurar por detalhes que justifiquem a alteração. Mudança inesperada no procedimento devido a complicações ou falha no dispositivo ou equipamento deve ser considerada evento adverso, particularmente se houver aumento no tempo de permanência ou ocorrência de lesão evidente.
**S3 Orientação** - A admissão em Unidade de Terapia Intensiva pode ser indicação pós-operatória normal ou pode ser inesperada. As internações inesperadas frequentemente estão relacionadas às ocorrências de eventos adversos cirúrgicos. Por exemplo, a admissão na Unidade de Terapia Intensiva após o reparo de aneurisma aórtico pode ser esperada, mas a admissão após artroplastia de joelho seria incomum. O revisor precisa verificar o motivo da internação na Unidade de Terapia Intensiva.
**S4 Orientação** - Anestésicos, sedativos ou medicamentos para dor podem resultar em depressão respiratória, exigindo o uso de BiPap* ou reintubação pós-operatória, o que seria evento adverso.<br>*O termo BiPap significa *Bilevel Positive Pressure Airway* (equipamento de ventilação não invasiva que oferece dois níveis de pressão inspiratória e expiratória, administrado por intermédio de máscara nasal ou facial).
**S5 Orientação** - Qualquer imagem que não seja rotineira ao procedimento requer investigação. Raio-x realizado devido à suspeita de itens retidos ou contagem incorreta de instrumento ou compressa é considerado gatilho positivo. A identificação de item retido que necessite novo procedimento é considerada evento adverso. Se o item retido for identificado e removido, sem qualquer evidência adicional de dano ou reoperação do paciente, não se considera evento adverso.
**S6 Orientação** - Todas os óbitos que ocorrem no intra-operatório são considerados eventos adversos, a menos que o óbito seja claramente esperado e a cirurgia tenha sido de indicação extrema. Óbitos pós-operatórios exigem revisão nos registros em busca de especificidades, mas em geral todos serão considerados eventos adversos.
**S7 Orientação** - A ventilação mecânica de curto prazo está planejada no pós-operatório de procedimentos cardíacos, torácicos grandes e certos procedimentos abdominais. Se o paciente necessitar de ventilação mecânica superior a 24 horas, um evento adverso intra-operatório ou pós-operatório deve ser considerado. Pacientes com doença pulmonar ou muscular preexistente podem ter mais dificuldade para sair rapidamente da ventilação mecânica no pós-operatório, mas isso não deve excluir automaticamente a possibilidade de ser evento adverso. Os revisores devem usar o julgamento clínico para determinar se os cuidados intra-operatórios e pós-operatórios eram necessários ou parte do processo da doença.
**S8 Orientação** - Revisar anotações da anestesia e da cirurgia para determinar o motivo da administração. Hipotensão causada por sangramento ou sedação excessiva são exemplos de eventos adversos que podem ser tratados com estes medicamentos.
**S9 Orientação** - Aumento nos níveis de troponina no pós-operatório pode indicar evento cardíaco. Revisores precisarão utilizar julgamento clínico para determinar se um evento cardíaco ocorreu.
**S10 Orientação** - Revisar as anotações cirúrgicas e pós-operatórias em busca de evidências de que o procedimento incluía reparo ou remoção de algum órgão. A remoção ou reparo deve fazer parte de procedimento planejado ou este é evento adverso e, provavelmente, resultado de intercorrência cirúrgica como lesão acidental.
**S11 Orientação** - Trata-se de qualquer uma entre várias complicações, incluindo, mas não se limitando, a Embolia Pulmonar, Trombose Venosa Profunda, Lesão por Pressão, Isquemia do Miocárdio, Falência Renal, etc.

FONTE: A autora (2019).
Adapted from the *Institute for Healthcare Improvement Global Trigger Tool for Measuring Adverse Events*. http://www.ihi.org/resources/Pages/IHIWhitePapers/IHIGlobalTriggerToolWhitePaper.aspx

6 DISCUSSÃO

6.1 CULTURA DE SEGURANÇA

Os resultados desta pesquisa mostram a percepção dos profissionais de saúde sobre as dimensões da cultura de segurança como frágeis, na maioria das respostas, bem como, indicam as lacunas e fatores que podem contribuir para a melhoria da qualidade dos processos de trabalho e SP em unidades de internação cirúrgicas e centros cirúrgicos de três hospitais brasileiros.

Pesquisas, estruturas e ferramentas de avaliação foram desenvolvidas para auxiliar as organizações de saúde a mensurar e compreender o tipo de cultura existente, e os problemas que dificultam os esforços de melhoria (MORELLO; LOWTHIAN; BARKER et al., 2013). A cultura de segurança deve ser avaliada e compreendida antes da execução de qualquer ação, pois tal avaliação fornece indicador concreto do estado atual da cultura na instituição, bem como, permite o acompanhamento da sua evolução (CARVALHO; ARRUDA; NASCIMENTO, 2017).

Em relação aos dados profissionais e sóciodemográficos dos participantes desta pesquisa, verificou-se que quase a totalidade tinha contato direto com o paciente. E, deste modo, no processo em busca da melhoria da qualidade da assistência, a implantação de estratégias para a segurança envolve os profissionais que prestam cuidado direto ao paciente (REIS; HAYAKAWA; MURASSAKI et al., 2017) Tais colaboradores precisam ser, continuamente, incentivados para a manuten-

ção ou melhoria da própria cultura de segurança (KAWAMOTO; OLIVEIRA; TONIN et al., 2016), ressaltando-se a relevância de pesquisas nesta modalidade.

Quanto ao tempo de atuação dos profissionais nos Hospitais B e C, a maioria possui de um a cinco anos. Estes resultados divergem dos verificados no Hospital A, onde prevaleceu o tempo de 21 anos, ou mais, de vínculo empregatício. Ao analisar os dados pode-se valorar o elevado percentual de respostas negativas e neutras verificadas na avaliação da cultura de segurança dos três hospitais, destacadamente no Hospital A. Tal fato pode ser explicado porque profissionais mais experientes sentem-se mais à vontade para revelar suas verdadeiras percepções, por se sentirem mais seguros para detectar riscos, ou pelo tempo de trabalho que pode lhes conferir maior autonomia ao opinar.

Quando a instituição não sofre as consequências das altas taxas de substituição de pessoal, a estabilidade pode contribuir para mudanças no trabalho; afirmativa de estudo que indicou 60% do total de profissionais com mais de cinco anos de atuação (CARVALHO; GOTTEMS; PIRES et al., 2015).

Semelhantemente a esta pesquisa, estudo brasileiro que avaliou a cultura de segurança de três hospitais de ensino do Estado do Ceará mostrou que os profissionais com menos de seis meses de atuação responderam mais positivamente quando comparados aos profissionais com tempo de atuação superior. Os autores concluíram que aqueles que possuem menos de seis meses na instituição estão em processo de adaptação ao ambiente de trabalho e têm percepções mais positivas. Ao passo que os profissionais com mais tempo de atuação percebem melhor as competências individuais e coletivas quanto às questões de segurança (CARVALHO; ARRUDA; NASCIMENTO et al, 2017).

Dados de outro estudo desenvolvido na capital da Arábia Saudita também corroboram com os dados desta pesquisa; o longo tempo de experiência dos profissionais mostrou associação, progressivamente menor, sobre a SP, ou seja, o maior tempo de serviço resultou em menor percentual de respostas positivas (BONDEVIK; HOFOSS; HANSEN et al., 2014).

Nos três cenários avaliados nesta pesquisa constatou-se prevalência de áreas frágeis na cultura de segurança, ou seja, não foi alcançado o escore geral positivo ≥75%, tal como estabelecido pela AHRQ (SORRA; GRAY; STREAGLE et al., 2016).

Esse dado é semelhante a diversos estudos brasileiros realizados em hospitais públicos e/ou universitários, os quais expressaram escores de cultura inferior

ao padronizado pela referida agência norte-americana, nas Regiões Nordeste (ANDRADE; LOPES; SOUZA FILHO et al., 2018; TAVARES; MOURA; AVELINO et al., 2018), Centro-oeste, (CARVALHO; GOTTEMS; PIRES et al., 2015) Sudeste, (MARINHO; RADUNZ; BARBOSA, 2014) e Sul (CORREGGIO; AMANTE; BARBOSA, 2014; CRUZ; ROCHA; MAURICIO et al., 2018).

Semelhantemente, resultados nos quais o escore positivo não foi alcançado foram identificados nos Estados Unidos da América (HEFNER; HILLIGOSS; KNUPP et al., 2017), Irã (KIAEI; ZIAEE; MOHEBBIFAR et al., 2016), Portugal (EIRAS; ESCOVAL; GRILLO et al., 2014) , Eslovênia (ROBIDA, 2013), Alemanha, (GAMBASHIDZE; HAMMER; BROSTERHAUS et al., 2017), Peru (ARRIETA; SUÁREZ; HAKIM, 2017), Tunísia (MALLOULI; TLILI; AOUICHA et al., 2017) e Etiópia. (MEKONNEN; MCLACHLAN; BRIEN et al., 2017).

Estudo de revisão sistemática que englobou 21 países, com diferentes estágios de desenvolvimento, demonstrou que as culturas organizacionais hospitalares requerem melhorias, pois se apresentam subdesenvolvidas ou frágeis (REIS; PAIVA; SOUZA, 2018). Verifica-se que estudos realizados na perspectiva das unidades hospitalares, ou por categorias profissionais em hospitais, e que utilizaram o HSOPSC, serviram de base para a construção de medidas de intervenção em dimensões classificadas como fragilizadas (TOMAZONI; ROCHA; SOUZA et al., 2014; TOMAZONI; ROCHA; KUSAHARA et al., 2015). Esses estudos reiteram a importância dessa investigação para subsidiar ações direcionadas ao contexto institucional e, sobretudo, às lacunas identificadas na cultura de segurança.

Ressalta-se que o índice estabelecido para reconhecer a cultura fortalecida (≥75%), possivelmente, está acima da realidade brasileira e de outros países, pois a diversidade demonstra resultados de cultura de segurança frágil. Contudo, permite o reconhecimento da urgente necessidade de implantação de ações fortalecedoras da cultura de segurança. E, eventualmente, aponta a necessidade de rever o escore estabelecido como necessário para o reconhecimento de cultura de segurança positiva.

A partir destas informações iniciais, descrevemos a avaliação realizada pelos profissionais de saúde em relação às 12 dimensões da cultura de segurança que compõem o referido instrumento de coleta de dados.

A análise da dimensão **Trabalho em equipe dentro das unidades** identifica a existência de trabalho em equipe nas unidades, com apoio e respeito mútuo (REIS; LAGUARDIA; VASCONCELOS et al., 2016). No Hospital B, os dados mostram que esta foi a única dimensão fortalecida da pesquisa, com 77% de respostas positivas ($p<0{,}001$). Os profissionais deste hospital também demonstram percepção menos negativa em seis dimensões de cultura de segurança ($p<0{,}001$).

Deste modo, o Hospital B (gestão privada) foi o único a apresentar cultura de segurança forte nesta dimensão e, o maior índice de respostas positivas indica a existência de clima de trabalho cordial, com respeito às diferentes opiniões. Infere-se que os profissionais trabalham com tranquilidade e promovem o fortalecimento das ações para a segurança do paciente cirúrgico.

Assim como aqui apresentado, estudo brasileiro realizado no Rio Grande do Norte, que analisou a cultura de segurança do paciente em hospitais com diferentes tipos de gestão, verificou que o hospital privado alcançou níveis de fortaleza (≥75%) em cinco dimensões (ANDRADE; LOPES; SOUZA FILHO et al., 2018). Resultados positivos semelhantes foram verificados em outro estudo brasileiro realizado no Ceará (TAVARES; MOURA; AVELINO et al., 2018).

Investigações internacionais também registraram cultura fortalecida nesta dimensão. Na Argentina, pesquisa realizada em hospital privado atingiu 79,4% de respostas positivas e, os autores descrevem a existência de tendência ou vontade dos profissionais para oferecer o melhor para os pacientes, por meio do trabalho em equipe, até mesmo quando as condições de trabalho não são adequadas (RAMOS; COCA; ABELDANO, 2017). Estudo desenvolvido no Peru obteve 76% de respostas positivas para esta dimensão e diferenças significativas em oito dimensões, quando comparados o setor privado e público (ARRIETA; SUÀREZ; HAKIM, 2017). Dados semelhantes foram obtidos em investigação realizada na Jordânia (KHATER; AKHU-ZAHEYA; AL-MAHASNEH et al., 2015).

Pesquisadores defendem ser de extrema importância permitir que os profissionais de saúde contribuam com ideias e sugestões, e atuem de forma proativa para o fortalecimento da cultura de segurança (MINUZZ; SALUM; LOCKS, 2016). Em consonância, demais pesquisadores defendem que o trabalho em equipe é importante estratégia para a prevenção de erros em saúde (NOORD; WAGNER; DYCK et al., 2014).

Infere-se que os profissionais, quando se sentem respeitados no seu local de atuação, tornam-se mais propensos a realizar o trabalho em equipe, ou seja, com cooperação mútua para executar o cuidado seguro. Deste modo, ressalta-se a importância da avaliação sistemática da cultura de segurança nas instituições de saúde (CARVALHO; GÖTTEMS; PIRES et al., 2015).

Em relação aos três hospitais participantes, verifica-se que a dimensão Trabalho em equipe dentro das unidades apresentou classificação de cultura de segurança fortalecida no Hospital B e adequada nos Hospitais A e C. Equipes que trabalham de maneira cooperativa, bem coordenada e com canal de comunicação adequado, previnem situações de descontinuidade no cuidado e de alto risco para os pacientes (CARVALHO; GÖTTEMS; PIRES et al., 2015). Os hospitais investigados demonstram trabalhar em equipe e priorizam a realização de assistência segura. Diante deste fato, destaca-se a importância do incentivo de ações fortalecedoras e mantenedoras desta importante área da cultura de segurança.

Na dimensão **Expectativas sobre seu supervisor/chefe - ações promotoras de segurança**, bem como, na dimensão **Aprendizado organizacional - melhoria contínua** observa-se escores mais elevados de respostas positivas, e com significância estatística nos três hospitais. Apesar dos escores serem classificados como adequados, ainda não estão fortalecidos, porém, apresentam potencial para se tornarem áreas fortes da cultura de segurança. Os resultados indicam que estes profissionais reconhecem que suas sugestões e opiniões para a promoção da SP são respeitadas e consideradas pela liderança. Bem como, esses percebem que os erros comunicados podem proporcionar mudanças positivas para a melhoria contínua dos processos de trabalho nas unidades de internação cirúrgicas e centros cirúrgicos.

As dimensões supracitadas apresentaram respostas neutras, ou seja, percentual de respostas positivas superior à 51% e inferior à 74%. Neste contexto, as dimensões da cultura de segurança são consideradas como adequadas, com potencial de melhorias (AHRQ, 2017). Tais parâmetros foram evidenciados em outro estudo brasileiro, realizado em hospital de ensino no Estado de São Paulo que, semelhantemente, apresentou melhores resultados na dimensão Expectativas sobre seu supervisor/chefe - ações promotoras de segurança, e na dimensão Aprendizado

organizacional - melhoria contínua. Os pesquisadores analisaram que a maioria dos participantes acreditava que suas sugestões eram consideradas pelo supervisor/chefe em prol da melhoria da qualidade, bem como, revelaram a existência da cultura de aprendizado na instituição (SILVA-BATALHA; MELLEIRO, 2015).

Deste modo, destaca-se a postura das lideranças frente aos erros, com efetiva demonstração e correspondentes ações da importância atribuída às ocorrências, e busca de soluções preventivas, com a inclusão da equipe. Pois, afinal, os prestadores de cuidado direto têm o contexto assistencial cotidiano como base para a análise dos fatores contributivos ao erro e, também, das necessidades e ajustes à prática assistencial. As lideranças possuem, possivelmente, a visão geral da instituição, limites e possibilidades. Esse conjunto tem potencial para o planejamento de ações realísticas, aplicáveis e com potencial de mudanças e preenchimento das lacunas identificadas coletivamente.

Sobre a dimensão relacionada à **Frequência de relatos de eventos**, observa-se que as respostas negativas e neutras foram prevalentes nos Hospitais A e B, o que revela cultura fragilizada. Apenas o Hospital C apresentou escore de respostas positivas adequadas, porém com necessidade de melhorias. Destaca-se que a notificação de erros contribui para o aprimoramento de práticas seguras e, a análise destas falhas produz aprendizado para todos os profissionais envolvidos, assim como, para a instituição em geral (WANG; LIU; YOU et al., 2014). Ou seja, o relato de eventos oportuniza estudo e compreensão de fatores colaborativos ao erro e, consequentemente, para o planejamento de ações direcionadas à prevenção.

Portanto, essas constituem estratégias que permitem a alteração dos fatores contribuintes para o erro e dificultam a reincidência (MARINE; RADÜNZ; TOURINHO et al., 2016). Neste contexto, autores descrevem a importância da equipe valorizar também os relatos dos pacientes, por permitirem a detecção de erros, e a implementação de medidas corretivas, além de tornar estes erros preveníveis no futuro (CORREIA; MARTINS; FORTE, 2017).

Nesta pesquisa, o maior percentual de respostas negativas foi identificado na dimensão **Respostas não punitivas ao erro**, que investiga se funcionários sentem que seus erros e relatórios de erros podem ser usados contra eles e mantidos em seu arquivo pessoal.

Diversos estudos apresentaram cultura organizacional punitiva frente aos erros no mundo (WANG; LIU; YOU et al., 2014; GÖZLÜ; KAYA, 2016; AHRQ, 2017; ELMONTSRI; ALMASHRAFI; BANARSEE et al., 2017; ALI; IBRAHEM; AL MUDAF et al., 2018), incluindo no Brasil (SANTIAGO; TURRINI 2015; SOUZA; KAWAMOTO; OLIVEIRA et al., 2015; MATIELLO; LIMA; COELHO et al., 2016; ANDRADE; MELO; SILVA et al., 2017; CRUZ; ROCHA; MAURÍCIO et al., 2018). Tais resultados são preocupantes e demonstram o desencorajamento dos profissionais em relatar e notificar os erros.

Revisão sistemática sobre cultura de segurança do paciente nos países árabes mostrou que os profissionais de saúde acreditam ainda existir cultura de culpa e, por esta razão, este sério problema precisa de estratégias para ser aprimorado. Os autores reforçam a necessidade de políticas públicas para o desenvolvimento de sistemas de notificação com vistas à identificação de riscos aos pacientes, e para que as organizações e os profissionais aprendam com os erros (ELMONTSRI; ALMASHRAFI; BANARSEE et al., 2017). Desafios para a resolução dos problemas relacionados à resposta não punitiva ao erro também foram evidenciados nos hospitais suecos (DANIELSSON; NILSEN; RUTBERG et al., 2017) e em áreas cirúrgicas na Tunísia (MALLOULI; TLILI; AOUICHA et al., 2017) e no Irã (RAEISSI; REISI; NASIRIPOUR, 2018).

Os erros existem e devem ser considerados como parte da condição inevitável do ser humano. No entanto, é fundamental a criação de todas as condições possíveis para minimizar sua incidência e risco. A culpa da ocorrência não deve ser designada às pessoas somente, mas como falhas no processo de trabalho dos sistemas de saúde (VINAGRE; MARQUES, 2018). Pois, esses ocorrem devido às falhas sequenciais do sistema, e não por fatores isolados, como o descuido do profissional de saúde (TOMAZONI; ROCHA; KUSAHARA et al., 2015).

A cultura de segurança positiva na resposta não punitiva ao erro contribui para maior notificação e tratamento adequados das ocorrências (DUARTE; STIPP; SILVA et al., 2015), além de ser crucial para a correção das falhas e prevenção de EA, com o objetivo de aperfeiçoar a assistência (VINAGRE; MARQUES, 2018). No contexto das unidades cirúrgicas, estudo descreve que a subnotificação de EA é fator que precisa ser desenvolvido com a finalidade de estimular os relatos e a criação de ambientes não punitivos. (BOHOMOL; MELO, 2019). Ressalta-se contudo, a baixa confiabilidade alcançada nesta dimensão (alfa de Conbach -

Hospital A=0,10; Hospital B=0,70 e Hospital C=0,56), que pode mostrar incoerência entre as respostas e fator a ser considerado na análise dos resultados.

Ancorada na prática assistencial, é possível afirmar que, sobretudo, no ambiente da sala cirúrgica, os erros são frequentes e, reiteradamente, ignorados ou julgados como parte do risco cirúrgico. Em hospitais de ensino, pode-se apontar como fatores de prejuízo ao paciente desde o ambiente de aprendizagem, no qual o estudante não possui domínio técnico ainda, até a ausência do professor em tempo integral. Deste modo, destaca-se a relevância desta cultura "permissiva" ser transformada e, progressivamente, o ambiente cirúrgico transformado, no qual a equipe multiprofissional reconheça os erros como evitáveis e notificáveis. Essa transformação fará com que as ocorrências, em especial em sala cirúrgica, não sejam omitidas, mas contribuam para mudanças em prol da qualidade da segurança assistencial.

As demais dimensões da cultura de segurança, nominadas **Apoio da gestão para a segurança do paciente, Percepção geral da segurança do paciente, Retorno da informação e comunicação sobre o erro, Adequação de profissionais** e **Passagem de plantão/turnos e transferências** apresentaram-se fragilizadas nos três hospitais, o que mostra áreas a serem trabalhadas para alcançar melhorias na segurança dos pacientes cirúrgicos. Contudo, a confiabilidade testada pelo alfa de Cronbach foi variável entre os hospitais (entre pobre e aceitável), com exceção à dimensão **Adequação de profissionais**, que foi inaceitável (<0,50). A seguir, cada uma destas dimensões é contextualizada.

Quanto à dimensão **Apoio da gestão para a segurança do paciente**, as respostas negativas e neutras prevaleceram nos três hospitais avaliados. As respostas mostram que a percepção é de que a gestão hospitalar não está promovendo a SP como fator relevante e primordial. A análise das respostas às três perguntas que fazem parte desta dimensão também demostra que a alta gestão das organizações não proporciona os meios e estratégias favorecedoras da cultura de SP. Tal fato serve de alerta e deve ser considerado, afinal foi verificado nos três hospitais avaliados, mesmo em detrimento das diferentes formas de gestão (federal, privado e estadual).

Os dados são preocupantes, pois os hospitais avaliados nesta pesquisa também são classificados como Hospitais de Ensino. Esta classificação indica que

esses pertencem, ou são conveniados, à Instituição de Ensino Superior; e oferecem campo para a prática do ensino, com programas de residência multiprofissional em saúde (BRASIL, 2015). Deste modo, essas instituições têm responsabilidades cruciais na formação profissional e são formadores de opinião e modelo de comportamento para distintas categorias, por isso devem contribuir para o ensino dos conceitos, processos e práticas seguras em saúde.

Além do mais, o fato da alta gestão não dar a devida prioridade à SP, de acordo com a percepção dos participantes, pode significar descaso para com os que necessitam de assistência cirúrgica especializada. Afinal, os pacientes/usuários estão expostos à inúmeros riscos e ocorrência de EA devido ao grau de complexidade que este tipo de assistência exige. Torna-se importante evidenciar que os três hospitais possuem Núcleo de Segurança do Paciente e profissionais capacitados que atuam no gerenciamento de riscos. Ou seja, são instituições que observam a legislação quanto aos preceitos estruturais do tema em questão.

Contudo, as referidas análises possibilitam a compreensão de que a adoção de práticas seguras está associada à gestão dos riscos, e esta estratégia deve compreender a revisão frequente dos processos de trabalho, com vistas à prevenção de incidentes relacionados à assistência à saúde, instituição de barreiras de segurança e avaliação frequente das práticas de melhorias adotadas (BRASIL, 2016).

O estabelecimento da cultura de segurança positiva depende, diretamente, das ações da alta direção, pois a falta de prioridade na área da SP pode influenciar, negativamente, os profissionais que buscam a melhoria da qualidade dos atendimentos. Pois, a partir do momento que percebem que não possuem poder de decisão, suas ações podem se tornar limitadas (KAWAMOTO; OLIVEIRA; TONINI et al., 2016). Neste sentido, a implantação de medidas para o fortalecimento da SP deve ser prioridade de todos os profissionais, em especial daqueles que possuem cargos que estão no topo do organograma, para que os resultados positivos sejam evidenciados nas instituições de saúde (COSTA; RAMOS; GABRIEL et al., 2018).

Os riscos relacionados aos cuidados à saúde são elevados nas organizações hospitalares e, diversos estudos nacionais e internacionais corroboram com os resultados aqui apresentados, pois demonstram que o apoio da alta gestão em prol da SP apresenta falhas a serem corrigidas (BRASIL, 2013b; LAUS; MENEGUETI; SANTOS et al., 2014; SILVA-BATALHA; MELLEIRO, 2015;

KAWAMOTO; OLIVEIRA; TONINI et al., 2016; PINHEIRO; SILVA JUNIOR, 2017; ARRIETA; SUÁREZ; HAKIM, 2017).

Outra dimensão que se apresenta fragilizada nos três hospitais é a **Percepção geral sobre a segurança do paciente**. Esta avalia o grau de notificação de erros, sejam estes detectados antes de afetar o paciente, sem a possibilidade de causar danos ou que poderiam, mas não prejudicaram o paciente (AHRQ, 2017). As adequações necessárias para o fortalecimento desta dimensão envolvem o desenvolvimento de procedimentos, sistemas e protocolos para prevenção de erros, e melhoria contínua dos cuidados e segurança dos pacientes cirúrgicos. As ferramentas informatizadas são consideradas importantes estratégias para a promoção da qualidade e segurança do cuidado nas instituições de saúde (GOMES; ASSIS; FERREIRA et al., 2017). Assim como, a utilização de protocolos para à aplicação de *checklists*, que são efetivos para a identificação de incidentes e essenciais para alcançar melhorias na assistência à saúde (SALVADOR; COSTA; GOMES et al., 2017).

Estudo realizado na Índia demonstrou que a implementação de *checklist* adaptado sobre a segurança cirúrgica teve associação com a redução da mortalidade e de complicações (CHAUDHARY; VARMA; KAPOOR et al., 2015). Demais estudos confirmam a eficácia destas ferramentas de segurança na minimização de complicações pós-operatórias (MAYER; SEVDALIS; ROUT et al., 2016; HAUGEN; WAEHLE; ALMELAND et al., 2019).

Apesar dos esforços e recomendações da OMS (OMS, 2009), observa-se que na prática não há efetiva adesão dos profissionais ao uso eficaz dos *checklists* de segurança; estudos demonstram a ocorrência de EA devido ao não cumprimento do protocolo da Cirurgia Segura (CABRAL; EGGENBERGER; KELLER et al., 2016; BEKS; CLAESSEN; OH et al., 2016; REED; GANYANI; KING et al., 2016). Importante refletir que, na prática cirúrgica, a equipe não adere ao protocolo devido à falha de divulgação, ou pela falta da percepção dos profissionais em relação à importância do *checklist* de segurança. Neste sentido, para que o protocolo seja implementado, adequadamente e de forma homogênea, o conhecimento da equipe cirúrgica é essencial (FREITAS; ANTUNES; LOPES et al., 2014; SANTANA; FREITAS; FERRAZ et al., 2016).

Contraponto às avaliações positivas sobre o Aprendizado organizacional - melhoria contínua, os profissionais avaliaram de maneira significativamente negativa

a dimensão referente ao **Retorno da informação e comunicação sobre o erro**, conforme demonstrado nos resultados com significância estatística na associação entre os três hospitais. As respostas negativas e neutras demonstram que os EA são divulgados satisfatoriamente nas instituições analisadas, e que o retorno da informação e comunicação sobre o erro ocorrem raramente para os profissionais.

Ser recompensado por relatar algum problema de SP incentiva o desenvolvimento da comunicação entre os profissionais. A implementação de ações que garantam o desenvolvimento de cultura de segurança positiva deve focar no incentivo da notificação interna e discussão dos erros (SANTIAGO; TURRINE, 2015). Além da promoção do *feedback* aos profissionais que prestam cuidados diretos de saúde, pois são consideradas estratégias fundamentais para a cultura de segurança eficaz e prevenção de recorrências de erros (MINUZZI; SALUM; LOCKS, 2016). Nesta pesquisa, os dados apontam que, além da deficiência no *feedback* aos profissionais sobre as mudanças implementadas após a ocorrência dos erros, existem falhas que precisam ser corrigidas por meio de estratégias, como discussão acerca de maneiras para evitar os erros na assistência cirúrgica.

Mudanças culturais positivas foram conquistadas quando houve melhoria nos mecanismos de retorno da informação sobre os eventos e sobre a comunicação de melhorias efetuadas em decorrência desses. Pois, o foco na melhoria da resposta ao erro é crucial para a adequação das notificações (BURLISON; QUILLIVAN; KATH et al., 2016). Portanto, as evidências demonstram que o retorno das informações sobre o erro pode estimular, positivamente, a cultura de segurança do paciente, desde que os resultados sejam adaptados para cada realidade e compreensíveis para os profissionais responsáveis pelo atendimento nos diversos departamentos das organizações de saúde (ZWIJNENBERG; HENDRIKS; HOOGERVORST-SCHILP et al., 2016).

Quanto à dimensão **Adequação de profissionais**, a análise dos escores revela fragilidades nos três hospitais analisados, apesar destes apresentarem diferentes tipos de gestão. Verifica-se que este fato é uma constante no Brasil e em vários lugares do mundo. A percepção dos respondentes desta pesquisa, em relação ao fato de não haver quantitativo de profissionais suficientes para desenvolver o trabalho, foram observadas conforme porcentagens semelhantes em diversos estudos (EL-JARDALI; SHEIKH; GARCIA et al., 2014; KHATER; AKHU-ZAHEYA; AL-MAHASNEH et al., 2015; ALSWAT; ABDALLA; TITI et al., 2017;

NAJJAR; NAFOURI; VANHAECHT et al., 2015). Contudo, a confiabilidade foi inaceitável, podendo inferir discurso retório de falta de profissionais.

Na Espanha, estudo demonstrou que os principais pontos fracos para a cultura de segurança foram insatisfação no trabalho por sobrecarga, condições inadequadas de trabalho e alta jornada (GAMA; OLIVEIRA; HERNANDÉZ, 2013). Estudo palestino em hospitais públicos, para avaliar as mudanças na cultura de segurança entre 2011 e 2016, após implementações de medidas em prol da SP, verificou efetividade geral positiva em várias dimensões. No entanto, os resultados demonstraram que houve diminuição significativa nas respostas positivas quanto ao dimensionamento de pessoal (HAMDAN; SALEEM; 2018)

No Brasil, investigação em unidade de internação cirúrgica de hospital universitário, no sul do país, inferiu que a ocorrência de EA está relacionada a inúmeros fatores, dentre eles, o quantitativo de profissionais (SELL, AMANTE; MARTINS et al., 2018). No ambiente de centro cirúrgico, fatores institucionais e organizacionais limitantes, como recursos humanos e materiais, podem contribuir para atrasos recorrentes no início das cirurgias, item que foi destacado como motivo de conflitos entre as equipes médica e de enfermagem (CAUDURO; VALLE; CRUZ, 2017)

Outro estudo demonstrou que a grande maioria dos médicos e enfermeiros entrevistados acreditam que a sobrecarga de trabalho tem associação com a ocorrência de EA (CHAKRAVARTY, 2013). Pesquisa que analisou a carga de trabalho da equipe de enfermagem, e sua relação com a SP, descreve que quanto maior o número de pacientes que necessitam de assistência, maior é a ocorrências de quedas, infecções, rotatividade de profissionais e índice de absenteísmo. (MAGALHÃES; DALL’AGNOL; MARCK, 2013).

Na área cirúrgica, investigação realizada em centro cirúrgico brasileiro também demonstrou que a dimensão Adequação de profissionais está fragilizada; os autores observaram a necessidade de treinamento profissional e estratégias de melhorias no dimensionamento da equipe (MARINHO, RANDÜNZ, BARBOSA, 2014). Portanto, os resultados desta pesquisa, e de outros estudos aqui relatados, reforçam a necessidade de os hospitais brasileiros valorizarem seus profissionais e desenvolverem ações para a redução de erros e EA que podem ocorrer em detrimento de cargas de trabalho excessivas e esgotamento dos profissionais.

Os resultados desta pesquisa vão ao encontro dos estudos relatados acima e outros que identificaram essa dimensão como fragilizada, tanto na instituição de modo
geral, como em setores específicos (GÖZLÜ; KAYA, 2016; NAJJAR; BAILLIEN; VANHAECHT et al., 2018; BOHOMOL; MELO, 2019). Mudanças devem ser viabilizadas e implementadas por toda organização hospitalar nos níveis estratégico, administrativo e operacional, em especial para o desenvolvimento de ações que fortaleçam a cultura não punitiva, bem como, a avaliação do dimensionamento de profissionais que realizam assistência ao paciente no período perioperatório. (BOHOMOL; MELO, 2019).

A dimensão analisada sobre a **Passagem de plantão/turnos e transferências** demonstrou ser outra área fragilizada nos três hospitais da pesquisa. Os resultados apontam para falhas na transmissão de informações relacionadas ao tratamento do paciente durante a mudança de turnos e entre as unidades hospitalares. Neste sentido, estudo descreve que o compartilhamento de informação, o relato de incidentes e a adequada documentação garantem a efetividade durante a passagem de plantão (OLIVEIRA; LEITÃO; SILVA et al., 2014). A identificação das necessidades do paciente, e o conhecimento sobre os riscos aos quais os pacientes estão expostos, são medidas que permitem a elaboração de plano de cuidados e intervenções para a segurança da assistência (PEREIRA; ABREU; BONFIM et al., 2017).

Inúmeras pesquisas foram realizadas nos hospitais dos Estados Unidos para avaliação da cultura de segurança, com a utilização do questionário HSOPSC da AHRQ. A partir de dados divulgados destes estudos, em 2010, desenvolveu-se outro para analisar, especificamente, as transferências dos pacientes, cultura e práticas de segurança, pelo método da regressão linear múltipla hierárquica. Os resultados demonstraram que as percepções dos profissionais sobre a SP estão relacionadas com as melhorias nos sistemas de transferências, os quais podem ser fortalecidos com treinamento da equipe multiprofissional e monitoramento da ocorrência de eventos indesejados (LEE; PHAN; DORMAN et al., 2016).

Estudo realizado em 2017, e que comparou dois períodos gerenciais distintos no mesmo Hospital A desta pesquisa, verificou evolução positiva nesta dimensão e reflexo na prática assistencial dos profissionais que atuam no contexto cirúrgico, apesar dos escores nos dois períodos permaneceram baixos.

Os pesquisadores descrevem que este progresso pode ser justificado pelo aumento da força de trabalho e implantação de ferramentas gerencias, como os *checklists* de segurança cirúrgica pré e pós-operatório, para a comunicação da equipe
da unidade de internação cirúrgica e a do centro cirúrgico (ALPENDRE; CRUZ; DYNIEWICZ., et al., 2017). Assim como, em decorrência da elaboração e implementação de diversos formulários para passagem de plantões padronizados nas unidades de internação (BATISTA, 2018).

Nos Hospitais B e C, aqui analisados, não existem formulários e protocolos para passagem de plantão nas unidades de internação cirúrgica, mas os checklists de segurança cirúrgica da OMS estão em processo de implementação nos respectivos centros cirúrgicos. Tais dados revelam iniciativas positivas, pois estudo de revisão sistemática revelou melhorias na transferência de informações, com o uso de checklists cirúrgicos (PUCHER; JOHNSTON; AGGARWAL et al., 2015).

Ao analisar o constructo cultura de segurança em associação com as categorias profissionais (médicos assistentes, médicos residentes e enfermagem), os resultados evidenciam que a percepção das equipes de enfermagem e medicina diferem entre os hospitais. Tal análise se reflete nas respostas fortalecidas na dimensão **Expectativas sobre seu supervisor/chefe** e **Ações promotoras de segurança** que foram informadas pela enfermagem nos três hospitais avaliados. Conforme estudo brasileiro, realizado em hospitais do Ceará, a equipe de enfermagem também apresentou resultados positivos na percepção da cultura de segurança quando comparada com outros profissionais (CARVALHO; ARRUDA; NASCIMENTO et al., 2017).

Na percepção dos enfermeiros, o fato de participar dos processos para implantação das metas de segurança e oferecer assistência segura ao paciente origina sentimentos de satisfação (REIS; HAYAKAWA; MURASSAKI et al., 2017), e a relação positiva entre os profissionais de enfermagem e a chefia é fator de proteção a erros e que promove a qualidade e segurança no cuidado aos pacientes (WONG; LASCHINGER, 2015).

Nesta perspectiva, em comparação aos médicos assistentes e médicos residentes, a percepção mais positiva da enfermagem em relação ao apoio recebido dos seus supervisores e chefes, potencialmente, explica a significância estatística verificada nesta dimensão. Considera-se, também, que a equipe de enfermagem é

proativa quando da discussão de ações gerenciais, elaboração e implantação de protocolos diversos, inclusive relativos à SP. Além desses profissionais assumirem cuidado ininterrupto ao paciente e terem, historicamente, a passagem de plantão como um instrumento gerencial, e que propicia a detecção de não conformidades e da continuidade do cuidado.

As respostas da categoria enfermagem nas dimensões **Aprendizado organizacional - melhoria contínua** e **Frequência de relatos de eventos** também apresentaram maiores escores em comparação aos demais profissionais. Pesquisadores descrevem que diferenças de percepção estão associadas a maior aproximação desta categoria aos diversos aspectos relacionados à SP (TEREANU; SAMPIETRO; SARNATARO et al., 2018).

Em oposição aos resultados desta pesquisa, estudo realizado em hospitais públicos no Kuwait mostraram que os enfermeiros tendem a relatar menos eventos. (ALI; IBRAHEM; AL MUDAF et al., 2018). Importante lembrar, como descrito anteriormente, que os erros costumam ser subnotificados por diversas razões, tais como medo, sentimento de humilhação, cultura punitiva e acompanhamento limitado pós relato de um erro, sem retorno da informação para o aprendizado dos profissionais e da organização.

A avaliação das dimensões da cultura de segurança denominadas **Trabalho em equipe entre as unidades** e **Abertura de comunicação** apresentaram-se frágeis nos três hospitais, com significância estatística, principalmente na percepção dos médicos residentes, que responderam às questões do questionário de forma negativa/neutra em todas as perguntas destas dimensões. Tais resultados corroboram com estudo que mostrou que o estresse, os conflitos entre a equipe multiprofissional e a comunicação deficiente contribuem para a baixa cultura de segurança entre os médicos residentes e médicos assistentes (CAUDURO; VALLE; CRUZ, 2017).

Várias ações podem ser desenvolvidas pelas unidades cirúrgicas para o desenvolvimento da dimensão **Trabalho em equipe** e para implementação de práticas seguras, tais como: fornecer educação profissional desde a formação acadêmica, treinamentos e dinâmicas para o aprimoramento de atitudes e competências (BRASIL, 2013b, KAWAMOTO; OLIVEIRA; TONINI et al., 2016), afinal os médicos residentes relacionam-se com profissionais da equipe multiprofissional e de diversos setores do hospital.

A dimensão **Abertura de comunicação** remete ao fato de que os profissionais da equipe devem ter liberdade para falar e manifestar suas opiniões so bre os aspectos que possam colocar a segurança do paciente em risco (BOHOMOL; MELO, 2109). Baixos escores verificados nesta dimensão merecem atenção, pois a comunicação efetiva é considerada uma das metas internacionais para a SP pela OMS e *Joint Commission International* (JCI), 2013). A comunicação entre os profissionais é fundamental para práticas seguras, principalmente em setores com múltiplos profissionais e desenvolvimento de atividades complexas (CLANTON; CLARK; LOGGINS et al., 2018), como centros cirúrgicos e unidades de internação cirúrgicas.

O cuidado ao paciente cirúrgico se torna fragilizado quando ocorrem falhas na comunicação (CAUDURO; VALLE; CRUZ, 2017). Conforme o Plano Nacional de Segurança do Paciente, a comunicação eficaz da equipe multiprofissional em ambientes cirúrgicos é basilar para evitar suspensões de cirurgias, procedimentos e exames (BRASIL, 2014). Apesar de diversas iniciativas para promover a comunicação nos hospitais, profissionais de saúde de um centro cirúrgico de hospital brasileiro relataram não verificar mudanças positivas na comunicação interpessoal, mesmo após a implantação do *checklist* cirúrgico sugerido pela OMS (PANCIERI; SANTOS; ÁVILA et al., 2013). No Reino Unido, relatório descreveu que a falha na comunicação foi o segundo fator contributivo para incidentes cirúrgicos (MUSHTAQ; O'DRISCOLL; SMITH et al., 2018).

Fragilidades na cultura de segurança quanto à abertura de comunicação para erros e falhas assistenciais também foram identificadas em instituições hospitalares no Perú, em estudo que obteve a participação de 1679 profissionais de saúde e apenas 35% de respostas positivas nesta dimensão (ARRIETA; SUAREZ; HAKIM, 2018). Em investigação realizada na China, esta dimensão apresentou índice significativamente menor de respostas positivas, equivalente a 27,4%, e esteve associada com maior tempo de atuação profissional (ZHAO; LIU; WANG et al., 2017).

Ações de melhorias, com objetivo de promover a abertura de comunicação, são necessárias ao seu fortalecimento, pois esta dimensão é essencial para a SP e englobam ações para sistematização do retorno da informação mediante comunicação do erro e estímulo a comunicação de EA (BATISTA; CRUZ; ALPENDRE et al., 2019). A falta de honestidade e clareza na comunicação entre os

profissionais resulta em entendimento impreciso e maior exposição ao erro. A comunicação aberta resulta em entendimento impreciso e maior exposição ao erro. A comunicação aberta
resulta em cultura de segurança positiva e, por isso, deve ser estratégica e estender-se à equipe multidisciplinar (KUNSCH, 2016).

A dimensão Abertura de comunicação pode ser fortalecida com elaboração de protocolos gerenciais e treinamentos para equipe multiprofissional que atuam na assistência perioperatória, além do fortalecimento no ensino profissional em saúde

Com relação ao Número de eventos notificados nos últimos 12 meses pelas três categorias profissionais (médicos assistentes, médicos residentes e equipe de enfermagem), os resultados mostram predomínio de nenhum evento notificado. Tais resultados corroboram com estudo baiano, no qual a maioria dos profissionais informaram não terem notificado eventos (SILVA; ROSA, 2016).

A notificação de eventos é considerada estratégia essencial na prevenção de erros, conforme demonstrado na maioria dos artigos analisados em estudo de revisão de literatura (VINAGRE; MARQUES, 2018). No entanto, o caminho a percorrer ainda é considerado longo, por isso é vital a criação de cultura de responsabilidade e não de culpa dos profissionais envolvidos (MENDES; GUERREIO; BARROSO, 2014).

Existe a urgente necessidade de construir ambiente de trabalho não punitivo e desenvolvimento de inciativas para relatos voluntários de eventos (WANG; LIU; YOU et al., 2014). A notificação de eventos oportuniza sua investigação, com a identificação dos fatores organizacionais e individuais colaborativos ao erro, modos recorrentes de erro e suas consequências para o paciente e para a organização. Esses elementos contribuem, de modo insubstituível, para fundamentar ações realísticas à prevenção de EA. Deste modo, atrela-se às notificações sua análise sistemática e retorno das informações, de modo genérico, aos notificadores; dimensão que se mostrou fragilizada nos hospitais participantes. Desta forma, melhorias no retorno das informações, potencialmente, contribuirá para alavancar as notificações de EA, formando um círculo virtuoso para a SP.

Sobre os resultados da **Nota de segurança**, atribuída pelos profissionais, verifica-se que a maior porcentagem de respostas foi para nota excelente/muito boa, atribuída pela enfermagem do Hospital A, seguida pela mesma avaliação dos médicos assistentes do Hospital C. Resultado diferente foi verificado no Hospital B,

pois a maior porcentagem de respostas foi para a nota regular, atribuída pelos médicos residentes. Neste contexto, a nota geral de segurança do paciente, independentemente da asso
ciação entre as categorias profissionais, apresentou prevalência de notas excelente/muito boa nos Hospitais A e C e nota regular no Hospital B. Os dados aqui relatados corroboram com estudo brasileiro realizado em três hospitais do Rio Grande do Norte, onde a maioria dos participantes classificaram a SP como boa ou excelente (ANDRADE; LOPES; SOUZA FILHO et al., 2018).

Neste contexto, a nota geral de segurança pode estar relacionada ao perfil de atendimento e/ou condições econômicas dos hospitais participantes desta pesquisa. A nota atribuída pelos profissionais do Hospital B, que avaliaram a segurança da instituição como regular, induz a algumas reflexões. Trata-se de hospital que realiza cirurgias eletivas, mas também apresenta significante demanda para atendimento de pacientes politraumatizados, com ferimentos por arma de fogo/branca, ou complicações clínicas agudizadas em pacientes que necessitam ser submetidos a cirurgias de emergência e urgência. Vale ressaltar que a gravidade dos diagnósticos apresentados pelos pacientes pode interferir na evolução dos resultados, que podem ser satisfatórios, ou não, com risco aumentado para ocorrência de sequelas ou óbitos. Cabe ainda refletir que, no período em que esta pesquisa foi realizada, o referido hospital passava por graves problemas financeiros.

Locais com este perfil de atendimento, e dificuldades financeiras, podem apresentar potencial de riscos aumentados para a realização de cuidados seguros, em detrimento de características como superlotação, alta carga de trabalho, problemas na infraestrutura, falta de recursos materiais e humanos, demora no atendimento e a insatisfação de profissionais e usuários (SCHMIDT; MÜLLER; SANTOS et al., 2014; LIMA; SANTOS; ANDRADE et al., 2015). Estes fatores interferem na qualidade da assistência e aumentam os riscos para ocorrência de erros ou EA.

Em contrapartida, há de se considerar que o Hospital C avaliou a instituição como excelente/muito boa em relação à segurança do paciente; este realiza atendimentos semelhantes ao Hospital B, ou seja, faz cirurgias eletivas conforme agendamento via ambulatorial e cirurgias de urgência/emergência via pronto socorro.

A justificativa sobre a diferença da nota atribuída pelos profissionais de saúde nestes hospitais, e que apresentam as mesmas características de atendimento, pode
estar relacionada ao fato de o Hospital C não apresentar as graves dificuldades financeiras observadas no Hospital B. Quanto ao Hospital A, verifica-se que este não presta atendimento de emergência via pronto socorro, mas trabalha como retaguarda e atende casos de urgência após encaminhamento da Central de Regulação de Leitos após solicitações das Unidades de Pronto Atendimento. Os diversos fatores causais aqui apresentados, em conjunto com as dimensões da cultura de segurança que se apresentaram fragilizadas, podem ter sido determinantes para a nota de segurança atribuída pela equipe.

No entanto, é possível constatar que, de modo geral, as dimensões com maior número de respostas positivas, apresentaram homogeneidade nos resultados em relação às diferentes categorias profissionais (médicos assistentes, médicos residentes e enfermagem). Da mesma maneira, ocorreu similaridade entre as categorias profissionais nas dimensões avaliadas negativamente. Resultados semelhantes foram encontrados em estudo realizado em hospital público no sul do Brasil, que avaliou a percepção de diferentes categorias profissionais sobre a cultura de segurança (MINUZZI; SALUM; LOCKS, 2016), embora a SP não seja problema específico de uma categoria profissional, mas trata-se de processo que engloba transformação institucional (MENDES; GUERREIRO; BARROSO, 2014). Contudo, as categorias profissionais Enfermagem e Medicina tem formação distinta, o que pode influenciar na percepção diversa e que não pode ser, nesta pesquisa, suficientemente esgotada a discussão.

Estudo de revisão de literatura identificou nos 12 artigos analisados e publicados entre 2012 e 2017 a relação direta de cultura de segurança positiva com a redução de EA na assistência à saúde. Deste modo, há necessidade de tornar o sistema mais seguro, ao invés de tentar alterar apenas a condição humana (VINAGRE; MARQUES, 2018).

Para a avaliação das propriedades psicométricas do instrumento de coleta de dados utilizou-se o Índice Alfa de *Cronbach,* que mede a correlação entre as respostas do questionário e baseia-se na correlação média entre as perguntas. Por este motivo, espera-se encontrar variações entre as percepções dos indivíduos, afinal são sujeitos sociais e com diferentes experiências (BOHOMOL; MELO, 2019).

Nesta pesquisa, o cálculo deste coeficiente demonstrou confiabilidade geral satisfatória nos três hospitais participantes. Verificou-se boa consistência interna do questionário no Hospital A e excelente nos Hospitais B e C. O melhor índice foi observado na dimensão “Frequência de relato de eventos”, pois as respostas dos profissionais foram consistentes em todas as perguntas que a compõem.

No entanto, verificou-se inconsistência inaceitável em algumas dimensões do instrumento, com destaque para a dimensão “Adequação de profissionais”, conforme os valores de Alfa de *Cronbach* verificado nos três hospitais participantes: 0,26 (Hospital A), 0,47 (Hospital B) e 0,46 (Hospital C); e na dimensão que analisou “Respostas não punitivas ao erro”, que obteve o menor índice de confiabilidade, com Alfa de *Cronbach,* equivalente à 0,10 no Hospital A.

Os indices apontam para inconsistência entre as respostas às perguntas que compõem a dimensão. Ou seja, colocam em dúvida os respectivos resultados; seja devido às perguntas elaboradas, seja pelas respostas inconsistentes ou incoerentes entre si; e que mostram incongruência nas respostas que compõem uma mesma dimensão. Vale ressaltar que índices de baixa consistência foram verificados em algumas dimensões na versão traduzida para o contexto brasileiro, e na versão adaptada e validada em versão brasileira eletrônica (REIS; LAGUARDIA; VASCONCELOS et al., 2016; ANDRADE; MELO; SILVA et al., 2017). Demais estudos também apresentaram baixos valores de Alfa de *Cronbach* em algumas dimensões do HSOPSC. (VERBEEK-VAN NOORD; WAGNER; VAN DYCL et al., 2014; SILVA-BATALHA; MELLEIRO, 2015; MEKONNEN; MCLACHLAN; BRIEN et al., 2017). Tais resultados sugerem estudos para melhor elucidação deste aspecto em especial.

Enfim, em virtude do aumento da capacidade cirúrgica global (SHRIME; BICKLER; ALKIRE et al., 2015), a segurança dos processos que abrangem a assistência perioperatória e a redução de erros cirúrgicos depende do trabalho em equipe, comunicação assertiva, hierarquias com modelo de gestão participativo e com estruturas horizontais para que ocorra o fortalecimento da cultura de segurança e das práticas e processos perioperatórios (COSTA; RAMOS; GABRIEL et al., 2018; SMILEY; OFORI; SPANGLER et al., 2019).

Esta etapa da pesquisa teve como objetivo a avaliação da cultura de segurança e, neste contexto, a cultura institucional se reflete nos processos

perioperatórios e precisa ser considerada como um dos métodos de avaliação que pode ser utilizado para a redução de riscos e promoção da qualidade da assistência cirúrgica (SMILEY; OFORI; SPANGLER et al., 2019).

As fragilidades identificadas nos hospitais apontam a necessidade de ações promotoras da cultura de segurança, a partir da análise das lacunas identificadas. Deste modo, a partir do reconhecimento do importante papel, missão e valores das instituições de saúde, líderes da hierarquia hospitalar, supervisores/chefias departamentais e profissionais que tem contato direto com o paciente tem a oportunidade de propor mudanças positivas para promoção de processos e sistemas em prol da SP.

Destaca-se que a cultura justa e não punitiva frente aos erros é fundamental para o fortalecimento da cultura de segurança, assim, os erros devem ser vistos como oportunidade de aprendizagem e melhoria dos processos. Além disso, os recursos e estrutura para garantir a segurança devem ser suficientes e adequados, a aceitação da responsabilidade deve ser compreendida por todos os profissionais, independente do nível hierárquico, em relação à segurança individual e da equipe, pacientes e visitantes. A segurança perioperatória deve tornar-se prioridade entre as metas estratégicas dos hospitais, frente aos riscos apontados na literatura e reiterados nos resultados apresentados.

Considerando esta pesquisa, recomenda-se que as dimensões fragilizadas da cultura de segurança sejam alvo de urgente intervenções e ações de melhorias. Quanto às dimensões fortalecidas ou adequadas, infere-se que são indicadores que podem influenciar, positivamente, na redução da ocorrência dos EA cirúrgicos, pois permitem a criação de barreiras para que estes não ocorram.

6.2 EVENTOS ADVERSOS CIRÚRGICOS

A alta incidência de EA cirúrgicos evitáveis nos hospitais participantes desta pesquisa, identificados por intermédio da técnica de revisão retrospectiva de prontuários, demonstra a importância de se compreender as características e as causas associadas à sua ocorrência; e representam sinais de alerta sobre a possível magnitude do problema em hospitais brasileiros. Esta pesquisa apresenta o panorama dos EA cirúrgicos de três hospitais de ensino da região sul do Brasil.

Os EA cirúrgicos são relevantes devido ao impacto causado sobre a saúde dos pacientes e por serem, potencialmente, preveníveis (COSTA; MOREIRA; GUSMÃO, 2017). Tais desfechos indesejáveis devem ser combatidos, pois podem até mesmo levar ao óbito, conforme verificado em estudos pioneiros realizados no Brasil;

os quais identificaram como mais prevalentes os EA cirúrgicos (MENDES; PAVÃO; MARTINS et al., 2005; MENDES; MARTINS; ROSENFELD et al., 2009; MENDES; PAVÃO; MARTINS et al., 2013).

Sua ocorrência e evitabilidade estão associadas à falha ativa ou à condição latente, e até mesmo violação de normas e padrões (REASON, 2000); correlacionados a recursos materiais, tecnológicos e humanos (HENRIQUES; COSTA; LACERDA, 2016). Neste contexto, a prevalência de EA cirúrgicos remete aos problemas na qualidade do cuidado prestado em todo o mundo (WHO, 2008b; OMS, 2009; MENDES; PAVÃO; MARTINS et al., 2013; HENRIQUES; COSTA; LACERDA, 2016).

Assume-se, nesta pesquisa, que a causa dos EA cirúrgicos é multifatorial e está atribuída à complexidade dos procedimentos, à interação entre os integrantes da equipe multiprofissional, e ao trabalho realizado sob pressão dentro do Centro Cirúrgico. Por sua vez, os procedimentos cirúrgicos são fundamentais para o tratamento de patologias agudas e crônicas e para a prevenção de agravos a integridades física, contudo, esses estão associados a riscos de complicações e morte (SECANELL; ORREGO; VILA et al., 2014; ZAPATA; SAMANIEGO; CUÉLLAR et al., 2015).

A literatura aponta limitações na técnica de revisão retrospectiva de prontuários, como a dificuldade no uso rotineiro em serviços de saúde e problemas relacionados à subestimação de taxas de eventos, devido ao desempenho dos revisores que atuam nas fases de rastreamento de pEA (WILSON; MICHEL; OLSEN et al., 2012). Contudo, essa metodologia foi utilizada nos principais estudos sobre a ocorrência de EA e permanece como a mais utilizada na fase diagnóstica, ou de mensuração de frequência, e classificação de tipos de EA (CORRÊA; MENDES, 2017).

Historicamente a notificação de IRAS, anteriormente nominadas infecções hospitralares, se iniciou com a notificação passiva, na qual os profissionais notificavam espontaneamente as ocorrências, por eles consideradas como infecção

decorrente da assistência. Progressivamente, os profissionais do Serviço de Controle de Infecção Hospitalar passaram a buscar os casos, ativamente, por meio da revisão prospectiva de prontuários, prática que perdura e é indicada pelos órgãos governamentais. Eventualmente, na área da SP é possível que à prática da notificação de EA pela equi

pe seja atrelada a busca ativa de casos, o que contribuiria para o aprimoramento do diagnóstico situacional. Contudo, destaca-se a importância educativa da notificação do evento pelo profissional, como elemento de reconhecimento e comprometimento com a SP e ações corretivas.

Os prontuários, apesar de apresentarem subnotificações na análise do registro do evento, são considerados como método padrão ouro neste tipo de investigações sobre a qualidade da assistência (PARANAGUÁ; BEZERRA; SANTOS et al., 2014; NOVARETTI; SANTOS; QUITÉRIO et al., 2014) e promovem o desenvolvimento de estratégias de gestão da segurança em diversos países. (MENDES; TRAVASSOS; MARTINS et al., 2005; MENDES; TRAVASSOS; MARTINS et al., 2008).

Na primeira fase desta investigação, para a identificação de pEA, utilizou-se metodologias consagradas e ferramentas validadas, como o formulário de rastreamento preconizado pelo protocolo *Canadian Adverse Events Study* – CAES (BAKER; NORTON; FLINTOFT, et al., 2004), traduzido e adaptado transculturalmente para o contexto brasileiro (MENDES; TRAVASSOS; MARTINS, et al., 2008) e o módulo cirúrgico do *Global Trigger Tool* desenvolvido pelo *Institute of Healthcare Improvement* (IHI) dos Estados Unidos (GRIFFIN; RESAR, 2009). Os resultados mostram que tais ferramentas foram complementares entre si e apresentaram pistas aos investigadores, ao sinalizarem rastreadores positivos durante a busca de casos.

Importante destacar que no Hospital A as revisões foram realizadas em prontuários físicos, no Hospital B em prontuários eletrônicos durante o período de internação dos pacientes, bem como, em registros escritos dos retornos ambulatoriais no pós-operatório. E no Hospital C, as análises retrospectivas ocorreram, na totalidade, em prontuários eletrônicos. Os prontuários representam a forma de comunicação escrita entre a equipe multiprofissional e contempla o resultado dos atendimentos prestados aos pacientes durante o período de internação hospitalar (PARANAGUÁ; BEZERRA; SANTOS et al., 2014;

NOVARETTI; SANTOS; QUITÉRIO et al., 2014); bem como do atendimento ambulatorial. O prontuário eletrônico consiste em documento legal que contêm dados sobre informações pessoais e história pregressa de saúde e doença. Tais informações podem subsidiar a tomada de decisão pelo profissional da equipe multidisciplinar de saúde durante os cuidados prestados (MARTINS; LIMA, 2014).

Durante as análises retrospectivas de prontuários, conforme protocolo sugerido pelo CAES, constatou-se 53 critérios de rastreamento no Hospital A, 128 no Hospital B e 126 no Hospital C, durante a primeira fase investigativa de busca por pEA cirúrgicos. Todos os rastreadores apresentaram positividade em algum momento das avaliações, porém, os que mais se destacaram foram: 7 - Outras complicações inesperadas ao tratamento; 5 - Retorno não planejado à sala de cirurgia (apenas nos hospitais B e C) e 12 - Infecção/ Septicemia. Verifica-se que muitos dos rastreadores positivos encontrados nesta pesquisa também foram identificados em outros estudos (LETAIEF; MHAMDI; EL-ASADY et al., 2010; MOURA; MENDES, 2012; RAFTER; HICKEY; CONROY et al., 2017; PIERDEVARA; VENTURA; EIRAS, 2016).

Em relação aos critérios propostos no módulo cirúrgico *Global Trigger Tool*, identificou-se 48 rastreadores no Hospital A, 102 no Hospital B e 90 no Hospital C. Tais sinalizações indicaram a ocorrência de pEA cirúrgicos e os 11 *triggers* se monstraram positivos em algum momento das avaliações retrospectivas dos prontuários. Contudo, prevaleceram os seguintes gatilhos rastreadores: S11 - Ocorrência de qualquer complicação cirúrgica, S1 - Retorno à sala cirúrgica (apenas nos Hospitais B e C) e S10 - Lesão, reparo ou remoção de órgão durante o procedimento cirúrgico.

Na área da SP os rastreadores que recebem denominação em inglês de *trigger tools* têm sido, amplamente, utilizados com a finalidade de facilitar a detecção de EA nos registros de saúde. A partir dessa sinalização, recomenda-se analisar o caso para confirmação, ou não, da ocorrência do dano ao paciente, sua gravidade e fatores contribuintes (CORRÊA; MENDES, 2017). A metodologia *Global Trigger Tool* é ferramenta viável, útil para detectar as características dos EA e indicada para promover a segurança dos pacientes em estratégias de prevenção, no contexto hospitalar. Além disso é ferramenta que fornece informações importantes aos profissionais que trabalham na gestão da qualidade e implementação de medidas para melhoria contínua (PIERDEVARA; VENTURA;

EIRAS et al., 2016; PIERDEVARA; VENTURA; EIRAS et al., 2017; HU; WU; ZHAN et al., 2019).

Neste contexto, estudo realizado nos Estados Unidos, em oito hospitais de cuidados agudos e durante período de cinco anos, demonstrou que a metodologia *Global Trigger Tool*, que foi aprimorada para uso nos respectivos contextos, permitiu a caracterização significativa de vários tipos de EA e as áreas de atendimento de sua ocorrência. Contudo, os pesquisadores revelaram que a descoberta mais importante foi de que a categoria denominada "cirúrgico/processual" correspondeu aos EA prevalentes (KENNERLY; KUDYAKOV; GRACA et al., 2014).

Foram observados gatilhos, ou pistas semelhantes, ao desta pesquisa em estudo retrospectivo de prontuários de pacientes admitidos em departamento de cirurgia geral na Espanha, que identificou 340 gatilhos em 125 pacientes. Os autores concluíram que a ferramenta *Global Trigger Tool* demonstrou alta sensibilidade e especificidade, o que indica sua capacidade de mostrar a realidade do EA em pacientes cirúrgicos (ZAPATA; SAMANIEGO; CUÉLLAR et al., 2015).

A frequência de pEA cirúrgicos detectada na primeira fase da pesquisa foi de 24,1% no Hospital A, 18% no Hospital B e 16,4% no Hospital C. Os dados se mostram inferiores aos verificados por revisores da Suécia, no qual 34,3% dos prontuários apresentaram pEA (HALFON; STAINES; BURNAND, 2017) e, em estudo realizado em hospitais irlandeses, com 45% de pEA (RAFTER; HICKEY; CONROY et al., 2017). No entanto, dado semelhante ao verificado nesta pesquisa foi demonstrado em estudo desenvolvido em oito países do Oriente Médio e África do Sul, que resultou em taxa média geral de 21,6% prontuários com critérios de rastreamento positivo, indicando pEA, na revisão primária (WILSON; MICHEL; OLSEN et al., 2012).

A segunda fase desta investigação permitiu a confirmação diagnóstica da ocorrência da maioria dos pEA, indicando a prevalência de 21% no Hospital A, 14,2% no Hospital B e 12,7% no Hospital C. Valores superiores foram registrados em estudo espanhol com prevalência 36,8% (ZAPATA; SAMANIEGO; CUÉLLAR et al., 2015), em estudo americano que apresentou taxa de complicações cirúrgicas de 39,7% (MEHTA; DIMOU; ADHIKARI et al., 2016), e em estudo brasileiro, realizado em três hospitais da Região Sudeste, com 66,7% de EA evitáveis, sendo que 32,3% destes foram relacionados à cirurgia (MENDES;

PAVÃO; MARTINS, 2013).

Demais estudos realizados nesta temática mostram resultados semelhantes aos verificados no Hospital B e Hospital C, porém inferiores ao detectado no Hospital A. São esses: prevalência de 13,1% em estudo realizado em 30 hospitais públicos de cuidados agudos na Europa (RAFTER; HICKEY; CONROY et al., 2017); 15,1% em hospital universitário do Japão (SHIOZAKI; MORIMATSU; MATSUSAKI et al., 2016) e de 15,4% em hospitais da Suécia (NILSSON; RISBERG; MONTGOMERY et al., 2016).

Outras pesquisas que avaliaram a ocorrência de EA foram: pesquisa no Texas - Estados Unidos, realizado em oito hospitais agudos durante período de cinco anos, que verificou 40,5% (n=676) de eventos relacionados especificamente à categoria cirúrgica/processo (KENNERLY; KUDYAKOV; GRACA et al., 2014) e estudo europeu, realizado em 30 hospitais públicos de cuidados agudos da Irlanda que detectou taxas de EA cirúrgicos de 13,1% (RAFTER; HICKEY; CONROY et al., 2017).

Os dados desta pesquisa mostram que os EA foram detectados, prevalentemente, durante o período de internação no Hospital B e Hospital C; ao contrário do observado no Hospital A, cujos EA foram detectados, em sua maioria, no retorno ambulatorial dos pacientes.

Em investigação realizada no mesmo Hospital A, porém, em período e modelo de gestão diferentes, entre junho de 2014 a maio de 2015, identificou-se a prevalência de 21,8% de EA cirúrgicos; em 52,4% dos casos a detecção ocorreu no retorno ambulatorial (BATISTA; CRUZ; ALPENDRE et al., 2019). Esse fato demonstra característica específica deste hospital, com retorno ambulatorial e registro das complicações em prontuário; e reitera a importância da investigação e vigilância pós alta para real conhecimento da prevalência, e consequentes ações preventivas no contexto da assistência cirúrgica.

Outro estudo que utilizou 10 indicadores de segurança do paciente para mensurar EA evitáveis cirúrgicos, com utilização de dados administrativos e ambulatoriais atribuíveis aos cuidados hospitalares, identificou 11.141 eventos que ocorreram dentro de 30 dias após a alta. Os mais frequentemente identificados foram lesão por pressão e embolia pulmonar ou trombose venosa profunda; mais de 60% destes foram detectados dentro de 14 dias após a alta, na primeira consulta de

retorno ambulatorial (MULL; BORZECKI; CHEN et al., 2014), assim como ocorreu no Hospital A desta pesquisa.

Em relação aos resultados do Hospital B e do Hospital C, dados semelhantes foram observados em hospitais irlandeses, pois a maioria dos EA foram detectados e registrados durante o internamento (72,4%) em comparação aos detectados após a
alta (23,5%) (RAFTER; HICKEY; CONROY et al., 2017).

Ao analisar o número de EA por paciente, grau de dano e evitabilidade, foi possível realizar diagnóstico situacional sobre a ocorrência desses agravos. A maioria dos pacientes avaliados apresentou um EA cirúrgico: Hospital A (75,6%), Hospital B (41,2%) e Hospital C (54,8%). Entretanto, no Hospital B, destaca-se maior frequência de dois eventos (35,3%), quando comparado aos demais hospitais (19,5% no Hospital A e 19,4% no Hospital C). Nos Hospitais B e C taxas expressivas da ocorrência de três a nove eventos por paciente (23,5% e 25,8%, respectivamente) foram observadas. Deste modo, infere-se que, potencialmente, a gravidade ou condição clínica do paciente pode contribuir para a ocorrência de diversos incidentes com dano em um mesmo indivíduo.

Observa-se que na Suécia 26% dos pacientes operados apresentaram pelo menos um EA (RUTBERG; RISBERG; SJODAH et al., 2014), assim como, demais estudos registraram a evidência da vulnerabilidade a esses incidentes com dano (PARANAGUÁ; BEZERRA; SILVA et al., 2013; NILSSON; RISBERG; MONTGOMERY et al., 2016; SHIOZAKI; MORIMATSU; MATSUSAKI et al., 2016; RAFTER; HICKEY; CONROY et al., 2017).

Quanto à gravidade, os classificados como “Fortemente prevenível” ou “Potencialmente prevenível”, identificados nesta pesquisa, corresponderam a maioria (92,5% no Hospital A, 93,62% no Hospital B e 96,36% no Hospital C). Em relação ao grau de dano, prevaleceu a classificação moderados nos três hospitais (Hospital A -43,4%; Hospital B -47,44% e Hospital C 49,09%).

Assim como observado nesta pesquisa, revisão sistemática sobre estudos retrospectivos que investigaram a ocorrência de EA cirúrgicos mostrou que foram moderados ou leves (ANDERSON; DAVIS; HANNA et al., 2013). Valores semelhantes foram evidenciados também em hospitais irlandeses, onde mais de dois terços dos 70% de eventos evitáveis causaram danos do tipo leve a moderado (RAFTER; HICKEY; CONROY et al., 2017). Porcentagens diferentes foram

verificadas em hospitais suecos, com 62,5% de EA classificados como potencialmente evitáveis e mais da metade destes causaram prolongamento da internação ou readmissão. Porém, verificou-se que 4,7% destes eventos foram responsáveis por danos permanentes ou morte (NILSSON; RISBERG; MONTGOMERY et al., 2016).

Estudo realizado em 300 hospitais de nove países europeus avaliou se as diferenças na proporção de pacientes para enfermeiros e as qualificações educacionais destes enfermeiros poderiam estar associadas à variação da mortalidade hospitalar, após procedimentos cirúrgicos e verificou que o aumento da carga de trabalho de um enfermeiro por um paciente aumenta em 7% a probabilidade de um paciente internado morrer dentro de 30 dias após sua admissão. O aumento de 10% de enfermeiros que possuem graduação em nível superior está associado com a diminuição dessa probabilidade em 7%. Estas descobertas enfatizam a importância da educação de nível superior para os enfermeiros na redução de mortes hospitalares evitáveis (AIKEN; SLOANE; BRUYNEEL et al., 2014).

Neste sentido, os resultados sobre os EA cirúrgicos que resultaram em óbito apresentaram diferença com significância estatística ($p<0,001$): Hospital A (1,9%), Hospital B (11,54%) e Hospital C (9,09%). Estudo brasileiro apresentou nove pacientes com danos permanentes (21,9%), dos quais sete (17,1%) evoluíram para óbito (MOURA; MENDES, 2012). Outros estudos também revelaram taxas acerca dessa variável de desfecho em pacientes cirúrgicos: 3,21% (PARANAGUÁ; BEZERRA; SILVA et al., 2013); 3,6% (ANDERSON; DAVIS; HANNA et al., 2013), 5,8% (MEHTA; DIMOU; ADHIKARI et al., 2016) e 6,7% (RAFTER; HICKEY; CONROY et al., 2017).

Em contrapartida aos diversos resultados sobre óbitos decorrentes de EA cirúrgicos relatados acima, estudo com amostra estratificada de 400 internações cirúrgicas não identificou óbito relacionado ao EA (HALFON; STAINES; BURNAND, 2017). Deste modo, conclui-se que os Hospitais B e C apresentam dados preocupantes, com altas taxas de óbitos decorrentes de EA cirúrgicos. Tais dados podem estar relacionados à gravidade dos atendimentos realizados nestes hospitais, porém, sugere-se vigilância e investigação para compreender os motivos desta alta incidência.

Em termos gerais sobre óbitos em organizações hospitalares, investigação

conduzida no Brasil para estimativas destas ocorrências em relação ao número de internações no SUS e na saúde privada, detectou que no ano de 2015 ocorreram entre 104.187 e 434.112 possíveis óbitos associados a EA. Estas estimativas chamam a atenção sobre a necessidade de compreender o contexto no qual os pacientes estão sujeitos a danos causados pela assistência à saúde, a fim de direcionar políticas públicas e ações para melhoria da qualidade dos serviços prestados (MAIA; FREITAS; GALLO et al., 2017).

A análise das caraterísticas demográficas e clínicas dos pacientes com EA cirúrgicos demonstraram que a média de idade (em anos) foi de 49,3 (DP±15,1) no Hospital A, 47,63 (DP±18,5) no Hospital B e 48,0 no Hospital C (DP±20,1), com prevalência de pacientes do sexo feminino nos hospitais A e B. Diferentes estudos para investigação de EA cirúrgicos corroboram com os dados apresentados no Hospital A e Hospital B, com predominância do sexo feminino e média de idade entre 37 e 49 anos (BEZERRA; BEZERRA; PARANAGUÁ et al., 2015; PARANAGUÁ, BEZERRA; MOREIRA et al., 2016; ARAÚJO; CARVALHO, 2018); (AGUIAR; GOMES; SANTOS et al., 2018).

Em contrapartida, os resultados demonstrados no Hospital C, sobre a prevalência de pacientes do sexo masculino, foi semelhante a estudo realizado em 63 hospitais suíços, porém, a média de idade prevalente foi de 69 anos (NILSSON; RISBERG; MONTGOMERY et al., 2016). A prevalência do sexo masculino no Hospital C pode ter ocorrido pelo fato de ser um hospital de referência para atendimento de emergência, principalmente para pacientes politraumatizados e com problemas ortopédicos agudos.

Diversos estudos demostram que a frequência de EA aumenta com a idade, bem como, o risco pode ser maior entre pacientes cirúrgicos, com ocorrência de complicações, tais como infecção, insuficiência renal e trombose venosa profunda (ADAM; RITZ; KATHER et al., 2014; NILSSON; RISBERG; MONTGOMERY et al., 2016). O progresso das técnicas cirúrgicas ampliou as indicações e intervenções em pacientes mais velhos e frágeis (NILSSON; RISBERG; MONTGOMERY et al., 2016), corroborando com maior frequência na condição cirúrgica. Porém, tais evidências não representam os resultados desta pesquisa, pois evidenciou-se menor incidência de complicações cirúrgicas para os pacientes com idade ≥ 60 anos. Tal fato pode ser explicado pelo fato do Hospital B e Hospital C serem especializados em

atendimentos de urgência e emergência a pacientes que sofreram acidentes com politraumatismos, os quais são mais prevalentes entre indivíduos adultos jovens.

Nos três hospitais investigados a análise das comorbidades/fatores de risco destaca a Hipertensão arterial e a variável “Outros” (Cardiopatia, Artrite, Osteoporose, Sarcoma, Metástase, Hepatite, Problema Renal, Drogadicção e Etilismo). Porém, o Tabagismo foi o fator de risco mais prevalente no Hospital A. Estudo brasileiro realizado em centro cirúrgico de hospital de ensino corrobora com dados aqui apresentados, pois a hipertensão arterial foi reportada como de maior frequência. Esta, e outras comorbidades, foram identificadas em 23,7% dos pacientes que sofreram algum tipo de incidente cirúrgico (BEZERRA; BEZERRA; PARANAGUÁ et al., 2015).

Outro estudo que acompanhou amostra de 69 pacientes cirúrgicos, do primeiro dia de pré-operatório até a alta, em hospital universitário público de grande porte do Estado do Rio de Janeiro, constatou que entre idosos que apresentaram prolongamento dos dias de internamento, houve associação com doenças crônicas, como hipertensão arterial sistêmica, diabetes *mellitus* e neoplasias. Já nos adultos jovens, destacaram-se as comorbidades anemia e hipertensão arterial sistêmica (PEREIRA; SANTANA; SANTOS et al., 2014). Investigação sobre ocorrência de complicações cirúrgicas no Estado de Pernambuco também descreveu a hipertensão arterial como fator de risco prevalente, com 59,9% (n=118) dos casos investigados (AGUIAR; GOMES; SANTOS et al., 2018).

Quanto ao fator de comorbidade Tabagismo, prevalente no Hospital A, são comprovados os benefícios aos pacientes cirúrgicos quando param de fumar, conforme revela estudo que avaliou a relação entre a duração do uso de tabaco e os problemas respiratórios em adultos nos Estados Unidos. Os resultados foram consistentes com estratégias de prevenção pois, minimizam os sintomas respiratórios, a hiper-responsividade brônquica e previnem o declínio excessivo da função pulmonar entre fumantes com DPOC e entre os sem sintomas crônicos. As recomendações dos serviços que atuam como força tarefa na prevenção do uso do cigarro nos Estados Unidos sugerem que os fumantes recebam aconselhamento e terapias farmacológicas, afinal, estas medidas aumentam as taxas de cessação do tabagismo (LIU; PLEASANTS; CROFT et al., 2015).

Para reduzir os possíveis riscos inerentes à cirurgia, a assistência deve ser planejada de acordo com a condição clínica do paciente, e de forma individualizada

(VITURI; ÉVORA, 2014). Neste contexto, a realização de cirurgias eletivas minimiza os riscos, pois permite o planejamento de assistência conforme as respectivas particularidades, estado de saúde e características da cirurgia (SENA; NASCIMENTO; MAIA, 2013).

Deste modo, as diferentes comorbidades e fatores de risco devem ser avaliados e, nesta pesquisa, todos os pacientes submetidos à cirurgia eletiva passaram por avaliação anestésica pré-operatória e receberam assistência de enfermagem específica para este momento. Porém, muitos dos EA cirúrgicos identificados ocorreram em pacientes submetidos à cirurgia de urgência e emergência, os quais não puderam passar por esta avaliação, principalmente nos Hospitais B e C, em virtude das características e gravidade do atendimento agudo.

Neste contexto, merece destaque iniciativas, como a denominada *Safe abdominal surgery* (Cirurgia abdominal segura), adotada por mais de 40 departamentos cirúrgicos suecos desde 2011, que tem por objetivo reduzir as complicações gerais, com avaliação do paciente em cada etapa do processo cirúrgico,

desde a decisão cirúrgica até o seguimento após a alta hospitalar, durante três meses (SPANJERSBERG; REURINGS; KEUS et al., 2011). Além disso, ações para que o paciente pare de fumar e ingerir bebidas alcoólicas antes da cirurgia são incentivadas (GRONKJAER; ELIASEN; SKRUBBELTRANG et al., 2014; THOMSEN; VILLEBRO; MOLLER, 2014).

Características relativas à internação foram analisadas, e a média de internamento em dias dos pacientes com EA cirúrgico foi de quatro dias no Hospital A (1 – 98 dias), 12 dias no Hospital B (1-72 dias) e 17 dias no Hospital C (1-117 dias). A média de internamento foi maior no Hospital B e Hospital C, possivelmente tais resultados ocorreram em virtude da gravidade dos casos agudos atendidos, e decorrentes de situações de emergência, os quais necessitaram de tratamento cirúrgico e tiveram seu período de internação prolongado.

Na assistência cirúrgica, o estudo do perfil das internações é relevante em virtude das diversas especialidades de tratamento, complexidade do cuidado prestado, arsenal tecnológico, rotatividade das internações e exigência de conhecimento especializado fundamental para assistência segura e de qualidade (PARANAGUÁ; BEZERRA; SANTOS et al., 2014). Segundo a Agência Nacional de Saúde Suplementar, o tempo de internação ideal, para hospitais de grande porte,

oscila entre quatro e cinco dias. Sabe-se que a média de permanência, em hospitais de cuidados agudos, acima de sete dias, está relacionada a vários riscos como, por exemplo, de infecção hospitalar. Este indicador de desempenho permite a implementação de boas práticas clínicas e a gestão eficiente do leito hospitalar (BRASIL, 2013d). Tanto os erros como os EA podem implicar em aumento do tempo de internação e custos assistenciais, bem como, encargos jurídicos. (RUTBERG; RISBERG; SJÖDAHL et al., 2014).

A permanência do paciente cirúrgico por tempo maior do que o esperado pode indicar o diagnóstico de enfermagem denominado “recuperação cirúrgica retardada” (SANTANA; AMARAL; PEREIRA et al., 2014), definido como extensão do número de dias de pós-operatório necessários para iniciar e desempenhar atividades que mantêm a vida, a saúde e o bem-estar (HERDMAN, 2012). A presença deste diagnóstico de enfermagem indica limitações nos pacientes e influencia os padrões de qualidade da assistência (PEREIRA; SANTANA; SANTOS et al., 2014).

O tempo de internação pré-operatório dos pacientes ≥24 horas apresentou significância estatística entre os hospitais (p=0,006), pois a porcentagem resultante no Hospital B (44,1%) foi superior à verificada no Hospital A (14,6%) e Hospital C (16,1%). Por esta razão, é fundamental o preparo correto do paciente no período pré-operatório para minimizar, ou evitar, a ocorrência de algum incidente durante a cirurgia, ou no período pós-operatório. (WHO 2008b; WHO, 2009a; OMS, 2009). Estudo brasileiro sobre pacientes submetidos às cirurgias limpas, para correção de fraturas de fêmur, demonstrou que o tempo de internação pré-operatório foi considerado fator de risco para complicações, entre elas, a infecção cirúrgica (PEREIRA; REZENDE; COUTO et al., 2015).

Os dados sobre episódios de readmissão mostraram significância estatística entre os hospitais (p=0,003): Hospital A (9,8%), Hospital B (29,4%) e Hospital C (45,2%). Deste modo, o Hospital C apresentou maior frequência de readmissões em decorrência de EA cirúrgicos, seguido do Hospital B. Neste contexto, estudo americano com 39.616 pacientes submetidos a procedimento cirúrgico primário, entre 2007 a 2010, comparou diferentes escores de comorbidade para previsão de resultados cirúrgicos e detectou taxa de readmissão de 14,1% em 30 dias (MEHTA; DIMOU; ADHIKARI et al., 2016).

Dados semelhantes ao Hospital A, porém com valores bem inferiores ao apresentado nos Hospitais B e C foram encontrados em estudo que analisou 9.274 procedimentos cirúrgicos com taxa de readmissão de 9,9%. Os investigadores concluíram que os EA cirúrgicos estão associados ao aumento no número de readmissões; e referem que a prevenção, ou a mitigação de seus efeitos, pode contribuir com a melhoria da qualidade e reduzir readmissões (NANDAN; BOHNEN; CHANG et al., 2017).

No entanto, outro fator que se observa na literatura está relacionado às causas das readmissões cirúrgicas, pois, embora sua redução seja consistente com a melhoria da qualidade, a validade do uso de métrica geral para todas as readmissões de uma instituição hospitalar pressupõe que todos os eventos sejam evitáveis (MCINTYRE; ARBABI; ROBINSON et al., 2016; GRAHAM; MULL; WAGNER et al., 2019).

Neste contexto, revisão retrospectiva de prontuários de pacientes readmitidos dentro de 30 dias após a alta do serviço de cirurgia geral em Washington nos Estados Unidos, entre 1º de julho de 2014 e 30 de junho de 2015,

em um centro de trauma nível I e hospital pertencente à rede de segurança revelou que das 2.100 altas a taxa geral de readmissão foi de 8,2% (n=173). Provável complicação cirúrgica evitável foi verificada em 9,2% (n=16) dos casos. Detectou-se que a maioria dos pacientes apresentaram outros motivos não relacionados à cirurgia 35,8% (n=62) para readmissão, tais como: uso de drogas injetáveis, falta de moradia ou dificuldade de chegar às consultas de atendimento, infecções não detectáveis durante a admissão índice e sequelas próprias de sua lesão ou condição (MCINTYRE; ARBABI; ROBINSON et al., 2016).

Entretanto, estudo recente sobre as readmissões cirúrgicas no âmbito nacional dos EUA, para comparar nova métrica de qualidade para classificação de readmissões, especificamente associadas à cirurgia, com a métrica geral existente (GRAHAM; MULL; WAGNER et al., 2019) usou definição validada pelo consenso de um painel de especialistas (COPELAND; GRAHAM; RICHMAN et al., 2017; MULL; GRAHAM; MORRIS et al., 2018). Os participantes foram questionados da seguinte maneira: “O motivo da readmissão reflete possíveis problemas cirúrgicos na qualidade dos cuidados na admissão índice?”. As respostas foram consideradas readmissões cirúrgicas se o painel concordasse que elas estavam “provavelmente

relacionadas” ou “diretamente relacionadas. Após três rodadas, o painel de especialistas identificou 16 razões para a readmissão associada à qualidade do procedimento cirúrgico. Os resultados demostraram que as correlações entre as complicações pós-alta hospitalar e a nova métrica específica para EA cirúrgicos foram superiores à métrica geral existente, ou seja, as complicações pós alta hospitalar podem ser utilizadas como indicador de qualidade cirúrgica. (GRAHAM; MULL; WAGNER et al., 2019). Importante salientar que os investigadores coletaram dados sobre as comorbidades dos pacientes e características da cirurgia, assim como realizado na presente pesquisa sobre a ocorrência de EA cirúrgicos.

Os dados de readmissão em diferentes estudos demonstram a necessidade de ações de vigilância pós-alta hospitalar, a fim de acompanhar a evolução do paciente, identificar eventos resultantes da assistência e elaboração de estratégias e medidas preventivas em prol da SP. Destaca-se o desenvolvimento do Programa Gestão de Altas, a partir de 2018, entre o Hospital
e a Prefeitura Municipal de Curitiba, com vistas a estreitar a comunicação entre o hospital e as unidades de saúde (EMPRESA BRASILEIRA DE SERVIÇOS HOSPITALARES (EBSERH), 2018).

A avaliação de pacientes operados em hospital dia na cidade brasileira de Salvador-Bahia, entre os anos de 2010 e 2014 para estimar a incidência de EA cirúrgicos, demonstrou que cirurgias realizadas em regime ambulatorial estão relacionadas a menores taxas de EA cirúrgicos. Contudo, os autores destacam ser indispensável a criação de sistema de seguimento dos pacientes após alta, no sentido de evitar a subnotificação e subregistros dos dados, o que pode ocultar informações e identificar taxas irreais (COSTA; MOREIRA; GUSMÃO, 2017).

As cirurgias eletivas, com EA, foram prevalentes no Hospital A (80,5%;n=33) e no Hospital B (58,8%;n=20), porém, no Hospital C, prevaleceram as cirurgias de emergência (48,4%;n=15). Importante ressaltar que as cirurgias de emergência não foram prevalentes no Hospital B, conforme o esperado, apesar deste hospital possuir as mesmas características de atendimento para casos graves e agudos do Hospital C.

Nos três hospitais investigados predominaram EA em cirurgias classificadas como limpas (Hospital A=53,7%; Hospital B=64,7% e Hospital

C=58,1%) e os procedimentos cirúrgicos realizados sob anestesia geral (Hospital A= 39%; Hospital B=79,4% e Hospital C=48,4%).

Análise retrospectiva de prontuários realizada em hospital privado na cidade de São Paulo, para identificar a ocorrência de EA graves em pacientes cirúrgicos (n=19), e seus possíveis desfechos, mostrou prevalência de cirurgias de urgência (52,53%; n=10), classificadas segundo o potencial de contaminação como limpas (57,89%;n=11) e anestesia geral (84,21%;n=16) (ARAÚJO; CARVALHO, 2018). Os dados sobre as variáveis relativas ao procedimento cirúrgico e anestésico equivalem aos evidenciados nesta pesquisa, apenas diferem quanto à urgência dos procedimentos realizados.

Os resultados em relação ao risco cirúrgico, conforme o escore da *American Society of Anesthesiology* (ASA), foram diferentes nos três hospitais analisados: no Hospital A prevaleceu ASA II (53,7%), Hospital B foi ASA III (38,2%) e no Hospital C foi ASA I (54,8%). Houve significância estatística nos pacientes com risco cirúrgico ASA IV ($p<0,001$) pois, foram identificados somente no Hospital B (17,7%). O escore ASA deve ser avaliado no pré-operatório, em conjunto com outros fatores de risco (idade, histórico de doença pulmonar obstrutiva crônica, tipo de cirurgia e status funcional, entre outros) pois, estão associados à insuficiência respiratória pós-operatória (BRUECKMANN; VILLA-URIBE; BATEMAN et al., 2013). Diversos autores relataram que o escore ASA II ou superior é um dos fatores de risco independentes para complicações pulmonares no período perioperatório (BRUECKMANN; VILLA-URIBE; BATEMAN et al., 2013; LI; YANG; ZHOU et al., 2013; CANET; SABATÉ; MAZO et al., 2015). Para os pacientes considerados de alto risco, o tempo cirúrgico deve ser minimizado (MISKOVIC; LUMB, 2017).

Estudo português demonstrou evidências da correlação do risco cirúrgico ASA e a ocorrência de Trombose Venosa Profunda no pós-operatório, em 55,1% dos pacientes classificados como ASA III, em 21,4% como ASA II e em 13,5% como ASA IV (AMARAL; PEREIRA; MORETO et al., 2017). Pesquisadores referem que pacientes classificados como ASA II, com histórico de Hipertensão Arterial Sistêmica sem tratamento, apresentam risco elevado de evento cardiocerebral quando submetidos à cirurgia (TRUJILLO; AVELAR, 2019). Nestas circunstâncias, pacientes hipertensos com ASA II e que precisam ser submetidos a cirurgias não eletivas, com evidência de lesão de órgãos, devem ser manejados com cuidado, além de se considerar monitoramento invasivo para controle da pressão arterial e venosa

central, a fim de assegurar o adequado manejo das variações da pressão arterial (JAMES; OPARIL; CARTER et al., 2014). Tal fato foi observado em diversos pacientes que apresentaram EA cirúrgicos e foram operados nos Hospitais B e C.

Quanto ao tempo de duração cirúrgica, no Hospital A prevaleceram as cirurgias acima de 120 minutos, no Hospital B as cirurgias abaixo de 120 minutos e, no Hospital C os resultados foram equivalentes. Os procedimentos acima de 120 minutos ocorreram, em sua maioria, nos pacientes jovens e adultos (abaixo de 60 anos). Estes dados corroboram com estudo brasileiro que analisou o diagnóstico de enfermagem denominado “recuperação cirúrgica retardada” em adultos e idosos hospitalizados em hospital universitário público, onde o procedimento prolongado na população adulta foi superior em 50% quando comparado aos pacientes idosos (PEREIRA; SANTANA; SANTOS et al., 2014).

Todos os pacientes cirúrgicos encontram-se em risco durante os procedimentos, porém, o fator tempo prolongado aumenta este risco em relação à ocorrência de pEA relacionados à cirurgia, anestesia, e até mesmo para a ocorrência de infecção, o que pode retardar o tempo de recuperação pós-operatória. Além do mais, o tempo prolongado está diretamente associado às cirurgias de grande porte que envolvem grandes ressecções e sangramentos, além de exposição à temperatura ambiente de cavidades corporais ou grandes vasos que, por sua vez, causam o retardo da recuperação cirúrgica (PONTES; SALAZAR; TORRES, 2013).

Na análise das especialidades cirúrgicas que resultaram em EA observa-se que no Hospital A e no Hospital B houve prevalência de EA cirúrgicos nos pacientes operados pela Cirurgia Geral (73,2% e 64,7% respectivamente) e, no Hospital C prevaleceram eventos nos pacientes operados pela especialidade de Ortopedia (51,6%). Contudo, a análise da prevalência geral de cirurgias realizadas, por especialidade, não foi realizada por não ser objeto de estudo desta pesquisa.

Em relação ao tipo de cirurgias com ocorrência de EA, no Hospital A prevaleceram as cirurgias de Hernioplastias Incisionais e Herniorrafias (26,84%), seguidas das Artroplastias Totais de Quadril/Joelho (9,76%) e Videocolecistectomias (9,76%), que resultaram em 46,36% (n=19) e foram, em sua grande maioria realizadas de forma eletiva.

No Hospital B, dentre as cirurgias com ocorrência de EA, prevaleceram as seguintes: Colecistectomia (12,1%), Laparotomias Exploradoras (9,1%),

Enterectomia/Enteroanastomose/ Enterorrafia e Ligadura de Segmento Entérico (9,0%), em conjunto com as cirurgias neurológicas de Craniectomia Descompressiva (1,5%), Cranioplastia (1,5%), Craniotomia (6,1%), Derivação Ventricular Externa (3,0%) e Microdissecção de Tumor Cerebral (1,5%), que representaram o total de 43,8% (n=29) dos procedimentos realizados. Constatou-se que neste hospital (B) os pacientes, durante a mesma abordagem cirúrgica, foram submetidos a múltiplos procedimentos cirúrgicos, o que explica a diversidade e quantidade de procedimentos. Destacaram-se as cirurgias eletivas e de urgência.

No Hospital C, dentre as cirurgias com ocorrência de EA, prevaleceram as cirurgias ortopédicas, que representaram 42,4% (n= 24). Dentre estas, merecem destaque a Redução Aberta e Fixação Interna de Fratura (8,8%), Instalação de Fixador Externo para Estabilização de Fratura Óssea (7%), Osteossíntese para Fixação de Fratura (5,3%) e Desbridamento de Tecidos Desvitalizados pós trauma e Curativo Cirúrgico (5,3%). A maioria dos pacientes foram submetidos a cirurgias de emergência.

Pesquisa aponta que o risco da ocorrência de EA é maior em pacientes traumatizados do que para pacientes eletivos, com taxa de complicações de 11,4% *versus* 4,1% respectivamente (SATHIYAKUMAR; THAKORE; GREENBERG et al., 2015). Tal evidência corrobora com os dados desta pesquisa, principalmente nos resultados evidenciados nos Hospitais B e C, que realizam elevado número de atendimento em pacientes politraumatizados. O Hospital A não realiza atendimento em pacientes vítimas de politraumatismo.

Os resultados sobre a prevalência e evitabilidade de EA cirúrgicos, bem como, a apresentação sobre as diversas variáveis demográficas e clínicas dos pacientes, internamento, procedimento cirúrgico e anestésico, aqui apresentados, apontam para a importância de estratégias promotoras da segurança cirúrgica com vistas à redução dos danos aos pacientes. Neste sentido, revisão sistemática demonstrou que o emprego das listas de verificação de segurança faz parte destas estratégias (HOWELL; PANESAR; BURNS et al., 2014). As orientações para sua utilização no ambiente cirúrgico, bem como, nos períodos pré e pós-operatórios, constam no Programa Cirurgias Seguras Salvam Vidas (WHO, 2008b; OMS, 2009).

A assistência ao paciente cirúrgico, em qualquer etapa operatória, implica em ações contínuas em prol da segurança. Desta forma, o cuidado no pré, trans e pós-operatório tem associação com a qualidade da assistência prestada e com a recuperação do paciente (HENRIQUES; COSTA; LACERDA, 2016). Nos três hospitais participantes deste estudo observou-se o uso do checklist cirúrgico da OMS nos respectivos centros cirúrgicos, o qual aborda três momentos importantes de checagem: na entrada do paciente (antes da indução anestésica), *Time out* ou pausa (antes da incisão cirúrgica) e saída (antes do paciente deixar a sala cirúrgica). O diferencial foi observado no Hospital A, que padronizou, complementarmente, outra lista de verificação para segurança cirúrgica para períodos pré e pós-operatórios, para uso nas unidades de internação (ALPENDRE; CRUZ; DYNIEWICZ et al., 2017).

No Hospital A as respectivas listas de verificação cirúrgica estão em processo de melhoria contínua, a fim de que a segurança dos pacientes em todo período perioperatório possa ser contemplada. Adaptar protocolos do *checklist* de cirurgia segura ao contexto local promove sua utilização eficaz. Porém, estudos demonstram que apenas exigir o uso de *checklists* cirúrgicos não é suficiente para garantir sua implantação e aceitação (GILES; MUNN; AROMATARIS et al., 2017). Por este motivo, outras iniciativas são necessárias para a implementação eficaz, taiscomo: treinamento em equipe, educação continuada e medidas de engajamento multidisciplinar com incorporação de *feedback* da equipe para modificações e adaptações necessárias, a fim de evitar redundâncias ou informações duplicadas (LEAPE, 2014; URBACH; GOVINDARAJAN; SASKIN et al., 2014; GAGLIARDI; STRAUS; SHOJANIA et al., 2014; TREADWELL; LUCAS; TSOU, 2014; GILES; MUNN; AROMATARIS et al., 2017).

Apesar das iniciativas em prol das melhorias da segurança cirúrgica, os resultados desta pesquisa demonstram a ocorrência de EA cirúrgicos por falhas técnicas operatórias e/ou causas clínicas. Quanto aos eventos relacionados à falhas técnicas, destacaram-se: Hematoma/seroma, Deiscência, Perfuração/Laceração, Necrose de parede, Eventração, Hemorragia, Fístulas, Reposicionamento de dispositivos e falhas na fixação do foco da fratura. Tais eventos representaram taxas de 49,1% no Hospital A, 34,6% no Hospital B e 25,5% no Hospital C.

Estes índices alertam para a necessidade do aprimoramento da técnica cirúrgica para a prevenção de danos, e são semelhantes a estudo chinês que constatou ocorrência de 61,6% (n=16) de EA decorrentes dessas falhas (WANG; SHIH; CHEN et al., 2016), bem como, de estudo brasileiro que demonstrou falhas técnicas em centro cirúrgico em 27% (n=7) como causas dos EA (BEZERRA; BEZERRA; PARANAGUÁ et al., 2015). Em hospitais irlandeses a frequência de EA cirúrgicos relacionados à falhas técnicas operatórias foram prevalentes. (RAFTER; HICKEY; CONROY et al., 2017). Deste modo, esses incidentes, causados por falha técnica cirúrgica, podem resultar em dano grave para o paciente e resultar até mesmo em óbito (BEZERRA; BEZERRA; PARANAGUÁ et al., 2015).

Observa-se, portanto, que a maioria dos eventos foram relacionados a causas clínicas decorrentes da cirurgia, e não especificamente à falhas técnicas operatórias, principalmente no Hospital B e Hospital C. No entanto, no Hospital A as respectivas causas foram semelhantes. Dados similares ao desta pesquisa foram observados em estudo de revisão sistemática, que revelou a prevalência de EA relacionados ao manejo do tratamento durante a internação cirúrgica em relação aos erros de técnica cirúrgica (ANDERSON; DAVIS; HANNA et al., 2013).

Nesta pesquisa, os EA cirúrgicos decorrentes do manejo clínico foram: ocorrência de Infecção Associada à Atenção de Saúde - IRAS (Pneumonia, Infecção do Trato Urinário e Infecção de Sítio Cirúrgico, Sepse/Choque Séptico), Retenção Urinária, Embolia Gasosa, Broncoaspiração, Hipóxia, Trombose Venosa Profunda, Intoxicação por Opióide, Nefropatia/ Insuficiência Renal Aguda, Infarto Agudo do Miocárdio, Parada Cardiorrespiratória, Reação à medicação, Pneumotórax, Pneumoencéfalo Hipertensivo, Insuficiência Adrenal, Queda e Lesão por Pressão.

A análise dos dados entre os três hospitais demonstrou que as maiores porcentagens de EA cirúrgicos foram classificados na categoria IRAS, com resultados semelhantes: Hospital A (54,7%), Hospital B (53,6%) e Hospital C (47,3%). Dentre essas, o EA cirúrgico que atingiu as mais altas taxas de ocorrência foi Infecção de Sítio Cirúrgico (ISC), conforme mostra os resultados: 32,1% (n=17) no Hospital A, 21,7% (n=17) no Hospital B e 16,4% (n=9) no Hospital C.

No cenário perioperatório, uma das maiores preocupações é implementar ações para evitar a ocorrência de ISC e, neste contexto, diversos fatores de risco foram descritos e divididos em três categorias principais: a) características centradas no paciente, como idade, obesidade, desnutrição, Diabetes mellitus e outras comorbidades; b) características do procedimento cirúrgico, como classificação da ferida, complexidade da cirurgia, habilidade técnica do cirurgião, uso apropriado de profilaxia antimicrobiana, manutenção da normotermia, dentre outros; e c) ambiente da sala cirúrgica, como condições de saneamento, temperatura, umidade, ventilação e padrões de tráfego (MACEDO; KANO; BRAGA et al, 2013).

As ISC são os eventos considerados mais comuns entre os pacientes cirúrgicos, e representam 16% de todas as IRAS (CANADIAN ASSOCIATION OF WOUND CARE, 2017). No Brasil, segundo a ANVISA, as IRAS ocupam a terceira posição entre todas as demais, e compreendem de 14 a 16% (BRASIL, 2017c). Estudo evidenciou que a principal associação da interrupção na cicatrização foi a ISC (PEREIRA; SANTANA; SANTOS et al., 2014), e que ocorre em até 30 dias após o procedimento cirúrgico e/ou 90 dias após inserção de implante (CDC, 2017a).

A utilização de protocolos assistenciais, *bundles*, guias e manuais sobre segurança do paciente cirúrgico são relevantes para a redução das taxas de ISC nos hospitais (BRASIL, 2017c). A literatura mostra a iniciativa de segurança importante, que é a elaboração de *checklist* para prevenção de ISC (ROSCANI; FERRAZ; OLIVEIRA FILHO et al., 2015). Outro fator que contribui para prevenção da ISC está relacionado à cultura de segurança em ambientes de centro cirúrgico e unidades de internação cirúrgica. Tal fato foi evidenciado em estudo transversal realizado em sete hospitais americanos que fez a associação de escores de cultura de segurança com a redução das taxas de ISC em cirurgias de cólon (FAN; PAWLIK; DANIELS et al., 2016).

Incidência de ISC menor que 10% foi verificada em revisão de literatura e os autores referem a hipótese de que pode existir a subnotificação dos casos, pois no Brasil, a maior parte dos hospitais realiza vigilância de ISC apenas no período da internação, e não inclui o acompanhamento e avaliação após a alta hospitalar (SANTOS; BAYLÃO; BORGES et al., 2015). A subnotificação de EA foi detectada nesta pesquisa nos três hospitais participantes, conforme já foi relatado no capítulo

6.1 sobre a Cultura de Segurança, pois em relação ao número de eventos notificados nos últimos 12 meses pelas três categorias profissionais (médicos assistentes, médicos residentes e equipe de enfermagem), os resultados evidenciaram dado preocupante sobre o predomínio de nenhum evento notificado. Culturalmente, pelo fato dos Serviços de Controle de Infecção Hospitalar realizarem a notificação ativa de casos, é possível que os profissionais não considerem necessário notificar os casos de IRAS. Potencialmente este é um tema a ser explorado em pesquisa futura.

Observou-se subnotificação de EA também em centro cirúrgico privado na capital do Rio Grande do Sul-Brasil. A subnotificação pode estar vinculada à falta de conhecimento dos profissionais em relação às consequências ao paciente e o medo da punição pela instituição (SILVA; OLIVEIRA JUNIOR; OLIVEIRA et al., 2015). Tais dados foram confirmados nesta pesquisa, nos três hospitais investigados. Desta forma, é importante relembrar sobre a avaliação da cultura de segurança , referida no capítulo 6.1, e cujos resultados com maior percentual de respostas negativas foram identificados na dimensão “Respostas não punitivas ao erro”, a qual investigou se funcionários sentem que seus erros e relatórios de erros não são usados contra eles e não são mantidos em seu arquivo pessoal.

Neste contexto, as notificações devem ser incentivadas, pois são estratégias que permitem modificações e planejamento de processos de trabalho mais seguros (SILVA; OLIVEIRA JUNIOR; OLIVEIRA et al., 2015). Além das notificações, é imprescindível que as informações registradas em prontuários sejam completas e capazes de descrever, com clareza, a história da doença, comorbidades, tratamentos ofertados, cuidados realizados, evolução apresentada e a continuidade da assistência aos pacientes (PARANAGUÁ; BEZERRA; SANTOS et al., 2014; NOVARETTI; SANTOS; QUITÉRIO et al., 2014). Deficiências nos registros dos dados interferem nas avaliações da ocorrência de EA cirúrgicos, fato que foi verificado em estudo que mostrou que em 85,9% (n=644) das internações de pacientes submetidos à intervenção cirúrgica, 24,5% (n=184) não foram registrados aspectos essenciais sobre a evolução diária da ferida operatória (PARANAGUÁ; BEZERRA; MOREIRA et al., 2016).

A avaliação de indicadores da assistência cirúrgica que evidenciam as causas dos EA torna-se fundamental nas organizações de saúde (BERRÍOS-TORRES; UMSCHEID; BRATZLER et al., 2017; CDC, 2017a). Os indicadores são

sinalizadores que identificam a ocorrência de possíveis desvios e a necessidade de revisão de ações assistenciais para resolução de problemas, a fim de interligar estratégias, recursos e processos organizacionais (DIAS; MARTINS; HADDAD, 2014). Deste modo, os profissionais precisam repensar suas ações e, as instituições de saúde sistematizar o processo de avaliação destes indicadores em busca da qualidade e segurança dos pacientes na prática clínica (PARANAGUÁ; BEZERRA; MOREIRA et al., 2016).

Diante do exposto, e da elevada prevalência de EA cirúrgico relacionados à ISC detectada, sugere-se a implementação contínua de educação continuada aos profissionais para a assistência cirúrgica segura, a fim de prevenir falhas técnicas e eventos relacionadas ao manejo clínico, bem como, durante os cuidados prestados. Outra estratégia importante é o incentivo dos registros completos e fidedignos da evolução do paciente, os quais podem contribuir para as investigações destes agravos, gerenciamento de riscos, planejamento de estratégias e ações de melhoria, a fim de minimizar a ocorrência de erros e prevenir futuras complicações.

No Hospital C, em relação ao tipo de EA cirúrgico relacionados à IRAS, observa-se que a ocorrência de Sepse/Choque Séptico ficou em primeiro lugar na estatística, e foi equivalente à taxa observada para ISC, ou seja, igual a 16,4% (n=9). Tal evento ficou em segundo lugar em termos de prevalência no Hospital B, com taxa de 12,7% (n=10) e na quarta posição no Hospital A, com taxa igual a 5,6% (n=3).

A Sepse é definida como disfunção orgânica com risco de vida causada por resposta desregulada do hospedeiro à infecção, e está associada à mortalidade hospitalar superior a 10%. Choque séptico é definido como um subconjunto da sepse, no qual anormalidades circulatórias, celulares e metabólicas estão associadas ao risco maior de mortalidade. Pacientes com choque séptico podem ser clinicamente identificados devido a necessidade de vasopressores para manter pressão arterial média de 65 mm Hg, ou superior, e nível de lactato sérico superior à 2 mmol / L (> 18 mg / dL), na ausência de hipovolemia. O choque séptico está associado às taxas de mortalidade hospitalar superiores a 40% (SINGER; DEUTSCHMAN; SEYMOUR et al., 2016).

Evidencia-se que Sepse/Choque séptico é causa de elevado índice da morbidade e mortalidade (VICENT; MARSHALL; NAMENDYS-SILVA et al., 2014; LEVY; RHODES; PHILLIPS et al., 2015; RHODES; PHILLIPS; BEALE et al., 2015).

Nesta questão, sistemas de saúde norte-americanos e órgãos governamentais responsáveis desenvolveram estratégias em grande escala para melhorar o tratamento, tanto em ações de melhoria da qualidade sob orientação clínica, como pelo desenvolvimento de políticas de saúde nos níveis regional e nacional (BARBASH; KAHN; THOMPSON, 2016; HERSHEY; KAHN, 2017).

Metanálise realizada em 27 estudos de sete países de alta renda, em quatro continentes, estimou a incidência e mortalidade mundial de sepse nos últimos 36 anos, correspondendo à 437 para sepse e 270 para sepse grave por 100.000 pessoas-ano; a mortalidade hospitalar foi de 43% para sepse durante esse período. Os autores relatam que os dados epidemiológicos são escassos e inexistentes nos países de baixa e média renda, por isso destacam a urgente necessidade de implementação de estratégias globais para mensurar indicadores de morbimortalidade, particularmente nestes países (FLEISCHMANN; SCHERAG; ADHIKARI et al., 2016). Neste aspecto, tal recomendação inclui os hospitais brasileiros.

A literatura mostra que no Brasil a mortalidade por sepse em hospitais públicos, vinculados ao SUS, é muito elevada e acima da média mundial (SANTOS; FERRAZ; CHAGAS, 2016). Outro estudo brasileiro, que teve por objetivo caracterizar o perfil epidemiológico das autorizações de internação hospitalar por Sepse em Alagoas, no período de 2012 a 2017, mostrou incidência de 66,2 para cada 100.000 habitantes. Maior índice foi identificado nos anos de 2016 e 2017, equivalente a 80,9 para cada 100.000 habitantes (SILVA; FARIA; GUIMARÃES et al., 2019). No país, dados analisados de 2006 a 2015 demonstraram que a incidência anual de sepse aumentou para 50,5% (de 31,5 / 100.000 para 47,4/100.000 pessoas por ano). A mortalidade aumentou para 85% (de 13,3/100.000 para 24,6/100.000 pessoas por ano) e a letalidade apresentou índice crescente para 23% (de 42,7% para 51,1%), no mesmo período (QUINTANO; HAMACHER; JAPIASSÚ et al., 2018).

Análise de 507 EA em unidades cirúrgicas de 63 hospitais da Suécia resultou em taxa de 13,2% (n=30) de casos com sepse/choque séptico (NILSSON; RISBERG; MONTGOMERY et al., 2016). Nos Estados Unidos, as taxas de incidência de sepse foram evidenciadas em 535 casos por 100.000 pacientes ao ano e permanecem com estimativas crescentes (WALKEY; LAGU; LINDENAUER, 2015).

A mortalidade hospitalar naquele país persiste em altos índices, entre 25 e 30% (COHEN; VINCENT; ADHIKARI et al., 2015).

Estudo de coorte retrospectivo que usou dados de alta hospitalar de 2012 e 2013, sobre cuidados agudos gerais na Pensilvânia-Estados Unidos, identificou pacientes adultos com sepse em 152 hospitais, com os seguintes focos: bacteremia, pneumonia, trato urinário, pele/tecidos moles e gastrointestinal. Em 2012 foram encontrados 115.213 casos e, em 2013 foram identificados 120.941. Deste modo, a taxa geral de mortalidade não ajustada devido a Sepse/Choque Séptico em 30 dias foi de 18,5% em 2012 e 18,2% em 2013 (DARBY; DAVIS; BARBASH et al., 2019).

Seu diagnóstico assertivo e precoce possibilita a implantação do tratamento nas primeiras seis horas após atendimento, com provável redução de mortalidade (SILVA; FARIA; GUIMARÃES et al., 2019). Deste modo, a sepse é reconhecida como importante problema de saúde pública da atualidade, e evidencia a necessidade da implementação de estratégias de enfrentamento eficientes, bem como, ações preventivas (COOKE; IWASHYNA, 2014; GOULART; FERREIRA JÚNIOR; SARTI et al., 2019).

Os estudos descritos acima mostram como esta patologia é considerada grave, potencialmente fatal e com incidência global, portanto, infere-se que ações de educação continuada dos profissionais para identificação de sinais e sintomas preditivos, diagnóstico e tratamento precoces, bem como análise das causas representem medidas eficazes de prevenção.

Observa-se que no Hospital A Hematoma/Seroma, ficou em segundo lugar na prevalência, com 18,9% (n=10). No Hospital B a segunda posição foi para Deiscência de sutura em 12,7% (n=10) que por sua vez, alcançou a terceira posição no Hospital A, em 17% (n=9) dos casos analisados. A formação de hematoma e seroma é caracterizada pelo acúmulo de líquido seroso no espaço morto, entre as áreas destacadas durante a cirurgia (CAMMAROTA; RIBEIRO JÚNIOR; LIMA et al., 2016; MOHAMMAD, 2019). No exame clínico, o seroma é detectado pela presença de abaulamento e flutuação do tecido, e sua origem é multifatorial, pois inclui a drenagem de vasos linfáticos e sanguíneos que normalmente drenam fluidos intersticiais (CAMMAROTA; RIBEIRO JÚNIOR; LIMA et al., 2016).

A ocorrência desses EA apresentou taxas menores em relação aos resultados desta pesquisa, quando comparado a estudo espanhol, com 8,9% dos pacientes submetidos a tratamento cirúrgico, especificamente na especialidade de

Cirurgia Geral (ZAPATA; SAMANIEGO; CUÉLLAR et al., 2015). Para que ocorra a cicatrização do sítio cirúrgico são indicados os pontos dérmicos que nem sempre eliminam completamente a formação do espaço morto na área cirúrgica, principalmente se a excisão se estender à gordura profunda ou fáscia. A aspiração de líquidos no local da cirurgia no pós-operatório é o resultado de eventos que, por sua vez, prejudicam a cicatrização de partes moles. Estudo concluiu que o procedimento técnico auxiliar na prevenção da ocorrência de hematoma e seroma refere-se à colocação de pontos mais profundos, como os realizados em suturas fasciais, porém, esta técnica exige do cirurgião mais tempo para sua realização (MOHAMMAD, 2019).

Estudo encontrou dado relevante quanto à hipertensão arterial como fator de risco estatisticamente significativo (p =0,031) nos pacientes com hematoma e seroma em sítio cirúrgico (CAMMAROTA; RIBEIRO JÚNIOR; LIMA et al., 2016), assim como, foi evidenciado na maioria dos pacientes com EA cirúrgicos desta pesquisa. Este fator de risco, associado às falhas na técnica cirúrgica, pode explicar alta incidência de Hematoma/Seroma e outras complicações identificadas nos hospitais avaliados.

Em relação à ocorrência de Deiscência de sutura, é importante descrever seu conceito, pois trata-se de abertura da ferida que foi previamente suturada, com extravasamento de secreção serosa sanguinolenta no local da incisão cirúrgica. Classifica-se como parcial, quando apresenta alguns centímetros de abertura, ou como completa. A causa pode estar relacionada ao excesso de tensão na sutura, infecção ou hematoma (JESUS, 2018). Taxas inferiores ao relatado nesta pesquisa foram detectadas em outro estudo brasileiro que demonstrou ocorrência de 3,67% (n=8) dos casos analisados (PARANAGUÁ, BEZERRA, SILVA et al., 2013) e em estudo americano que encontrou apenas dois casos em 676 EA cirúrgicos avaliados (KENNERLY; KUDYAKOV; GRACA et al., 2014).

A ocorrência de deiscência de sutura mobiliza toda a equipe de saúde para prestação de assistência específica e está associada ao tempo de internação, custos e riscos. Estudo retrospectivo em prontuários realizado em duas unidades de internação cirúrgica geral, de hospital universitário de grande porte, analisou 10 casos de deiscências, na maioria mulheres, com período registrado de ocorrência a partir de 96 horas de pós-operatório. Este detectou que a região abdominal foi o sítio cirúrgico mais prevalente; os pesquisadores concluíram que não houve uniformidade

de procedimentos e cuidados realizados pela equipe médica e de enfermagem (MARQUES; ALMEIDA; FARIAS et al., 2016).

A utilização de protocolos clínicos para equipe médica e planos operacionais padronizados para a equipe de enfermagem, bem como, o uso de produtos e coberturas específicas, instituição de comissão de curativos ou equipe especializada para o cuidado das feridas, podem direcionar a tomada de decisão mais acertada e segura para cada paciente. Neste sentido, destaca-se a importância de pesquisas para avaliar o perfil de pacientes com deiscência no local do sítio cirúrgico, gerenciamento de casos no ambiente hospitalar, uniformização de técnicas cirúrgicas e condutas de tratamento, bem como, medidas preventivas.

Dados diferentes dos detectados no Hospital A e B foram evidenciados no Hospital C, em relação à segunda e terceira posições na prevalência de EA cirúrgicos, pois, foram identificados a ocorrência de Fístulas (liquórica, entérica ou pancreática) em 9,1% (n=5) e Infecção do trato Urinário em 7,3% (n=4). As Fístulas também foram identificadas no Hospital A com prevalência de 3,8% (n=2) e no Hospital C em 7,7% (n=6). A Infecção do Trato Urinário representou 2,6% (n=2) no Hospital B e não foi registrada sua ocorrência no Hospital A.

As Fístulas pós-operatórias representam grave complicação e os fatores predisponentes envolvem o aumento da pressão intraluminal devido à estenose distal, tensão excessiva na linha da sutura, isquemia tecidual e hematoma (PÉRISSÉ; PÉRISSÉ; BERNARDO JÚNIOR et al., 2015). Assim como apresentado nesta pesquisa, dados semelhantes foram demonstrados em estudo transversal, retrospectivo e de caráter analítico realizado em hospital universitário, para verificar a prevalência e os fatores associados às complicações pós-operatórias de sítio cirúrgico. Porém, em pacientes submetidos às cirurgias bariátricas; e revelou taxas de 6,75% (n=3) de ocorrência de fístulas (AGUIAR; GOMES; SANTOS et al., 2018).

Quanto à Infecção de Trato Urinário (ITU), que faz parte das IRAS detectadas nesta pesquisa, a literatura aponta que, aproximadamente, 80% estão associadas ao uso de cateter vesical. No entanto, este risco aumenta com a duração do cateterismo, pois pode chegar a 5% para cada dia de uso. Assim, após 28 dias de cateterização, o risco eleva-se para 100%, o que resulta em, aproximadamente, 4% dos pacientes que apresentam evolução para outras patologias como sepse secundária à infecção, com taxa de mortalidade estimada em até 30%

(TALESCHIAN-TABRIZI; FARHADI; MADANI et al., 2015; TENKE; MEZEI; BODE et al., 2017).

Estudo epidemiológico realizado em Unidade de Terapia Intensiva em Minas Gerais, para identificar os fatores relacionados à ocorrência de ITU, demonstrou que a maioria dos pacientes procedia do pronto socorro (43,8%) para especialidades cirúrgicas (56,2%), com prevalência de número de internações da especialidade cirúrgica neurológica. A maioria das inserções realizadas em pacientes cirúrgicos não possuía indicações adequadas (72,9%) e o tempo de permanência do cateter vesical (média de 5,7 dias) foi maior do que o indicado pelas diretrizes de prevenção de infecção. (MOTA; OLIVEIRA, 2019). Essas recomendam o uso desse dispositivo em
cirurgias específicas, ou seja, que sua remoção aconteça o mais rapidamente possível, de preferência dentro de 24 horas, a menos que haja outras indicações para a sua permanência (LO; NICOLLE; COFFIN et al., 2014; DUDECK; EDWARDS JÚNIOR; ALLEN-BRIDSON et al., 2015). A literatura descreve que muitos cirurgiões acreditam que o cateterismo seja parte padrão das cirurgias, porém, os achados revelam que os pacientes consideram este procedimento invasivo (DUDECK; EDWARDS JÚNIOR; ALLEN-BRIDSON et al., 2015), doloroso e desconfortável (COFEN, 2013; LEHMAN; DONLAN, 2015). Neste contexto, os esforços para limitar tal procedimento são considerados como solução viável (DURANT, 2017). Outras ações recomendadas para a prevenção de ITU incluem o manejo adequado de cateter, elaboração de estratégias apropriadas de prevenção (LO; NICOLLE; COFFIN et al., 2014), realização de técnica asséptica para inserção de cateter e cuidados prestados com higiene e segurança durante o esvaziamento e troca de bolsas (CDC, 2017b). O Conselho Federal de Enfermagem (COFEN) determina esse procedimento como privativo ao enfermeiro (COFEN, 2013).

Ressalta-se que no Hospital A também foram detectadas complicações pós-operatórias relacionados a Retenção Urinária, com taxa equivalente a 5,6% (n=3) e Intoxicação por Opióide 1,9% (n=1). Tais EA foram considerados potencialmente não evitáveis, pois estavam relacionadas ao uso de opióides durante a anestesia ou analgesia pós-operatória por cateter peridural para controle da dor aguda no pós-operatório. E, consequentemente, esses fármacos causaram retenção urinária, demandando cateterismo vesical para resolução do problema; conclui-se que estes fatores limitam o potencial de evitabilidade.

Com relação as demais IRAS evidenciadas nesta pesquisa, os dados mostram a ocorrência de Pneumonia, conforme as respectivas taxas: Hospital B=2,6% (n=2) e Hospital C=3,6% (n=3). Tais resultados foram superiores ao encontrado em estudo brasileiro que recrutou 59 hospitais para avaliar complicações pulmonares no pós-operatório, entre estas, a pneumonia associada a tratamento com antibióticos, que foi equivalente a 1,6% (n=40) (CABRAL; SILVA; BORGES, 2014).

A avaliação da duração de internação apontou para longa permanência hospitalar em pacientes com EA cirúrgicos pulmonares, com média de 12 dias, enquanto pacientes que não sofreram complicações tiveram média de três dias de internação. A taxa de mortalidade em 30 dias de pós-operatório foi de 35 pacientes, dos quais 24 tiveram complicações respiratórias, o que representou 19,5% dos 123 pacientes detectados com complicações pulmonares (CABRAL; SILVA; BORGES, 2014).

Logo, as complicações pulmonares pós-operatórias são comuns, dispendiosas e aumentam a mortalidade dos pacientes. Alterações no sistema respiratório ocorrem imediatamente após a indução da anestesia geral, a movimentação respiratória e a função muscular são alteradas. Adicionalmente, os volumes pulmonares são reduzidos e a atelectasia se desenvolve em >75% dos pacientes que recebem um medicamento bloqueador neuromuscular. O sistema respiratório pode levar seis semanas para retornar ao seu estado pré-operatório após anestesia geral em grandes cirurgias (MISKOVIC; LUMB, 2017).

Em 2015, uma força-tarefa conjunta européia publicou diretrizes para definições de resultados clínicos perioperatórios e considerou a infecção respiratória, insuficiência respiratória, derrame pleural, atelectasia, pneumotórax, broncoespasmo, pneumonite por aspiração às medidas compostas; pneumonia definida, síndrome do desconforto respiratório agudo e embolia pulmonar como EA (JAMMER; WICKBOLDT, N.; SANDER et al., 2015). A incidência de complicações pulmonares perioperatória em grandes cirurgias variou de <1 a 23% (BABLEKOS; MICHAELIDES; ANALITE et al., 2014; YANG; TENG; LEE et al., 2015; CANET; SABATÉ; MAZO et al., 2015). Nesta pesquisa, além da Pneumonia, foram identificadas outras complicações pulmonares como Broncoaspiração (Hospital C=1,8%;n=1), Pneumotórax (Hospital B=2,6%;n=2 e Hospital C=3,6;n=2) e Hipóxia (Hospital C=5,6%;n=3).

Os efeitos respiratórios adversos da anestesia geral se iniciam assim que o paciente perde a consciência e o impulso respiratório central é deprimido, o que prejudica as respostas contra a hipóxia (LUMB, 2016). Deste modo, a hipóxia também é classificada, por alguns estudos, como complicação pulmonar perioperatória (GROSSE-SUNDRUP; HENNEMAN; SANDBERG et al., 2012).

Além dos EA cirúrgicos já referidos, como Hematoma/ Seroma, Deiscência de Sutura e Fístulas, outros EA cirúrgicos relacionados a falhas técnicas foram evidenciados, como Perfuração/Laceração, Necrose de parede e Eventração.

A perfuração no intraoperatório pode atingir diversos órgãos e sistemas, como parede abdominal e torácica, vísceras e órgãos, vasos sanguíneos, articulações, implantes, além de outras estruturas anatômicas (CANTON; NOGUEIRA; FERREIRA et al., 2019). Estudo realizado sobre EA cirúrgicos graves observou taxa de 21,05% (n=4) relacionada à lesão de órgãos digestórios durante cirurgias videolaparoscópicas, como jejuno, fígado e estômago. Os autores relatam que, especificamente, a lesão de jejuno refletiu no aumento do tempo de internação e cirurgia para realização de ileostomia, o que causou impacto na vida do paciente (ARAÚJO; CARVALHO, 2018).

Nos Estados Unidos, em hospital do Texas, as lacerações representaram 7,1% (n=48), destas 6,5% foram consideradas preveníveis; quase a metade dos casos causaram prolongamento da internação hospitalar (KENNERLY; KUDYAKOV; GRACA, 2014). Nesta pesquisa, Perfuração/Laceração representou 3,9% (n=3) no Hospital B para perfuração intestinal e 1,8% (n=1) para perfuração de intestino delgado por cautério.

A complicação cirúrgica Eventração se caracteriza como a protrusão/projeção de conteúdos abdominais através de uma região enfraquecida da parede abdominal, seja por trauma ou como resultado de incisões cirúrgicas. É uma complicação frequente em cirurgias abdominais, ocorrendo em 2% a 35% das laparotomias (FIKATAS; SCHOENING; LEE et al., 2013). Os achados desta pesquisa demostraram um caso de eventração no Hospital B que representou taxa de 1,3%.

Ocorrência de Hemorragia foi registrada nos Hospitais B e C e não foi detectada no Hospital A, bem como, Choque Hemorrágico foi evidenciado apenas no Hospital B. Os Hospitais B e C prestam cuidados agudos com porta de entrada via pronto socorro para pacientes encaminhados via Serviços de Atendimentos

Médicos de Urgência ou demais ambulâncias provenientes das Unidades de Pronto Atendimento (UPA). Neste contexto, a hemorragia é a maior causa de morte potencialmente prevenível após um trauma, em cuja fase aguda deve-se priorizar a interrupção do sangramento (BRANDAO; MACEDO; RAMOS, 2019).

A hemorragia pode ocorrer durante ou após a cirurgia, e apresenta como fatores etiológicos a hemostasia ineficiente ou ruptura de alguma ligadura. Sua consequência clínica está correlacionada ao calibre do vaso, do tipo de sangramento e da quantidade de sangue perdida numa determinada fração de tempo. A hipovolemia, por sua vez, coloca em risco a vida do paciente, depende de diagnóstico precoce e hemostasia eficaz e, frequentemente, da necessidade de reoperação do paciente (STRACIERI, 2008).

Como possível evolução, o choque hemorrágico ocorre em virtude de perdas sanguíneas para o meio externo ou para o meio interno (hemotórax, hemorragia digestiva, hemoperitônio, entre outros), consiste em estado de má perfusão tecidual, sendo o tipo hemorrágico o mais comum em vítimas de trauma. Nessas circunstâncias, utilizam-se estratégias para garantir oferta tecidual de oxigênio, além de prevenir e tratar coagulopatias. Outro ponto fundamental é a adesão aos protocolos de tratamento que devem servir como pilar para o planejamento das condutas (BRANDAO; MACEDO; RAMOS, 2019).

Nesta pesquisa evidenciou-se as seguintes taxas de Hemorragia / Choque Hemorrágico: Hospital B foi de 7,7% (n=6) e Hospital C foi de 5,6% (n=3). Taxas superiores foram relatadas em estudo americano que mostrou prevalência de 35,7% (n=241), a grande maioria (98,3%) foi considerada possivelmente evitáveis (KENNERLY; KUDYAKOV; GRACA et al., 2014).

No Brasil, investigadores pioneiros nos estudos de prevalência de EA cirúrgicos apontaram a ocorrência de hemorragia em 12,2% (n=5) dos pacientes avaliados (MOURA; MENDES, 2012). Na assistência cirúrgica ambulatorial identificou-se hemorragia em 5,20% dos casos, após avaliação de 55.879 pacientes operados num hospital dia entre os anos de 2010 e 2014 (COSTA; MOREIRA; GUSMÃO, 2017). Outro estudo brasileiro, desenvolvido em hospital privado de São Paulo, identificou dois EA graves relacionados ao choque hemorrágico, que representou taxa de 10,53%. Os autores relatam que um destes eventos ocorreu durante cirurgia eletiva de Revascularização de Miocárdio, devido desconexão

acidental do circuito de circulação extracorpórea, com perda de sangue significativa (ARAÚJO; CARVALHO, 2018).

A Trombose Venosa Profunda (TVP) foi evidenciada em 1,9% (n=1) no Hospital A e em 1,3% (n=1) no Hospital B. Tais dados corroboram com estudo realizado no Japão que identificou taxas de 1% (n=3) (SHIOZAKI; MORIMATSU; MATSUSAKI et al., 2016). Em Portugal, em 67.635 internamentos cirúrgicos identificou-se 90 casos de tromboembolismo venoso (TEV) no pós-operatório, o que correspondeu ao risco de 1,33/1000 episódios. Entre as especialidades cirúrgicas avaliadas, a Neurocirurgia apresentou maior risco, seguida da Urologia e Cirurgia Ge ral e, a especialidade com menor risco, foi a Cirurgia Plástica (AMARAL; PEREIRA; MORETO et al., 2017).

Embora seja reconhecido o elevado risco de TEV em cirurgia de grande porte realizada na especialidade de Ortopedia (AMARAL; PEREIRA; MORETO et al., 2017), os resultados desta pesquisa não demonstraram ocorrência desse EAe/ou embolia nesta especialidade, mesmo nas análises dos registros de retornos ambulatoriais. Tais dados podem ser explicados pelo avanço nas terapias preventivas, como a profilaxia medicamentosa, deambulação precoce e uso de meias compressivas, bem como, em razão do tipo de anestesia realizada.

As anestesias do neuroeixo (espinhal, epidural e caudal) que também podem ser combinadas (geral e regional) foram equivalentes a 58,6% no Hospital A, 17,6% no Hospital B e 51,5% no Hospital C. Dados evidenciados em metanálise demonstraram que a anestesia peridural modifica, radicalmente, o risco destes eventos embólicos, com reduções de 44% para TEV e 55% para Embolia Pulmonar (RODGERS; WALKER; SCHUG et al., 2000). Este fato pode contribuir para a explicação da redução de risco na Ortopedia, onde a anestesia do neuroeixo é a técnica mais utilizada nas cirurgias de grande porte de membros inferiores (AMARAL; PEREIRA; MORETO et al., 2017).

Frente à ocorrência de Hemorragia/Choque hemorrágico, TVP e embolias utilizam-se estratégias para garantir oferta tecidual de oxigênio, prevenção e tratamento das coagulopatias, cirurgias para reparação de danos. Outro ponto fundamental é a adesão aos protocolos de profilaxia e tratamento, os quais devem servir como pilar para o planejamento das condutas e prevenção (BRANDÃO; MACEDO; RAMOS, 2019).

A ocorrência de Nefropatia foi verificada em 1,9% (n=1) no Hospital A, Insuficiência Renal Aguda em 5,15% (n=4) no Hospital B, e em 3,6% (n=2) no Hospital C. Taxas superiores foram verificadas em estudo brasileiro realizado em hospital público de São Paulo, em pacientes submetidos a cirurgias não cardíacas internados na unidade de terapia intensiva, que verificou o desenvolvimento de lesão renal aguda em 35,7% (n=35) e destes, 68,57% (n=24) necessitaram de diálise. O risco anestésico elevado nas cirurgias eletivas, intercorrências no intraoperatório e presença da doença renal crônica foram fatores que influenciaram na lesão renal (PEREIRA; BADAOUI; SOEIRO et al., 2016).

Neste contexto, lesão renal aguda perioperatória é mais comum do que se reconheceu anteriormente, especialmente em pacientes de alto risco submetidos a procedimentos de maior risco. É fundamental identificar pacientes em risco, e estabelecer estratégias preventivas que incluam a prevenção de agentes nefrotóxicos e a hiperglicemia, otimização da hemodinâmica, restauração do volume circulante e instituição de monitoramento hemodinâmico (MEERSCH; SCHMIDT; ZARBOCK, 2017).

Por sua vez, a Insuficiência Renal Aguda ocorre quando há perda súbita da filtração glomerular por algumas horas ou mais lentamente, durante alguns dias; e as consequências são o acúmulo de escórias nitrogenadas e o desequilíbrio eletrolítico no organismo do indivíduo (LI; BURDMANN; MEHTA, 2013; SCHMIDT; WIESE; PEREIRA et al., 2018). Esta patologia está diretamente relacionada às taxas de morbimortalidade hospitalar em curto e longo prazos (MEERSCH; SCHMIDT; ZARBOCK, 2017).

Nos Estados Unidos estudo evidenciou que nove pacientes apresentaram Lesão Renal/Insuficiência Renal (1,33%) e quatro pacientes foram acometidos de Infarto Agudo de Miocárdio (0,59%). (KENNERLY; KUDYAKOV; GRACA et al., 2014). Problemas cardíacos graves também foram evidenciados nesta pesquisa e considerados EA cirúrgicos pois, acometeram um paciente que sofreu Infarto Agudo do Miocárdio (IAM) no pós-operatório (1,8%) no Hospital C e um paciente que sofreu Parada Cardiorrespiratória (PCR) intraoperatória (1,3%).

Em relação à população mundial, número crescente de pacientes com múltiplas comorbidades tem sido submetido a operações não cardíacas, com consequente aumento das complicações cardiovasculares associadas. Deste modo o IAM perioperatório pode se tornar um problema frequente. No Brasil, tal evidência

não é diferente, mesmo com os avanços nas técnicas cirúrgicas e anestésicas. A ocorrência de IAM perioperatório prolonga a necessidade de terapia intensiva e a estadia hospitalar, aumenta o custo da internação e diminui a sobrevida a longo prazo (GUALANDRO; CALDERARO; YU et al., 2012). Estudo revelou que a incidência de PCR no intraoperatório variou de 2,56 a 44 casos por 10 mil procedimentos; os autores descrevem que este tipo de complicação é o evento mais grave, potencialmente reversível, que pode ocorrer durante o procedimento cirúrgico (VANE; NUZZI; ARANHA et al., 2016).

Nos prontuários analisados alguns dos EA cirúrgicos confirmados foram encontrados de forma específica em determinado hospital. No Hospital B identificou-se Pneumoencéfalo Hipertensivo em um paciente cirúrgico, o que representou taxa de 1,3%, assim como, eventos classificados na categoria “Medicação/fluídos endovenosos”, como Insuficiência Adrenal e Reação à medicação, ambos com taxas equivalentes de 1,3% (n=1). E evento classificado na categoria “Recursos/gestão organizacional” relacionado ao Cancelamento de sala cirúrgica, que representou taxa de 1,3% (n=1). No Hospital C detectou-se problema relacionado à “Infraestrutura/local/instalações”, como a Falha na assepsia do ambiente cirúrgico, evidenciado em 1,8% (n=1) dos casos analisados.

O Pneumoencéfalo hipertensivo é definido como uma coleção intracraniana de ar, geralmente associada a lesão traumática craniofacial (75% dos casos) ou a iatrogenias, e é considerado emergência neurocirúrgica (PARREIRA; PONTES; ANTONUCCI et al., 2018). Insuficiência Adrenal pode ser desencadeada por deficiência na síntese de cortisol, bem como, ocorrer em pacientes em situações de estresse. Existem medicamentos que interferem na função adrenal e o uso concomitante reduz as concentrações de corticosteroides (CASTRO, FREIXES; NOVOA et al., 2019). Esse é considerado urgência médica, potencialmente letal, geralmente é subdiagnosticado devido à inespecificidade de sintomas, porém, o diagnóstico e tratamento com glicocorticoides é vital (BANCOS; HAHNER; TOMLINSON et al., 2015; CASTRO, FREIXES; NOVOA et al., 2019).

A Reação adversa a medicamentos (RAM) é definida pela OMS como qualquer resposta prejudicial, ou indesejável, e não intencional a medicamento em doses normalmente utilizadas no homem para profilaxia, diagnóstico, tratamento de doença ou para modificação de funções fisiológicas (WHO, 2005). Esse EA pode ser melhor referido como efeito não desejado atribuível à administração de

medicamento (ORGANIZAÇÃO PAN-AMERICANA DA SAÚDE (OPAS), 2011). Sua prevenção faz parte do terceiro Desafio Global de Segurança do Paciente da OMS, denominado "Medicação sem danos", o qual propõe soluções para garantir a segurança das práticas de medicação, e alcançar o engajamento e comprometimento dos Estados Membros e órgãos (WHO, 2017).

EA relacionados à medicação foram identificadas em estudo que avaliou incidentes em clínica cirúrgica e os dados mostram que foram associados às reações
adversas e alérgicas, resultando em adição ou substituição da terapia medicamentosa (PARANAGUÁ, BEZERRA; SILVA et al., 2013). Fato semelhante ocorreu em paciente no Hospital B, devido ao uso de antibiótico Meropenem com ocorrência de dano leve.

Análise das suspeitas de reações adversas a medicamentos notificadas no sistema de farmacovigilância do Brasil (NOTIVISA-medicamento) entre 2008 e 2013 identificou 26.554 notificações, o que representou taxa de notificação de 22,8 milhões de habitantes/ano. Prevaleceram as reações adversas graves (59,2%), com destaque para as que resultaram em efeito clinicamente importante (83,1%); os medicamentos mais frequentemente associados foram os agentes neoplásicos e imunomodulares (32,1%) e anti-infeciosos gerais para uso sistêmico (27%), ao passo que o sistema-órgão-classe mais afetado foi o relacionado à afecções da pele e distúrbios afins (23,7%) (MOTA; VIGO; KUCHENBECKER, 2019). Nesta pesquisa, a paciente afetada por RAM no Hospital B apresentou exantema, precisou ser medicada com corticóides endovenosos e teve suspenso o uso do antibiótico.

Sobre o evento detectado no Hospital B, classificado na categoria "Recursos/gestão organizacional" denominado Cancelamento de sala Cirúrgica, verificou-se que a paciente foi vítima de acidente moto *versus* automóvel, com fratura em tornozelo, e teve sua cirurgia cancelada por três vezes por questões administrativas. As falhas de agendamento para realização do tratamento cirúrgico causaram danos a paciente, com posterior ISC e prolongamento do tempo de internação. A análise deste caso identificou falta de planejamento das ações administrativas para programação da cirurgia. Estudo desenvolvido em hospital público de São Paulo detectou taxa de cancelamento de cirurgias eletivas de 6,79%, porém, os pesquisadores descrevem que esta taxa pode ser reduzida para 1,36%, ao considerarem que 80% das razões para cancelamento são evitáveis (SANTOS;

BOCCHI, 2017). No Hospital B este EA cirúrgico foi identificado, representou taxa de 1,3% e foi considerado evitável, porém, o agravante esteve relacionado ao fato de se tratar de cirurgia de urgência.

Nesta pesquisa, analisa-se que a taxa identificada no Hospital B foi baixa (1,3%) e, tal fato, pode ser explicado pela omissão desta informação no prontuário e porque representou o percentual calculado de prontuários de pacientes submetidos a cirurgia que sofreram EA, diferente da maioria dos estudos publicados, que analisam esta variável como indicador de qualidade em relação a todos os pacientes internados

para cirurgia, independente da ocorrência ou não de EA cirúrgicos, geralmente eletivas, além de associarem causas clínicas e não clínicas. Investigação em hospital público do Distrito Federal, de janeiro a outubro de 2015, mostrou que foram programadas 6.926 cirurgias, das quais foram realizadas 4.587 e suspensas 2.339, o que representou taxa de suspensão cirúrgica de 33,8%. Os principais motivos de suspensão foram causas injustificadas em 30,1% e causas administrativas em 21,8% (GOMES. FRANCO; MORAIS et al., 2018). Taxas inferiores foram verificadas em hospital de ensino no nordeste brasileiro que foi de 19,5% (CARVALHO; SOBRAL; MARINHO; RADUNZ; TOURINHO et al., 2016).

Segundo a literatura, essas altas taxas são comuns em hospitais públicos (MACEDO; KANO; BRAGA et al., 2013), e constituem avaliação negativa para a instituição (GOMES. FRANCO; MORAIS et al., 2018), fato que remete aos resultados analisados sobre as dimensões da cultura de segurança que, em sua maioria, se demonstraram frágeis ou negativas nos três hospitais investigados. Neste sentido, os inúmeros transtornos causados devido à suspensão de cirurgias podem ser analisados por duas vertentes: com base nas repercussões geradas para o indivíduo e seus familiares, e outra para avaliar as consequências causadas para a instituição de saúde e os profissionais (HORI; NAKAYAMA; SAKAMOTO, 2016). Tal fato foi constatado no Hospital C em relação à instituição e à paciente vítima do dano decorrente da assistência à saúde e seus familiares.

Alguns dos principais motivos para suspensão dos procedimentos cirúrgicos na literatura são: questões administrativas e estruturais; condições clínicas inadequadas; atrasos da equipe cirúrgica; falhas de comunicação entre equipe médica, CC e unidades de internação; falta de insumos necessários; falhas no pré-operatório e falta de exames previamente solicitados (CALLEGARI; BATISTA;

SILVEIRA et al., 2015; CARVALHO; SOBRAL; MARINHO et al., 2016). Deste modo, os fatores determinantes para sua ocorrência devem ser rigorosamente controlados e restringidos (GOMES; FRANCO; MORAIS et al., 2018). O monitoramento dos indicadores relacionados à suspensão de cirurgia deve ser contínuo para subsidiar o planejamento de estratégias de intervenção e minimização dos transtornos causados para os clientes, familiares e instituição (CARVALHO; SOBRAL; MARINHO et al., 2016).

Evento cirúrgico denominado Falha na assepsia do ambiente cirúrgico, ocorrido no Hospital C, foi classificado como problemas com "Infraestrutura/local/instalações" e aconteceu com paciente do sexo feminino, vítima de acidente moto *versus* automóvel que necessitou ser operada duas vezes para tratamento de fratura subtrocanteriana à esquerda. Durante a realização da segunda cirurgia, que teve três dias de intervalo da primeira, foi registrado em prontuário a presença de duas moscas dentro da sala. Em decorrência deste fato, houve prolongamento do tempo de internação para administração de antibioticoterapia endovenosa por cinco dias, e o tratamento foi mantido por via oral após alta por sete dias.

O Centro Cirúrgico (deve ficar próximo das unidades de internação cirúrgicas, da Unidade de Terapia Intensiva e de emergência para que o atendimento dos pacientes possa ser agilizado. Por ser considerado zona crítica para transmissão de infecção hospitalar, deve ficar longe do fluxo de pessoas, de poeira e outros contaminantes (BRITO, 2006). Neste contexto, não deve apresentar falhas em suas instalações e funcionamento, inclusive quanto às rotinas de limpeza e assepsia, pois, podem causar complicações pós-operatórias. Tais falhas podem envolver recursos humanos, tecnológicos e materiais, bem como, de planejamento inadequado de sua infraestrutura, manutenção e segurança (MEDEIROS, ARAÚJO FILHO, 2017; RIBEIRO; MATTOS, MORAIS et al., 2019).

Nesta pesquisa, EA diferentes, porém classificados na mesma categoria de "dispositivo/equipamento médico" ocorreram nos Hospitais B e C. Evidenciou-se no Hospital B taxa de 1,3% em relação a Problema técnico no microscópio; 1,3% por Falha no material de implante; 1,3% devido a Perda da jejunostomia e 1,3% em relação a Reposicionamento do dispositivo de Derivação Ventricular (DVE). No Hospital C, identificou-se taxa de 3,6% devido a Falha no material de implante; 3,6%

em razão da Luxação de Prótese Total de Quadril e 1,8% correlacionada à Quebra de material de síntese.

Os EA cirúrgicos denominados Perda da jejunostomia, Reposicionamento da DVE e Luxação de Prótese total de quadril foram problemas relacionados aos dispositivos, porém, associados às falhas na técnica cirúrgica, razão esta que já foi descrita anteriormente, em virtude dos danos preveníveis que acometem os pacientes, além do fato da necessidade de reoperações.

Os eventos nominados Problema técnico do microscópio, Falha no material de implante e Quebra de material de síntese foram associados a erros de fabricação e qualidade dos dispositivos. Neste sentido, os erros de fabricação se referem às fases de desenho e produção do equipamento, ou seja, aos erros conceituais ou quando o equipamento é produzido fora das especificações (SILVA; AMANTE, 2015; DUARTE; STIPP; SILVA et al., 2016). Tais erros são entendidos como falha dos equipamentos/dispositivos e considerado fator importante correlacionado aos danos originados nos pacientes (RIBEIRO; SILVA; FERREIRA et al., 2018).

A análise dos hospitais investigados identificou a ocorrência de outros eventos preveníveis decorrentes da assistência cirúrgica, como Lesão de pele e mucosa no Hospital A (5,6%;n=3); Lesão por pressão no Hospital B (1,3%;n=1) e Queda no Hospital C (1,8%;n=1).

As Lesões de pele ocasionadas no período intraoperatório podem variar desde eritema, que compromete a integridade da pele a lesões extensas, como queimaduras causadas por bisturi elétrico e acessórios, ou mesmo lesões por fricção ou cisalhamento, em decorrência da exposição cirúrgica sem os devidos cuidados de prevenção no posicionamento (SPRUCE, 2017). Pode se apontar outras causas, como tempo prolongado de cirurgia e imobilização do paciente, reação alérgica à adesivos médico-hospitalares ou procedimentos clínicos. No entanto, destaca-se que tais lesões são EA passíveis de prevenção. Taxas semelhantes de lesão de pele verificadas no Hospital A (5,6%) foram relatadas em outro estudo brasileiro realizado em hospital universitário que analisou sua ocorrência no período intraoperatório (4,5%). Dos 154 pacientes avaliados, sete apresentaram 11 lesões de pele, como lesões por pressão estágio I, estágio II e abrasão, predominantemente no glúteo e no tórax (BEZERRA; GALVÃO; VIEIRA et al., 2019). Escores superiores ao desta pesquisa foram verificados na Suécia, que analisou 507 EA em unidades cirúrgicas de 63 hospitais e constatou que as lesões de pele e

mucosas acometeram 31 pacientes (6,1%) e 94% foram considerados evitáveis (NILSSON; RISBERG; MONTGOMERY et al., 2016).

Os cuidados de enfermagem dedicados ao paciente no período intraoperatório refletirão no pós-operatório, pois muitas lesões de pele têm seu princípio na sala de operação que se intensificam na fase do pós-operatório (BEZERRA; GALVÃO; VIEIRA et al., 2019). Tal fato foi observado em um paciente que foi acometido por Lesão por Pressão estágio II no Hospital B e que representou taxa de 1,3% dos EA cirúrgicos analisados. As variáveis tempo do procedimento cirúrgico, tipo de anestesia e comorbidades são os principais fatores associados à ocorrência das lesões (BEZERRA; GALVÃO; VIEIRA et al., 2019). Danos originários dos cuidados em saúde como as lesões de pele são decorrentes da falta de proteção de proeminências ósseas e áreas de alta pressão (SPRUCE, 2017), assim como, déficit nas ações preventivas relativas à limpeza, hidratação da pele e mudança de decúbito, que são responsáveis pela ocorrência destes eventos preveníveis.

Nesta pesquisa, o evento Queda foi evidenciado no Hospital C e representou 1,8% dos EA cirúrgicos analisados. O paciente em questão tinha 51 anos de idade e sofreu queda de nível no período pós-operatório, no momento em que estava desacompanhado. Os registros em prontuário descrevem que a queda ocorreu no curto espaço de tempo que ele ficou sozinho no banheiro, quando se levantou da cadeira de banho e caiu; apresentou lesões traumáticas na pele com posterior presença de equimose e hematoma, porém, sem fratura. A queda é definida como um deslocamento não intencional do corpo para um nível inferior à posição inicial, com incapacidade de correção em tempo hábil, determinado por circunstâncias multifatoriais e que pode comprometer sobremaneira a estabilidade de quem a sofre (WHO, 2009a). Sua ocorrência é considerada EA de importância epidemiológica e clínica (ABREU; REINERS; AZEVEDO et al., 2014).

Investigação realizada no sul do Brasil em unidades clínicas e cirúrgicas detectou que a maioria dos pacientes que sofreram queda com dano eram idosos (78%), do sexo feminino (55%), em tratamento clínico (68%) e desacompanhados (59,4%). As quedas ocorreram da própria altura (63,4%) e no quarto (67,3%) (LUZIA; PRATES; BOMBARDELLI et al., 2019). Muitos destes achados corroboram com as causas identificadas pela ocorrência de queda evidenciada nesta pesquisa. A prevalência de queda foi observada em estudo realizado na Suécia que

demonstrou taxa de 2,4% (NILSSON; RISBERG; MONTGOMERY et al., 2016) e em estudo desenvolvido no Brasil, que evidenciou taxa de 2,7%. (PARANAGUÁ; BEZERRA; SILVA et al., 2013). Essas taxas estão associadas com as características das instituições hospitalares e dos pacientes (BITTENCOURT; GRAUBE; STUMM et al., 2017).

Diversos outros estudos foram realizados com o objetivo de caracterizar as quedas no ambiente hospitalar (PRATES; LUZIA; ORTOLAN et al., 2014; MENEGUIN; AYRES; BUENO, 2014; ARANDA-GALLARDO; MORALES-ASENCIO; CANCA-SANCHEZ et al., 2014; GUILLAUME; CRAWFORD; QUIGLEY, 2016). Dentre as estratégias para redução dos incidentes de segurança está a prevenção de quedas durante a hospitalização, e representa uma das metas estabelecidas pelo Plano Nacional de Segurança do Paciente (BRASIL, 2013b).

Acredita-se que ações com foco na redução de ocorrência de quedas reflete na qualidade da assistência dos hospitais que prezam por realizar tratamento e cuidados livres de riscos e de danos aos pacientes. Dentre as ações, recomenda-se a capacitação dos profissionais para melhorar a comunicação e os processos de notificação e registros. Ainda, instituição de programas de prevenção de quedas e uso de instrumento padronizado para avaliação de risco, mensuração e identificação dos eventos, disponibilização de instalações adequadas e seguras, bem como, estratégias de melhorias nos processos de trabalho e cultura organizacional para compreender e implementar as mudanças necessárias.

Sobre a ocorrência de EA cirúrgicos aqui evidenciados é importante destacar que estes potencialmente estão associados a diversos fatores, como déficit no quantitativo de profissionais, falta de orientação pré-operatória aos pacientes, preparo inadequado do sítio cirúrgico, falta de liderança na equipe, procedimentos inadequados no intraoperatório e educação continuada insuficiente, semelhantemente ao demosntrado em estudo (SELL; AMANTE; MARTINS et al., 2018). Outros fatores podem ser citados como patologia clínica associada à ocorrência do dano, estado clínico do paciente no pré-operatório, tipo de anestesia, estágio do agravo que levou o paciente à cirurgia e cuidados pós-operatórios (AGUIAR, GOMES; SANTOS et al., 2018).

Neste contexto, a postura frente à segurança do paciente deve ser incentivada nas instituições de saúde e pode interferir na cultura sistêmica das organizações (BRASIL, 2013c). Por este motivo, considera-se que as pesquisas com

foco na cultura de segurança, conforme as percepções dos profissionais, pode direcionar ações para o (re)planejamento do cuidado, com vistas ao atendimento seguro (SOUZA; KAWAMOTO; OLIVEIRA et al., 2015).

A cultura positiva é indispensável em prol do desenvolvimento de ações para prevenção e minimização de eventos com dano relacionados aos cuidados prestados (MINISTERIO DE SANIDAD, SERVICIOS SOCIALES E IGUALDAD, 2015). Assim como, as organizações de saúde necessitam estruturar todo o sistema de forma segura, com desenvolvimento de estratégias e metodologias que permitam a identificação de EA, causas de sua ocorrência, grau de danos ocasionados aos pacientes e à instituição, para que ações corretivas sejam implementadas e medidas preventivas sejam estabelecidas (SILVA; ALVES; SANCHES et al., 2016).

As limitações desta pesquisa se referem à incompletude dos registros médicos e de enfermagem, mesmo nos hospitais que utilizam prontuários eletrônicos, assim como, pela ausência de registros em alguns casos com desfecho de óbito no âmbito domiciliar e/ou outra instituição hospitalar, o que inviabilizou a investigação do critério de rastreamento encontrado na fase I para prosseguir na análise pelos especialistas na fase II.

Houve dificuldade operacional para o acesso aos prontuários e/ou fichas ambulatoriais em vários momentos, em detrimento da dependência prévia de autorização dos diretores dos hospitais, gerências de enfermagem e administrativas locais e aos programas internos de computador, mesmo com aprovação dos respectivos comitês de ética. A pesquisa dependeu da solicitude dos profissionais do serviço de informática para cadastramento de senha, bem como, das secretárias responsáveis em separar e disponibilizar os registros escritos. Infere-se que o tema da pesquisa para avaliação da cultura de segurança e ocorrência de EA cirúrgicos pode ter intimidado a alta gestão hospitalar e demais chefias, o que demonstra a necessidade de mudanças culturais no modo de pensar e enfrentar os problemas causadores de danos aos pacientes cirúrgicos em hospitais brasileiros.

Considerando os EA cirúrgicos identificados nos três hospitais analisados e, principalmente, em relação à alta prevalência de ISC, recomenda-se a vigililância após a alta hospitalar, bem como, o incentivo às notificações e registros em prontuários dos EA, tanto nas unidades de internação cirúrgicas, como nos ambulatórios. As pesquisas na área da segurança cirúrgica do paciente devem orientar os processos decisórios e de intervenção, a fim de causar mudanças na

prática assistencial, melhoria da qualidade da assistência perioperatória, minimização dos riscos e prevenção de EA cirúrgicos.

Considerando os EA cirúrgicos identificados nos três hospitais analisados e, principalmente, em relação à alta prevalência de ISC, recomenda-se a vigilância após a alta hospitalar, bem como, o incentivo às notificações e registros em prontuários dos EA, tanto nas unidades de internação cirúrgicas, como nos ambulatórios. As pesquisas na área da segurança cirúrgica do paciente devem orientar os processos decisórios e de intervenção, a fim de causar mudanças na prática assistencial, melhoria da qualidade da assistência perioperatória, minimização dos riscos e prevenção de EA cirúrgicos.

6.3 TRADUÇÃO, ADAPTAÇÃO TRANSCULTURAL E VALIDAÇÃO DO MÓDULO CIRÚRGICO *GLOBAL TRIGGER TOOL DO INSTITUTE OF HEALTHCARE IMPROVEMENT*

As etapas de tradução, adaptação transcultural e validação o módulo cirúrgico do *Global Trigger Tool* do IHI, e as respectivas orientações sobre cada *trigger* foram realizadas conforme metodologia proposta; a versão final foi encaminhada para os autores do instrumento original (BEATON; BOMBADIER; GUILEMIN et al., 2000; LINO; BRÜGGEMANN; SOUZA et al., 2017) para ser publicada no site da instituição, com objetivo de ser compartilhada e aplicada em pesquisas para identificação e mensuração de EA cirúrgicos no Brasil.

Na trajetória dos profissionais de saúde há diferença entre a realidade do trabalho e o aprendizado acadêmico. Em segurança do paciente, o instrumento rastreador de possíveis EA é, na modalidade acadêmica, uma teoria a ser apreendida e compreendida para sua adequada utilização. Enquanto que na prática profissional, singular, complexa e dinâmica, o instrumento apresentado potencialmente proporciona maior destreza e objetividade para investigação da ocorrência desses eventos. Ter disponível um roteiro traduzido, adaptado e validado para o contexto brasileiro significa aprendizagem rica e produtiva.

No processo de adaptação transcultural de instrumentos dois componentes são essenciais, podendo ser realizados de forma combinada, ou não: a tradução literal de palavras e de sentenças, de um idioma para outro, e a adaptação, que diz respeito à cultura, contexto e estilo de vida da população-alvo. O uso combinado de

tradução e adaptação está indicado quando o pesquisador fizer uso de um instrumento de pesqui
sa em uma população de cultura e língua diferentes daquela para qual o instrumento original foi elaborado (GUILLEMIN; BOMBARDIER; BEATON, 1993).

Estudo de revisão integrativa de artigos que descreviam o processo de adaptação transcultural de instrumentos de pesquisa, conduzido pela enfermagem do Brasil, utilizados na prática clínica hospitalar e publicados entre 2005-2016, evidenciou que em oportunidades diversas, os pesquisadores priorizam a avaliação psicométrica dos instrumentos, em detrimento do pilar que sustenta uma confiável adaptação transcultural, que é o respeito ao rigor metodológico (LINO; BRÜGGEMANN; SOUZA et al., 2017).

A validade transcultural é uma avaliação realizada para se alcançar a validade de construto do instrumento e, diz respeito à medida em que as evidências suportam a inferência de que o instrumento original e uma adaptado culturalmente são equivalentes (POLIT, 2015). Neste sentido, é essencial a utilização de método consistente para tradução, adaptação transcultural e validação de instrumento de pesquisa (AHRQ, 2015).

O Grupo *Consensus-based Standards for the Selection of Health Measurement Instruments* formado por equipe multidisciplinar internacional em prol da melhoria da seleção de instrumentos de medida utilizados na pesquisa e prática clínica, indica que os itens sejam traduzidos e retrotraduzidos por tradutores independentes, que a versão traduzida seja revisada por comitê de especialistas e que o instrumento seja submetido a pré-teste (MOKKINK; TERWEE; PATRICK et al., 2012; MOKKINK; PRINSEN; BOUTER et al., 2016).

Tais estudos são justificados em virtude do aumento do número de estudos multicêntricos, multinacionais e multiculturais, e que exigem o mesmo rigor metodológico adotado na elaboração de novo instrumento (BEATON; BOMBADIER; GUILEMIN et al., 2000). Deste modo, a garantia de uso de qualquer instrumento traduzido, adaptado e validado ancora-se na rigorosidade dos processos de tradução e adaptação cultural adotados, para alcançar a equivalência entre a ferramenta original e a traduzida (EPSTEIN; SANTO; GUILLEMIN, 2015).

Devido à crescente necessidade de cuidados cirúrgicos (SHRIME; BICKLER; ALKIRE et al., 2015), há interesse de métodos confiáveis para mensurar a

segurança do paciente neste ambiente hospitalar, considerado de alto risco para a ocorrência de
EA, em detrimento de cuidados complexos, interdisciplinares, forte dependência da atuação individual, resultando em potenciais complicações intra e pós-operatórias, inclusive de morte (SECANELL; ORREGO; VILA et al., 2014).

O *IHI Surgical Trigger Tool* pode oferecer metodologia com abordagem prática e fácil de usar para detectar problemas na assistência cirúrgica, pois pode ser a base para estimar a frequência de eventos com danos nas organizações e determinar o impacto das intervenções, com foco na redução de EA em pacientes cirúrgicos (GRIFFIN; CLASSEN, 2008). Revisão sistemática sugere que esta metodologia é indicada para mensuração de EA e como estratégia para compreensão e caracterização dos eventos, com a finalidade de promover ações para melhoria da qualidade na assistência à saúde (HIBBERT; MOLLOY; HOOPER et al., 2016).

Quanto à eficácia da metodologia proposta pelo IHI, estudo que utilizou instrumento *Global Trigger Tool,* aprimorado e adaptado para uso nos hospitais de cuidados agudos nos Estados Unidos, a fim de identificar as características dos EA gerais (clínicos e cirúrgicos) durante 5 anos, demonstrou que este criou impulso para os esforços de melhoria direcionados ao atendimento cirúrgico, nos domínios dos processos de atendimento, cultura organizacional e tecnologia (KENNERLY; KUDYAKOV; GRACA et al., 2014).

Na Suécia a tradução e adaptação do método *Global Trigger Tool* resultou em estudo, entre 2009-2012, da análise de 960 prontuários com 271 EA. Destes, 174 ocorreram na assistência cirúrgica e 97 na assistência clínica, o que correspondeu a 33,2 EA/1.000 pacientes dia, sendo que 71% dos eventos eram evitáveis (RUTBERG; RISBERG, SJODAH et al., 2014). Em 2013, outro estudo sueco em 3.301 prontuários identificou 658 EA cirúrgicos, sendo 62,5% classificados como provavelmente evitáveis ou evitáveis. Aproximadamente 50% dos EA contribuíram para a readmissão ou assistência hospitalar prolongada, 4,7% para danos permanentes ou para morte (NILSSON; RISBERG; MONTGOMERY et al., 2016).

Diversos pesquisadores mostram o interesse pelo método elaborado pelo IHI para diagnóstico abrangente de situações que colocam em risco a vida de pacientes e demonstram, no Brasil, e no mundo, sua ampla utilização para investigação da ocorrência de EA na assistência hospitalar (HWANG; CHIN; CHANG, 2014;

KENNERLY; KUDYAKOV; GRACA et al., 2014; HWANG; KIM; PARK et al., 2018), na administração de medicamentos (HARKANEN; KERVINEN; AHONEN et al., 2015; VERONEZE; MALUF; GIORDANI, 2017; HU; QIN; ZHAN et al., 2019), e em diversas áreas do hospital tais como: unidades clínicas (PIERDEVARA; VENTURA; EIRAS et al., 2016), Unidade de Terapia Intensiva (UTI) adulto (ROQUE; TONINI; MELO, 2016), UTI neonatal e pediátrica (HOOPER; TIBBALLS, 2014), unidades pediátricas (STOCKWELL; BISARYA; CLASSEN et al., 2015; DAVENPORT; DOMÍNGUEZ; FERREIRA et al., 2017) e ambientes cirúrgicos (ZAPATA; SAMANIEGO; CUÉLLAR et al., 2015).

Todos as pesquisas são relevantes para a segurança do paciente e em especial aquelas menos frequentemente dirigidas ao paciente cirúrgico, o que justifica a relevância de estudos desta natureza. Nesta pesquisa, as etapas foram seguidas, analisadas e documentadas para alcançar os objetivos propostos. Durante as etapas para tradução (Etapa 1), síntese das traduções (Etapa 2) e retrotradução (Etapa 3), foram realizadas alterações na gramática e adaptações de termos para utilização no Brasil, a fim de assegurar a compreensão e a escolha correta na versão traduzida, além de garantir sua compreensão e clareza (BEATON; BOMBADIER; GUILEMIN et al., 2000). Não foi excluído nenhum item do instrumento original.

Na Etapa 4, para validação do instrumento, foram avaliadas a versão V6, que foi a síntese das traduções e retrotraduções realizadas anteriormente, bem como, as equivalências semântica, idiomática, conceitual, cultural e de conteúdo, para verificação da concordância entre os especialistas, de acordo com a técnica Delphi *on-line*.

Conforme a referida técnica, os questionários são utilizados para coleta de dados e as respostas, para cada questão, são consideradas para a reformulação do instrumento na rodada subsequente, até atingir o consenso esperado (PERROCA, 2011). Neste contexto, o uso da técnica Delphi como referencial metodológico é importante pois, permite que a coleta e análise de dados se torne um processo dinâmico, por intermédio de *feedbacks* controlados de opiniões até que seja alcançado o referido consenso sobre o assunto em avaliação (MASSAROLI; MARTINI; LINO et al., 2017).

A validade de conteúdo refere-se à análise do instrumento em relação ao universo dos conceitos e domínios que se deseja medir, ou seja, se o instrumento é

adequado para os fins almejados (PASQUALI, 2009), ou seja, refere-se à propriedade de um instrumento medir exatamente o que se propõe (POLIT, 2015). Destaca-se, portanto, a inexistência de métodos totalmente objetivos para garantir que um instrumento atinja de modo adequado o conteúdo em medição, pois não há teste estatístico capaz de determinar se uma medida abrange ou não, toda a área do conteúdo ou se representa adequadamente a construção do instrumento (PASQUALI, 2009).

Desta forma, a validade de conteúdo, ancora-se no julgamento de especialistas da área, que analisam à adequação dos itens em representar o universo hipotético do conteúdo nas proporções corretas. Ao final da análise dos especialistas, é possível calcular o índice de validade de conteúdo (IVC) que indica a extensão da concordância dos juízes (PASQUALI, 2009).

Para a verificação da validade de instrumentos autores sugerem a porcentagem de concordância mínima de 80% entre os especialistas (COLUCI; ALEXANDRE; MILANI, 2015; LINO; BRÜGGEMANN; SOUZA et al., 2017) e IVC nos valores entre 0,5 e 0,8, pois este índice mede a proporção de juízes que estão em concordância sobre determinados aspectos e itens do instrumento, por permitirem a análise de cada item do instrumento individualmente e em seguida de forma integral. (REVORÊDO; DANTAS; MAIA et al., 2016).

A avaliação de conteúdo deve ser realizada por comitê de especialistas composto por cinco a dez *experts* na área do instrumento. Quanto maior o resultado do IVC, maior é o nível de concordância entre os especialistas (COLUCI; ALEXANDRE; MILANI, 2015). Ressalta-se que nesta pesquisa, seguiu-se criteriosa seleção de profissionais especialistas para assegurar a qualidade do processo avaliativo e, conforme os parâmetros pré-definidos de IVC≥0,80 e escore de concordância ≥80%, os resultados demonstram que o consenso foi atingido na primeira rodada de avaliação, com média geral de 92,4% de concordância e IVC geral do instrumento de 1,38.

Para o processo de adaptação cultural, recomenda-se a avaliação das propriedades psicométricas do instrumento, a fim de verificar se as características do instrumento original foram mantidas. Esta avaliação consiste na verificação dos parâmetros de validade e confiabilidade do instrumento, considerando-se os valores atribuídos a cada item do constructo avaliado (PASQUALI, 2009). Deste modo, a análise de validade do módulo cirúrgico GTT do IHI e as respectivas orientações foi

alcançada conforme índices satisfatórios de IVC e validade transcultural (equivalências semântica, idiomática, conceitual, cultural e de conteúdo).

Para a análise da confiabilidade, estudos indicam a avaliação da consistência interna do instrumento pela mensuração do coeficiente alfa de *Cronbach*; a confiabilidade é a capacidade em reproduzir um resultado de forma consistente, no tempo e no espaço (SOUZA; ALEXANDRE; GUIRARDELLO, 2017). Nesta pesquisa, este coeficiente apresentou valor geral de 0,83 para os 11 *triggers* e as respectivas orientações e valor geral de 0,77 para as equivalências semântica, idiomática, conceitual, cultural e de conteúdo. O coeficiente alfa de *Cronbach* foi considerado satisfatório, demonstrando a alta confiabilidade do instrumento.

Enfim, a confiabilidade e a validade são consideradas as principais propriedades de medida de instrumentos e a avaliação dessas propriedades é útil para demonstrar que os instrumentos são válidos e confiáveis, de modo a garantir a qualidade dos resultados dos estudos (SOUZA; ALEXANDRE; GUIRARDELLO, 2017).

A realização da Etapa 5 (Pré-teste) teve como objetivo verificar se todos os itens estavam compreensíveis para a finalidade a que se destina e deve ser realizada em amostra de 30-40 indivíduos (BEATON; BOMBADIER; GUILEMIN et al., 2000; LINO; BRÜGGEMANN; SOUZA et al., 2017). Nesta pesquisa, para realização desta etapa utilizou-se a versão V7, durante a análise retrospectiva de amostra/aleatória/simples de 244 prontuários de pacientes submetidos à cirurgia no Hospital C, para identificar ocorrência de EA; o tempo máximo para análise de cada prontuário pelos pesquisadores foi de 20 minutos, conforme metodologia do IHI (GRIFFIN; RESAR, 2009).

Os atributos simplicidade, aplicabilidade e possibilidade de mensuração são considerados essenciais para que o instrumento seja utilizado de forma eficaz em instituições de saúde (WHO, 2008b). Verificou-se que a versão traduzida, adaptada e validada para cultura brasileira do módulo cirúrgico GTT do IHI é confiável, de fácil compreensão e aplicação prática, pois permite o aprimoramento da coleta de dados nas análises retrospectivas de prontuários para identificação e mensuração de pEA cirúrgicos.

Na etapa 6 (Avaliação pelos autores do instrumento) a versão V8 elaborada pela pesquisadora e a professora orientadora foi encaminhada para os autores do instrumento original para ciência, avaliação e aprovação. O cumprimento e a

descrição
de cada etapa desenvolvida nos estudos de adaptação transcultural fazem toda diferença no resultado e, demonstram ao leitor, a seriedade com que deve ser conduzido. A produção confiável de instrumentos que passaram por este processo permite sua utilização prática em outros estudos (COLUCI; ALEXANDRE; MILANI, 2015; LINO; BRÜGGEMANN; SOUZA et al., 2017). Nesta pesquisa, as etapas foram realizadas de modo a atingir índices de validade e confiabilidade adequados.

Como limitações da pesquisa, apresenta-se o fato de que para se atingir o nível de consenso com seis especialistas e alcançar IVC válido, foi necessário que cinco destes avaliassem cada item com as opções de resposta concordo (+1) ou concordo totalmente (+2). Além disto, verifica-se que o modulo cirúrgico do instrumento foi traduzido, adaptado e validado em poucos países, o que minimiza os processos para discussão e comparação dos resultados.

Infere-se que o instrumento validado no Brasil permitirá generalizar seu uso nas investigações que buscam a identificação de EA cirúrgicos no país, por isso, recomenda-se a utilização da metodologia GTT em outros serviços hospitalares, a fim de impulsionar os esforços de melhoria para minimização da ocorrência desses eventos e fortalecimento da cultura organizacional e de segurança nos hospitais.

7 CONCLUSÃO

Avaliou-se a cultura de segurança dos três hospitais de ensino com o uso do instrumento HSOPSC nas unidades de centro cirúrgico e de internação cirúrgicas, e evidenciou-se fragilidades nos três hospitais e diferenças nas percepções dos profissionais. Tal avaliação fornece indicadores sobre o estado real em que se encontra a cultura nas instituições e contribui para o planejamento e acompanhamento temporal de estratégias de melhorias.

A dimensão “Trabalho em equipe dentro das unidades” foi a única fortalecida e identificada, apenas, no Hospital B, e remete ao fato destes profissionais se sentirem respeitados em seu local de atuação para a realização do trabalho em equipe, em busca da assistência cirúrgica segura.

As dimensões “Expectativas sobre seu supervisor/chefe - ações promotoras de segurança” e “Aprendizado organizacional - melhoria contínua” apresentaram respostas neutras, com significância estatística nos três hospitais. Deste modo, os escores são classificados como adequados, porém, não estão fortalecidos, e mostram que essas dimensões apresentam potencial para se tornarem áreas fortes da cultura de segurança.

Os resultados demonstram fragilidades em seis das 12 dimensões que compreendem o instrumento de avaliação: “Apoio da gestão para a segurança do paciente”, “Percepção geral da segurança do paciente”, “Retorno da informação e comunicação sobre o erro”, “Adequação de profissionais”, “Passagem de plantão/turnos e transferências” e “Respostas não punitivas ao erro”.

Duas áreas avaliadas da cultura de segurança merecem destaque e representam sinal de alerta nos três hospitais participantes. A primeira se refere à “Respostas não punitivas ao erro” com o maior percentual de respostas negativas. Esta dimensão faz parte do contexto que analisa a unidade de trabalho ou departamento hospitalar. A segunda área em destaque compreende a avaliação do número de eventos notificados nos últimos 12 meses, de acordo com as três categorias profissionais (médicos assistentes, médicos residentes e equipe de enfermagem), cujos resultados mostram alta frequência de respostas para ausência de notificações. Apenas no Hospital A, na categoria médicos assistentes, verificou-se prevalência para uma a duas notificações anuais.

A dimensão "Respostas não punitivas ao erro" e os resultados do número de eventos notificados nos últimos 12 meses estão correlacionados pois, a resposta punitiva ao erro é a maior barreira à notificação de EA cirúrgicos.

A análise dos dados mostra que dimensões neutras podem ser aperfeiçoadas, enquanto as fragilizadas devem ser analisadas e desenvolvidas para que ocorra a promoção de cultura positiva, principalmente, em busca do fortalecimento de cultura justa e não punitiva frente aos erros cirúrgicos.

Na primeira fase desta pesquisa utilizou-se o módulo cirúrgico GTT do IHI como um dos instrumentos para rastreamento de potenciais EA cirúrgicos. Os 11 *triggers* (gatilhos ou pistas), e respectivas orientações, foram traduzidos, adaptados transculturalmente e validados para o português do Brasil, com avaliação das equivalências semântica, idiomática, conceitual, experimental e de conteúdo. Na análise dos especialistas, os resultados comprovam validade de conteúdo suficientes, bem como alta confiabilidade. Durante o pré-teste do instrumento verificou-se que esse é de fácil compreensão, tem aplicabilidade prática e pode auxiliar nas pesquisas em outros cenários cirúrgicos, pois permite o aprimoramento da coleta de dados nas análises retrospectivas de prontuários durante as investigações para identificação e mensuração de EA cirúrgicos.

Quanto à ocorrência de EA cirúrgicos, os resultados permitiram a avaliação das causas de sua ocorrência, prevalência, grau de dano e evitabilidade, de acordo com a avaliação dos diferentes contextos organizacionais. Quase a totalidade destes eventos foram identificados durante o internamento no Hospital B e Hospital C, ao contrário dos resultados encontrados no Hospital A, que foram detectados no retorno ambulatorial e representaram, aproximadamente, mais da metade dos identificados.

A maioria dos pacientes apresentou um EA cirúrgico; entretanto, taxas superiores em relação à ocorrência de três a nove eventos por paciente prevaleceram no Hospital B e C. Em relação à gravidade dos eventos observou-se prevalência de danos leves e/ou moderados nos três hospitais, no entanto, evidenciou-se taxas relevantes de danos graves e óbitos no Hospital B e Hospital C. Em relação à evitabilidade, classificaram-se como evitáveis pois, as maiores porcentagens referem-se aos "potencialmente preveníveis", seguidos dos "fortemente preveníveis".

A média de dias de internamento apresentou diferença, com significância estatística, entre os hospitais. Evidenciaram-se taxas de prevalência elevadas e

equivalentes em relação ao tempo de internação ≥6 dias nos Hospitais B e C. Detectou-se o prolongamento da internação que possivelmente decorreu, não somente em virtude da gravidade do diagnóstico principal, mas também, pela ocorrência de EA cirúrgicos. Tais circunstâncias foram observadas em vários momentos da investigação e a análise dos dados, em relação ao maior tempo de internação, equivaleu a 98 dias no Hospital A, 72 dias no Hospital B e 117 dias no Hospital C.

O tempo de internação pré-operatório ≥24 horas mostrou que a taxa verificada no Hospital B foi, consideravelmente, maior que a observada no Hospital A e Hospital C, praticamente equivalentes e com significância estatística. É fato comprovado que o prolongamento do período pré-operatório e da internação hospitalar total aumenta os riscos de complicações pós-operatórias e, consequentemente, a ocorrência de EA cirúrgicos.

Outro resultado a destacar refere-se às readmissões; o Hospital A apresentou menor prevalência, seguido do Hospital B, porém, o maior número de casos de pacientes que sofreram readmissões decorrentes do EA ocorreu no Hospital C.

Desta forma, as variáveis analisadas como período total de internação, tempo de internação pré-operatório ≥24 horas e readmissão representam alguns dos indicadores utilizados na prática assistencial em saúde, os quais permitem a análise dos riscos a que os pacientes podem estar expostos, além da avaliação da qualidade dos tratamentos e cuidados prestados nos hospitais participantes desta pesquisa.

Os resultados sobre os principais eventos adversos demonstram variedade de danos e maior prevalência de Infecção de Sítio Cirúrgico. Quanto ao segundo e terceiro lugares no *rankink* de prevalência concentraram-se: Septicemia/Choque Séptico nos Hospitais B e C; Hematoma/Seroma no Hospital A; Deiscência de Sutura nos Hospitais A e B; Fístulas e Infecção do Trato Urinário no Hospital C.

Destaca-se a ausência de eventos como retenção de instrumentos ou demais materiais utilizados em cirurgia, troca de paciente, local ou lado a ser operado, suscitando possível evolução nas ações em prol da segurança do paciente cirúrgico, decorrente de recomendações e políticas internacionais e nacionais.

Os achados de pesquisa mostram ambiente de cuidados inseguros na assistência perioperatória, a qual precisa de melhorias e adequação às

recomendações internacionais e diretrizes nacionais. Considera-se organização hospitalar segura quando há esforço e cooperação coletiva, de todos os profissionais de saúde, em todos os níveis hierárquicos, para fortalecer a cultura de segurança do paciente.

Torna-se imprescindível o reconhecimento da problemática inerente à frágil cultura de segurança do paciente e a ocorrência de danos evitáveis para o desenvolvimento de ações corretivas em prol da segurança cirúrgica. Os resultados mostram fragilidades na cultura de segurança na metade das dimensões, escores de respostas positivas inferior ao estabelecido para a cultura positiva nas demais e alta prevalência de EA cirúrgicos evitáveis nos três hospitais analisados, além de índices preocupantes de danos graves e óbitos em dois hospitais.

Portanto, infere-se que as evidências científicas encontradas e os demais estudos publicados nesta área, e descritos nesta pesquisa, direcionam a existência de associação entre escores frágeis de cultura de segurança e alta prevalência de EA cirúrgicos.

REFERÊNCIAS

ALSWAT, K.; ABDALLA, R.A.M.; TITI, M.A. et al. Improving patient safety culture in Saudi Arabia (2012–2015): trending, improvement and benchmarking. **BMC Health Serv Res,** v. 17, n. 516, 2017. Disponível em:<https://bmchealthservres.biomedcentral.com/articles/10.1186/s12913-017-2461-3 doi:10.1186/s12913-017-2461-3>. Acesso em: 12 ago. 2019.

ABREU, H.C.A.; REINERS, A.A.O.; AZEVEDO, R.C.S. et al. Urinary incontinence in the prediction of falls in hospitalized elderly. **Revista da Escola de Enfermagem da USP**, v. 48, n. 5, p. 851-856, 2014. Disponível em:<http://www.scielo.br/scielo.php?script=sci_arttext&pid=S0080-62342014000500851&lng=en&nrm=iso>. Acesso em: 20 set. 2019.

ADAMS, P.D.; RITZ, J.R.N.; KATHER, R.B.S. et al. The differential effects of surgical harm in elderly populations. Does the adage: “they tolerate the operation, but not the complications” hold true? **The Am J Surg**, v. 208, n. 4, p. 656–662, 2014. Disponível em:<https://www.americanjournalofsurgery.com/article/S0002-9610(14)00206-2/fulltext>. Acesso em: 02 de set. 2019.

AGENCY FOR HEALTHCARE RESEARCH AND QUALITY (AHRQ). **Care safer II: An Updated Critical Analysis of the Evidence for Patient Safety Practices**.Rockville, MD-Agency for Healthcare Research and Quality,2013. Disponível em:<www.ahrq.gov/research/findings/evidence-based-reports/ptsafetyuptp.html>. Acesso em: 23 de set. 2018.

AGENCY FOR HEALTHCARE RESEARCH AND QUALITY (AHRQ). **Hospital Survey on Patient Safety Culture.** Rockville, MD-AHRQ, 2017. Disponível em:<http://www.ahrq.gov/professionals/qualitypatientsafety/patientsafetyculture/hospital/index.html>. Acesso em: 05 de abr. 2018.

AGENCY FOR HEALTHCARE RESEARCH AND QUALITY (AHRQ). **Medical Office Survey on Patient Safety Culture**. Rockville, MD-AHRQ, 2015. Disponível em:<http://www.ahrq.gov/professionals/quality-patient-safety/patientsafetyculture/medical-office/2012/index.html>. Acesso em: 07 de abr. 2018.

AGENCY FOR HEALTHCARE RESEARCH AND QUALITY (AHRQ). **Quality Indicator User Guide: Patient Safety Indicators (PSI) Composite Measures**. Columbus-AHRQ, 2011. Disponível em:<http://www.qualityindicators.ahrq.gov/Downloads/Modules/PSI/V43/Composite User_Technical_Specification_PSI_4.3.pdf>. Acesso em: 23 de set. 2018.

AGUIAR, P.V.; GOMES, E.T.; dos SANTOS, I.N. et al. Pacientes submetidos a cirurgias bariátricas: fatores associados a complicações pós-operatórias de sítio cirúrgico. **Revista SOBECC**, São Paulo, v. 23, n. 1, p. 28-35, 2018. Disponível em: <https://revista.sobecc.org.br/sobecc/article/view/390>. Acesso em: 9 de out. 2019.

AIKEN, L.H.; SLOANE, D.M.; BRUYNEEL, L. et al. Nurse staffing and education and hospital mortality in nine European countries: a retrospective observational study. **The Lancet**, v. 383, n. 9931, p. 1824-1830, 2014. Disponível em:<https://www.thelancet.com/journals/lancet/article/PIIS0140-6736(13)62631-8/fulltext>. Acesso em: 05 de ago. 2019.

ALEXANDRE, N.M.C.; COLUCI, M.Z.O. Validade de conteúdo nos processos de construção e adaptação de instrumentos de medida. **Ciên Saúde Coletiva**. v. 16, n. 7, p. 3061-8, 2011. Disponível em:<http://dx.doi.org/10.1590/S1413-81232011000800006> Acesso em: 05 out. 2018.

ALI, H.; IBRAHEM, S. Z.; MUDAF, A.B. et al. Baseline assessment of patient safety culture in public hospitals in Kuwait. **BMC Health Serv. Research**, v. 18, n. 1, p. 158, 2018. Disponível em:<https://www.ncbi.nlm.nih.gov/pmc/articles/PMC5840785/>. Acesso em: 15 set. 2019.

ALPENDRE, F.T; CRUZ, E.D.A; DYNIEWICZ, A.M. et al. Safe surgery: validation of pre and postoperative checklists. **Rev. Latino-Am. Enferm.**, Ribeirão Preto, v. 25, e2907, 2017. Disponível em:<http://dx.doi.org/10.1590/1518-8345.1854.2907>. Acesso em: 15 ago. 2019.

ALSWAT, K; ABDALLA, R.A.M; TITI, M.A. et al. Improving patient safety culture in Saudi Arabia (2012–2015): trending, improvement and benchmarking. **BMC Health Services Research**, v. 17, n. 516, não p., 2017. Disponível em:<https://doi.org/10.1186/s12913-017-2461>. Acesso em: 5 de set. 2019.

AMARAL, C.; PEREIRA, L.G.; MORETO, A. et al. The postoperative venous thromboembolism (TREVO) study – Risk and case mortality by surgical specialty. **Rev Port Cardio**l, v. 36, n. 9, p. 609-616, 2017. Disponível em:<https://www.ncbi.nlm.nih.gov/pubmed/28867599>. Acesso em: 12 de out. 2019.

AMERICAN COLLEGE OF HEALTHCARE EXECUTIVES (ACHE). Institute for Healthcare Improvement (IHI). National Patiente Safety Foudation (NPSF). LUCIAN LEAPE INSTITUTE. **Leading a culture of cafety: a blueprint for success.** Boston, MA-American College of Healthcare Executives e Institute for Healthcare Improvement, 2017. Disponível em:<http://www.ihi.org/resources/Pages/Publications/Leading-a-Culture-of-Safety-A-Blueprint-for-Success.aspx>. Acesso em: 25 de nov. 2018.

ANDRADE, L.E.L.; LOPES, J.M.; SOUZA FILHO, M.C.M. et al. Patient safety culture in three Brazilian hospitals with different types of management. **Ciênc. Saúde Coletiva**, v. 23, n. 1, p. 161-172, 2018. Disponível em: <https://dx.doi.org/10.1590/1413-81232018231.24392015>. Acesso em: 03 de jul. 2019.

ANDRADE, L.E.L.; MELO, L.O.M.; SILVA, I.G. et al. Adaptação e validação do Hospital Survey on Patient Safety Culture em versão brasileira eletrônica. **Epidemiol. Serv. Saúde**, Brasília, v. 26, n. 3, p. 455-468, 2017. Disponível em:<http://www.scielo.br/scielo.php?script=sci_arttext&pid=S2237-96222017000300455&lng=en&nrm=iso>. Acesso em: 05 ago. 2019. http://dx.doi.org/10.5123/s1679-49742017000300004.

ANDERSON, O.; DAVIS, R.; HANNA, G.B. et al. Surgical adverse events: a systematic review. **Am J Surg**, v. 206, n. 2, p. 253-62, 2013. Disponível em:<https://www.americanjournalofsurgery.com/article/S0002-9610(13)00175-X/fulltext>. Acesso em: 12 de set. 2019.

ARANDA-GALLARDO, M.; MORALES-ASENCIO, J.M.; CANCA-SANCHEZ, J.C. et al. Circumstances and causes of falls by patients at a Spanish acute care hospital. **J Eval Clin Pract.**, v. 20, n. 5, p. 631-637, 2014. Disponível em:<https://doi.org/10.1111/jep.12187>. Acesso em: 5 de out. 2019.

ARAÚJO, I.S.; CARVALHO, R. Eventos adversos graves em pacientes cirúrgicos: ocorrência e desfecho. **Rev. SOBECC**, v. 23, n. 2, p. 77-83, 2018. Disponível em:<https://revista.sobecc.org.br/sobecc/article/view/77>. Acesso em: 12 out. 2019.

ARAVIND, M.; CHUNG, K.C. Evidence-based medicine and hospital reform: tracing origins back to Florence Nightingale. **Plast Reconstr Surg**, v. 125, n. 1, p. 403-9, 2010. Disponível em:<https://www.ncbi.nlm.nih.gov/pmc/articles/PMC4386633/?report=reader>. Acesso em: 19 de set. 2019.

ARRIETA, A; SUÁREZ, G; HAKIM, G. Assessment of patient safety culture in private and public hospitals in Peru. **Int J Qual Health Care**, Oxford, v. 30, n. 3, não p., 2018. Disponível em:<https://doi.org/10.1093/intqhc/mzx165>. Acesso em: 05 de jul. 2019.

BABLEKOS, G.D.; MICHAELIDES, S.A.; ANALITE, A. et al. Effects of laparoscopic cholecystectomy on lung function: a systematic review. **World J Gastroenterol**, v. 20, n. 46, p. 17603–17617, 2014. Disponível

em:<https://www.ncbi.nlm.nih.gov/pmc/articles/PMC4265623>. Acesso em: 20 de out. 2019.

BAKER, G.R.; NORTON, P.G.; FLINTOFT, V. et al. The Canadian Adverse Events Study: the incidence of adverse events among hospital patients in Canada. **CMAJ**, v. 25, n. 170(11), p. 1678-1686, 2004. Disponível em:<https://www.ncbi.nlm.nih.gov/pmc/articles/PMC408508/>. Acesso em: 03 de jun. 2017.

BANCOS, I.; HAHNER, S.; TOMLINSON, J. et al. Diagnosis and management of adrenal insufficiency. **The Lancet**, v. 3, n. 3, p. 216-226, 2015. Disponível em:<https://www.thelancet.com/journals/landia/article/PIIS22138587(14)70142-1/fulltext>. Acesso em: 19 de out. 2019.

BARBASH, I.J.; KAHN, J.M.; THOMPSON, B.T. Medicare's Sepsis reporting program: two steps forward, one step Back. **Am J Respir Crit Care Med**, v. 194, n. 2, p. 139, 2016. Disponível em:<https://doi.org/10.1164/rccm.201604-0723ED>**.** Acesso em: 10 de out. 2019.

BARBOSA, M.H.; OLIVA, A.M.B.; SOUSA, NETO A.L. Ocorrência de lesões perioperatórias por posicionamento cirúrgico. **Rev. Cubana Enfermería**. v. 27, n. 1, p. 31-41, 2011. Disponível em:<http://scielo.sld.cu/pdf/enf/v27n1/enf05111.pdf>. Acesso em: 16 de out. 2019.

BATISTA J.; CRUZ E.D.A.; ALPENDRE F.T. et al. Cultura de segurança e comunicação sobre erros cirúrgicos na perspectiva da equipe de saúde. **Rev. Gaúcha Enferm.**, Porto Alegre, v. 40, n. 20180192, não p., 2019. Disponível em:<https://doi.org/10.1590/1983-1447.2019.20180192>. Acesso em: 10 de set. 2019.

BATISTA, J. Avaliação da cultura de segurança e ocorrência de EAs cirúrgicos em diferentes naturezas administrativas da gestão pública. 152 p, 2018. Disponível em:<https://acervodigital.ufpr.br/handle/1884/55069>. Acesso em: 10 de set. 2019.

BEATON, D.E.; BOMBARDIER, C., GUILLEMIN, F. et al. Guidelines for the Process of Cross-Cultural Adaptation of Self-Report Measures. **Spine,** v. 25, n. 24, p. 3186-3191, 2000. Disponível em:<https://www.ncbi.nlm.nih.gov/pubmed/11124735>. Acesso em: 30 de maio 2018.

BEKS, R.B.; CLAESSEN, F.M.A.P.; OH, L.S. et al. Factors associated with adverse events after distal biceps tendon repair or reconstruction. **J Shoulder Elbow Surg**, v.

25, n. 8, p. 1229–1234, 2016. Disponível em:<http://dx.doi.org/10.1016/j.jse.2016.02.032>. Acesso em: 16 de ago. 2019.

BERRÍOS-TORRES, S.I.; UMSCHEID, C.A.; BRATZLER, D.W. et al. Centers for Disease Control and Prevention Guideline for the Prevention of Surgical Site Infection, 2017. **JAMA Surg**, v. 152, n. 8, p. 784-791, 2017. Disponível em:<https://doi.org/10.1001/jamasurg.2017.0904>. Acesso em: 05 de set. 2019.

BEZERRA, M.B.G.; GALVÃO, M.C.B.; VIEIRA, J.C.M. et al. Factors associated with skin lesions resulting during the intraoperative period. **Rev. SOBECC,** São Paulo, v. 24, n. 2, p. 76-84, 2019. Disponível em:<https://revista.sobecc.org.br/sobecc/article/view/465/pdf>. Acesso em: 11 de set. 2019.

BEZERRA, W.R.; BEZERRA, A.L.Q.; PARANAGUÁ, T.T.B. et al. Ocorrência de incidentes em um centro cirúrgico: estudo documental. **Rev. Eletr. Enf.**, v. 17, n. 4, não p, 2015. Disponível em:<http://dx.doi.org/10.5216/ree.v17i4.33339>. Acesso em: 15 de nov. 2017.

BITTENCOURT, V.; GRAUBE, S.; STUMM, E. et al. Factors associated with the risk of falls in hospitalized adult patients. **Revista da Escola de Enfermagem da USP**, v. 51, p. e03237, 2017. Disponível em:<http://www.periodicos.usp.br/reeusp/article/view/134929>. Acesso em: 13 de out. 2019.

BOHOMOL, E.; MELO, E.F. Cultura de segurança do paciente em centro cirúrgico: percepção da equipe de enfermagem. **Rev. SOBECC**, São Paulo, v. 24, n. 3, p. 132-138, 2019. Disponível em:<https://revista.sobecc.org.br/sobecc/article/view/491>. Acesso em: 30 de jun. 2018.

BONDEVIK, G.T.; HOFOSS, D.; HANSEN, E.H. et al. Patient safety culture in Norwegian primary care: a study in out-of-hours casualty clinics and GP practices. **Scand J Prim Health Care**, v. 32, n. 3, p. 132-8, 2014. Disponível em:<https://doi.org/10.3109/02813432.2014.962791>. Acesso em: 5 de set. 2018.

BRANDAO, P.F.; MACEDO, P.H.A.P.; RAMOS, F.S. Choque hemorrágico e trauma: breve revisão e recomendações para manejo do sangramento e da coagulopatia. **RMMG**, v. 27, n. 4, 2019. Disponível em:<http://www.dx.doi.org/10.5935/2238-3182.20170041>. Acesso em: 15 de out. 2019.

BRASIL. Agência Nacional de Vigilância Sanitária (ANVISA). **Assistência Segura: Uma Reflexão Teórica Aplicada à Prática.1 Ed**. Brasília, DF- Editora-2013a.

Disponível em:<https://www.segurancadopaciente.com.br/central_conteudo/assistencia-segura-uma-reflexao-teorica-aplicada-a-pratica/>. Acesso em: 10 de mar. 2018.

BRASIL. Agência Nacional de Vigilância Sanitária (ANVISA). **Critérios Diagnósticos de Infecções Relacionadas à Assistência à Saúde. 2. Ed**. Brasília, DF-ANVISA-2017c. Disponível em:<http://www.saude.df.gov.br/institucional/982-infeccoes-relacionadas-a- assistencia-a-saude.html>. Acesso em: 12 de set. 2019.

BRASIL. Agência Nacional de Vigilância Sanitária (ANVISA). **Implantação do Núcleo de Segurança do Paciente em Serviços de Saúde-Série Segurança do Paciente e Qualidade em Serviços de Saúde**. Brasília, DF- ANVISA, 2016. Disponível em:<https://www20.anvisa.gov.br/segurancadopaciente/index.php/publicacoes/item/caderno-6-implantacao-do-nucleo-de-seguranca-do-paciente>. Acesso: 24 de nov. 2018.

BRASIL. Agência Nacional de Vigilância Sanitária (ANVISA). **NOTIVISA**. 2019 Disponível em:<http://www20.anvisa.gov.br/segurancadopaciente/index.php/notificacoes>. Acesso em 30 de set. 2019.

BRASIL. Agência Nacional de Vigilância Sanitária (ANVISA). **Plano Nacional para a Prevenção e o Controle da Resistência Microbiana nos Serviços de Saúde**. Brasília, DF-ANVISA, 2017b. Acesso em: 10 de out. 2019.

BRASIL. Ministério da Saúde. Agência Nacional de Vigilância Sanitária (ANVISA). **Documento de referência para o Programa Nacional de Segurança do Paciente**. ANVISA-Brasília, 2014. Disponível em:<http://bvsms.saude.gov.br/bvs/publicacoes/documento_referencia_programa_nacional_seguranca.pdf>. Acesso em: 24 de nov. 2018.

BRASIL. Ministério da Saúde. Agência Nacional de Saúde Suplementar (ANS). **Média de permanência geral**. Rio de Janeiro. ANS, 2013d. Disponível em:<http://www.ans.gov.br/images/stories/prestadores/E-EFI-05.pdf>. Acesso em: 03 de ago. 2019.

BRASIL. Ministério da Saúde. Agência Nacional de Vigilância Sanitária (ANVISA). **Gestão de Riscos e Investigação de Eventos Adversos Relacionados à Assistência à Saúde**.Brasília, DF-ANVISA, 2017a. Acesso em: 15 de fev. 2019.

BRASIL. Ministério da Saúde. Agência Nacional de Vigilância Sanitária (ANVISA).

Relatórios dos estados. Brasília, DF-ANVISA, 2018a. Disponível em:<www20.anvisa.gov.br/segurancadopaciente/index.php/publicacoes/category/relatorios-dos-estados>. Acesso: 21 de nov. 2018.

BRASIL. **Cadastro Nacional dos Estabelecimentos de Saúde (CNES)**. Brasil, 2018c. Disponível em:<http://cnes.datasus.gov.br/>. Acesso: 04 de set. 2018.

BRASIL. Ministério da Saúde. Conselho Nacional de Saúde. Resolução nº 466, de 12 de dezembro de 2012. **Diretrizes e normas regulamentadoras de pesquisa envolvendo seres humanos**, Brasília, 2012, p.12, dez.2012.

BRASIL. Ministério da Saúde. Departamento de Informática do Sistema Único de Saúde. **DATASUS**-Departamento de Informática do SUS. TABNET, 2018b. Disponível em:<http://datasus.saude.gov.br/datasus>. Acesso em: 25 de nov. 2018.

BRASIL. Ministério da Saúde. Portaria interministerial nº 285, de 24 de março de 2015. Redefine o Programa de Certificação de Hospitais de Ensino (HE). **Ministério da Saúde**, Brasília, DF. Disponível em:<http://bvsms.saude.gov.br/bvs/saudelegis/gm/2015/prt0285_24_03_2015.html>. Acesso em: 07 de fev. 2019.

BRASIL. Ministério da Saúde. Portaria interministerial nº 2.400, de 02 de outubro de 2007. Estabelece os requisitos para certificação de unidades hospitalares como Hospitais de Ensino (HE). **Ministério da Saúde**, Brasília, DF, 03 outubro 2007. Disponível em:<https://abmes.org.br/arquivos/legislacoes/Portaria-Interministerial-2400-2007-10-02.pdf>. Acesso em: 07 de fev. 2019.

BRASIL. Portaria nº 529 de 1º de abril de 2013. Institui o Programa Nacional de Segurança do Paciente (PNSP). **Ministério da Saúde**, Brasília, 2013b. Disponível em:<http://www.in.gov.br/autenticidade.html>. Acesso em: 06 de maio 2017.

BRASIL. Resolução – RDC n° 36 de 25 de julho de 2013. Institui ações para a segurança do paciente em serviços de saúde e dá outras providências. **Diário Oficial da União**, Brasília, n.36, 23 de julho de 2013c. Disponível em:<http://bvsms.saude.gov.br/bvs/saudelegis/anvisa/2013/rdc0036_25_07_2013.html>. Acesso em 01 de jun. 2017.

BRITO, L.F.M. **Segurança aplicada às instalações hospitalares**. 4. ed. São Paulo: Editora Senac São Paulo, 2006.

BRUECKMANN, B.; VILLA-URIBE, J.L.; BATEMAN, B.T. et al. Development and validation of a score for prediction of postoperative respiratory complications. **Anesthesiology**, v. 118, n. 6, p. 1276-85, 2013. Disponível em:<https://anesthesiology.pubs.asahq.org/article.aspx?articleid=1918355>. Acesso em: 15 de out. 2019.

BURLISON, J.D.; QUILLIVAN, R.R.; KATH, L.M et al. A multilevel analysis of U.S. Hospital Patient Safety Culture relationships with perceptions of voluntary event reporting. **J Patient Saf**., não p, 2016. Disponível em:<https://www.ncbi.nlm.nih.gov/pmc/articles/PMC5415419/>. Acesso em: 12 de ago. 2019.

BUSSAB, W.O.; MORETTIN, P. A. **Estatística básica**. 6. ed. São Paulo: Saraiva, 2017. 568p.

CABRAL, G.D.B.; SILVA, R.F.; BORGES, Z.D.O. Postoperative pulmonar complications: predictors. **RMMG**, v. 24, n. 8, p. 73-80, 2014. Disponível em:<http://www.dx.doi.org/10.5935/2238-3182.20140130>. Acesso em: 10 de sep. 2019.

CALDANA, G.; GUIRARDELLO, E.B.; URBANETTO, J.S. et al. Rede Brasileira de Enfermagem e Segurança do Paciente: desafios e perspectivas. **Texto Contexto - Enferm**., Florianópolis, v. 24, n. 3, p. 906-911, 2015. Disponível em:<http://www.scielo.br/pdf/tce/v24n3/pt_0104-0707-tce-24-03-00906.pdf>. Acesso em: 07 de set. 2018.

CALLEGARI, D.C.; BATISTA, H.M.T; SILVEIRA, G.B.M. et al. Monitoring the suspension of surgical procedures. **Arquivos Internacionais de Medicina**, v. 8, p. 1755-7682, 2015. Disponível em:<http://dx.doi.org/10.3823/1710>. Acesso: 20 de [out. 2019.

CAMERINI, F.G.; SILVA, L.D. Segurança do paciente: análise do preparo de medicação intravenosa em hospital da rede sentinela. **Texto Contexto - Enferm**, Florianópolis, v. 20, n. 1, p. 41-49, 2011. Disponível em:<http://www.index-f.com/textocontexto/2011pdf/20-041.pdf>. Acesso em: 05 de jun. 2017.

CAMMAROTA, M.C.; RIBEIRO JUNIOR, I.; LIMA, R.Q. et al. Estudo do uso de pontos de adesão para minimizar a formação de seroma após mastectomia com reconstrução imediata. **Rev. Bras. Cir. Plást**., v. 31, n. 2, p. 158-165, 2016. Disponível em:<http://www.dx.doi.org/10.5935/2177-1235.2016RBCP0026>. Acesso em 12 out. 2019.

CAMPOS, F.P; RÉDUA, T.S; ALVARELI, L.V.G. A influência da cultura organizacional para o desempenho das organizações. **Revista Janus**, n. 14, p. 21-31, 2011. Disponível em:<http://publicacoes.fatea.br/index.php/janus/article/viewFile/974/754>. Acesso em: 22 de nov. 2018.

CANADIAN ASSOCIATION OF WOUND CARE. **Best practice recommendations for the prevention and management of surgical wound complication.** -Canada, Wound Care, 2017. Disponível em:<https://www.woundscanada.ca/docman/public/555-bpr-prevention-and-management-of-surgical-wound-complications-v2>. Acesso em: 18 de set. 2019.

CANET, J.; SABATÉ, S.; MAZO, V. et al. Development and validation of a score to predict postoperative respiratory failure in a multicentre European cohort. **EJA**, v. 32, n. 7, p. 58-7, 2015. Disponível em:<https://journals.lww.com/ejanaesthesiology/Fulltext/2015/07000/Development_and_validation_of_a_score_to_predict.3.aspx>. Acesso em: 20 de out. 2019.

CANTON, E.J.; NOGUEIRA, M.L.P.; FERREIRA, L.A.C. et al. Perfuração intestinal em lipoaspiração: relato de caso. **Rev. Bras. Cir. Plást**., v. 34, n. 3, p. 67-68, 2019. Disponível em:<http://www.rbcp.org.br/details/2490/perfuracao-intestinal-em-lipoaspiracao--relato-de-caso>. Acesso em: 15 de out. 2019.

CAPUCHO, H.C.; CASSIANI, S.H.B. Necessidade de implantar programa nacional de segurança do paciente no Brasil. **Rev. Saúde Pública**, São Paulo, v. 47, n. 4, p. 791-798, 2013. Disponível em:<http://www.scielo.br/scielo.php?script=sci_arttext&pid=S0034-89102013000400791&lng=en&nrm=iso>. Acesso em: 05 de jun. 2019.

CARBONI, R.M.; REPPETTO, M.A.; NOGUEIRA, V.O. Erros no exercício da enfermagem que caracterizam imperícia, imprudência e negligência: uma revisão bibliográfica. **Rev Paul Enferm**, v. 29, n. 1-2-3, p. 100-7, 2018. Disponível em:<http://repen.com.br/revista/wp-content/uploads/2018/12/REPEn_2018_v29n1-2-3_a10.pdf>. Acesso em: 10 de set. 2019.

CARVALHO, P.; GOTTEMS, L.; PIRES, M. et al. Cultura de segurança no centro cirúrgico de um hospital público, na percepção dos profissionais de saúde. **Rev. Latino-Am. Enfermagem**, v. 23, n. 6, p. 1041-1048, 2015. Disponível em:<http://www.periodicos.usp.br/rlae/article/view/108014>. Acesso em: 10 de mar. 2018.

CARVALHO, R.E.F.L.; ARRUDA, L.P.; NASCIMENTO, N.K.P. et al. Avaliação da cultura de segurança em hospitais públicos no Brasil. **Rev. Latino-Am. Enfermagem**, Ribeirão Preto, v. 25, n. 2849, não p., 2017. Disponível

em:<http://dx.doi.org/10.1590/15188345.1600.2849>. Acesso em: 23 de ago. 2019.

CARVALHO, T.A.; SOBRAL, C.B.; MARINHO, P.M.L. et al. Suspensão de cirurgias em um hospital universitário. **Rev SOBECC**. V. 21, n. 4, p. 186-91, 2016. Disponível em:<https://doi.org/10.5327/Z1414-4425201600040002>. Acesso em: 22 de out. 2019.

CARVALHO, T.A.; SOBRAL, C.B.; MARINHO, P.M.L. et al. Suspensão de cirurgias em um hospital universitário. **Rev SOBECC,** São Paulo, v. 21, n. 4, p. 186-191, 2016. Disponível em:<https://revista.sobecc.org.br/sobecc/article/view/46>. Acesso em: 20 de out. 2019.

CARVALHO, V.; CASSIANI, S. Erros na medicação: análise das situações relatadas pelos profissionais de enfermagem. Medicina, v. 33, n. 3, p. 322-330, 2000. Disponível em:<https://doi.org/10.11606/issn.2176-7262.v33i3p322-330>. Acesso em: 20 de mai. 2019.

CASTRO, A.V.; REZENDE, M. A técnica Delphi e seu uso na pesquisa de enfermagem: revisão bibliográfica. **Rev Min Enferm**., v. 13, n. 3, p. 429-34, 2009. Disponível em:<http://www.reme.org.br/artigo/detalhes/209> Acesso em:12 de set. 2018.

CASTRO, M.A.; FREIXES, M.C.; NOVOA, P.M. et al. Guía para el manejo y la prevención de la insuficiencia suprarrenal aguda. **EDN**., S2530-0164, 2019. Disponível em:<https://doi.org/10.1016/j.endinu.2019.01.004>. Acesso em: 19 de out. 2019.

CAUDURO, F.L.F.; VALLE, M.F.; CRUZ, E.D.A. Percepção da cultura de segurança entre médicos. **Rev. Uniandrade**, **(Online)**, Curitiba, v. 18, n. 1, p. 24-34, 2017. Disponível em:<https://www.uniandrade.br/revistauniandrade/index.php/revistauniandrade/article/view/6 68>. Acesso em: 15 de set. 2017.

CAUDURO, F.L.F.; SARQUIS, L.M.M.; CRUZ, EDA. Safety culture among surgical center professional. **Cogitare enferm**., v. 20, n. 1, p. 128-37, 2015. Disponível em:<http://revistas.ufpr.br/cogitare/article/view/36645/24860>. Acesso em: 19 de maio 2018.

CENTERS FOR DISEASE CONTROL AND PREVENTION; NATIONAL HEALTHCARE SAFETY NETWORK (NHSN). **Patient Safety Component Protocol**.-New York-NHSN, 2017b. Disponível em:<https://www.cdc.gov/nhsn/pdfs/pscmanual/pcsmanual_current.pdf>. Acesso em:

15 out. 2019.

CENTERS FOR DISEASE CONTROL AND PREVENTION. **The National Healthcare Safety Network. Surgical Site Infection (SSI) Event. Manual: patient safety component manual** [Internet]. Atlanta: CDC; 2017a. Disponível em:<https://www.cdc.gov/nhsn/pdfs/ pscmanual/pcsmanual_current.pdf>. Acesso em: 15 out. 2019.

CHAKRAVARTY, B.A. A survey of attitude of frontline clinicians and nurses towards adverse events. **Medic J Armed Forces India**, v. 69, n. 4, p. 335-340, 2013. Disponível em:<http://www.mjafi. net/article/S0377-1237(13)00013-0/fulltext>. Acesso em: 10 de set. 2019.

CHAUDHARY, N.; VARMA, V.; KAPOOR, S. et al. Implementation of a surgical safety checklist and postoperative outcomes: a prospective randomized controlled study. **J Gastrointest Surg**., v. 19, n. 5, p. 935-42, 2015. Disponível em:<https://doi.org/10.1007/s11605-015-2772-9>. Acesso de: 29 de nov. 2018.

CLANTON, J.; CLARK, M.; LOGGINS, W. et al. Effective handoff communication. In: Firstenberg MS, Stawicki S, editors. Vignettes in patient safety. **IntechOpen**, v. 2, p. 25-44, 2018. Disponível em:<https://doi.org/10.5772/intechopen.69747>. Acesso em: 15 de jun. 2019.

COHEN, J.; VINCENT, J.L.; ADHIKARI, N.K. et al. Sepsis: a roadmap for future research. **Lancet Infect Dis**, v. 15, n. 5, p. 581-614, 2015. Disponível em:<https://www.thelancet.com/journals/laninf/article/PIIS1473-3099(15)70112-X/fulltext>. Acesso em: 14 de set. 2019.

COLUCI MZO, ALEXANDRE NMC, MILANI D. Construction of measurement instruments in the area of health. **Ciênc. saúde coletiva**. [Internet], v. 20, n. 3, p. 925-936, 2015. Disponível em:<http://dx.doi.org/10.1590/1413-81232015203.04332013>. Acesso em: 22 de set. 2018.

CONRARDY, J.A.; BRENEK, B.; MYERS, S. Determining the state of knowledge for implementing the universal protocol recommendations: an integrative review of the literature. **AORN J.**, v. 92, n. 2, p. 194-207, 2010. Disponível em:<https://www.ncbi.nlm.nih.gov/pubmed/20678608>. Acesso em: 10 de fev. 2019.

CONSELHO FEDERAL DE ENFERMAGEM (COFEN). Enfermagem em Números, 2019. Disponível em:<http://www.cofen.gov.br/enfermagem-em-numeros>. Acesso em: 08 de nov. 2019.

CONSELHO FEDERAL DE ENFERMAGEM (COFEN). Resolução nº 0450/2013. Normatiza o procedimento de Sondagem Vesical no âmbito do Sistema Cofen /Conselhos Regionais de Enfermagem. Brasília: COFEN; 2013. Disponível em:<http://novo.portalcofen.gov.br/resolucao-cofen-no-04502013-4_23266.html.>. Acesso em: 07 nov. 2019.

CONSELHO FEDERAL DE ENFERMAGEM (COFEN). Resolução nº 564 de 6 de novembro de 2017. **Aprova o novo Código de Ética dos Profissionais de Enfermagem (CEPE)**. Diário Oficial da República Federativa do Brasil, Brasília, 2017. Disponível em:<http://www.cofen.gov.br/resolucao-cofen-no-5642017_59145.html>. Acesso em 05/11/2019.

COOKE, C.R.; IWASHYNA, T.J. Sepsis mandates: improving inpatient care while advancing quality improvement. **JAMA**, v. 312, n. 14, p. 1397–8, 2014. Disponível em:<https://jamanetwork.com/journals/jama/article-abstract/1911335>. Acesso em: 14 de set. 2019.

COPELAND, L.A.; GRAHAM, L.A.; RICHMAN, J.S. et al. A study to reduce readmissions after surgery in the Veterans Health Administration: design and methodology. **BMC Health Serv Res.**, v. 17, n. 1, p. 198, 2017. Disponível em:<https://bmchealthservres.biomedcentral.com/articles/10.1186/s12913-017-2134-2>. Acesso em: 19 de out. 2019.

CORRÊA, C.D.T.S.O.; MENDES, W. Proposal of a trigger tool to assess adverse events in dental care. **Cad. Saúde Pública**, v. 33, n. 11, não p., 2017. Disponível em:<https://doi.org/10.1590/0102-311X00053217>. Acesso em: 15 de out. 2019.

CORREGGIO, T.C.; AMANTE, L.N.; BARBOSA, S.F.F. Avaliação da cultura de segurança do paciente em Centro Cirúrgico. **Rev. SOBECC**, São Paulo, v. 19, n. 2, p. 67-73, 2014. Disponível em:<http://www.sobecc.org.br/arquivos/artigos/2015/pdfs/site_sobecc_v19n2/02_sobecc_v19n2.pdf>. Acesso em: 16 de set. 2019.

CORREIA, T.; MARTINS, M.; FORTE, E. Processes developed by managers regarding the errors. **Revista Enfermagem Referência**., v. 4, n. 12, p. 75-84, 2017. Disponível em:<https://doi.org/10.12707/RIV16073>. Acesso em: 10 de ago. 2019.

COSTA, E. A.; MOREIRA, L.; GUSMÃO, M. E. Incidência de eventos adversos cirúrgicos em hospital dia. **Vigilância Sanitária em Debate: Sociedade, Ciência & Tecnologia**, v. 5, n. 2, p. 77-82, 2017. Disponível em:<https://doi.org/10.22239/2317-269x.00865>. Acesso em: 10 de set. 2019.

COSTA, D.B. da.; RAMOS, D.; GABRIEL, C.S. et al. Cultura de segurança do paciente: avaliação pelos profissionais de enfermagem. **Texto Contexto - Enferm**, Florianópolis, v. 27, n. 3, e2670016, 2018. Disponível em:<http://dx.doi.org/10.1590/0104-07072018000267001 6>. Acesso em: 26 de ago. 2019.

COUTO, R.C.; PEDROSA, T.M.G.; ROBERTO. B.A.D. et al. Anuário da segurança assistencial hospitalar no Brasil. **IESS**, não p., 2018. Disponível em:<https://repositorio.observatoriodocuidado.org/handle/handle/1646>. Acesso em: 25 de jan. 2019.

CRONBACH, L. J. Coefficient alpha and the internal structure of tests. Psychometrika, n. 16, p. 297–334. 1951.

CRUZ, E.D.A. Challenges in research into patient safety. **Cogitare enferm.**, v. 21, n. 5, não p., 2016. Disponível em:<http://dx.doi.org/10.5380/ce.v21i5.48090>. Acesso em: 22 de nov. 2018.

CRUZ, E.D.A.; ROCHA, D.J.M.; MAURICIO, A.B. et al. Cultura de segurança entre profissionais de saúde em hospital de ensino. **Cogitare enferm.**, v. 23, n. 1, e50717, 2018. Disponível em:< http://dx.doi.org/10.5380/ce.v23i1.50717>. Acesso em: 05 de jul. 2019.

CURADO, M.A.S.; TELES, J.; MARÔCO, J. Análise de variáveis não diretamente observáveis: influência na tomada de decisão durante o processo de investigação. **Revista da Escola de Enfermagem da USP**, v. 48 n. 1, p. 149-156, 2014. Disponível em:<http://www.scielo.br/pdf/reeusp/v48n1/pt_0080-6234-reeusp-48-01-146.pdf>. Acesso em: 12 de out. 2018.

DAL SASSO; G.T.M.; SOUZA, M. L. A simulação assistida por computador: a convergência no processo de educar-cuidar da enfermagem. **Texto Contexto - Enferm**. v. 20, p. 36-44. Florianópolis, 2011. Disponível em:<http://www.scielo.br/scielo.php?script=sci_arttext&pid=S0104-07072006000200006&lng=en&nrm=iso>. Acesso em: 12 de set. 2018.

DANIELSSON, M.; NILSEN P.; RUTBERG, H. et al. A National Study of patient safety culture in hospitals in Sweden. **J Patient Saf.**, não p., 2017. Disponível em:<https://www.ncbi.nlm.nih.gov/pubmed/28234728>. Acesso em: 15 de set. 2019.

DARBY, J.L.; DAVIS, B.S.; BARBASH, I.J. et al. An administrative model for benchmarking hospitals on their 30-day sepsis mortality. **BMC Health Services Research**, v. 19, n. 211, 2019. Disponíve

em:<https://bmchealthservres.biomedcentral.com/articles/10.1186/s12913-019-4037-x>. Acesso em: 14 de set. 2019.

DAVENPORT, M.C.; DOMÍNGUEZ, P.A.; FERREIRA, J.P. et al Detección de eventos adversos en pacientes pediátricos hospitalizados mediante la Herramienta de Disparadores Globales Global Trigger Tool. **Arch Argent Pediatr**. v. 115, n. 4, p. 357-363, 2017. Disponível em:<https://www.sap.org.ar/uploads/archivos/files_ao_davenport_26-6-17pdf_1498513664.pdf>. Acesso em: 05 de maio 2019.

DAVIES, H.T.; NUTLEY, S.M.; MANNION, R. Organisational culture and quality of health care. **Qual Health Care**, v. 9, n. 2, p. 111-9, 2000. Disponível em:<https://www.ncbi.nlm.nih.gov/pmc/articles/PMC1743521/>. Acesso em: 25 de out. 2018.

DIAS, A.O.; MARTINS, E.A.P.; HADDAD, M.C.L. Instrumento para avaliação da qualidade do Time de Resposta Rápida em um hospital universitário público. **Rev. Bras. Enferm.**, Brasília, v. 67, n. 5, p. 700-707, 2014. Disponível em:<http://www.scielo.br/scielo.php?script=sci_arttext&pid=S0034-71672014000500700&lng=en&nrm=iso>. Acesso: 15 de Out. 2019.

DOYLE, D.J; GARMON, E.H. American Society of Anesthesiologists Classification (ASA Class) In: **StatPearls [Internet]**. Treasure Island (FL): StatPearls Publishing; 2017 Jun-. Disponível em:<https://www.ncbi.nlm.nih.gov/books/NBK441940>. Acesso em: 10 de jun. 2019.

DUARTE, S.C.M.; STIPP, M.A.C.; SILVA, M.M. et al. Adverse events and safety in nursing care. **Rev. Bras.Enferm**, v. 68, n. 1, p. 136-146, 2015. Disponível em:<https://www.ncbi.nlm.nih.gov/pubmed/25946507>. Acesso em: 5 de set. 2019.

DUDECK, M.A.; EDWARDS JUNIOR, R.; ALLEN-BRIDSON, K. et al. National Healthcare Safety Network report, data summary for 2013: device-associated module. **Am J Infect Control** [Internet], v. 43, n. 3, p. 206-21, 2015. Disponível em:<https://www.ncbi.nlm.nih.gov/pmc/articles/PMC4653815>. Acesso em: 12 de out. 2019.

DURANT, D.J. Nurse-driven protocols and the prevention of catheter-associated urinary tract infections: a systematic review. **Am J Infect Control**, v. 45, n. 12, p. 1331-41, 2017. Disponível em:<https://www.ncbi.nlm.nih.gov/pubmed/28982611>. Acesso: 18 de out. 2019.

EIRAS, M.; ESCOVAL, A.; GRILLO, I.M. et al. The hospital survey on patient safety

culture in Portuguese hospitals: instrument validity and reliability. **Int J Health Care Qual Assur**, Bingley, v. 27, n. 2, p. 111–22, 2014. Disponível em:<https://www.ncbi.nlm.nih.gov/pubmed/24745137>. Acesso em: 05 de jul. 2019.

EL-JARDALI, F.; DIMASSI, H.; JAMAL, D.; JAAFAR, M.; HEMADEH, N. Predictors and outcomes of patient safety culture in hospitals. **BMC Health Services Research**, v. 11, n. 1, p. 45, 2011. Disponível em:<https://bmchealthservres.biomedcentral.com/articles/10.1186/1472-6963-11-45>. Acesso em: 05 der jun. 2018.

EL-JARDALI, F.; SHEIKH, F.; GARCIA, A.N. et al. Patient safety culture in a large teaching hospital in Riyadh: baseline assessment, comparative analysis and opportunities for improvement. **BMC Health Ser Res**, Nova Iorque, v. 14, n. 122, não p., 2014. Disponível em:<https://bmchealthservres.biomedcentral.com/articles/10.1186/1472-6963-14-122>. Acesso: 10 de ago. 2019.

ELMONTSRI, M.; ALMASHRAFI, A.; BANARSEE, R. et al. Status of patient safety culture in Arab countries: a systematic review. **BMJ Open**, v. 7, n. 2, p. 013487, 2017. Disponível em:<10.1136/bmjopen-2016-013487>. Acesso em: 05 de set. 2019.

EPSTEIN, J.; SANTO, R. M.; GUILLEMIN, F. A review of guidelines for cross-cultural adaptation of questionnaires could not bring out a consensus. **Jounal of Clim. Epidemiol**, Oxford, v. 68, n. 4, p. 435-441, 2015. Disponível em:<https://www.ncbi.nlm.nih.gov/pubmed/25698408>. Acesso em: 10 de jan. 2019.

ESHER, Â.; SANTOS, E.M.; MAGARINOS-TORRES, R. et al. Construindo critérios de julgamento em avaliação: especialistas e satisfação dos usuários com a dispensação do tratamento do HIV/AIDS. **Ciênc Saúde coletiva**. v. 17, n. 1, p. 203-214, 2012. Disponível em:<http://www.scielo.br/scielo.php?script=sci_arttext&pid=S1413-81232012000100022&lng=en&nrm=iso>. Acesso em: 08 de set. 2018.

FAN, C.J.; PAWLIK, T.M.; DANIELS, T. et al. Association of Safety Culture with Surgical Site Infection Outcomes. **J Am Coll Surg.**, v. 222, n. 2, não p., 2016. Disponível em:<https://doi.org/ 10.1016/j.jamcollsurg.2015.11.008>. Acesso em: 02 sep. 2019.

FASSINI, P.; HAHN, G.V. Riscos à segurança do paciente em unidade de internação hospitalar: concepções da equipe de enfermagem. **Revista de Enfermagem da UFSM**, Rio Grande do Sul, v. 2, n. 2, p. 290-299, 2012. Disponível em:<http://cascavel.ufsm.br/revistas/ojs-2. 2.2/index.php/reufsm/article/view/4966>. Acesso em: 16 de abr. 2017.

FERRAZ, E.M. A cirurgia segura. Uma exigência do século XXI. **Rev. Col. Bras. Cir,** v. 36, n. 4, p. 281-282, 2009. Disponível em:<http://www.scielo.br/pdf/rcbc/v36n4/a01v36n4.pdf>. Acesso em: 20 de mar. 2018.

FIGUEIREDO, M.A.G.; PERES, M.A.A. Identidad de la enfermera: una reflexión desde la perspectiva de Dubar. **Rev. Enf. Ref**., Coimbra, v. serI, n. 20, p. 149-154, 2019. Disponível em:<http://www.scielo.mec.pt/scielo.php?script=sci_arttext&pid=S0874028320190001000016&lng=pt&nrm=iso>. Acesso em: 07 de out. 2019.

FIKATAS, P.; SCHOENING, W.; LEE, J.E. et al. Incidence, risk factors and management of incisional hernia in a high-volume liver transplant center. **Ann Transplant**, v. 16, n. 18, p. 223-30, 2013. Disponível em:<https://www.ncbi.nlm.nih.gov/pubmed/23792524>. Acesso em: 02 de out. 2019.

FLEISCHMANN, C.; SCHERAG, A.; ADHIKARI, N.K.J. et.al. Assessment of global incidence and mortality of hospital-treated Sepsis. Current estimates and limitations. **Am J Respir Crit Care Med**., v. 193, n. 3, p. 259–72, 2016. Disponível em:<https://www.atsjournals.org/doi/full/10.1164/rccm.201504-0781OC>. Acesso em: 15 de set. 2019.

FLIN, R.; BURNS, C.; MEARNS, K. et al. Measuring safety climate in health care. **Qual Saf Heal Care** [Internet]. v. 15, n. 2, p. 109-115, 2006. Disponível em:<https://www.ncbi.nlm.nih.gov/pmc/articles/PMC2464831>. Acesso em: 25 de out. 2018.

FLIN, R.H.; PATEY, R. E.; GLAVIN, R.J. et al. Anaesthetists' non-technical skills, BJA: British. **Journal of Anaesthesia**, v. 105, n. 1, p. 38–44, 2010. Disponível em:< https://doi.org/10.1093/bja/aeq134>. Acesso em: 19 de nov. 2018.

FREITAS M.R. de; ANTUNES A.G.; LOPES B.N.A. et al. Avaliação da adesão ao checklist de cirurgia segura da OMS em cirurgias urológicas e ginecológicas, em dois hospitais de ensino de Natal, Rio Grande do Norte, Brasil. **Cad. Saúde Pública**, v. 30, n. 1, p. 137-148, 2014. Disponível em:<http://dx.doi.org/10.1590/0102-311X00184612>. Acesso em: 12 de ago. 2019.

GABRANI J.C.; KNIBB W.; PETRELA E. et al. Provider Perspectives on Safety in Primary Care in Albania. **Journal of Nursing Scholarship**. v. 48, n. 6, p. 552-560, 2016. Disponível em:<https://doi.org/10.1111/jnu.12236>. Acesso em: 11 de set. 2019.

GAGLIARDI, A.R.; STRAUS, S.E.; SHOJANIA, K.G. et al. Multiple interacting factors influence adherence, and outcomes associated with surgical safety checklists: a Qualitative Study. **PLOS ONE**, n. 26, p. 9, 2014. Disponível em:<https://doi.org/10.1371/journal.pone.0108585>. Acesso em: 6 de out. 2019.

GAMA, Z. A. S.; OLIVEIRA, A. C. S.; HERNÁNDEZ, P. J. S. Cultura de seguridad del paciente y factores asociados en una red de hospitales públicos españoles. **Cad. Saúde Pública,** Rio de Janeiro, v. 36, n. 4, p. 43-48, 2013. Disponível em:<http://www.scielo.br/pdf/csp/v29n2/15.pdf>. Acesso em: 10 de jun. 2019.

GAMBASHIDZE, N.; HAMMER, A.; BROTERHAUS, M. et al. Evaluation of psychometric properties of the German Hospital Survey on Patient Safety Culture and its potential for cross-cultural comparisons: a cross-sectional study. **BMJ Open**, v. 7, e018366, 2017. Disponível em:<https://bmjopen.bmj.com/content/7/11/e018366>. Acesso em: 06 de set. 2019.

GANDHI, T.K.; BERWICK, D.M.; SHOJANIA, K.G. Patient safety at the crossroads. **JAMA**, Boston, v. 315, n. 17, p. 1829-30, 2016. Disponível em:<https://jamanetwork.com/journals/jama/article-abstract/2518272>. Acesso em: 03 de mar. 2018.

GAWANDE, A. **Checklist: como fazer as coisas bem feitas**. Rio de Janeiro: Sextante, 2011.

GERSHON, R.R.M.; KARKASHIAN, C.D.; GROSCH, J.W. et al. Hospital safety climate and its relationship with safe work practices and workplace exposure incidents. **American Journal of Infection Control**, v. 28, n. 3, p. 211-221, 2000. Disponível:<https://www.ajicjournal.org/article/S0196-6553(00)18842-X/fulltext>. Acesso em: 02 jun. 2019.

GILES, K.; MUNN, Z.; AROMATARIS, E. et al. Use of surgical safety checklists in Australian operating theatres: an observational study. **ANZ Journal of Surgery**, v. 87, n. 12, p. 971-975, 2017. Disponível em:<https://doi.org/10.1111/ans.13638>. Acesso em: 06 de out. 2019.

GIOVINAZZO, R. A. Modelo de Aplicação da Metodologia Delphi pela Internet - Vantagens e Desvantagens. **FEA USP**, v. 2, n. 2, não p., 2001. Disponível em:<http://www.fecap.br/adm_online/art22/renata.htm>. Acesso em: 10 de set. 2018.

GOMES, A.T.L.; ASSIS, Y.M.S.; FERREIRA, L.L. et al. Tecnologias aplicadas à segurança do paciente: uma revisão bibliométrica. **RECO**, Divinópolis, v. 7, e1473, 2017. Disponível

em:<http://seer.ufsj.edu.br/index.php/recom/article/viewFile/1473/1719>. Acesso em: 10 de jul. 2019.

GOMES, J.R.A.A.; FRANCO, R.V.B.; MORAIS, D.S.V.D. et al. Fatores determinantes para suspensões de cirurgias eletivas em um hospital do Distrito Federal. **Revista SOBECC**, São Paulo, v. 23, n. 4, p. 184-188, 2018. Disponível em:<https://sobecc.emnuvens.com.br/sobecc/article/view/419>. Acesso em: 28 out. 2019.

GOMES, S. M. **A efetiva prevenção e reparação do dano cirúrgico: descompasso entre as diretrizes públicas de saúde para a segurança do paciente e a tendência jurisprudencial brasileira.** In: CASTRO G.F. (J.d. Organizadores.). Aspectos, opiniões e destaques sobre segurança. Belo Horizonte, MG: Edições Superiores, 2012. p. 247

GOULART, L.S.; FERREIRA JÚNIOR, M.A.; SARTI, E.C.F.B. et al. Os enfermeiros estão atualizados para o manejo adequado do paciente com sepse? **Esc. Anna Nery**, Rio de Janeiro, v. 23, n. 4, não p., 2019. Disponível em:<http://dx.doi.org/10.1590/2177-9465-ean-2019-0013>. Acesso em: 13 de out. 2019.

GÖZLÜ, K.; KAYA, S. Patient safety culture asp by nurses in a Joint Commission International Accredited Hospital in Turkey and its comparison with Agency for Healthcare Research and Quality Data. **J Patient Saf Qual Improv**., v. 4, n. 4, p. 441-9, 2016. Disponível em:<http://dx.doi.org/10.22038/PSJ.2016.7640>. Acesso em: 12 de ago. 2019.

GRAHAM, L.A.; MULL, H.J.; WAGNER, T.H. et al. Comparison of a Potential Hospital Quality Metric With Existing Metrics for Surgical Quality–Associated Readmission. **JAMA**., v. 2, n. 4, não p., 2019. Disponível em:<https://jamanetwork.com/journals/jamanetworkopen/fullarticle/2730777>. Acesso em: 10 de out. 2019.

GRIFFIN, F.A.; CLASSEN, D.C**.** Detection of adverse events in surgical patients using the Trigger Tool approach. **BMJ Quality & Safety**, v. 17, n. 4, p. 253-258, 2008. Disponível em:<http://dx.doi.org/10.1136/qshc.2007.025080>. Acesso em: 16 abr. 2019.

GRIFFIN, F. A.; RESAR, R. K.; IHI Global Trigger Tool for Measuring Adverse Events 2.ed. **IHI**, Cambridge Massachusetts: Institute for Healthcare Improvement, 2009. Disponível em:<http://app.ihi.org/webex/gtt/ihiglobaltriggertoolwhitepaper2009.pdf>. Acesso em: 02 de out. 2019.

GRIFFITHS, P.; BALL, J.; MURRELLS, T. et al. Registered nurse, healthcare support worker, medical staffing levels and mortality in English hospital trusts: a cross-sectional study. **BMJ Open**, v. 6, n. 2, não p., 2016. Disponível em:<http://bmjopen.bmj.com/content/6/2/e008751.full>. Acesso em: 02 de jun. 2018.

GRIGOLETO, A.R.L.; GIMENES, F.R.E.; AVELAR M.C.Q. Segurança do cliente e as ações frente ao procedimento cirúrgico. **Revista Eletronica de Enfermagem**, v. 13, n. 2, p. 347-54, 2011. Disponível em:<https://www.revistas.ufg.br/fen/article/view/10326/9642>. Acesso em: 02 de jun. 2018.

GRONKJAER, M.; ELIASEN, M.; SKOV-ETTERUP, L. et al. Preoperative smoking status and postoperative complications. **Ann Surg**., v. 259, n. 1, p. 52–71, 2014. Disponível em:<https://www.ncbi.nlm.nih.gov/pubmed/23799418>. Acesso em: 14 de out. 2019.

GROSSE-SUNDRUP, M.; HENNEMAN, J.P.; SANDBERG, W.S. et al. Intermediate acting non-depolarizing neuromuscular blocking agents and risk of postoperative respiratory complications: prospective propensity score matched cohort study. **BMJ**, v. 345, e6329, 2012. Disponível em:<https://www.bmj.com/content/345/bmj.e6329>. Acesso em: 03 de out. 2019.

GUALANDRO, D.M.; CALDERARO, D.; YU, P.C. et al. Infarto agudo do miocárdio após operações não cardíacas. **Arq. Bras. Cardiol**. São Paulo, v. 99, n. 5, p. 1060-1067, 2012. Disponível em:<http://www.scielo.br/scielo.php?script=sci_arttext&pid=S0066-782X2012001400013&lng=en&nrm=iso>. Acesso em: 29 de out. 2019.

GUILLAUME, D.; CRAWFORD, S.; QUIGLEY, P. Characteristics of the middle-age adult inpatient fall. **Appl Nurs Res**., v. 31, p. 65-71, 2016. Disponível em:<https://doi.org/10.1016/j.apnr.2016.01.003>. Acesso em: 15 de out. 2019.

GUILLEMIN, F.; BOMBARDIER, C.; BEATON, D. Cross- Cultural Adaptation of Health- Related Quality of Life Measures: Literature Review and Proposed Guidelines. **J Clin Epidemiol**., v. 46, n. 12, p. 1417-32, 1993. Disponível em:<https://www.jclinepi.com/article/0895-4356(93)90142-N/pdf>. Acesso em: 05 de out. 2019.

GULDENMUND, F. The nature of safety culture: a review of theory and research. **J Saf. Sci**. [Internet], v. 34, n. 1-3, p. 215-57, 2000. Disponível em:<http://www.sciencedirect.com/science/article/pii/S092575350000014X>. Acesso em: 10 de maio 2018.

HADDAD N. Metodologia de estudos em ciências da saúde. 1. ed. **Roca**, São Paulo, v. 13, n. 1, p. 223-245, 2004. Disponível em:<file:///C:/Users/User/Downloads/847-962-1-PB%20(2).pdf>. Acesso em: 02 de jun. 2018.

HALFON, P.; STAINES, A.; BURNAND, B. Adverse events related to hospital care: a retrospective medical records review in a Swiss hospital. **International Journal for Quality in Health Care**, v. 29, n. 4, p. 527–533, 2017. Disponível em:<https://doi.org/10.1093/intqhc/mzx061>. Acesso em: 12 de out. 2019.

HALLIGAN, M.; ZECEVIC, A. Safety Culture in Healthcare: a review of concepts, dimensions, measures and progress. **BMJ Qual Saf**. v. 20, n. 4, p. 338-43, 2011. Disponível em:<https://www.ncbi.nlm.nih.gov/pubmed/21303770>. Acesso em: 12 de set. 2018.

HAMDAN, M.; SALEEM, A. A. Changes in Patient Safety Culture in Palestinian Public Hospitals: impact of Quality and Patient Safety Initiatives and Programs. **J Patiet Saf**, v. 14, n. 3, p. e67 – e73, Sep. 2018. Disponível em:<https://insights.ovid.com/pubmed?pmid=29985885>. Acesso em: 12 de set. 2018.

HANDLER, S.M.; CASTLE, N.G.; STUDENTSKI, S.A. et al. Patient safety culture assessment in the nursing home. **Qual Saf Health Care**, v. 15, n. 6, p. 400-4, 2006, Disponível em:<https://www.ncbi.nlm.nih.gov/pubmed/17142586>. Acesso em: 08 de set. 2018.

HARKANEN, M.; KERVINEN, M.; AHONEN, J. et al. Patient-specific risk factors of adverse drug events in adult inpatients- evidence detected using the Global Trigger Tool method. **J Clin Nurs**. [Internet], v. 24, n. x, p. 582-591, 2015. Disponível em:<https://doi.org/10.1111/jocn.12714>. Acesso em: 16 de out. 2019.

HAUGEN, A.S.; WAEHLE, H.V.; ALMELAND, S.K. et al. Causal Analysis of World Health Organization’s Surgical Safety Checklist Implementation Quality and Impact on Care Processes and Patient Outcomes: Secondary Analysis From a Large Stepped Wedge Cluster Randomized Controlled Trial in Norway. **Ann Surg**, v. 269, n. 2, p. 283–90, 2019. Disponível em:<https://www.ncbi.nlm.nih.gov/pubmed/29112512>. Acesso em: 5 de ago. 2019.

HEFNER, J.L.; HILLIGOSS, B.; KNUPP, A.; et al. Cultural transformation after implementation of a crew resource management: is it really possible? **Am J Med Qual**, Tucson, v. 32, n. 4, p. 384–90, 2017. Disponível em:<https://www.ncbi.nlm.nih.gov/pubmed/27422314>. Acesso em: 05 de jul. 2019.

HEMPEL, S.; MAGGARD-GIBBONS, M.; NGUYEN, D.K. et al. Wrong-Site Surgery, Retained Surgical Items, and Surgical Fires A Systematic Review of Surgical Never Events. **JAMA Surg**, v. 150, n. 8, p. 796–805, 2015. Disponível em:<https://www.ncbi.nlm.nih.gov/pubmed/26061125>. Acesso em: 29 de dez. 2018.

HENRIQUES, A.H.B.; COSTA, S.S. da; LACERDA, J.S. Assistência de enfermagem na segurança do paciente cirúrgico: revisão integrativa. **Cogitare enferm.**, v. 21, n. 4, p. 1-9, 2016. Disponível em:<http://www.redalyc.org/articulo.oa?id=483653833023>. Acesso em: 05 de jun. 2017.

HERDMAN, T.H. **Nursing diagnoses: definitions and classification 2012-2014**. Oxford: Wiley-Blackwell. 2012.

HERSHEY, T.B.; KAHN, J.M. State Sepsis mandates - a new era for regulation of hospital quality. **N Engl J Med**, v. 376, n. 24, p. 2311-2313, 2017. Disponível em:<https://www.ncbi.nlm.nih.gov/pubmed/28528558>. Acesso em: 05 de jun. 2017.

HIBBERT, P.D.; MOLLOY, C.J.; HOOPER, T.D. et al. The application of the Global Trigger Tool: a systematic review. **International Journal for Quality in Health Care**, v. 28, n. 6, p. 640-649, 2016. Disponível em:<https://doi.org/10.1093/intqhc/mzw115>. Acesso em: 02 de out. 2019.

HOCHMAN, B.; NAHAS, F.X.; OLIVEIRA FILHO, R.S. et al. Desenhos de pesquisa. **Acta Cir. Bras.**, São Paulo, v. 20, n. 2, p. 2-9, 2005. Disponível em:<http://www.scielo.br/scielo.php?script=sci_arttext&pid=S010286502005000800002&lng=en&nrm=is>. Acesso em: 17 de set. 2019.

HOFSTEDE, G. Dimensionalizing Cultures: The Hofstede Model in Context. **Online Readings in Psychology and Culture**, v. 2, n. 1, não p., 2011. Disponível em:<https://doi.org/10.9707/2307-0919.1014>. Acesso em: 11 de set. 2018.

HOOPER, A.J.; TIBBALLS, J. Comparison of a Trigger Tool and voluntary reporting to identify adverse events in a paediatric intensive care unit. **Anaesth Intensive Care**. [Internet], v. 42, n. 2, p. 163-164, 2014. Disponível em:<http://dx.doi.org/10.1177/0310057X1404200206>. Acesso em: 02 de set. 2019.

HORI, Y.; NAKAYAMA, A.; SAKAMOTO, A. Surgery cancellations after entering the operating room. **JA Clinical Reports**, v. 23, n. 4, p. 184-188, 2016. Disponível em:<https://doi.org/10.1186/s40981-016-0066-1>. Acesso em: 20 de out. 2019.

HOWELL, A.M.; PANESAR, S.S.; BURNS, E.M. et al. Reducing the burden of surgical harm. A systematic review of the interventions used to reduce adverse events in surgery. **Ann Surg**., v. 259, p. 630–641, 2014. Disponível em:<https://www.thelancet.com/journals/landia/article/PIIS2213-8587(14)70142-1/fulltext>. Acesso em: 20 de out. 2019.

HUDELSON, P.M. Culture and quality: an anthropological perspective. **Int J Qual Health Care.** [Internet], v. 16, n. 5, p. 345-6, 2004. Disponível em:<http://www.ncbi.nlm.nih.gov/pubmed/15375093>. Acesso em: 07 de jun. 2018.

HU, Q.; QIN, Z.; ZHAN, M. et al. Development of a trigger tool for the detection of adverse drug events in Chinese geriatric inpatients using the Delphi method. **Int J Clin Pharm**., v. 41, n. 2, p. 1174–1183, 2019. Disponível em:<https://doi.org/10.1007/s11096-019-00871-x>. Acesso em: 20 de out. 2019.

HU, Q.; WU, B.; ZHAN, M. et al. Adverse events identified by the global trigger tool at a university hospital: A retrospective medical record review. **J Evid Based**, v. 12, n. 2, p. 91-97, 2019. Disponível em:<https://doi.org/10.1111/jebm.12329>. Acesso em: 20 de out. 2019.
IMPERÍCIA. In: DICIONÁRIO PRIBERAM DA LÍNGUA PORTUGUESA. Disponível em:<https://dicionario.priberam.org/imper%C3%ADcia>. Acesso em: 05 de nov. 2019.

HWANG, J.; CHIN, H.J.; CHANG, Y. Characteristics associated with the occurrence of adverse events: a retrospective medical record review using the Global Trigger Tool in a fully digitalized tertiary teaching hospital in Korea. **J Eval Clin Pract**., v. 20, n. 1, p. 27-35, 2014. Disponível em:<https://doi.org/10.1111/jep.12075>. Acesso em: 03 de maio 2019

HWANG, J.; KIM, J.; PARK, J.W. Adverse Events in Korean Traditional Medicine Hospitals: A Retrospective Medical Record Review. **J Patiet Saf**., v. 14, n. 3., p. 157-163, 2018. Disponível em:<https://insights.ovid.com/pubmed?pmid=26001551>. Acesso em: 05 de nov. 2019.

INTERNATIONAL ATOMIC ENERGY AGENCY (IAEA). **Safety Culture**-IAEA, 2015. Disponível em:<http://www-ns.iaea.org/tech-areas/operational-safety/safety-culture-home.asp>. Acesso em: 30 nov. 2018.

JAMES, P.; OPARIL, S.; CARTER, B. et al. Evidence-based guideline for the management of the high blood pressure in adults. Report from the panel members appointed to the Eighth Joint National Committee (JNC 8). **JAMA**, v. 311, n. 5, p. 507-520, 2014. Disponível em:<https://jamanetwork.com/journals/jama/fullarticle/1791497>. Acesso em: 10 de jun. 2019.

JAMMER, I.B.; WICKBOLDT, N.; SANDER, M. et al. Standards for definitions and use of outcome measures for clinical effectiveness research in perioperative medicine European Perioperative Clinical Outcome (EPCO) definitions. A statement from the ESA-ESICM joint taskforce on perioperative outcome measures. **Eur. J Anaesthesiol**, v. 32, n. 2, p. 88-105, 2015. Disponível em:<https://journals.lww.com/ejanaesthesiology/fulltext/2015/02000/Standards_for_definitions_and_use_of_outcome.6.aspx>. Acesso em: 20 de out. 2019.

JESUS, A.D.M. Intervenção e acompanhamento do grupo de pele no tratamento da deiscência de sutura em ferida operatória: relato de caso. **Rev UNILUS Ensino e Pesquisa**, v. 15, n. 41, p. 114, 2018. Disponível em:<http://revista.lusiada.br/index.php/ruep/article/view/1070/u2018v15n41e1070>. Acesso em: 10 de out. 2019.

JOINT COMMISSION INTERNATIONAL. **Joint Commission International Accreditation Standards for Hospitals.** 5. Ed.-Oakbrook Terrace-JCI, 2013. Disponível em:<https://www.jcrinc.com/assets/1/14/JCIH14_Sample_Pages.pdf>. Acesso em: 13 de nov. 2018.

JHA, A.K.; LARIZGOITIA, I.; AUDERA-LOPEZ, C. et al. The global burden of unsafe medical care: analytic modelling of observational studies. **BMJ quality & safety**, v. 22, n. 10, p. 809-815, 2013. Disponível em:<http://qualitysafety.bmj.com/content/qhc/22/10/809.full.pdf>. Acesso em: 04 de maio 2017.

KAWAMOTO, A.M.; OLIVEIRA, de J.L.C.; TONINI N.S. et al. Liderança e cultura de segurança do paciente: percepções de profissionais em um hospital universitário. **Rev. Online de Pequisa**, v. 8, n. 2, p. 4387-4398, 2016. Disponível em:<http://www.seer.unirio.br/index.php/cuidadofundamental/article/view/4530/pdf_188>. Acesso em: 10 de ago. 2019.

KEENEY, S.; HASSON, F.; MCKENNA, H. Consulting the oracle: ten lessons using the Delphi technique in nursing research. **Journal of Advanced Nursing**, v. 53 n. 2, p. 205-212, 2006. Disponível em:< https://onlinelibrary.wiley.com/doi/abs/10.1111/j.1365-2648.2006.03716.x>. Acesso em: 10 de ago. 2019.

KELLICUT, D.C.; KUNCIR, E.J.; WILLIAMSON, H.M. et al. Surgical Team Assessment Training: improving surgical teams during deployment. **Am J Surg**., v. 208, n. 2, p. 275-83, 2014. Disponível em:<https://www.americanjournalofsurgery.com/article/S0002-9610(14)00211-6/fulltext>. Acesso em: 16 de ago. 2019.

KENNERLY, D.A.; KUDYAKOV, R.; GRACA, B. et al. Characterization of Adverse Events Detected in a Large Health Care Delivery System Using an Enhanced Global Trigger Tool over a Five-Year Interval. **Health Serv. Res**. [Internet], v. 49, n. 5, p. 1407–1425, 2014. Disponível em:<http://europepmc.org/articles/pmc4213042>. Acesso em: 13 set. 2019.

KHATER, W.A.; AKHU-ZAHEYA, L.M.; AL-MAHASNEH, S.I. et al. Nurses' perceptions of patient safety culture in Jordanian hospitals. **Int Nurs Rev**, Oxford, v. 62, n. 1, p. 82–91, 2015. Disponível em:<https://www.ncbi.nlm.nih.gov/pubmed/25439981>. Acesso em: 10 de ago. 2019.

KIAEI, M.Z; ZIAEE, A; MOHEBBIFAR, R et al. Patient safety culture in teaching hospitals in Iran: assessment by the Hospital Survey on Patient Safety Culture (HSOPSC). **J Health Man Info**, Isfahan, v. 3, n. 2, p. 51-56, 2016. Disponível em:<http://applications.emro.who.int/imemrf/J_Health_Manag_Inform/J_Health_Manag_Inform_2016_3_2_51_56.pdf>. Acesso em: 05 de jul. 2019.

KOHN, L.T.; CORRIGAN, J.M.; DONALDSON, M.S. **To Err is Human: building a safer health system:** Committee on Quality of Health Care in America, Institute of Medicine, Washington: National Academy Press. 2000.

KRAUSE R.T.; HIDLEY J.H. **Taking the Lead in Patient Safety: How Healthcare Leaders Influence Behaviour and Create Culture**. New Jersey: Published by John Wiley & Sons. 2009. Hardback 287pp.

KUNSCH, M. M. K. **Comunicação organizacional estratégica: aportes conceituais e aplicados**. São Paulo: Summus. 2016.
LAUS, A.M.; MENEGUETI, M.G.; SANTOS, J.A. et al. Perfil das quedas em pacientes hospitalizados. **Ciênc Cuid Saúde**, v. 13, n. 4, p. 688-95, 2014. Disponível em:<http://www.periodicos.uem.br/ojs/index.php/CiencCuidSaude/article/view/19234/pdf_250>. Acesso em: 20 de nov. 2018.

LEAPE, L.L. O enigma da lista de verificação. **N. Engl. J. Med**., v. 370, n. 11, p. 1063-4, 2014. Disponível em:<https://www.nejm.org/doi/10.1056/NEJMe1315851>. Acesso em: 6 de out. 2019.

LEE, S.H.; PHAN, P.H.; DORMAN, T. et al. Handoffs, safety culture, and practices: evidence from the hospital survey on patient safety culture. **BMC Health Services Research**, v. 16, n. 254, p. 1-8, 2016. Disponível em:<https://bmchealthservres.biomedcentral.com/articles/10.1186/s12913-016-1502-7>. Acesso em: 02 de set. 2019.

LEHMAN, S.M.; DONLAN, R.M. Bacteriophage-mediated control of a two-species biofilm formed by microorganisms causing catheter-associated urinary tract infections in an in vitro urinary catheter model. **Antimicrob Agents Chemother** [Internet]. v. 59, n. 2, p. 1127-37, 2015. Disponível em:<https://www.ncbi.nlm.nih.gov/pmc/articles/PMC4335898/>. Acesso em: 17 de out. 2019.

LETAIEF, M.; EL-MHAMDI, S.; EL-ASADY, R. et al. A. Adverse events in a Tunisian hospital: results of a retrospective cohort study. **Int J Qual Health Care**, v. 22, n. 5, p. 380-85, 2010. Disponível em:<www.ncbi.nlm.nih.gov/pubmed/20685729>. Acesso 02 jun. 2017.

LEVY, M.M.; RHODES, A.; PHILLIPS, G.S. et al. Surviving Sepsis campaign: association between performance metrics and outcomes in a 7.5-year study. **Crit Care Med**, v. 43, n. 1, p. 3–12, 2015. Disponível em:<10.1097/CCM.0000000000000723>. Acesso em: 05 de out. 2019.

LI, C.; YANG, W.H.; ZHOU, J. et al. Risk factors for predicting postoperative complications after open infrarenal abdominal aortic aneurysm repair: results from a single vascular center in China. **J Clin Anesth**, v. 25, n. 5, p. 371-8, 2013. Disponível em:< https://doi.org/10.1016/j.jclinane.2013.01.013>. Acesso em: 15 de out. 2019.

LIKERT, R. A technique for the measurement of attitudes. Archives of Psychology. v. 22, n. 140 p. 1-50, 1932.

LIMA, C.A.; SANTOS, B.T.P.S.; ANDRADE, D.L.B. et al. Quality of emergency rooms and urgent care services: user satisfaction. **Einstein**, São Paulo, v. 13, n. 4, p. 587-593, 2015. Disponível em:<http://dx.doi.org/10.1590/S1679-45082015GS3347>. Acesso em: 02 de ago. 2019.

LINO, C.R.M.; BRÜGGEMANN, O.M.; SOUZA, M.L. et al. The cross-cultural adaptation of research instruments conducted by nurses in Brazil: an integrative review. **Texto Contexto - Enferm**, v. 26, n. 4, não p., 2017. Disponível em:<http://dx.doi.org/10.1590/0104-07072017001730017>. Acesso em: 10 de jan. 2019.

LI, P.K.T.; BURDMANN, E.A.; MEHTA, R.L. Injúria renal aguda um alerta global. **J Bras Nefrol**, v. 35, n. 1, p. 1-5, 2013. Disponível em:<http://dx.doi.org/10.5935/01012800.20130001>. Acesso em: 02 de out. 2019.

LIU, Y.; PLEASANTS, R.A.; CROFT, J.B. et al. Smoking duration, respiratory symptoms, and COPD in adults aged ≥45 years with a smoking history. **Int J Chron Obstruct Pulmon Dis.**, v. 10, p. 1409-1416, 2015. Disponível em:<https://www.ncbi.nlm.nih.gov/pmc/articles/PMC4516194/#!po=69.0476>. Acesso em: 15 de out. 2019.

LO, E.; NICOLLE, L.E.; COFFIN, S.E. et al. Strategies to prevent catheter-associated urinary tract infections in acute care hospitals: 2014 update. **Infect Control Hosp Epidemiol,** v. 5, n. 35, p. 464-79, 2014. Disponível em:<https://www.ncbi.nlm.nih.gov/pubmed/24709715>. Acesso em: 07 de out. 2019.

LONGO, D.R.; HEWETT, J.E., GE, B. et al. The long road to patient safety. **J Am Med Assoc.** v. 294, n. 22, p. 2858-65. 2005

LORENZINI, E.; SANTI, J.A.R.; BÁO, A.C.P. Patient safety: analysis of the incidents notified in a hospital, in south of Brazil. **Rev Gaúcha Enferm** [Internet], v. 35, n. 2, p. 121-7, 2014. Disponível em:<http://www.scielo.br/scielo.php?script=sci_arttext&pid=S1983-14472014000200121&lng=en&nrm=iso&tlng=en>. Acesso em: 10 de jan. 2019.

LOURENÇÃO, D.C.A. TRONCHIN, D.M.R. Patient safety in the surgical environment: translation and cross-cultural adaptation of validated instrument. **Acta Paul Enferm**., v. 29, n. 1, p. 1-8, 2016. Disponível em:<http://www.scielo.br/pdf/ape/v29n1/en_1982-0194-ape-29-01-0001.pdf>. Acesso em: 19 de jun. 2018.

LUMB, A.B. **Nunn's Applied Respiratory Physiology**. 8 ed. Londres: Elsevier. 2016.

LUZIA, M.F.; PRATES C.G.; BOMBARDELLI, C.F. et al. Características das quedas com dano em pacientes hospitalizados. **Rev. Gaúcha Enferm**., v. 40, e20180307, 2019. Disponível em:<http://www.scielo.br/scielo.php?script=sci_arttext&pid=S198314472019000200408&lng=en&nrm=iso>. Acesso em: 25 de out. 2019.

MACEDO, J.M.; KANO, J.A.; BRAGA, E.M. et al. Cancelamento de cirurgias em um hospital universitário: causas e tempo de espera para novo procedimento. **Rev SOBECC.** v. 18, n. 1, p. 26-34, 2013. Disponível em:<http://www.sobecc.org.br/arquivos/artigos/2012/pdf/Artigos-Cientificos/Ano18_n1_jan_mar2013_cancelamento-de-cirurgias-em-um-hospital-universitario.pdf>. Acesso em: 14 de out. 2019.

MAGALHÃES, A.M.M.; DALL'AGNOL, C.M.; MARCK, P.B. Carga de trabalho da

equipe de enfermagem e segurança do paciente-estudo com método misto na abordagem ecológica restaurativa. **Rev. Latino-Am. Enfermagem**., v. 21, p. 146-154, 2013. Disponível em:<http://www.scielo.br/scielo.php?script=sci_arttext&pid=S0104-11692013000700019&lng=en&tlng=en>. Acesso em: 01 de jun. 2017.

MAIA, C.S.; FREITAS, D.R.C. de; GALLO, L.G. et al. Registry of adverse events related to health care that results in deaths in Brazil. **Epidemiol. Serv. Saúde**, Brasília, v. 27, n. 2, p. 150-63, 2018. Disponível em:<http://dx.doi.org/10.5123/s1679-49742018000200004>. Acesso em: 19 de set. 2019.

MALLOULI, M.; TLILI, M.A.; AOUICHA, W. et al. Assessing patient safety culture in Tunisian operating rooms: a multicenter study. **Int J Qual Health Care**, v. 29, n. 2, p. 176-182, 2017. Disponível em:<https://www.medscape.com/medline/abstract/28073903>. Acesso em: 8 de set. 2019.

MARDON, R.E.; KHANNA, K.M.A.; SORRA. J.et al. Exploring Relationships Between Hospital Patient Safety Culture and Adverse Events. **Journal of Patient Safety**, v. 6, n. 4, p. 226-232, 2010. Disponível em:<https://journals.lww.com/journalpatientsafety/Abstract/2010/12000/Exploring_Relationships_Between_Hospital_Patient.6.aspx>. Acesso em: 04 de nov. 2018.

MARINHO, M.M.; RADUNZ, V.; BARBOSA, S.F.F. Avaliação da cultura de segurança pelas equipes de enfermagem de unidades cirúrgicas **Texto Contexto - Enferm**, Florianópolis, v. 23, n. 3, p. 581-590, 2014. Disponível em:<http://www.scielo.br/scielo.php?script=sci_arttext&pid=S0104-07072014000300581&lng=en&nrm=iso&tlng=pt>. Acesso em: 16 de set. 2017.

MARINHO, M.; RADUNZ, V.; TOURINHO, F. et al. Intervenções educativas e seu impacto na cultura de segurança: uma revisão integrativa. **Enfermagem Foco**, v. 7, n. 2, p. 72-77, 2016. Disponível em:<http://revista.cofen.gov.br/index.php/enfermagem/article/view/799%20v.%207,%20n.2,%20p.%2072-77,%202016>. Acesso em: 12 de mar. 2018.

MARQUES, G.S.; ALMEIDA, P.F.; FARIAS, L.R.C. et al. Estudo preliminar sobre registros de deiscência de feridas operatórias em um hospital universitário. **Revista HUPE**, v. 15, n. 4, p. 312, 2016. Disponível em:<file:///C:/Users/User/Downloads/v15n4a03%20(3).pdf>. Acesso em: 13 de out. 2019.

MARTINS, C.; LIMA, S.M. Vantagens e desvantagens do prontuário eletrônico para instituição de saúde. **Rev. Admin. Saúde**, São Paulo, v. 16, n. 63, p. 61-66, 2014.

Disponível em: <https://pesquisa.bvsalud.org/sms/resource/pt/lil-771428>. Acesso em: 30 de jun. 2018.

MARTINS F.Z.; DALL'AGNOL C.M. Centro cirúrgico: desafios e estratégias do enfermeiro nas atividades gerenciais. **Rev. Gaúcha Enferm.[online]**, v. 37, n. 4, e56945, 2016. Disponível em:<http://www.seer.ufrgs.br/index.php/RevistaGauchadeEnfermagem/article/view/56945/40368>. Acesso 30 jun. 2018.

MASSAROLI, A.; MARTINI, J.G.; LINO, M.M. et al. Método Delphi como referencial metodológico para a pesquisa em enfermagem. **Texto Contexto - Enferm.**, v. 26, n. 4, e1110017, 2017. Disponível em:<http://dx.doi.org/10.1590/0104070720170011110017>. Acesso em: 08 de jan. 2019.

MATA, L. R.F.; CARVALHO, E. C. C.; NAPOLEÃO, A. A. Validação por peritos de intervenções de enfermagem para a alta de pacientes submetidos à Prostatectomia. **Texto contexto enfermagem**, v. 20 p. 36-44, 2011. Disponível em:<http://www.scielo.br/pdf/tce/v20nspe/v20nspea04.pdf>. Acesso em: 20 de set. 2018.

MATIELLO, R.D.C.; LIMA, E.F.A.; COELHO, M.C.R. et al. Patient safety culture from the perspective of nurses. **Cogitare enferm.**, v. 21, n. 5, não p., 2016. Disponível em:<http://revistas.ufpr.br/cogitare/article/view/45408>. Acesso em: 15 de maio 2018.

MATOS, J.C.; RODRIGUES, M.C.S.; BORGES, M.S. et al. Safety culture of the patient in healthe: reflexive analysis. **Rev enferm UFPEon line**., v. 10, n. 6, p. 2223-9, 2016. Disponível em:<http://www.revista.ufpe.br/revistaenfermagem/index.php/revista/article/view/7730/pdf_10434>. Acesso em: 20 de nov. 2018.

MATTHIENSEN, A. Uso do Coeficiente Alfa de Cronbach em Avaliações por Questionários. 1.ed. **Embrapa Roraima**, 2011. Disponível em:<http://ainfo.cnptia.embrapa.br/digital/bitstream/item/68073/1/DOC-48-2011-ID-112.pdf>. Acesso em: 04 de jun. 2018.

MAYER, E.K.; SEVDALIS, N.; ROUT, S. et al. Surgical checklist implementation project: the impact of variable WHO checklist compliance on risk-adjusted clinical outcomes after national implementation: a longitudinal study. **Ann Surg**.; v. 263, n. 1, p. 58-63, 2016. Disponível em:<https://spiral.imperial.ac.uk:8443/handle/10044/1/22141>. Acesso em: 16 de ago. 2019.

MCINTYRE, L.K.; ARBABI, S.; ROBINSON, E.F. et al. Análise de fatores de risco para readmissão do paciente 30 dias após a alta da cirurgia geral. **JAMA Surg.,** v. 151, n. 9, p. 855-861, 2016. Disponível em:<https://jamanetwork.com/journals/jamasurgery/fullarticle/2528953>. Acesso em: 02 de out. 2019.

MEDEIROS, A. C.; ARAÚJO FILHO, I. Centro cirúrgico e cirurgia segura. **J Surg Cl Res**, v. 8, n. 1, p. 77-105, 2017. Disponível em:<https://doi.org/10.20398/jscr.v8i1.13037>. Acesso em: 8 de out. 2019.

MEERSCH, M.; SCHMIDT, C.; ZARBOCK, A. Perioperative Acute Kidney InjuryAn Under Recognized Problem. **Anesth Analg**., v. 125, n. 4, p. 1223-1232, 2017. Disponível em:<https://www.ncbi.nlm.nih.gov/pubmed/28787339>. Acesso em: 02 de set. 2019.

MEHTA, H.B.; DIMOU, F.; ADHIKARI, D. et al. Comparison of Comorbidity Scores in Predicting Surgical Outcomes. **Med Care**., v. 54, n. 2, p. 180–187, 2016. Disponível em:<https://www.ncbi.nlm.nih.gov/pmc/articles/PMC4713334/#!po=2.08333>. Acesso em: 14 de set. 2019.

MEKONNEN, A.B.; MCLACHLAN, A.J.; BRIEN, J.E. et al. Hospital survey on patient safety culture in Ethiopian public hospitals: a cross- sectional study. **Safety in Health**, v. 3, n. 11, não p., 2017. Disponível em:<https://doi.org/10.1186/s40886-017-0062-9>. Acesso em: 12 de ago. 2019.

MENDES, C.M.F.; GUERREIRO, DA S.; BARROSO, F.F.M. Promoting a Culture of Safety in Primary Health Care. **Rev. Port Sau. Pub.**, Lisboa, v. 32, n. 2, p. 197-205, 2014. Disponível em:<https://www.elsevier.es/en-revista-revista-portuguesa-saude-publica-323-articulo-promoting-culture-safety-in-primary-health-care-S0870902514000509>. Acesso em: 25 de set. 2019.

MENDES JUNIOR, W.V. **Avaliação da ocorrência de eventos adversos em hospital no Brasil**.112f. Tese, (Doutorado em Ciências de Saúde Pública) -Escola Nacional de Saúde Pública Sergio Arouca. Rio de Janeiro, 2007. Disponível: <http://bvssp.icict.fiocruz.br/lildbi/docsonline/get.php?id=1080>.Acesso em: 04 de fev. 2018.

MENDES, W.; MARTINS, M.; ROZENFELD, S. et al. The assessment of adverse events in hospitals in Brazil., **Int J Qual Health Care**, v. 21, n. 4, p. 279–284, 2009. Disponívem em:<https://doi.org/10.1093/intqhc/mzp022>. Acesso em: 04 de jun. 2018.

MENDES, W.; PAVÃO, A.L.B.; MARTINS, M. et al. Características de eventos

adversos evitáveis em hospitais do Rio de Janeiro**. Revista da Associação Médica Brasileira**, v. 59, n. 5, p. 421-428, 2013. Disponível em:<http://www.scielo.br/scielo.php?pid=S0104-2302013000500006&script=sci_arttext>. Acesso em: 17 de maio 2019.

MENDES, W.; TRAVASSOS, C.; MARTINS, M. et al. Adaptação dos instrumentos de avaliação de eventos adversos para uso em hospitais brasileiros. **Rev. Bras. Epidemiol**, v. 11, n. 1, p. 55-66, 2008. Disponível em:<http://www.scielo.br/scielo.php?script=sci_arttext&pid=S1415-790X2008000100005>. Acesso em: 04 de jun. 2018.

MENEGUIN, S.; AYRES, J.A.; BUENO, G.H. Caracterização das quedas de pacientes em hospital especializado em cardiologia. **Rev Enferm UFSM**, v. 4, n. 4, p. 784-91, 2014. Disponível em:<https://periodicos.ufsm.br/reufsm/article/view/13554/pdf>. Acesso em: 04 de set. 2019.

MINISTERIO DE SANIDAD, SERVICIOS SOCIALES E IGUALDAD. **Estrategia de Seguridad del Paciente del Sistema Nacional de Salud. -**Seguridad del paciente-2015. Disponível em:<http://www.seguridaddelpaciente.es/resources/documentos/2015/Estrategia%20Seguridad%20del%20Paciente%202015-2020.pdf>. Acesso em: 15 de set. 2019.

MINUZZI, A.P.; SALUM, N.C.; LOCKS, M.O.H. Avaliação da cultura de segurança do paciente em terapia intensiva na perspectiva da equipe de saúde. **Texto Contexto - Enferm**., Florianópolis, v. 25, n. 2, e1610015, p. 1-9, 2016. Disponível em:<http://www.scielo.br/scielo.php?script=sci_arttext&pid=S010407072016000200313&lng=en&nrm=iso>. Acesso em: 24 de maio 2019.

MISKOVIC, A.; LUMB, A.B. Postoperative pulmonary complications. **BJA: British Journal of Anesthesia**, v. 118, n. 3, p. 317–334, 2017. Disponível em:<https://www.ncbi.nlm.nih.gov/pmc/articles/PMC4613407/>. Acesso em: 15 de out. 2019.

MOHAMMAD, R.N. A novel stitch for closure of wounds having dead space to eliminate the risk of hematoma and seroma formation. **Journal of the American Academy of Dermatology**, v. 0, n. 0, não p., 2019. Disponível em:<https://www.jaad.org/article/S0190-9622(18)33000-7/fulltext>. Acesso em: 25 de out. 2019.

MOKKINK, L.B.; PRINSEN, C.A.C.; BOUTER, L.M. et al. The COnsensus-based Standards for the selection of health Measurement Instruments (COSMIN) and how to select an outcome measurement instrument. **Braz J Phys Ther**. v. 20, n. 2, p. 105-

13, 2016. Disponível em:<https://www.ncbi.nlm.nih.gov/pubmed/26786084>. Acesso em: 10 de out. 2019.

MOKKINK, L.B.; TERWEE, C.B.; PATRICK, D.L. et al. **COSMIN checklist manual**. Amsterdam: COSMIN; 2012. Disponível em:<http://www.cosmin.nl/images/upload/files/COSMIN%20checklist%20manual%20v9.pdf.>. Acesso em: 10 de out. 2019.

MORELLO, R.T.; LOWTHIAN, J.A.; BARKER, A.L. et al. Strategies for improving patient safety culture in hospitals: a systematic review. **BMJ Qual Saf**, v. 22, n. 1, p. 11-8, 2013. Disponível em:<https://www.ncbi.nlm.nih.gov/pubmed/22849965>. Acesso em: 10 de set. 2019.

MOTA, D.M.; VIGO, A.; KUCHENBECKER, R.S. Reações adversas a medicamentos no sistema de farmacovigilância do Brasil, 2008 a 2013: estudo descritivo. **Cad. Saúde Pública**, Rio de Janeiro, v. 35, n. 8, e00148818, 2019. Disponível em:<http://www.scielo.br/scielo.php?script=sci_arttext&pid=S0102-311X2019001005005&lng=en&nrm=iso>. Acesso em: 10 de out. 2019.

MOTA, É.C.; OLIVEIRA, A.C. Infecção do trato urinário associada a cateter vesical: por que não controlamos esse evento adverso? **Rev. Esc. Enferm. USP**, São Paulo, v. 53, e03452, 2019. Disponível em:<http://dx.doi.org/10.1590/s1980220x2018007503452>. Acesso em: 20 de out. 2019.

MOURA, M.L.O.; MENDES, W. Avaliação de eventos adversos cirúrgicos em hospitais do Rio de Janeiro. **Rev Bras Epidemiol**, v. 15, n. 3, p. 523-35, 2012. Disponível em:<http://www.scielosp.org/pdf/rbepid/v15n3/07.pdf>. Acesso em: 05 out. 2017.

MULL, H.J.; BORZECKI, A.M.; CHEN, Q. et al. The Human Factors Analysis Classification System (HFACS) Applied to Health Care. **American Journal of Medical Quality**, v. 29, n. 3, p. 181-190, 2014. Disponível em:<https://www.ncbi.nlm.nih.gov/pmc/articles/PMC4562225/>. Acesso em: 15 de out. 2019.

MULL, H.J.; GRAHAM, L.A.; MORRIS, M.S. et al. Association of postoperative readmissions with surgical quality using a Delphi consensus process to identify relevant diagnosis codes. ***JAMA Surg***., v. 153, n. 8, p. 728-737, 2018. Disponível em:<https://www.ncbi.nlm.nih.gov/pubmed/29710234>. Acesso: 19 de out. 2019.
MUSHTAQ, F.; O'DRISCOLL, C.; SMITH, F.C.T. et alContributory factors in surgical incidents as delineated by a confidential reporting system. **Ann R Coll Surg Engl**., v. 100, n. 5, p. 401-5, 2018. Disponível em:<https://doi.org/10.1308/rcsann.2018.0025>.

Acesso em: 10 de ago. 2019.

NAJJAR, S.; BAILLIEN, E.; VANHAECHT, K. et al. Similarities and differences in the associations between patient safety culture dimensions and self-reported outcomes in two different cultural settings: a national cross-sectional study in Palestinian and Belgian hospitals. **BMJ Open**, v. 8, n. 7, e021504, 2018. Disponível em:<http://dx.doi.org/10.1136/bmjopen-2018-021504>. Acesso em: 15 de ago. 2019.

NAJJAR, S.; NAFOURI, N.; VANHAECHT, K. et al. The relationship between patient safety culture and adverse events: a study in palestinian hospitals. **Safety in Health**, v. 1, n. 1, p. 16, 2015. Disponível em:<https://safetyinhealth.biomedcentral.com/articles/10.1186/s40886-015-0008-z>. Acesso em: 06 de jul. 2018.

NANDAN, A.R.; BOHNEN, J.D.; CHANG, D.C. et al. The impact of major intraoperative adverse events on hospital readmissions. **Am J Surg**., v. 213, n. 1, p. 10-7, 2017. Disponível em:<https://www.sciencedirect.com/science/article/abs/pii/S0002961016302926>. Acesso em: 15 de out. 2019.

NATIONAL QUALITY FORUM (NQF). **Safe Practices for Better Healthcare**. 2010. Disponível em:<http://www.qualityforum.org/publications/2010/04/safe_practices_for_better_healthcare_%E2%80%93_2010_update.aspx>. Acesso em: 20 de ago 2018.

NEUHAUSER, D. Florence Nightingale gets no respect: as a statistician that is. **Qual Saf Health Care**., v. 12, n. 4, p. 317, 2003. Disponível em:<https://www.ncbi.nlm.nih.gov/pmc/articles/PMC1743730/>. Acesso em: 02 set. 2019.

NIEVA, V.F.; SORRA, J. Safety culture assessment: a tool for improving patient safety in healthcare organizations. **Qual Saf Heal Care**, v. 12, n. 2, p. 17–23, 2003. Disponível em:<https://www.ncbi.nlm.nih.gov/pmc/articles/PMC1765782/>. Acesso em: 12 de jan. 2019.

NILSSON, L.; RISBERG, M.B.; MONTGOMERY, A. et al. Preventable Adverse Events in Surgical Care in Sweden: A Nationwide Review of Patient Notes. **Medicine,** v. 95, n. 11, e3047, 2016. Disponível em:<https://doi.org/10.1097/MD.0000000000003047>. Acesso em: 12 de set. 2019.

NOORD, I.; WAGNER, C.; DYCK, C. et al. Is culture associated with patient safety in the emergency department? A study of staff perspectives. **International Journal for**

Quality in Health Care, v. 26, n. 1, p. 64-70, 2014. Disponível em:<https://academic.oup.com/intqhc/article/26/1/64/2849752>. Acesso em: 24 de ago. 2019.

NORDÉN-HÄGG, A.; SEXTON, J.B.; KÄLVEMARK-SPORRONG, S. et al. Assessing Safety Culture in Pharmacies: The psychometric validation of the Safety Attitudes Questionnaire (SAQ) in a national sample of community pharmacies in Sweden. **BMC Clinical Pharmacology**, v. 10, n. 8, não p., 2010. Disponível em:<http://www.ncbi.nlm.nih.gov/pmc/articles/PMC2868807/>. Acesso em: 20 de jul. 2018.

NOVARETTI, M.C.Z.; SANTOS, E.V.; QUITÉRIO, L.M. et al. Sobrecarga de trabalho da Enfermagem e incidentes e eventos adversos em pacientes internados em UTI. **Rev. Bras. Enferm**., Brasília, v. 67, n. 5, p. 692-699, 2014. Disponível em:<http://www.scielo.br/scielo.php?script=sci_arttext&pid=S0034-71672014000500692&lng=en&nrm=iso>. Acesso em: 12 de set. 2019.

NATIONAL PATIENT SAFETY FOUNDATION (NPSF). **Free from Harm: accelerating patient safety improvement fifteen years after to err is human**. Boston: NPSF, 2015. Disponível em:<http://www.npsf.org/?page=freefromharm#form>. Acesso em: 10 de jun. 2019.

OLIVEIRA, R. M.; LEITÃO, I.M.T.A.; SILVA, L.M.S. et al. Estratégias para promover segurança do paciente: da identificação dos riscos às práticas baseadas em evidências. **Esc. Anna Nery**, Rio de Janeiro, v. 18, n. 1, p. 122-129, 2014. Disponível em:<http://www.scielo.br/scielo.php?script=sci_arttext&pid=S1414-81452014000100122>. Acesso em: 12 de mai. 2019.

ORGANIZAÇÃO MUNDIAL DA SAÚDE(OMS). **Estrutura Conceitual da Classificação Internacional sobre Segurança do Doente**. Lisboa: OMS, 2011. Divisão de Segurança do Doente-2011.

ORGANIZAÇÃO MUNDIAL DA SAÚDE (OMS). **Segundo desafio global para a segurança do paciente: Cirurgias Seguras Salvam Vidas (Orientações para cirurgia segura da OMS)**. Rio de Janeiro: ALIANÇA MUNDIAL PARA A SEGURANÇA DO PACIENTE, 2009. Disponível em:<http://bvsms.saude.gov.br/bvs/publicacoes/seguranca_paciente_cirurgias_seguras_salvam_vidas.pdf>. Acesso em: 12 de maio de 2019.

ORGANIZAÇÃO PAM-AMERICANA DA SAÚDE (OPAS). **Good Pharmacovigilance Practices for the Americas**-Washington, D.C- Rede PAHRF Documento Técnico Nº5, 2011. Disponível

em:<https://apps.who.int/medicinedocs/documents/s18625pt/s18625pt.pdf>. Acesso 04 de mar. 2018.

PAESE, F.; SASSO, G.T.M.D. Patient safety culture in primary health care. **Texto Contexto - Enferm.**, v. 22, n. 2, p. 302-10, 2013. Disponível em:<http://dx.doi.org/10.1590/S0104-07072013000200005>. Acesso em: 04 de mar. 2019.

PANCIERI, A.P.; SANTOS, B.P.; ÁVILA, M.A.G. et al. Safe surgery checklist: analysis of the safety and communication of teams from a teaching hospital. **Rev Gaúcha Enferm.**, v. 34, n. 1, p. 71-8, 2013. Disponível em:<https://doi.org/10.1590/S1983-14472013000100009>. Acesso em: 12 de jul. 2019.

PARANAGUÁ, T.T.B.; BEZERRA, A.L.Q.; MOREIRA, I.A. et al. Indicadores de asistencia en una clínica quirúrgica. **Enfermería Global**, v. 15, n. 3, p. 228-260, 2016. Disponível em:<https://revistas.um.es/eglobal/article/view/219751>. Acesso em: 07 de set. 2019.

PARANAGUÁ, T.T.B.; BEZERRA, A.L.Q.; SANTOS, A.L.M. et al. Prevalence and factors associated with incidents related to medication in surgical patients. **Revista da Escola de Enfermagem da USP**, São Paulo, v. 48, n. 1, p. 41-47, 2014. Disponível em:<http://www.scielo.br/scielo.php?script=sci_arttext&pid=S0080-62342014000100041&lng=en&nrm=iso>. Acesso: 12 de out. 2019.

PARANAGUÁ, T.T.B.; BEZERRA, A.L.Q.; SILVA, A.E.B.C.; et al. Prevalência de incidentes sem danos e eventos adversos em uma clínica cirúrgica. **Acta Paul. Enferm**, v. 26, n. 3, p. 256-262, 2013. Disponível em:<http://dx.doi.org/10.1590/S0103-21002013000300009>. Acesso em: 22 de nov. 2018.

PARREIRA, J.; PONTES, F.; ANTONUCCI, A. et al. Pneumoencéfalo Espontâneo Hipertensivo Secundário a Osteoma de Seio Frontal. **JBNC - Jornal Bras. de Neurocir**, v. 22, n. 2, p. 48-51, 2018. Disponível em:<http://jbnc.emnuvens.com.br/jbnc/article/view/963>. Acesso em: 22 de out. 2019.

PASQUALI, L. Psicometria. **Revista da Escola de Enfermagem da USP**, São Paulo, v. 43, p. 992-999, 2009. Disponível em:<http://www.scielo.br/scielo.php?script=sci_arttext&pid=S0080-62342009000500002&lng=en&nrm=iso>. Acesso em: 10 de fev. 2019.

PEDREIRA, M.L.G. **Enfermagem para segurança do paciente**. In: Pedreira, L.G.;

Harada, M.J.C.S. Enfermagem dia a dia: segurança do paciente. São Caetano do Sul: Yendis. 2009a. p. 23-31.

PEDREIRA, M.L.G. Práticas de enfermagem baseadas em evidências para promover a segurança do paciente. **Acta Paul. Enferm.,** São Paulo, v. 22, n. esp1, p. 880-881, 2009b. Disponível em:<http://www.repositorio.unifesp.br/bitstream/handle/11600/4821/S0103-21002009000700007.pdf?sequence=1>. Acesso em: 30 de maio 2018.

PEREIRA, B.J.; BADAOUI, M.M.; SOEIRO, E.M.D.S. et al. Lesão renal aguda no pós-operatório de cirurgias não cardíacas em pacientes com recuperação na unidade de terapia intensiva. **Rev Soc Bras Clin Med**., v. 14, n. 4, p. 190-4, 2016. Disponível em:<https://pesquisa.bvsalud.org/portal/resource/pt/biblio-827211>. Acesso em: 11 de out. 2019.

PEREIRA, B.M.T.; PEREIRA, A.M.T.; CORREIA, C.S. et al. Interrupções e distrações na sala de cirurgia do trauma: entendendo a ameaça do erro humano. **Rev. Col. Bras. Cir**., v. 38, n. 5, p. 292-8, 2011. Disponível em:<http://www.scielo.br/scielo.php?script=sci_arttext&pid=S0100-69912011000500002>. Acesso em: 02 de maio 2018.

PEREIRA, H.O.; REZENDE, E.M.; COUTO, B.R.G.M. et al. Tempo de internação pré-operatório: um fator de risco para reduzir a infecção cirúrgica em fraturas de fêmur. **Rev. Bras. Ortop.**, São Paulo, v. 50, n. 6, p. 638-646, 2015. Disponível em:<http://dx.doi.org/10.1016/j.rboe.2015.09.006>. Acesso em: 19 de set. 2019.

PEREIRA, G.N.; ABREU, R.N.D.C.; BONFIM, I.M. et al. Relação entre sistematização da assistência de enfermagem e segurança do paciente. **Enfermagem em Foco**, v. 8, n. 2, jul. 2017. Disponível em:<http://revista.cofen.gov.br/index.php/enfermagem/article/view/985>. Acesso em: 09 out. 2019.

PEREIRA, M. G. **Epidemiologia: teoria e prática**. 12. ed. Rio de Janeiro: Guanabara Koogan, 2008.

PEREIRA, S.K.; SANTANA, R.F.; SANTOS, I. dos. et al. Análise do diagnóstico de enfermagem: recuperação cirúrgica retardada em adultos e idosos hospitalizados. **Rev Min Enferm**., v. 18, n. 3, p. 660-66, 2014. Disponível em:<http://www.dx.doi.org/10.5935/1415-2762.20140048>. Acesso em: 14 de set. 2019.

PÉRISSÉ, L.G.S.; PÉRISSÉ, P.C.M.; BERNARDO JÚNIOR, C. Tratamento endoscópico das fístulas após gastrectomia vertical e by-pass gástrico em Y de Roux. **Rev Col Bras Cir**. v. 42, n. 3, p. 159-64, 2015. Disponível em:<10.1590/0100-69912015003006>. Acesso em: 06 de out. 2018.

PERROCA, M.G. Development and content validity of the new version of a patient classification instrument. **Rev. Latino-Am. Enfermagem.**, v. 19, n. 1, p. 58-66, 2011. Disponível em:<https://doi.org/10.1590/S0104-11692011000100009>. Acesso em: 2 de maio 2019.

PETERLINI, F.L.; SAES, A.C. **Culpa: cultura de não punição e accountability na assistência à saúde.** In: Fonseca, A.S.; Peterlini, F.L.; Costa, D.A.; Segurança do paciente. São Paulo: Martinari, 2014.

PIERDEVARA, L.; VENTURA, I.M.; EIRAS, M. et al. An experience with the Global Trigger Tool for the study of adverse events in a medical ward. **Rev. Enf. Ref.**, Coimbra, v. serIV, n. 9, p. 97-105, 2016. Disponível em:<http://www.scielo.mec.pt/scielo.php?script=sci_arttext&pid=S0874-02832016000200011&lng=pt&nrm=iso>. Acesso em: 17 de out. 2019.

PIERDEVARA, L.; VENTURA, I.M.; EIRAS, M.B. et al. Trigger Tool na Segurança do Doente: Uma Revisão Sistemática de Literatura. **Port J Public Health,** v. 35, n. 2, p. 69-76, 2017. Disponível em:<https://doi.org/10.1159/000479606>. Acesso em: 19 de jan. 2019.

PINHEIRO, M.P.; SILVA JUNIOR, O.C. Avaliação da cultura de segurança do paciente na organização hospitalar de um hospital universitário. **Enfermería Global,** v. 45, n. 45, p. 325-338, 2017. Disponível em:<http://scielo.isciii.es/pdf/eg/v16n45/pt_1695-6141-eg-16-45-00309.pdf>. Acesso em: 10 de ago. 2019.

POLIT, D.F. Assessing measurement in health: beyond reliability and validity. **Int J Nurs Stud.,** v. 52, n. 11, p. 1746-1753, 2015. Disponível em:<https://doi.org/10.1016/j.ijnurstu.2015.07.002>. Acesso em: 10 de nov. 2018.

POLIT, D.F.; BECK, C.T.; HUNGLER, B.P. **Fundamentos de pesquisa em enfermagem: métodos, avaliação e utilização**. 5 ed. Porto Alegre: Artmed, 2004.

POLIT, D.F; BECK, C.T. **Fundamentos de pesquisa em enfermagem: avaliação de evidências para a prática de enfermagem**. 7. ed. Porto Alegre, Editora Artmed, p. 669, 2011.

PONTES, S.R.S.; SALAZAR, R.M.; TORRES, O.J.M. Avaliação perioperatória de pacientes em unidade de terapia intensiva. **Rev. Col. Bras. Cir.**, Rio de Janeiro, v. 40, n. 2, p. 92-97, 2013. Disponível em:<http://www.scielo.br/scielo.php?script=sci_arttext&pid=S0100-69912013000200002&lng=en&nrm=iso>. Acesso em: 12 de out. 2019.

PRATES, C.G.; LUZIA, M.F.; ORTOLAN, M.R. et al. Falls in hospitalized adults: incidence and characteristics of these events. **Cienc Cuid Saúde**, v. 13, n. 1, p. 74-81, 2014. Disponível em:<http://periodicos.uem.br/ojs/index.php/CiencCuidSaude/article/viewFile/20728/pdf_145>. Acesso em: 17 de out. 2019.

PUCHER, P.H.; JOHNSTON, M.J.; AGGARWAL, R. et al. Effectiveness of interventions to improve patient handover in surgery: a systematic review. **Surgery**, v. 158, n. 1, p. 85-95, 2015. Disponível em:<http://dx.doi.org/10.1016/j.surg.2015.02.017>. Acesso em: 12 de ago. 2019.

QUINTANO NEIRA, R.A.; HAMACHER, S.; JAPIASSÚ, A.M. Epidemiology of sepsis in Brazil: Incidence, lethality, costs, and other indicators for Brazilian Unified Health System hospitalizations from 2006 to 2015. **PLoS One**, v. 13, n. 4, e0195873, 2018. Disponível em:<https://doi.org/10.1371/journal.pone.0195873>. Acesso em: 22 de out. 2019.

RABELO, R.A.; FERENHOF, H.A.; RADOS, G.V. et al. Gestão do Conhecimento em processos de transformação organizacional: o desenvolvimento da intimidade como fator facilitador. **Perspectivas em Gestão & Conhecimento**, João Pessoa, v. 2, n. 1, p. 21-35, 2012. Disponível em:<https://periodicos.ufpb.br/ojs2/index.php/pgc/article/view/12545>. Acesso em: 11 set. 2019.

RAEISSI, P.; REISI, N.; NASIRIPOUR, A.A. Assessment of patient safety culture in Iranian academic hospitals: strengths and weaknesses. **J Patient Saf**., v. 14, n. 4, p. 213-226, 2018. Disponível em:<https://doi: 10.1097 / PTS.0000000000000199>. Acesso em: 15 de set. 2019.

RAFTER, N.; HICKEY, A.; CONROY, R.M. et al. The Irish National Adverse Events Study (INAES): the frequency and nature of adverse events in Irish hospitals - a retrospective record review study. **BMJ Qual Saf**, v. 26, n. 2, p. 111-19, 2017. Disponível em:<http://dx.doi.org/10.1136/bmjqs-2015-004828>. Acesso em: 10 out. 2019.

RAMOS, F.; COCA, S.M.; ABELDANO, R.A. Percepción de la cultura de seguridad de pacientes en profesionales de una institución argentina. **Enferm. univ**, México, v.

14, n. 1, p. 47-53, 2017. Disponível em:<http://www.scielo.org.mx/scielo.php?script=sci_arttext&pid=S1665-70632017000100047&lng=es&nrm=iso>. Acesso em: 10 de set. 2019.

REASON, J. Human error: models and management. **BMJ**, v. 320, n. 7237, p. 768-70, 2000. Disponível em:<https://www.ncbi.nlm.nih.gov/pmc/articles/PMC1117770/>. Acesso em: 12 de jan. 2019.

REDE BRASILEIRA DE ENFERMAGEM E SEGURANÇA DO PACIENTE (REBRAENSP). **Estratégias para segurança do paciente: manual para profissionais da saúde.** -Porto Alegre-Rede Brasileira de Enfermagem e Segurança do Paciente, 2013.

REED, S.; GANYANI, R.; KING, R. et al. Does a novel method of delivering the safe surgical checklist improve compliance? A closed loop audit. **Int J Surg**., v. 32, n. x, p. 99-108, 2016. Disponível em:<http://dx.doi.org/10.1016/j.ijsu.2016.06.035>. Acesso em: 12 ago. 2019.> Acesso em: 03 de out. 2019.

REIS, C.T. **A cultura de segurança do paciente: validação de um instrumento de mensuração para o contexto hospitalar brasileiro.** 203f. Tese (Doutorado em Ciências da Saúde Pública) - Escola Nacional de Saúde Pública Sergio Arouca, Rio de Janeiro, 2013. Disponível em:<http://proqualis.net/sites/proqualis.net/files/000002441PwFnHv.pdf>. Acesso em: 03 de jun. 2017.

REIS, C.T.; LAGUARDI, J.; MARTINS, M. A. Adaptação transcultural da versão brasileira do Hospital Survey on Patient Safety Culture: etapa inicial. **Caderno de Saúde Pública**, Rio de Janeiro, v. 28, n. 11, p. 2199-2210, 2012. Disponível em:<http://www.scielo.br/pdf/csp/v28n11/19.pdf>. Acesso em: 03 de jun. 2017.

REIS, C.T.; LAGUARDIA, J.; VASCONCELOS, A.G.G.; MARTINS M. Reliability and validity of the Brazilian version of the Hospital Survey on Patient Safety Culture (HSOPSC): a pilot study. **Cad. Saúde Pública**, Rio de Janeiro, v. 32, n. 11, e00115614, 2016. Disponível em:<http://dx.doi.org/10.1590/0102-311x00115614>. Acesso em: 06 de nov. 2018.

REIS, C.T.; MARTINS, M.; LAGUARDIA, J. A segurança do paciente como dimensão da qualidade do cuidado de saúde: um olhar sobre a literatura. **Ciênc. saúde coletiva**, Rio de Janeiro, v. 18, n. 7, p. 2029-2036, 2013a. Disponível em:<http://www.scielo.br/scielo.php?script=sci_arttext&pid=S1413-81232013000700018&lng=en&nrm=iso>. Acesso em: 04 de jun. 2017.

REIS, C.T.; PAIVA, S.G.; SOUSA, P. The patient safety culture: a systematic review

by characteristics of Hospital Survey on Patient Safety Culture dimensions. **Int J Qual Health Care**, Oxford, v. 30, n. 9, p. 660-677, 2018. Disponível em:<https://doi.org/10.1093/intqhc/mzy080>. Acesso em: 05 de jun. 2019.

REIS, G.A.X.; HAYAKAWA, L.Y.; MURASSAKI, A.C.Y. et al. Implantação das estratégias de segurança do paciente: percepções de enfermeiros gestores. **Texto Contexto-enferm**., Florianópolis, v. 26, n. 2, p. e00340016, 2017. Disponível em:<http://www.scielo.br/scielo.php?script=sci_arttext&pid=S010407072017000200321&lng=en&nrm=iso>. Acesso em: 23 de set. 2019.

REVORÊDO, L.S.; DANTAS, M.M.C.; MAIA, R.S. et al. Validação de conteúdo de um instrumento para identificação de violência contra criança. **Acta Paul. Enferm.**, São Paulo, v. 29, n. 2, p. 205-217, 2016. Disponível em:<http://dx.doi.org/10.1590/1982-0194201600029>. Acesso em: 20 de jan. 2019.

RHODES, A.; PHILLIPS, G.; BEALE. R. et al. The surviving Sepsis campaign bundles and outcome: results from the international multicentre prevalence study on Sepsis (the IMPreSS study). **Intensive Care Med Springer Berlin Heidelberg**, v. 41, n. 9, p. 1620–8, 2015. Disponível em:<https://doi.org/10.1007/s00134-015-3906-y>. Acesso em: 10 de out. 2019.

RIBEIRO, G.S.R.; SILVA, R.C.; FERREIRA, M.A. et al. Equipment failure: conducts of nurses and implications for patient safety. **Rev. Bras Enferm** [Internet]. v. 71, n. 4, p. 1832-40, 2018. Disponível em:<http://dx.doi.org/10.1590/0034-7167-2016-0547>. Acesso em: 12 de out. 2019.

RIBEIRO, W.A.; MATTOS, I.F.; MORAIS, M.C. et al. Cirurgia segura: a enfermagem protagonizando a segurança do paciente no Centro Cirúrgico. **Revista Pró-univerSUS**, v. 10, n. 1, p. 66-71, 2019. Disponível em:<https://doi.org/10.21727/rpu.v10i1.1684>. Acesso em: 27 de out. 2019.

RIGOBELLO, M.C.G.; CARVALHO, R.E.F.L.; CASSIANI, S.H.B. et al. Clima de segurança do paciente: percepção dos profissionais de enfermagem. **Acta Paul. Enferm**., v. 25, n. 5, p. 728-735, 2012. Disponível em:<http://www.scielo.br/scielo.php?script=sci_arttext&pid=S0103-21002012000500013>. Acesso em: 10 de jul. 2019.

ROBBINS, S.P.; JUDGE, T.A.; SOBRAL, F. **Comportamento Organizacional**. 14ª ed. São Paulo: Pearson Prentice Hall, 2010.

ROBIDA, A. Hospital Survey on Patient Safety Culture in Slovenia: a psychometric evaluation. **Int J Qual Health Care**, Oxford, v. 25, n. 4, p. 469–75, 2013. Disponível

em:<https://www.ncbi.nlm.nih.gov/pubmed/23736832>. Acesso em: 05 de jul. 2019.

ROCROCKVILLE, W; SORRA, J; GRAY, L. et al. **AHRQ Hospital Survey on Patient Safety Culture: User's Guide**. Rockville, MD: Agency for Healthcare Research and Quality, 2016. Disponível em:<https://www.ahrq.gov/sites/default/files/wysiwyg/professionals/quality-patient-safety/patientsafetyculture/hospital/userguide/hospcult.pdf>. Acesso em: 29 de maio 2018.

RODGERS, A.; WALKER, N.; SCHUG, S. et al. Reduction of postoperative mortality and morbidity with epidural or spinal anaesthesia: results from overview of randomised trials. **BMJ**., v. 321, p. 1493-1497, 2000. Disponível em:<https://www.bmj.com/content/321/7275/1493>. Acesso em: 17 de out. 2019.

ROQUE, K.E.; TONINI, T.; MELO, E.C.P. Adverse events in the intensive care unit: impact on mortality and length of stay in a prospective study. **Cad. Saúde Pública**, Rio de Janeiro, v. 32, n. 10, e00081815, 2016. Disponível em:<http://www.scielo.br/scielo.php?script=sci_arttext&pid=S0102-311X2016001005001&lng=en&nrm=iso>. Acesso em: 15 de set. 2019.

ROSCANI, A.N.C.P.; FERRAZ, E.M.; OLIVEIRA FILHO, A.G. et al. Validação de checklist cirúrgico para prevenção de infecção de sítio cirúrgico. **Acta Paul. Enferm**., São Paulo, v. 28, n. 6, p. 553-565, 2015. Disponível em:<http://www.scielo.br/scielo.php?script=sci_arttext&pid=S0103-21002015000600553&lng=en&nrm=iso>. Acesso em: 25 de out. 2019.

RUTBERG, H.; RISBERG, M.B.; SJÖDAH, R. et al. Characterizations of adverse events detected in a university hospital: a 4-year study using the Global Trigger Tool method. **BMJ Open**., v. 4, n. 5, não p., 2014. Disponível em:<http://bmjopen.bmj.com/content/4/5/e004879>. Acesso em: 5 de set. 2019.

ROUQUAYROL, M. Z.; SILVA, M.G.C. **Rouquayrol epidemiologia & saúde**. 7. ed. Rio de Janeiro: MedBook, 2013. 709 p.

SCHARDOSIM, J. M., RUSCHEL, L.M., CUNHA, M.L.C. da. et al. Adaptação transcultural e validação clínica da Neonatal Skin Condition Score para o português do Brasil. **Rev. Latino-Am. Enfermagem**, v. 22, n. 4, p. 834-41, 2014. Disponível em:<http://www.scielo.br/pdf/rlae/v22n5/pt_0104-1169-rlae-22-05-00834.pdf>. Acesso em: 29 de set. 2018.

SALVADOR, P.T.C.O.; COSTA, da T.D.; GOMES A.T.L. et al. Segurança do paciente: caracterização de vídeos do YouTube. **Rev. Gaúcha Enferm**., Porto Alegre, v. 38, n.

1, e61713, 2017. Disponível em:< http://www.scielo.br/pdf/rgenf/v38n1/0102-6933-rgenf-1983-144720170161713.pdf>. Acesso em: 30 de nov. 2018.

SANTANA, H.T.; FREITAS, M.R.; FERRAZ, E.M. et al. WHO Safety Surgical Checklist implementation evaluation in public hospitals in the Brazilian Federal District. **J Infect Public Health**, v. 9, n. 5, p. 586-599, 2016. Disponível em:<https://doi.org/10.1016/j.jiph.2015.12.019>. Acesso em: 12 de ago. 2019.

SANTANA, R.F.; AMARAL, D.M.; PEREIRA, S.K. et al. The occurrence of the delayed surgical recovery nursing diagnosis among adults and the elderly**. Acta Paul. Enferm**., São Paulo, v. 27, n. 1, p. 35-39, 2014. Disponível em:<http://www.scielo.br/scielo.php?script=sci_arttext&pid=S0103-21002014000100008&lng=en&nrm=iso>. Acesso em: 17 de out. 2019.

SANTIAGO, T.H.R.; TURRINI, R.N.T. Cultura e clima organizacional para segurança do paciente em Unidades de Terapia Intensiva. **Revista da Escola de Enfermagem da USP**, São Paulo, v. 49, n. spe, p. 123-130, 2015. Disponível em:<http://www.scielo.br/scielo.php?script=sci_arttext&pid=S0080-62342015000700123&lng=en&nrm=iso>. Acesso em: 03 de Jun. 2018.

SANTOS, G.A.A.C.; BOCCHI, S.C.M. Cancelamento de cirurgias eletivas em hospital público brasileiro: motivos e redução estimada. **Rev Bras Enferm**., Brasília, v. 70, n. 3, p. 535-542, 2017. Disponível em:<http://www.scielo.br/scielo.php?script=sci_arttext&pid=S0034-71672017000300535&lng=en&nrm=iso>. Acesso em: 28 de out. 2019.

SANTOS, G.C.; BAYLÃO, A.F.G.; BORGES, S.C.F. et al. Incidência e fatores de risco de infecção de sítio cirúrgico: revisão integrativa. **Revista Eletrônica Intinerarius Reflectionis**, v. 11, n. 1, 2015. Disponível em:<https://www.revistas.ufg.br/rir/article/view/34142>. Acesso em: 12 de out. 2018.

SANTOS, L.L.B. dos; FERRAZ, C.S.M.; CHAGAS, M.M. Fatores de risco associados ao agravamento de sepse em pacientes em Unidade de Terapia Intensiva. **Cad. Saúde Colet.**, Rio de Janeiro, v. 24, n. 4, p. 388-396, 2016. Disponível em:<http://dx.doi.org/10.1590/1414462x201600040091>. Acesso em: 16 de out. 2019.

SATHIYAKUMAR, V.; THAKORE, R.V.; GREENBERG, S.E. et al. Adverse Events in Orthopaedics: Is Trauma More Risky? An Analysis of the NSQIP Data. **J Orthop Trauma**., v. 29, n. 7, p. 337-41, 2015. Disponível em:<https://www.ncbi.nlm.nih.gov/pubmed/26091531>. Acesso em: 02 de out. 2019.

SCHEIN, E.H. **Organizational culture and leadership**. San Francisco: Jossey-Bass Publishers, 1991.

SCHEIN, E.H. **Organizational culture and leadership**. 4 ed. San Francisco (USA): John Wiley & Sons, 2010. 446p.

SCHMIDT, L.; WIESE, L.P.L.; PEREIRA, E.M. et al. Lesão renal aguda em pacientes críticosperfil clínico e relação com processos infecciosos graves. **Rev Bras Farm Hosp Serv Saúde,** v. 7, n. 3, p. 19-24, 2016. Disponível em:<http://www.sbrafh.org.br/rbfhss/public/artigos/2016070303000956BR.pdf>. Acesso em: 17 de out. 2019.

SCHMIDT, S.M.S.; MÜLLER, F.M.; SANTOS, E. et al. Análise da satisfação dos usuários em um hospital universitário. **Saúde debate**, Rio de Janeiro, v. 38, n. 101, p. 305-317, 2014. Disponível em:<http://dx.doi.org/10.5935/01031104.20140028>. Acesso em: 02 de set.2019.

SCOTT, T.; MANNION, R.; DAVIES, H.T.O. et al. Implementing culture change in health care: theory and practice. **Int J Qual Health Care**, v. 15, n. 2, p. 111-8, 2003. Disponível em:<https://www.ncbi.nlm.nih.gov/pubmed/12705704>. Acesso em: 10 de nov. 2018.

SECANELL, M.; ORREGO, C.; VILA, M. et al. A surgical safety checklist implementation: experience of a start-up phase of a collaborative project in hospitals of Catalonia, **Spain Med.Clin**., v. 143, n. 1, p. 17-24, 2014. Disponível em:<https://doi.org/10.1016/j.medcli.2014.07.007>. Acesso em: 03 maio 2019.

SELL, B.T.; AMANTE, L.N.; MARTINS, T. et al. Dimensionamento dos profissionais de enfermagem e a ocorrência de eventos adversos em internação cirúrgica**. Cienc Cuid Saude,** v. 17, n. 1, 2018. Disponível em:<http://periodicos.uem.br/ojs/index.php/CiencCuidSaude/issue/view/1514>. Acesso em: 5 de out. 2019.

SELL, B.T.; AMANTE, L.N.; MARTINS, T. et al. Eventos adversos em uma unidade de internação cirúrgica: estudo descritivo. **Revista SOBECC**, São Paulo, v. 21, n. 3, p. 146-153, 2016. Disponível em:<https://revista.sobecc.org.br/sobecc/article/view/34>. Acesso em: 22 de maio 2017.

SENA, A.C.; NASCIMENTO, E.R.P.; MAIA, A.R.C.R. Prática de enfermagem do cuidado a pacientes submetidos à cirurgia eletiva no pré-operatório imediato. **Rev. Gaúcha Enferm**. Porto Alegre, v. 34, n. 3, p. 132-137, 2013. Disponível

em:<http://www.scielo.br/scielo.php?script=sci_arttext&pid=S1983-14472013000300017&lng=en&nrm=iso>. Acesso em: 09 de nov. 2019.

SEXTON, J.B.; HELMREICH, R.L.; NEILANDS, T.B. et al. The Safety Attitudes Questionnaire: psychometric properties, benchmarking data, and emerging research. **BMC Heal Serv Res** [Internet]. v. 6, n. 44, não p., 2006. Disponível em:<https://www.ncbi.nlm.nih.gov/pmc/articles/PMC1481614/>. Acesso em: 05 de jun. 2018.

SHIOZAKI, K.; MORIMATSU, H.; MATSUSAKI, T. et al. Observational study to assess and predict serious adverse events after major surgery. **Acta Med Okayama**, v. 70, n. 6, p. 461-467, 2016. Disponível em:<http://dx.doi.org/10.18926/AMO/54809>. Acesso em: 12 de set. 2019.

SHRIME, M.G.; BICKLER, S.W.; ALKIRE, B.C. et al. Global burden of surgical disease: an estimation from the provider perspective. **The Lancet Global Health**, v. 3, n. 2, p. S8-S9, 2015. Disponível em:<https://www.thelancet.com/journals/langlo/article/PIIS2214-109X(14)70384-5/fulltext>. Acesso em: 12 de set. 2019.

SILVA, A.C.A.B.; ROSA, D.O.S. Cultura de Segurança do Paciente em Organização Hospitalar. **Cogitare enferm**. v. 21, n. 5, p. 1-10, 2016. Disponível em:<http://dx.doi.org/10.5380/ce.v21i5.45583>. Acesso em: 10 de set. 2019.

SILVA, A.C.A.; DA SILVA, J.F.; SANTOS, L.R.O. et al. A segurança do paciente em âmbito hospitalar: revisão integrativa da literatura. **Cogitare enferm**, v. 21, n. 5, não p., 2016. Disponível em:<http://revistas.ufpr.br/cogitare/article/view/37763>. Acesso em: 24 de maio 2017.

SILVA, A.E.B.C. Segurança do paciente: desafios para a prática e a investigação em Enfermagem [editorial]. **Rev. Eletr. Enf**., v. 12, n. 3, p. 422, 2010. Disponível:<http://revistas.ufg.br/index.php/fen/article/view/11885/7815>. Acesso em: 24 de maio 2017.

SILVA, A.T.; ALVES, M.G.; SANCHES, R.S. et al. Assistência de enfermagem e o enfoque da segurança do paciente no cenário brasileiro. **Saúde Debate**. Rio de Janeiro, v. 40, n. 111, p. 292-301, 2016. Disponível em:<http://www.scielo.br/pdf/sdeb/v40n111/0103-1104-sdeb-40-111-0292.pdf>. Acesso em: 14 de set. 2019.

SILVA-BATALHA, E.M.S.; MELLEIRO, M.M. Cultura de segurança do paciente em um hospital de ensino: diferenças de percepção existentes nos diferentes cenários dessa instituição. **Texto Contexto - Enferm**, v. 24, n. 2, p. 432-41, 2015. Disponível

em:<http://www.scielo.br/pdf/tce/v24n2/pt_0104-0707-tce- 24-02-00432.pdf>. Acesso em: 7 de ago. 2018.

SILVA, F. G. da; OLIVEIRA JUNIOR, N.J.; OLIVEIRA, D.O. et al. Análise de eventos adversos em um Centro Cirúrgico ambulatorial. **Rev. SOBECC**, São Paulo, v. 20, n. 4, p. 202-209, 2015. Disponível em:<https://revista.sobecc.org.br/sobecc/article/view/91>. Acesso em: 19 de nov. 2018.

SILVA, R.; AMANTE, L.N. Checklist for the intrahospital transport of patients admitted to the intensive care unit. **Texto Contexto - Enferm.** [Internet]. v. 24, n. 2, p. 539-47, 2015. Disponível em:<http://www.scielo.br/pdf/tce/v24n2/0104-0707-tce-24-02-00539.pdf>. Acesso em: 02 de jun. de 2019.

SILVA, T.M.; FARIA, A.P.G.; GUIMARÃES, F.S. et al. Atualizações em Sepse como facilidade de diagnóstico precoce: um relato de caso. **Braz. J. Hea. Rev**., Curitiba, v. 2, n. 5, p. 3950-3956, 2019. Disponível em:<http://www.brazilianjournals.com/index.php/BJHR/article/view/3083>. Acesso em: 16 de out. 2019. Acesso em: 19 de out. 2019.

SINGER, M.; DEUTSCHMAN, C.S.; SEYMOUR C.W. et al. As definições do terceiro consenso internacional para sepse e choque séptico (sepse-3). **JAMA**, v. 315, n. 8, p. 801-810, 2016. Disponível em:< https://jamanetwork.com/journals/jama/article-abstract/2492881>. Acesso em: 20 de set. 2019.

SINGER, S.J.; VOGUS, T.J. Reducing hospital errors: interventions that build safety culture. **Annu Rev Public Health**, v. 34, p. 373-96, 2013. Disponível em:<https://www.ncbi.nlm.nih.gov/pubmed/23330698>. Acesso em: 11 de set. 2019.

SIQUEIRA, L.D.C.; CALIRI, M.H.L.; KALISCH, B. et al. Adaptação cultural e análise da consistência interna do instrumento MISSCARE para uso no Brasil. **Rev. Latino-Am. Enferm.**, v. 21, n. 2, p. 610-617, 2013. Disponível em:<https://doi.org/10.1590/S0104-11692013000200019>. Acesso em: 15 de maio 2017.

SMILEY, K.; OFORI, L.; SPANGLER, C. et al. Safety culture and perioperative quality at the Volta river authority hospital in Akosombo, Ghana. **World j sur**, v. 43, n. 1, p. 16-23, 2019. Disponível em:<https://doi.org/10.1007/s00268-018-4763-y>. Acesso em: 03 de jul. 2019.

SORRA, J.; GRAY, L.; STREAGLE, S. et al. **AHRQ Hospital Survey on Patient Safety Culture: User's Guide**. (Prepared by Westat, under Contract No.

HHSA290201300003C). AHRQ Publication No. 15-0049-EF (Replaces 04-0041). Rockville, MD: Agency for Healthcare Research and Quality, 2016. Disponível em:<https://www.ahrq.gov/sites/default/files/wysiwyg/professionals/quality-patient-safey/patientsafetyculture/hospital/userguide/hospcult.pdf>. Acesso em: 29 de maio 2018.

SORRA, J.S.; NIEVA, V.F. AHRQ Hospital Survey on Patient Safety Culture: User's Guide. **AHRQ**, v. 15, n. 16, p. 4-49, 2004. Disponível em:<https://www.ahrq.gov/sites/default/files/wysiwyg/professionals/quality-patient-safety/patientsafetyculture/hospital/resources/hospcult.pdf>. Acesso em: 06 jun. 2018.

SOUSA, P; MENDES, W. **Conhecendo os riscos nas organizações de saúde**. Rio de Janeiro: Editora Fiocruz, 2014. 452p. ISBN: 978-85-8432-013-4.

SOUZA, A.C. de.; ALEXANDRE, N.M.C.; GUIRARDELLO, E.B. Propriedades psicométricas na avaliação de instrumentos: avaliação da confiabilidade e da validade. **Epidemiol. Serv. Saúde**, v. 26, n. 3, não p, 2017. Disponível em:<https://doi.org/10.5123/S1679-49742017000300022>. Acesso em: 10 de out. 2019.

SOUZA, P.; MENDES, W. **Segurança do Paciente: criando organizações de saúde seguras**. 2ª ed. Rio de Janeiro: Editora Fiocruz, 2019. 208p.

SOUZA V.S. de; KAWAMOTO A.M.; DE OLIVEIRA J.L.C. et al. Erros e eventos adversos: a interface com a cultura de segurança dos profissionais de saúde. **Cogitare enferm.**, v. 20, n. 3, p. 475-482, 2015. Disponível em:<http://dx.doi.org/10.5380/ce.v20i3.40687>. Acesso em: 09 de jun. 2018.

SPANJERSBERG, W.R.; REURINGS, J.; KEUS, F. et al. Fast track surgery versus conventional recovery strategies for colorectal surgery. **Cochrane Database Syst Rev.,** v. 16, n. 2, p. CD007635. Disponível em:<https://www.ncbi.nlm.nih.gov/pubmed/21328298>. Acesso em: 02 de set. 2019.

SPRUCE, L. Back to basics: preventing perioperative pressure injuries. **AORN J**, v. 105, n. 1, p. 92-9, 2017. Disponível em:<https://doi.org/10.1016/j.aorn.2016.10.018>. Acesso em: 07 de ago. 2019.

STOCKWELL, D.C.; BISARYA, H.; CLASSEN, D.C. et al. A trigger tool to detect harm in pediatric inpatient settings. **PEDIATRICS**. [Internet]. v. 135, n. 6, p. 1036-1042, 2015. Disponível em:<https://doi.org/10.1542/peds.2014-2152>. Acesso em: 02 de out. 2019.

STRACIERI, L.D.S. Cuidados e complicações pós-operatórias. **Medicina**. v. 41, n. 4, p. 465-8, 2008. Disponível em:<https://www.revistas.usp.br/rmrp/article/viewFile/288/289>. Acesso em: 16 de out. 2019.

TALESCHIAN-TABRIZI, N.; FARHADI, F.; MADANI, N. et al. Compliance with guideline statements for urethral catheterization in an iranian teaching hospital. **Int J Health Policy Manag**, v. 4, n. 12, p. 805-11, 2015. Disponível em:<https://www.ncbi.nlm.nih.gov/pmc/articles/PMC4663083/>. Acesso em: 10 de out. 2019.

TAVARES, A.P.M.; MOURA, E.C.C.; AVELINO, F.V.S.D. et al. Cultura de segurança do paciente na perspectiva da equipe de enfermagem. **Rev. Rene**, Fortaleza, v. 19, e3152, 2018. Disponível em:<http://periodicos.ufc.br/rene/article/view/31319>. Acesso em: 16 de ago. 2019.

TEIXEIRA, T.C.A.; CASSIANI, S.H.B. Análise de causa raiz de acidentes por quedas e erros de medicação em hospital. **Acta Paul Enferm**, v. 27, n. 2, p. 100-7, 2014. Disponível em:<http://www2.unifesp.br/acta/pdf/v27/n2/v27n2a0.pdf#page=15>. Acesso em: 06 de jun. 2017.

TENKE, P.; MEZEI, T.; BODE, I. et al. Catheter-associated urinary tract infections. **Eur Urol Suppl**., v. 16, n. 4, p. 138-43, 2017. Disponível em:<https://doi.org/10.1016/j.eursup.2016.10.001>. Acesso em: 10 de out. 2019.

TEREANU, C.; SAMPIETRO, G.; SARNATARO, F. et al. Survey on Patient Safety Culture in the Republic of Moldova: a baseline study in three healthcare settings. **Clujul Med**., v. 91, n. 1, p. 65-74, 2018. Disponível em:<https://doi.org/10.15386/cjmed-869>. Acesso em: 07 de jul. 2019.

THE HEALTH FOUNDATION. **Evidence scan. Measuring safety culture**. London: The Health Foundation. 2011. Disponível em:<http://www.health.org.uk/sites/default/files/MeasuringSafetyCulture.pdf>. Acesso em: 21 de maio 2017.

THOMSEN, T.; VILLEBRO, N.; MOLLER, A.M. Interventions for preoperative smoking cessation (review). **Cochrane Database Syst Ver**., v. 27, n. 3, CD002294, 2014. Disponível em:<https://www.ncbi.nlm.nih.gov/pubmed/24671929>. Acesso em: 13 de out. 2019.

TIMM, M.; RODRIGUES, M.C.S. Adaptação transcultural de instrumento de cultura de segurança para a Atenção Primária. **Acta Paul Enferm.**, v. 29, n. 1, p. 26-37, 2016. Disponível

em:<http://www.scielo.br/scielo.php?pid=S0103210020160001000 26&script=sci_abstract&tlng=pt:>. Acesso em: 31 de out. 2018.

THE JOINT COMISSION. **Sentinel Event Data Root Causes by Event Type 2004-2012.** Oakbrook Terrace,IL-The Joint Commission, 2013. Disponível em:<http://www.jointcommission.org/assets/1/18/Root_Causes_Event_Type_04_4Q2012.pdf>. Acesso em: 18 de out. 2018.

TOBIAS, Q.G.C.; BEZERRA, A.L.Q.; BRANQUINHO, N.C.S.; SILVA, A.E.B.C. Cultura de Segurança do paciente em instituições de saúde: um estudo bibliométrico. **Enfermería Global**. [Internet], v. 13, n. 33, p. 349-361, 2014. Disponível em:<http://scielo.isciii.es/pdf/eg/v13n33/pt_revision1.pdf>. Acesso em: 05 de mar. 2019.

TOMASCHEWSKI-BARLEM, J. G., LERCH L.M., DEVOS, E.L et al. Adaptação transcultural e validação do instrumento Protective Nursing Advocacy Scale para enfermeiros brasileiros. **Rev. Latino-Am. Enfermagem**, v. 23, n. 4, p. 669-76, 2015. Disponível em:<http://www.scielo.br/scielo.php?pid=S0104-11692015000400669&script=sci_abstract>. Acesso em: 10 de out. 2018.

TOMAZONI, A.; ROCHA, P.; SOUZA, S. et al. Cultura de segurança do paciente em unidades de terapia intensiva neonatal: perspectivas da equipe de enfermagem e médica. **Rev Latino-Am. Enfermagem**, v. 5, n. 2, p. 755-763, 2014. Disponível em:<https://doi.org/10.1590/01041169.3624.2477>. Acesso em: 05 de maio 2017.

TOMAZONI, A.; ROCHA, P.K.; KUSAHARA, D.M. et al. Avaliação de cultura de segurança do paciente em terapia intensiva neonatal. **Texto Contexto - Enferm**, v. 24, n. 1, p. 161-9, 2015. Disponível em:<http://dx.doi.org/10.1590/0104-07072015000490014>. Acesso em: 08 set. 2019.

TREADWELL, J.R.; LUCAS, S.; TSOU, A.Y. Surgical checklists: a systematic review of impacts and implementation. **BMJQual. Saf.,** v. 23, n. 4, p. 299-318, 2014. Disponível em:<http://dx.doi.org/10.1136/bmjqs-2012-001797>. Acesso em: 6 de out. 2019.

URBACH, D.R.; GOVINDARAJAN, A.; SASKIN, R. et al. Introduction of surgical safety checklists in Ontario, Canada. **N. Engl. J. Med.**, v. 370, p. 1029-38, 2014. Disponível em:<https://www.nejm.org/doi/full/10.1056/nejmsa1308261>. Acesso em: 6 de out. 2019.

URBANETTO, J.S.; GERHARDT, L.M. Segurança do paciente na tríade assistência ensino pesquisa. **Rev. Gaúcha de Enferm.**, Porto Alegre, v. 34, n. 3, não p., 2013.

Disponível em:<http://seer.ufrgs.br/RevistaGauchadeEnfermagem/article/view/43294>. Acesso em: 15 jun. 2018.

VANE, F.M.; NUZZI, P.X.R.; ARANHA, F.G. et al. Perioperative cardiac arrest: an evolutionary analysis of the intra-operative cardiac arrest incidence in tertiary centers in Brazil. **Rev Bras Anestesiol**, v. 66, n. 2, p. 176-82, 2016. Disponível em:<https://doi.org/10.1016/j.bjan.2016.01.001>. Acesso em: 12 de set. 2019.

VARGAS-TRUJILLO, C.; AVELAR, D.L. Estrategias de manejo en el paciente con hipertensión arterial en el perioperatorio. **Rev. Mexicana de Anestesiol**., v. 42, n. 1, p. 166-168, 2019. Disponível em:<https://www.medigraphic.com/cgi-bin/new/resumen.cgi?IDARTICULO=87134>. Acesso em: 04 de out. 2019.

VERBEEK-VAN NOORD, I.N; WAGNER, C.; VAN DYCK, C. et al. Is culture associated with patient safety in the emergency department? A study of staff perspectives. **International Journal for Quality in Health Care**, v. 26, n. 1, p. 64-70, 2014. Disponível em:<http://intqhc.oxfordjournals.org/content/26/1/64>. Acesso em: 18 de jul. 2016.

VERONEZE, C.; MALUF, E.M.C.P.; GIORDANI, F. The use of trigger tools in the identification of adverse drug events. **Cogitare enferm** [Internet], v. 22, n. 2, não p., 2017. Disponível em:<http://dx.doi.org/10.5380/ce.v22i1.45632>. Acesso em: 01 de maio de 2019.

VICENT, C. **Segurança do paciente: orientações para evitar os eventos adversos**. Porto Alegre: Yendis, 2010.

VINAGRE, T.; MARQUES, R. Strategies for an Effective Safety Culture and Prevent Errors in Nursing: Literature **Review. Int J Nurs**., v. 5, n. 1, p. 25-32, 2018. Disponível em:<http://hdl.handle.net/10400.8/3911>. Acesso: 02 de set. 2019.

VINCENT, J.L.; MARSHALL, J.C.; NAMENDYS-SILVA, S.A. et al. Investigadores do ICON. Avaliação da carga mundial de doenças críticas: a auditoria de Cuidados Intensivos pelas Nações (ICON). **Lancet Respir Med**., v. 2, n. 5, p, 380-386, 2014. Disponível em:<https://www.sciencedirect.com/science/article/abs/pii/S221326001470061X >. Acesso em: 02 de set. 2019.

VITURI, D.; ÉVORA, Y.D.M. Fidedignidade de indicadores de qualidade do cuidado de enfermagem: testando a concordância e confiabilidade interavaliadores. **Rev**

Latino-Am Enfermagem, v. 22, n. 2, p. 234-240, 2014. Disponível em <http://dx.doi.org/10.1590/0104-1169.3262.2407>. Acesso: 25 de set. 2019.

WACHTER, R.M. **Compreendendo a segurança do paciente.** 2° ed. Artmed. Porto Alegre: AMGH, 2013.

WALKEY, A.J.; LAGU, T.; LINDENAUER, P.K. Trends in Sepsis and Infection Sources in the United States. A Population-Based Study. **Ann Am Thorac Soc.,** v. 12, n. 2, p. 216-220, 2015. Disponível em:<https://www.atsjournals.org/doi/full/10.1513/AnnalsATS.201411-498BC>. Acesso em: 14 de set. 2019.

WANG, C.H.; SHIH, C.L.; CHEN, W.J. et al. Epidemiology of medical adverse events: perspectives from a single institute in Taiwan. **J Formos Med Assoc**., v. 115, n. 6, p. 434-439, 2016. Disponível em:<https://doi. org/10.1016/j.jfma.2015.11.004>. Acesso em: 20 de set. 2019.

WANG, X.; LIU, K.; YOU, L.M. et al. The relationship between patient safety culture and adverse events: a questionnaire survey. **International Journal of Nursing Studies**, v. 51, n. 8, p. 1114-1122, 2014. Disponível em:<http://www.sciencedirect.com/science/article/pii/S0020748913003854>. Acesso em: 27 de set. 2018.

WILSON, R.M.; MICHEL, P.; OLSEN, S. et al. Patient safety in developing countries: retrospective estimation of scale and nature of harm to patients in hospital. **BMJ**, v. 344, p. e832, 2012. Disponível em:<http://www.bmj.com/content/344/bmj.e832.full>. Acesso em: 05 de jun. 2018.

WONG, C.; LASCHINGER, H. The influence of frontline manager job strain on burnout, commitment and turnover intention: A cross-sectional study. International **Journal of Nursing Studies**, Londres, v. 52, p. 1824–1833, 2015. Disponível em:<https://www.ncbi.nlm.nih.gov/pubmed/26394531>. Acesso em: 04 de nov. 2018.

WORLD HEALTH ORGANIZATION (WHO). **Guidelines for Safe Surgery, 2009**. Geneva: World Health Organization, 2009b. Disponível em:<https://proqualis.net/sites/proqualis.net/files/000000015kwXz3N.pdf>. Acesso em: 15 de maio 2019.

WORLD HEALTH ORGANIZATION (WHO). **Medication Without Harm - Global Patient Safety Challenge on Medication Safety.** Geneva: World Health Organization, 2017. Disponível em:<https://www.who.int/patientsafety/medication-safety/en/>. Acesso em: 15 de maio 2019.

WORLD HEALTH ORGANIZATION (WHO). **Patient Safety: Rapid assessment methods for estimating hazards, report of the WHO working group meeting.** Geneva: World Health Organization, 2003. Disponível em: https://apps.who.int/iris/handle/10665/42821. Acesso em: 06 abr., 2018.

WORLD HEALTH ORGANIZATION (WHO). **Summary of the evidence on patient safety: implications for research**. Geneva: World Alliance for Patient Safety, 2008. Acesso em: 15 de maio de 2019.

WORLD HEALTH ORGANIZATION (WHO). **Taxonomy: The conceptual framework for the international classification for patient safety: final technical report**. Genève: World Alliance For Patient Safety, 2009a. Disponíivel em:<http://www.who.int/patientsafety/taxonomy/icps_ full_report.pdf>. Acesso em: 02 de set. 2019.

WORLD HEALTH ORGANIZATION. WORLD ALLIANCE FOR PATIENT SAFETY**. WHO Draft guidelines for adverse event reporting and learning systems: from information to action**. Geneva: World Health Organization (WHO/EIP/SPO/QPS/05.3), 2005. Disponível em:<https://apps.who.int/iris/handle/10665/69797>. Acesso em: 5 out. 2019.

WORLD HEALTH ORGANIZATION (WHO). **World Alliance for Patient Safety. Forward programme**. World Health Organization, 2008a. Disponível em:<http://www.who.int/patientsafety/information_centre/reports/Alliance_Forward_Programme_2008.pdf>. Acesso em: 23 de Mar. 2019.

WORLD HEALTH ORGANIZATION (WHO). **World alliance for patient safety. The second Global Patient safety challenge. Safe surgery saves lives-Geneva-IRIS**, 2008b. Disponível em:<http://apps.who.int/iris/handle/10665/70080>. Acesso em: 23 Mar. 2019.

WORLD HEALTH ORGANIZATION (WHO). **WHO launches “Nine patient safety solutions**”. Geneva: World Health Organization, 2007. Disponível em:<http://www.who.int/mediacentre/news/releases/2007/pr22/en/>. Acesso em: 30 de maio 2018.

YAFANG, T. Relationship between organizational culture, leadership, behavior and job satisfaction. **BMC Health Serv. Res**. v. 11, n. 98, não p., 2011. Disponível em:<https://bmchealthservres.biomedcentral.com/articles/10.1186/1472-6963-11-98>. Acesso em: 05 de ago. 2018.

YANG, C.K.; TENG, A.; LEE, D.Y. et al. Pulmonary complications after major abdominal surgery: National Surgical Quality Improvement Program analysis. **Journal of Surgical Research**, v.198, n. 2, p. 441-449, 2015. Disponível em:<https://www.journalofsurgicalresearch.com/article/S0022-4804(15)00296-6/fulltext>. Acesso em: 20 de out. 2019.

ZAPATA, A.I.P.; SAMANIEGO, M.G.; CUÉLLAR, E.R., et al. Detection of adverse events in general surgery using the “Trigger Tool” methodology. **Cir. Esp**. (English Edition), v. 2, n. 93, p. 84-90, 2015. Disponível em:<https://www.elsevier.es/en-revista-cirugia-espanola-english-edition--436-pdf-S2173507714005298>. Acesso em: 15 de out. 2019.

ZHAO, X.; LIU, W.; WANG, Y. et al. Survey and analysis of patient safety culture in a county hospital. **Fam Med Community Health**., v. 5, n. 4, p. 299-310, 2017. Disponível em:<https://doi.org/10.15212/FMCH.2017.0137>. Acesso em: 2 de set. 2019.

ZWIJNENBERG, N.C.; HENDRIKS, M.; HOOGERVORST-SCHILP, J. et al. Healthcare professionals' views on feedback of a patient safety culture assessment. **BMC Health Serv Res**. v. 16, p. 199, 2016. Disponível em:<https://www.ncbi.nlm.nih.gov/pmc/articles/PMC4912740/>. Acesso em: 15 de set. 2019

APÊNDICE 1 – TERMO DE CONSENTIMENTO LIVRE E ESCLARECIDO

TERMO DE CONSENTIMENTO LIVRE E ESCLARECIDO

Nós, Prof. Drª Elaine Drehmer de Almeida Cruz, Enf.ª Ms. Francine Taporosky Alpendre e Enf.º Josemar Batista, pesquisadores da Universidade Federal do Paraná, estamos convidando você trabalhador da equipe multiprofissional a participar de um estudo **intitulado "Avaliação da cultura de segurança e ocorrência de eventos adversos cirúrgicos em hospitais brasileiros.** Esta pesquisa pretende verificar a percepção dos profissionais que prestam assistência à saúde sobre clima organizacional para segurança do paciente cirúrgico em hospitais de Curitiba (PR) e relacionar com a real ocorrência de eventos adversos, ou seja, danos ocasionados aos pacientes que foram submetidos à cirurgia.

O objetivo desta pesquisa é avaliar a relação entre cultura de segurança entre os trabalhadores da equipe multiprofissional, eventos adversos ocasionados em pacientes cirúrgicos e a gestão hospitalar.

Caso você participe da pesquisa, será necessário preencher o questionário validado e adaptado transculturalmente para o contexto brasileiro denominado Questionário sobre Segurança do Paciente em Hospitais (HSOPSC) - versão brasileira. Para tanto você deverá preencher mais da metade das perguntas que compõem o questionário. Considera-se que os riscos deste estudo podem estar relacionados a variável psicológica, caso você apresente algum constrangimento e/ou desconforto no preenchimento das perguntas do questionário. No entanto, se alguma pergunta do instrumento lhe causar desconforto, você poderá desistir do preenchimento do questionário ou não responder à pergunta que lhe causou desconforto.

Os benefícios esperados com essa pesquisa relacionam-se à compreensão dos problemas em relação a cultura de segurança entre o profissionais, elaboração e desenvolvimento de estratégias na prevenção de eventos adversos e gerenciais direcionadas à segurança do paciente. No entanto, nem sempre você será diretamente beneficiado com o resultado da pesquisa, mas poderá contribuir para o avanço científico.

Os pesquisadores Prof. Drª Elaine Drehmer de Almeida Cruz, Enf.ª Ms. Francine Taporosky Alpendre e Enf.º Josemar Batista, responsáveis por este estudo, poderão ser contatados nos seguintes locais e telefones:

- Pesquisador Responsável: Elaine Drehmer de Almeida Cruz.

Telefone para contato: (041) 3657-0362 - (041) 98416-8951
E-mail: elainedrehmercruz@gmail.com

- Pesquisador colaborador: Francine Taporosky Alpendre
  Telefone para contato: (041) 3360 -7831 - (041) 99102-1918
- E-mail: franalpendre@gmail.com
- Pesquisador colaborador: Josemar Batista

Telefone para contato: (045) 99948-6214
E-mail: josemar.batista1701@gmail.com

Endereço para contato dos pesquisadores: Av. Lothario Meissner, 632, bloco didático II, Jardim Botânico, de segunda à sexta-feira, no horário das 08:00 às 16:00 horas, para esclarecer eventuais dúvidas que você possa ter e fornecer-lhe as informações que queira, antes, durante ou depois de encerrado o estudo.

Se você tiver dúvidas sobre seus direitos como participante de pesquisa, você pode contatar Comitê de Ética em Pesquisa em Seres Humanos – CEP/HC/UPFR pelo Telefone 3360-1041. O CEP trata-se de um grupo de indivíduos com conhecimento científicos e não científicos que realizam a revisão ética inicial e continuada do estudo de pesquisa para mantê-lo seguro e proteger seus direitos.

A sua participação neste estudo é voluntária e se você não quiser mais fazer parte da pesquisa poderá desistir a qualquer momento e solicitar que lhe devolvam o termo de consentimento livre e esclarecido assinado.

As informações relacionadas ao estudo poderão ser conhecidas por especialistas que participaram da discussão para consenso após análise dos casos relativos a eventos adversos. No entanto, se qualquer informação for divulgada em relatório ou publicação, isto será feito sob forma codificada, para que a sua identidade seja preservada e seja mantida a confidencialidade.

As despesas necessárias para a realização da pesquisa não são de sua responsabilidade e pela sua participação no estudo você não receberá qualquer valor em dinheiro.

Quando os resultados forem publicados, não aparecerá seu nome, e sim um código.

Rubricas:

Participante da Pesquisa e /ou responsável legal_-__________

Pesquisador Responsável ou quem aplicou o

Eu,_______________________________________ li esse termo de consentimento e compreendi a natureza e objetivo do estudo do qual concordei em participar. A explicação que recebi menciona os riscos e benefícios. Eu entendi que sou livre para interromper minha participação a qualquer momento sem justificar minha decisão.

Eu concordo voluntariamente em participar deste estudo.

____

(Nome e Assinatura do participante da pesquisa ou responsável legal)
Local e data

(Somente para o responsável do projeto)

Declaro que obtive de forma apropriada e voluntária o Consentimento Livre e Esclarecido deste participante ou representante legal para a participação neste estudo.

____

(Nome e Assinatura do Pesquisador ou quem aplicou o TCLE)
Local e data

Rubricas:

Participante da Pesquisa e /ou responsável legal_-_________

Pesquisador Responsável ou quem aplicou o

APÊNDICE 2 – PERFIL DEMOGRÁFICO, CIRÚRGICO E ANESTÉSICO DOS PACIENTES

1. **Identificação:**
2. **Sexo:**

   ( ) Masculino ( ) Feminino

3. **Tipo de Procedimento Cirúrgico:**
4. **Idade:**

   ( ) 0 □20 ( ) 20 □40 ( ) 40 □60 ( ) ≥ 60

5. **Cirurgia:**

   ( ) Efetiva ( ) Emergência

6. **Potencial de Contaminação:**

   ( ) Limpa ( ) Potencialmente Contaminada

   ( ) Contaminada ( ) Infectada

7. **Risco Cirúrgico:**

   ( ) ASA I ( ) ASA II ( ) ASA III ( ) ASA IV ( ) ASA V

8. **Tipo de Anestesia:**

   ( ) Inalatória ( ) Geral ( ) Raquianestesia ( ) Peridural

   ( ) Local ( ) Outras

9. **Duração da Cirurgia:**

   ( ) ≥ 120 min ( ) > 120 min

10. **Tempo de Internação Pré-operatório**

    ( ) ≥ 24h ( ) < 24h

APÊNDICE 3 – SOLICITAÇÃO DE AUTORIZAÇÃO PARA TRADUÇÃO, ADAPTAÇÃO TRANSCULTURAL E VALIDAÇÃO

Authorization Request

Subject: Authorization for transcultural translation and adaptation of *Surgical Module Triggers*

As researchers of the Postgraduate Program in Nursing at the Federal University of Paraná, Curitiba, Brazil, we would like to request authorization to perform a transcultural translation and adaption to Brazilian Portuguese of the Surgical Module from the *Global Trigger Tool* and guidelines.

The translated and adapted version will be used in research approved by the Ethics Committee for Research with Human Beings of the institution participating in the study, under the Protocol No. 1.990.760. The purpose of the investigation is to assess the relationship between the safety culture and the occurrence of adverse surgical events in teaching hospitals in the southern region of Brazil. *Surgical Module Triggers* (S1 to S11) will be used for the identification and measurement of exclusively surgical Adverse Events during the retrospective review of medical records.

After the transcultural translation and adaptation, the instrument may be used by other Brazilian hospital services, according to the recommendations of the Institute for Healthcare Improvement Global Trigger Tool for Measuring Adverse Events and other current global and national legislation for Patient's Safety.

Thank you for your attention, we look forward to hearing from you.

Best regards,

Francine Taporosky Alpendre
Doctorate Student at the Postgraduate Program in Nursing
of the Federal University of Paraná (PPGENF-UFPR) – Curitiba/Brazil
Master in Nursing by PPGENF-UFPR - Curitiba/Brazil
Nurse at the Hospital of Clinics of UFPR - Curitiba/Brazil

Prof. Dr. Ana Maria Dyniewicz
Professor, PhD /PPGENF-UFPR, Curitiba/Brazil

Prof. Dr. Elaine Drehmer de Almeida Cruz
Professor, PhD and Tutor/PPGENF-UFPR, Curitiba/Brazil

APÊNDICE 4 – QUESTIONÁRIO PARA AVALIAÇÃO E VALIDAÇÃO DE INSTRUMENTO - FORMULÁRIO PARA ESPECIALISTA

IDENTIFICAÇÃO DO PROFISSIONAL ESPECIALISTA:______________________________

DATA: _____/_____/__________

BLOCO I - Surgical Module Global Trigger Tool for Measuring Adverse Events - Institute for Healthcare Improvement (2009)

	INSTRUMENTO ORIGINAL	INSTRUMENTO TRADUZIDO	INSTRUMENTO RETROTRADUZIDO	DISCORDO TOTALMENTE	DISCORDO	INDIFERENTE	CONCORDO	CONCORDO TOTALMENTE
S1	**Return to Surgery**	**Retorno à cirurgia**	**Return to Surgery**					
	A return to the operating room can either be planned or unplanned, and both can be a result of an adverse event. An example of an adverse event would be a patient who had internal bleeding following the first surgery and required a second surgery to explore for the cause and to stop the bleeding. Even if the second surgery is exploratory but reveals no defect, this should be considered an adverse event.	Um retorno à sala de cirurgia pode ser planejado ou não planejado, e ambos podem ser resultados de um evento adverso. Um exemplo de um evento adverso seria um paciente que tivesse uma hemorragia interna após a primeira cirurgia e necessitasse de uma segunda cirurgia para explorar a causa e parar o sangramento. Mesmo que a segunda cirurgia fosse exploratória e não indicasse nenhuma intercorrência, deveria ser considerada como um evento adverso.	Return to the operating room can be planned for or not , and both could be a result of an adverse event. An example of an adverse event could be a patient that had an internal bleeding after the first surgery and needed a second surgery to explore the cause and stop the bleeding. Even if the second surgery is exploratory but does not indicate an intercurrence, this should be considered an adverse event.					
S2	**Change in Procedure**	**Mudança no Procedimento**	**Change in Procedure**					
	When the procedure indicated on the post-operative notes is different from the procedure planned in the pre-operative notes or documented in the surgical consent, a reviewer should look for details as to why the change occurred. An unexpected change in procedure due to complications or device or equipment failure should be considered an adverse event, particularly if length of stay increases or obvious injury has occurred.	Quando o procedimento indicado nas anotações pós-operatórias for diferente em relação ao procedimento planejado nas anotações ou documentos pré-operatórios constantes no consentimento cirúrgico, um revisor deve procurar por detalhes que justifiquem a alteração. Uma mudança inesperada no procedimento devido a complicações ou falha nos dispositivos ou nos equipamentos deve ser considerada um evento adverso, particularmente se houver aumento no tempo de permanência do paciente ou se houver lesão evidente	When the registered procedure in the post operative documentation is different from the planned procedure in the pre-operative notes or included in the surgical consent documentation, a revisor must search for details that justify this change. An unexpected change in procedure due to complications or device or equipment failure should be considered an adverse event, particularly if when that increases the hospitalization period or in case an evident injury has occurred.					
S3	**Admission to intensive care post-op**	**Admissão ao tratamento intensivo no pós-operatório**	**Admission to intensive Care Post-op**					
	Admission to an intensive care unit can be either a normal post-operative journey or it may be unexpected. The unexpected admissions frequently are related to operative adverse events. For example, admission to intensive care following aortic aneurysm repair may be expected, but admission following knee replacement would be unusual. The reviewer needs to determine why intensive care admission occurred	A admissão em uma unidade de terapia intensiva pode ser uma indicação pós-operatória normal ou pode ser inesperada. As internações inesperadas frequentemente estão relacionadas à ocorrência de eventos adversos operatórios. Por exemplo, a admissão de um paciente em uma unidade de terapia intensiva após o reparo do aneurisma aórtico pode ser esperada, mas a admissão após uma cirurgia de substituição de joelho seria incomum. O revisor precisará verificar o porquê a internação na unidade de terapia intensiva ocorreu.	Admission to an intensive care unit can be either a normal post-operative indication or something unexpected. Unexpected admissions are frequently related to the occurrence of surgical Adverse events. For example, admission to Intensive Care following aortic aneurysm repair can be expected, but admission after knee replacement would be uncommon. The reviwer needs to find out why the admission at the intensive care unit happened.					
S4	**Intubation/reintubation/BiPap in post Anesthesia Care Unit (PACU)**	**Intubação/reintubação/ BiPap na unidade de recuperação pós anestesia (REPAI)**	**Intubation/reintubation/BiPap in post Anesthesia Care Unit (PACU)**					
	Anesthesia, sedatives, or pain medications can result in respiratory depression requiring the use of BiPap* or reintubation post-operatively, which would be an adverse event	Anestésicos, sedativos ou medicamentos para dor podem resultar em depressão respiratória, exigindo o uso de BiPap* ou reintubação pós-operatória, o que seria um evento adverso	Anesthesics, sedatives or pain killers could result in respiratory depression, which would require the use of BiPap or post-operative reintubation, which could be an adverse event					
S5	**X-Ray Intra-Operatively or in Post Anesthesia Ca**	**Raio-X intra-Operatório ou em Unidade de Cuidados Pós-Anestésicos**	**X-Ray Intra-Operative or in Post Anesthesia Care Unit**					
	Imaging of any kind that is not routine for the procedure requires investigation. An x-ray taken due to suspicion of retained items or incorrect instrument or sponge count would be a positive trigger. The identification of a retained item necessitating an additional procedure is an adverse event. If the retained item is identified and removed without any additional evidence of harm or re-operation to the patient, this is not considered an adverse event	Qualquer imagem que não seja rotineira ao procedimento requer investigação. Um raio-x realizado devido à suspeita de itens retidos ou contagem incorreta de instrumento ou compressa seria um gatilho positivo. A identificação de um item retido que necessite de um novo procedimento é um evento adverso. Se o item retido for identificado e removido sem qualquer evidência adicional de dano ou reoperação do paciente, isso não é considerado um evento adverso	Any image that is not routine to the procedure requires investigation. An X-ray performed due to suspicion of retained items or incorrect instrument or dressings count would be a positive trigger. The identification of a retained item requiring a new intervention is an adverse event. If the retained item is identified and removed without any additional evidence of injury or patient reoperation, this is not considered an adverse event					

S6	**Intra- or Post-Operative Death**	**Morte intra ou pós-operatória**	**Intra- or Post-Operative Death**					
	All deaths that occur intra-operatively should be considered adverse events unless death is clearly expected and the surgery was of a heroic nature. Post-operative deaths will require review of the record for specifics, but in general all post-op deaths will be adverse events	Todas as mortes que ocorrem no intra-operatório devem ser consideradas eventos adversos, a menos que a morte seja claramente esperada e a cirurgia tenha sido de indicação extrema. Mortes pós-operatórias deverão exigir revisão nos registros em busca de especificidades, mas em geral todas as mortes pós-operatórias serão eventos adversos	All deaths that occur in intra-operative period must be considered as adverse events unless death is clearly expected and the surgery was an extreme indication. Post-operative deaths shall require that the records be checked for specifics, but in general, all post operative deaths shall be considered adverse events					
S7	**Mechanical Ventilation Greater than 24 Hours Post-Operatively**	**Ventilação mecânica superior a 24 Horas no Pós-Operatório**	**Mechanical ventilation exceeding 24 hours in post-operative**					
	Short-term mechanical ventilation post-operatively for cardiac, major thoracic, and certain abdominal procedures is planned. If the patient requires mechanical ventilation beyond 24 hours, an intra-operative or post-operative adverse event should be considered. Patients with pre-existing pulmonary or muscular disease may experience more difficulty in quickly weaning from a ventilator post-operatively, but this should not automatically exclude the possibility of an adverse event. Reviewers must use clinical judgment to determine whether the intra-operative and post-operative care was event free or part of the disease process	A ventilação mecânica de curto prazo no pós-operatório para procedimentos cardíacos, torácicos grandes e certos procedimentos abdominais está planejada. Se o paciente necessitar de ventilação mecânica superior a 24 horas, um evento adverso intra-operatório ou pós-operatório deve ser considerado. Pacientes com doença pulmonar ou muscular pré-existente podem ter mais dificuldade em sair rapidamente da ventilação mecânica no pós-operatório, mas isso não deve excluir automaticamente a possibilidade de ser um evento adverso. Os revisores devem usar o julgamento clínico para determinar se os cuidados intra-operatórios e pós-operatórios eram necessários ou eram parte do processo da doença	Short-period mechanical ventilation in post-operative period for cardiac, major thoracic and some abdominal procedures is planned. If the patient needs mechanical ventilation up to 24 hours, an intra or post-operative adverse event should be considered. Patients with pre-existing pulmonary or muscular disease may face more difficulty in getting out of mechanical ventilation post operatively, but this should not automatically exclude the possibility of an adverse event. Revisors must use clinical judgement to determine if whether intra-operative or post-operative care were necessary or whether they were part of the disease process.					
S8	**Intra-Op, Epinephrine, Norepinephrine, Naloxone, or Romazicon**	**Uso intra operatório de Epinefrina, Norepinefrina, Naloxona ou Romazicon**	**Intra-Operative, Epinephrine, Norepinephrine, Naloxone, or Romazicon**					
	Review anesthesia and operative notes to determine the reason for administration. Hypotension caused by bleeding or over-sedation are examples of adverse events that might be treated with these medications	Revisar anotações da anestesia e operação para determinar o motivo da administração. Hipotensão causada por sangramento ou sedação excessiva são exemplos de eventos adversos que podem ser tratados com esses medicamentos	Revise anesthesia and operation notes to determine the motive for the administration. Hypotension caused by bleeding or excessive sedation are examples of adverse events that can be treated with these medications					
S9	**Post-Operative Increase in Troponin Levels Greater than 1.5 ng/ml**	**Aumento pós-operatório nos níveis de troponina superior a 1,5 ng / ml**	**Post-Operative Increase in Troponin Levels Greater than 1.5 ng/ml**					
	A post-operative increase in troponin levels may indicate a cardiac event. Reviewers will need to use clinical judgment as to whether a cardiac event has occurred	Um aumento pós-operatório nos níveis de troponina pode indicar um evento cardíaco. Revisores precisarão utilizar julgamento clínico para determinar se um evento cardíaco ocorreu	A post-operative increase in troponin levels may indicate a cardiac event. Reviewers shall need to use clinical judgment to determine whether a cardiac event has occurred					
S10	**Injury, Repair, or Removal of Organ**	**Lesão, reparo ou remoção de Órgão**	**Injury, Repair, or Removal of Organ**					
	Review operative notes and post-operative notes for evidence that the procedure included repair or removal of any organ. The removal or repair must be part of the planned procedure or this is an adverse event and likely the result of surgical misadventure such as an accidental injury	Revisar as anotações operatórias e pós-operatórias em busca de evidências de que o procedimento incluía reparo ou remoção de algum órgão. A remoção ou reparo deve fazer parte do procedimento planejado ou isto é um evento adverso e provavelmente resultado de uma intercorrência cirúrgica como uma lesão acidental	Review operative and post-operative notes for evidence that the procedure was to include repair or removal of any organ. The removal or repair must be part of the planned procedure or it is an adverse event and probably the result of a surgical intercurrence such as and accidental injury					
S11	**Any Operative Complication**	**Qualquer complicação operatória**	**Any Operative Complication**					
	This refers to any of a number of complications, including but not limited to PE*, DVT*, decubiti, MI*, renal failure, etc	Isso se refere a qualquer dentre inúmeras complicações incluindo, mas não se limitando a Embolia Pulmonar, Trombose Venosa Profunda, decúbito, Isquemia do Miocárdio, falência renal, etc	This refers to any among a number of complications, including but not limited to Pulmonary Embolism, Deep Vein Thrombosis, decubitus, Myocardial Ischemia, renal failure, etc					

BLOCO II - Surgical Module Global Trigger Tool for Measuring Adverse Events - Institute for Healthcare Improvement (2009)							
**AVALIAÇÃO DAS EQUIVALÊNCIAS**		**DISCORDO TOTALMENTE**	**DISCORDO**	**INDIFERENTE**	**CONCORDO**	**CONCORDO TOTALMENTE**	**OBSERVAÇÕES / SUGESTÕES**
**Equivalência semântica**	Considera a adaptação das palavras de acordo com a gramática e vocabulário da língua portuguesa.						
**Equivalência idiomática**	Está relacionada às expressões coloquiais, informais ou gírias utilizadas no país de origem que podem apresentar dificuldades de tradução						
**Equivalência conceitual**	Consiste em verificar se as palavras que têm conotação conceitual estão adequadas ao contexto estudado						
**Equivalência experencial**	É necessário identificar se a versão adaptada expressa a experiência vivenciada no cotidiano dos serviços de saúde no Brasil						
**Equivalência de conteúdo**	Avalia-se se os itens do instrumento em relação à aparência, clareza, aplicabilidade, conceitos, linguagem, bem como se o conteúdo é adequado ao que se propõe mensurar						

APÊNDICE 5 – E-MAIL ENVIADO AOS DESENVOLVEDORES REFERENTE À FINALIZAÇÃO DO PROCESSO DE TRADUÇÃO, ADAPTAÇÃO E VALIDAÇÃO

From: FRANCINE ALPENDRE <franalpendre@gmail.com>
Sent: Monday, August 12, 2019 9:23 PM
To: Info <info@IHI.org>; Frank Federico <ffederico@IHI.org>
Cc: Val Weber <VWeber@IHI.org>
Subject: Translation and cross-cultural adjustment to Brazilian Portuguese of the G.T.Tool surgical module, according to the authorization granted by IHI

We have forwarded an attached document regard the translation and cross-cultural adjustment to Brazilian Portuguese of the Global Trigger Tool surgical module, according to the authorization granted by the Institute for Healthcare Improvement in 2018.

The study followed the guidelines of Brazilian resolution 466/2012 and it was registered in the Brazil Platform approved by the Committee of Ethics in Research.

This study was carried out through methodological research, of quantitative approach comprised of six stages: translation, synthesis, back-translation, validation by experts, pre-test and presentation to its developers.

The surgical module of the instrument and its guidelines for the triggers (S1 to S11) contained in the Guide IHI Global Trigger Tool for Measuring Adverse Events (2009) were validated with Content Validity Index ≥1, degree of agreement ≥80% and Alpha of Cronbach >0.70.

Following your instruction we have included the credit line with the translated material:
Source: Adapted from the Institute for Healthcare Improvement Global Trigger Tool for Measuring Adverse Events.http://www.ihi.org/resources/Pages/IHIWhitePapers/IHIGlobalTriggerToolWhitePaper.aspx

As you have requested we are sending the version translated and adapted to Brazilian Portuguese of the Global Trigger Tool.

We appreciate the trust deposited for the translation and cross-cultural adaptation of the instrument to our language. We are at your disposal for any clarifications that may be necessary.

We look forward the publishing of the Brazilian Portuguese version on the Institute for Healthcare Improvement website.

Most respectfully,

Francine Taporosky Alpendre
Doctorate Student at the Postgraduate Program in Nursing
of the Federal University of Paraná (PPGENF-UFPR) – Curitiba/Brazil
Master in Nursing by PPGENF-UFPR - Curitiba/Brazil
Nurse at the Hospital of Clinics of UFPR - Curitiba/Brazil

Prof. Dr. Ana Maria Dyniewicz
Professor, PhD /PPGENF-UFPR, Curitiba/Brazil

Prof. Dr. Elaine Drehmer de Almeida Cruz
Professor, PhD and Tutor/PPGENF-UFPR, Curitiba/Brazil

APÊNDICE 6 – SUGESTÕES DOS ESPECIALISTAS SOBRE A TRADUÇÃO E ADAPTAÇÃO TRANSCULTURAL DOS TERMOS E FRASES DO MÓDULO CIRÚRGICO GLOBAL TRIGGER TOLL.

- T*rigger* S1 - trocado **retorno à cirurgia** para **retorno à sala cirúrgica***;*
- *Trigger* S3 - modificado a frase **admissão ao cuidado intensivo no pós-operatório** para **admissão em unidade de terapia intensiva no pós-operatório**;
- Trigger S3 orientação - foi modificada a frase *o* **revisor precisa verificar o porquê a internação na unidade de cuidado intensivo ocorreu** para **o revisor precisa verificar o motivo da internação na Unidade de Terapia Intensiva;**
- *Trigger* S6 - trocado termo **morte** por **óbito**;
- *Trigger* S9 - foi aceito a sugestão de escrita para **aumento nos níveis de troponina superior a 1,5 ng/ml no pós-operatório**
- *Trigger* S9 orientação - foi modificada a ordem dos termos na frase para melhor adaptação transcultural e compreensão para **aumento nos níveis de troponina superior a 1,5 ng/ml no pós-operatório pode indicar evento cardíaco***;*
- *Trigger* S11 - trocado o termo **decúbito** por **lesão por pressão;**
- *Trigger* S11 orientação - alterou-se a frase **isso se refere a qualquer dentre inúmeras complicações** pela frase **trata-se de qualquer uma entre várias complicações**;
- Modificado o termo **operatório** para **cirúrgico** e trocado o termo **operação** por **cirurgia** para padronização em todo o instrumento;
- Padronização do termo **unidade de recuperação pós-anestésica** em todo o instrumento**.**

ANEXO A – QUESTIONÁRIO HOSPITAL SURVEY ON PATIENT SAFETY CULTURE – HSOPSC

PESQUISA SOBRE SEGURANÇA DO PACIENTE

Esta pesquisa solicita sua opinião sobre segurança do paciente, erros associados ao cuidado de saúde e notificação de eventos no hospital e tomará cerca de 10 a 15 minutos para o preenchimento.
- *Se não quiser responder uma questão, ou se não se aplicar a você, pode deixá-la em branco.*

- Um "incidente" é definido como evento ou circunstância que poderia ter resultado, ou resultou, em dano desnecessário à saúde (RDC 36).
- "Segurança do paciente" é definida como redução, a um mínimo aceitável, do risco de dano desnecessário associado à atenção à saúde (RDC 36).
- "Erro" é uma falha na execução de uma ação planejada de acordo com o desejado ou o desenvolvimento incorreto de um plano (OMS, 2011).

SEÇÃO A - Seu serviço/unidade de trabalho: ________________________
Turno de trabalho: ____________

- *Considere serviço/unidade como área de trabalho, departamento, ou área clínica do hospital onde você passa a maior parte do seu tempo ou na qual presta a maior parte de seus serviços.*

Por favor, indique sua concordância ou discordância em relação às seguintes afirmações sobre seu serviço/unidade:

Pense em seu serviço/unidade de trabalho no hospital.	Discordo totalmente	Discordo	Nenhum	Concordo	Concordo totalmente
1. Nesta unidade, as pessoas apóiam umas às outras.	O	O	O	O	O
2. Temos profissionais suficientes para dar conta da carga de trabalho.	O	O	O	O	O
3. Quando há muito trabalho a ser feito rapidamente, trabalhamos juntos em equipe para concluí-lo devidamente.	O	O	O	O	O
4. Nesta unidade as pessoas se tratam com respeito.	O	O	O	O	O
5. Os profissionais desta unidade trabalham mais horas do que seria o melhor para o cuidado do paciente -considerar banco de horas, hora extra, APH	O	O	O	O	O
6. Estamos ativamente fazendo coisas para melhorar a segurança do paciente.	O	O	O	O	O
7. Utilizamos mais profissionais terceirizados do que seria desejável para o cuidado do paciente.	O	O	O	O	O
8. Os profissionais consideram que seus erros podem ser usados contra eles.	O	O	O	O	O
9. Erros têm levado a mudanças positivas por aqui.	O	O	O	O	O
10. É apenas por acaso, que erros mais graves não acontecem por aqui.	O	O	O	O	O
11. Quando uma área desta unidade fica sobrecarregada, os outros profissionais desta unidade/serviço ajudam.	O	O	O	O	O
12. Quando um evento é notificado, parece que o foco recai sobre a pessoa e não sobre o problema.	O	O	O	O	O
13. Após implementarmos mudanças para melhorar a segurança do paciente ocorrem, avaliamos a efetividade.	O	O	O	O	O
14. Nós trabalhamos em "situação de crise", tentando fazer muito e muito rápido.	O	O	O	O	O
15. A segurança do paciente jamais é comprometida em função de maior quantidade de trabalho a ser concluída.	O	O	O	O	O
16. Os profissionais se preocupam que seus erros sejam registrados em suas fichas funcionais.	O	O	O	O	O
17. Nesta unidade temos problemas de segurança do paciente.	O	O	O	O	O
18. Os nossos procedimentos e sistemas são adequados para prevenir a ocorrência de erros.	O	O	O	O	O

SEÇÃO B - O seu Supervisor/Chefe

Por favor, indique sua concordância ou discordância com relação às seguintes afirmações sobre seu supervisor/chefe imediato ou pessoa a quem se reporta diretamente.

Pense em seu serviço/unidade de trabalho no hospital.	Discordo totalmente	Discordo	Nenhum	Concordo	Concordo totalmente
1. O meu supervisor/chefe elogia quando vê um trabalho realizado de acordo com os procedimentos estabelecidos de segurança do paciente.	O	O	O	O	O
2. O meu supervisor/chefe realmente leva em consideração as sugestões dos profissionais para a melhoria da segurança do paciente.	O	O	O	O	O
3. Sempre que a pressão aumenta, meu supervisor/chefe quer que trabalhemos mais rápido, mesmo que isso signifique "pular etapas".	O	O	O	O	O
4. O meu supervisor/chefe não dá atenção aos problemas de segurança do paciente que acontecem repetidamente.	O	O	O	O	O

SEÇÃO C - Comunicação

Com que frequência as situações abaixo ocorrem na seu serviço/unidade de trabalho?

Pense em seu serviço/unidade de trabalho no hospital.	Discordo totalmente	Discordo	Nenhum	Concordo	Concordo totalmente
1. Nós recebemos informação sobre mudanças implementadas a partir das notificações de incidentes.	O	O	O	O	O
2. Os profissionais têm liberdade para falar ao ver algo que pode afetar negativamente o cuidado ao paciente.	O	O	O	O	O
3. Nós somos informados sobre os erros que acontecem neste serviço/unidade.	O	O	O	O	O
4. Os profissionais sentem-se à vontade para questionar as decisões ou ações de seus superiores.	O	O	O	O	O
5. Nesta unidade, discutimos meios de prevenir erros evitando que eles aconteçam novamente.	O	O	O	O	O
6. Os profissionais têm receio de perguntar, quando algo parece não estar certo.	O	O	O	O	O

SEÇÃO D - Frequência de notificações de incidentes

No seu serviço/unidade de trabalho no hospital, quando ocorrem as falhas a seguir, com que frequência elas são relatadas?

	Nunca	Raramente	Às vezes	Quase sempre	Sempre
1. Quando ocorre um erro, mas ele é percebido e corrigido antes de afetar o paciente, com que frequência ele é notificado?	O	O	O	O	O
2. Quando ocorre um erro, mas não há risco de dano ao paciente, com que frequência ele é notificado?	O	O	O	O	O
3. Quando ocorre um erro, que poderia causar danos ao paciente, mas não causa, com que frequência ele é notificado?	O	O	O	O	O

SEÇÃO E - Nota de Segurança do Paciente

Por favor, avalie a segurança do paciente na sua área/unidade de trabalho no hospital.

O	O	O	O	O
**A**	**B**	**C**	**D**	**E**
**Excelente**	**Muito boa**	**Regular**	**Ruim**	**Muito ruim**

SEÇÃO F - O seu hospital

Por favor, indique sua concordância ou discordância com relação às afirmações sobre seu hospital.

**Pense no seu hospital.**	**Discordo totalmente**	**Discordo**	**Nenhum**	**Concordo**	**Concordo totalmente**
1. A direção do hospital propicia um clima de trabalho que promove a segurança do paciente.	O	O	O	O	O
2. As unidades do hospital não estão coordenadas entre si.	O	O	O	O	O
3. O processo de cuidado é comprometido quando um paciente é transferido de uma unidade para outra.	O	O	O	O	O
4. Há uma boa cooperação entre as unidades do hospital que necessitam trabalhar em conjunto.	O	O	O	O	O
5. É comum a perda de informações importantes sobre o cuidado do paciente durante as mudanças de plantão ou de turno.	O	O	O	O	O
6. Muitas vezes é desagradável trabalhar com profissionais de outras unidades do hospital.	O	O	O	O	O
7. Com frequência ocorrem problemas na troca de informações entre as unidades do hospital.	O	O	O	O	O
8. As ações da direção do hospital demonstram que a segurança do paciente é uma das principais prioridades.	O	O	O	O	O
9. A direção do hospital só parece interessada na segurança do paciente quando ocorre algum evento adverso.	O	O	O	O	O
10. As unidades do hospital trabalham bem em conjunto para prestar o melhor cuidado aos pacientes.	O	O	O	O	O
11. Neste hospital, as mudanças de plantão ou de turno são problemáticas para os pacientes.	O	O	O	O	O

SEÇÃO G - Número de eventos notificados

Nos últimos 12 meses, quantas notificações de eventos você realizou?

- O a. Nenhuma
- O b. 1 a 2
- O c. 3 a 5
- O d. 6 a 10
- O e. 11 a 20
- O f. 21 ou mais

SEÇÃO H - Informações Gerais

- *<u>As informações a seguir contribuirão para a análise dos resultados da pesquisa.</u>*

1. Há quanto tempo você trabalha neste hospital?

- O a. Menos de 1 ano
- O b. 1 a 5 anos
- O c. 6 a 10 anos
- O d. 11 a 15 anos
- O e. 16 a 20 anos
- O f. 21 anos ou mais

2. Há quanto tempo você trabalha no seu atual serviço/unidade do hospital?

- O a. Menos de 1 ano
- O b. 1 a 5 anos
- O c. 6 a 10 anos
- O d. 11 a 15 anos
- O e. 16 a 20 anos
- O f. 21 anos ou mais

3. Normalmente, quantas horas por semana você trabalha neste hospital?

- O a. Menos que 20 horas por semana
- O b. 20 a 39 horas por semana
- O c. 40 a 59 horas por semana
- O d. 60 a 79 horas por semana
- O e. 80 a 99 horas por semana
- O f. 100 horas por semana ou mais

3.1 Você trabalha em outra instituição?

- O Sim
- O Não

Se sim, quantas horas trabalha na outra instituição?

- O menos de 20 horas por semana
- O 20 a 39 horas por semana
- O 40 a 59 horas por semana
- O trabalha com atendimento particular, esporadicamente

4. Qual é o seu cargo/função neste hospital?

- O a. Enfermeiro
- O b. Técnico de Enfermagem
- O c. Auxiliar de Enfermagem
- O d. Médico do Corpo Clínico/ Médico Assistente
- O e. Médico Residente/Médico em treinamento
- O f.Farmacêutico/Bioquímico/Biólogo/Biomédico
- O g. Nutricionista
- O h. Assistente Social
- O i. Odontólogo
- O j. Psicólogo
- O h. Fisioterapeuta/Terapeuta Respiratório/ Terapeuta Ocupacional/Fonoaudiólogo
- O Técnico (por ex.: ECG, Laboratório, Radiologia, Farmácia)
- O m. Administração/Direção
- O n. Auxiliar Administrativo/Secretário
- O o. Outro - especifique:________________

____

4.1 Você exerce função essencialmente administrativa?

- O Sim
- O Não

5. No seu cargo/função, em geral você tem interação ou contato direto com os pacientes?

- O a. SIM, em geral tenho interação ou contato direto com os pacientes.
- O b. NÃO, em geral NÃO tenho interação ou contato direto com os pacientes.

6. Há quanto tempo você trabalha na sua especialidade ou profissão atual?

____ anos

7. Qual seu grau de instrução?

- O a. Primeiro grau (Ensino Básico) Incompleto
- O b. Primeiro grau (Ensino Básico) Completo
- O c. Segundo grau (Ensino Médio) Incompleto
- O d. Segundo grau (Ensino Médio) Completo
- O c. Ensino Superior Incompleto
- O f. Ensino Superior Completo
- O g. Pós-graduação (Nível Especialização)
- O h. Pós-graduação (Nível Mestrado ou Doutorado)

8. Qual a sua idade?

____ anos

9. Indique o seu sexo:

- O Feminino
- O Masculino

OBRIGADO POR VOCÊ COMPLETAR ESTE QUESTIONÁRIO E PARTICIPAR DESTA PESQUISA

ANEXO B – CRITÉRIOS DE RASTREAMENTO DE POTENCIAIS EVENTOS ADVERSOS CIRÚRGICOS

CRITÉRIOS DE RASTREAMENTO POTENCIAIS EVENTOS ADVERSOS CIRÚRGICOS
Ao detectar na fase I ao menos 01 evento adverso deverá ser detalhado a ocorrência no campo informações. Em seguida, preencher o perfil clínico demográfico dos pacientes. Na fase II, caso não se confirme o potencial EA deverá ser justificado ao final dessa planilha.

Critério de Rastreamento (Fase I)		Definição	Confirmado Potencial EA (Fase II)			
1	( )	Ocorrência de lesão no paciente durante a internação (inclusive qualquer dano, lesão ou trauma ocorrido durante a internação Índice).	( )	Sim	( )	Não
2	( )	Reação adversa ao medicamento.	( )	Sim	( )	Não
3	( )	Transferência não planejada para a unidade de cuidados intensivos e semi-intensivos.	( )	Sim	( )	Não
4	( )	Transferência não planejada para outro hospital de cuidados agudos (excluindo as transferências para exames, procedimentos, ou cuidados especializados não disponíveis no hospital de origem).	( )	Sim	( )	Não
5	( )	Retorno não planejado à sala de cirurgia.	( )	Sim	( )	Não
6	( )	Remoção, lesão ou correção não planejada de órgão ou estrutura durante cirurgia ou procedimento invasivo.	( )	Sim	( )	Não
7	( )	Outras complicações inesperadas ocorridas durante a internação em referência que não sejam um desenvolvimento normal do paciente ou um resultado esperado do tratamento.	( )	Sim	( )	Não
8	( )	Desenvolvimento de alteração neurológica ausente na admissão, mas presente no momento da saída da internação Índice (inclui alterações neurológicas relacionadas aos procedimentos, tratamentos ou investigações.	( )	Sim	( )	Não
9	( )	Óbito.	( )	Sim	( )	Não
10	( )	Alta hospitalar inapropriada / planejamento de alta inadequado para a internação Índice (exclui alta à revelia).	( )	Sim	( )	Não
11	( )	Parada cardiorrespiratória revertida.	( )	Sim	( )	Não
12	( )	Infecção/septicemia hospitalar (exclui infecções/septicemia ocorridas em menos de 72 horas após a admissão).	( )	Sim	( )	Não

13	( )	Insatisfação com o cuidado recebido documentada no prontuário ou evidência de queixa apresentada (incluir documentos, queixas documentadas, conflitos entre o paciente/família e profissionais e alta à revelia).	( )	Sim	( )	Não
14	( )	Documentação ou correspondência indicando litígio, seja somente intenção ou ação efetiva.	( )	Sim	( )	Não
15	( )	Partindo de uma creatinina normal na internação, houve duplicação do seu valor durante a permanência no hospital?	( )	Sim	( )	Não
16	( )	Quaisquer outros eventos indesejados não mencionados acima.	( )	Sim	( )	Não

Informações/Justificativas:

Fonte: Adaptado de Mendes et. Al., (2008)

ANEXO C – CRITÉRIOS DE RASTREAMENTO EVENTOS ADVERSOS – MÓDULO CIRÚRGICO GLOBAL TRIGGER TOOL

	Surgical Module Triggers		
S1	Return to surgery		
S2	Change in procedure		
S3	Admission to intensive care post-op		
S4	Intubation/reintubation/BiPap in Post Anesthesia Care Unit (PACU)		
S5	X-ray intra-op or in PACU		
S6	Intra-op or post-op death		
S7	Mechanical ventilation greater than 24 hours post-op		
S8	Intra-op epinephrine, norepinephrine, naloxone, or romazicon		
S9	Post-op troponin level greater than 1.5 ng/ml		
S10	Injury, repair, or removal of organ		
S11	Any operative complication		

Fonte: *Surgical Module Global Trigger Tool for Measuring Adverse Events - Institute for Healthcare Improvement, 2009.*

ANEXO D – FORMULÁRIO DE INVESTIGAÇÃO DE EVENTOS ADVERSOS

1 – DADOS DE INTERNAÇÃO

Data de admissão_____/______/______ Data de alta/Óbito_____/______/______ Total dias de Internamento______

Diagnóstico principal: ________________________________ Diagnóstico de saída: ________________________________

Diagnósticos secundários: ______________________________________; ______________________________; ___________________________________

Observações: ____

2 – FATORES DE RISCO/COMORBIDADE

Intrínseco (relacionado ao paciente) ________________________________;__________________________;____;

___________________________________;__________________________;______________________________________;___________________________.

Extrínseco (relacionado a procedimentos)___________________________;__________________________;____;

___________________________________;__________________________;______________________________________;___________________________.

3 - CLASSIFICAÇÃO DO EA CIRÚRGICO

Evento mais importante (selecionar apenas um)	Outro (s) evento (s) correlatado (s) (selecionar todos os aplicáveis)
( ) Problema técnico (especificar) __________________________	( ) Problema técnico (especificar) ______________________
( ) Hemorragia grave	( ) Hemorragia grave
( ) Hemorragia leve	( ) Hemorragia leve
( ) Infecção de ferida cirúrgica	( ) Infecção de ferida cirúrgica
( ) Infecção não relacionada a ferida cirúrpgica (ex: abscesso)	( ) Infecção não relacionada a ferida cirúrgica (ex: abscesso)
( ) Outros problemas com a ferida cirúrgica (ex: deiscência)	( ) Outros problemas com a ferida cirúrgica (ex: deiscência)
( ) Ruptura de anastomose	( ) Ruptura de anastomose
( ) Dificuldade na definição anatômica (especificar) _____________________	( ) Dificuldade na definição anatômica (especificar) _______
( ) Perfuração (especificar) ______________________________	( ) Perfuração (especificar) ___________________________
( ) Colocação de prótese	( ) Colocação de prótese
( ) Acidente Vascular Cerebral	( ) Acidente Vascular Cerebral
( ) Insuficiência Cardíaca Congestiva	( ) Insuficiência Cardíaca Congestiva
( ) Infarto do Miocárdio	( ) Infarto do Miocárdio
( ) Trombose Venosa Profunda	( ) Trombose Venosa Profunda
( ) Pneumonia	( ) Pneumonia

( ) Embolia Pulmonar	( ) Embolia Pulmonar
( ) Outros eventos ou comentários ____________________	( ) Outros eventos ou comentários ____________________

FONTE: Adaptado de MENDES (2007).

ANEXO E – FORMULÁRIO DE AVALIAÇÃO E EVITABILIDADE DE EVENTO ADVERSO CIRÚRGICO

FORMULÁRIO DE AVALIAÇÃO E EVITABILIDADE EVENTO ADVERSO (Fase II)

Obs: deverá ser preenchido esse formulário para cada EA no paciente

Possível EA:

A. CONFIRMAÇÃO DO EVENTO ADVERSO CIRÚRGICO

1	( )	Praticamente nenhuma evidência de que o cuidado foi a causa da lesão ou dano.
2	( )	Evidência mínima a moderada de que o cuidado foi a causa da lesão ou dano
3	( )	Evidência improvável: menos do que 50 % de que cuidado foi a causa da lesão ou dano
4	( )	Evidência provável, mais do que 50% de que cuidado foi a causa da lesão ou dano.
5	( )	Evidência de moderada a forte de que cuidado foi a causa da lesão ou dano
6	( )	Evidência praticamente certa de que cuidado foi a causa da lesão ou dano

B. GRAU DE EVITABILIDADE DO EVENTO ADVERSO CIRÚRGICO

1	( )	Praticamente nenhuma evidência de possibilidade de evitabilidade.
2	( )	Evidência mínima a moderada para possibilidade de evitabilidade.
3	( )	Evidência improvável: pouco menos do que 50 % para possibilidade de evitabilidade.
4	( )	Evidência provável, pouco mais do que 50% para possibilidade de evitabilidade.
5	( )	Evidência de moderada a forte para possibilidade de evitabilidade.
6	( )	Evidência praticamente certa para possibilidade de evitabilidade.

Fonte: Mendes (2007).

ANEXO F – FORMULÁRIO DE AVALIAÇÃO DA CLASSIFICAÇÃO DO DANO DO EVENTO ADVERSO

	**GRAU DO DANO**	**DEFINIÇÃO**
( )	Nenhum	-
( )	Leve	Paciente apresentou sintomas leves, danos mínimos ou intermediários de curta duração sem intervenção mínima (pequeno tratamento ou observação)
( )	Moderado	Necessitou de intervenção (por ex. procedimento suplementar ou terapêutica adicional), prolongamento da internação, perda de função, danos permanentes ou em longo prazo
( )	Grave	Necessária intervenção para salvar a vida, grande intervenção médico/cirúrgica ou causou grandes danos permanentes ou em longo prazo, perturbação/risco fetal ou anomalia congênita.
( )	Óbito	Causado pelo evento adverso

Fonte: WHO (2009).

ANEXO G – EMAIL DE AUTORIZAÇÃO PARA REALIZAÇÃO DO PROCESSO DE TRADUÇÃO, ADAPTAÇÃO TRANSCULTURAL E VALIDAÇÃO

Permission request: Translate IHI Surgical Trigger Module into Brazilian Portugese Caixa de entrada × IHI ×

Val Weber <VWeber@ihi.org> 28 de mar de 2018 13:11
para eu, Info, Frank

inglês > português Traduzir mensagem Desativar para: inglês ×

Hello Francine,

Thank you for your interest in IHI and our trigger tool.

We thank you for asking permission and welcome your language translation of the trigger tool content. Since we do not speak your language, we will not be able to comment on your translation.

We do, however, ask that you provide proper attribution to IHI for the original content — with clear notation that the original content was adapted and translated to Brazilian Portugese by you.

Please include the following credit line with the translated material:
Source: Adapted from the Institute for Healthcare Improvement Global Trigger Tool for Measuring Adverse Events. http://www.ihi.org/resources/Pages/IHIWhitePapers/IHIGlobalTriggerToolWhitePaper.aspx

And, we also ask that you share the translated version with us when it is complete so that we might consider posting it in on the IHI website, to share with others.

Thank you,
--Val
Valerie Weber
Institute for Healthcare Improvement
53 State Street, 19th Floor
Boston, MA 02109
www.ihi.org

ANEXO H – E-MAIL RESPOSTA DO DESENVOLVEDOR DO INSTRUMENTO APÓS A FINALIZAÇÃO DO PROCESSO DE TRADUÇÃO, ADAPTAÇÃO TRANSCULTURAL E VALIDAÇÃO

From: FRANCINE ALPENDRE <franalpendre@gmail.com>
Sent: Monday, August 12, 2019 9:23 PM
To: Info <info@IHI.org>; Frank Federico <ffederico@IHI.org>
Cc: Val Weber <VWeber@IHI.org>
Subject: Translation and cross-cultural adjustment to Brazilian Portuguese of the G.T.Tool surgical module, according to the authorization granted by IHI

Frank Federico <ffederico@ihi.org> ter, 13 de ago 10:31
para eu, Info, Val

Thank you for your great work. Congratulations. Parabens.

Frank Federico, RPh

Vice President, Senior Safety Expert
Mobile: 617-899-1995

EA: Allison Luke (ALuke@ihi.org)
Office: +16175757758

Join IHI at the 2019 IHI National Forum on Quality Improvement in Health Care
Dec. 8–11, 2019 ihi.org/Forum

Frank Federico <ffederico@ihi.org> 13 de ago de 2019 17:30
para eu, Val

Sorry that this process is taking some time.
We now have two Portuguese-language versions of the Global trigger tool and your version of the surgical triggers.
My role now is to compare the different versions for consistency.

There is a place for your version of the surgical triggers on the website. We are working to find the best place to post it for maximum exposure and link to the other tools.
Did you prepare a manuscript that contains your research?

Frank Federico, RPh

Vice President, Senior Safety ExpertMobile: 617-899-1995

EA: Allison Luke (ALuke@ihi.org)
Office: +16175757758

Join IHI at the 2019 IHI National Forum on Quality Improvement in Health Care
Dec. 8–11, 2019 ihi.org/Forum

ANEXO I – PARECER CONSUBSTANCIADO CEP HOSPITAL A.

CEP/HC/UFPR

UFPR - HOSPITAL DE CLÍNICAS DA UNIVERSIDADE FEDERAL DO PARANÁ -

Plataforma Brasil

PARECER CONSUBSTANCIADO DO CEP

DADOS DO PROJETO DE PESQUISA

Título da Pesquisa: AVALIAÇÃO DA CULTURA DE SEGURANÇA E OCORRÊNCIA DE EVENTOS ADVERSOS CIRÚRGICOS EM HOSPITAIS BRASILEIROS

Pesquisador: ELAINE DREHMER DE ALMEIDA CRUZ

Área Temática:

Versão: 2

CAAE: 62101216.7.0000.0096

Instituição Proponente: Hospital de Clínicas da Universidade Federal do Paraná

Patrocinador Principal: Financiamento Próprio

DADOS DO PARECER

Número do Parecer: 1.990.760

Apresentação do Projeto:

Apresentação dos documentos pendentes e correções do projeto de pesquisa sugeridas no parecer número 1.891.184 emitido por este Comitê de Ética em Pesquisa em Seres Humanos.

Objetivo da Pesquisa:

Os objetivos do estudo permanecem os mesmo, não houve alteração.

Avaliação dos Riscos e Benefícios:

Foi acrescentado pelos pesquisadores o risco relacionado a manipulação do prontuário do paciente:

- Em caso de crime à violação de informações sigilosas que possam acarretar em danos físicos, psíquicos e morais aos pacientes envolvidos, os pesquisadores serão responsáveis em prestar a assistência necessária e estão cientes das penalidades cabíveis, conforme à legislação brasileira. Caso ocorra a perda e/ou extravio de documentos, os órgãos competentes serão comunicados imediatamente para elaboração de novo prontuário, onde será anotado a ocorrência de extravio do anterior, bem como, será avisado o paciente.

Comentários e Considerações sobre a Pesquisa:

Os pesquisadores apresentaram uma proposta de estudo relevante para instituições envolvidas:

Endereço: Rua Gal. Carneiro, 181
Bairro: Alto da Glória **CEP:** 80.060-900
UF: PR **Município:** CURITIBA
Telefone: (41)3360-1041 **Fax:** (41)3360-1041 **E-mail:** cep@hc.ufpr.br

Hospital de Clínicas da Universidade Federal do Paraná, Hospital do Trabalhador e Hospital Evangélico. Nesta etapa, fizeram as adequações necessárias para iniciar a coleta de dados com os profissionais das unidades cirúrgica e clinica cirúrgica dos hospitais mencionados e a busca de informações em prontuários dos pacientes que foram submetidos a procedimentos cirúrgicos a fim de levantar a ocorrência de algum evento adverso.

Considerações sobre os Termos de apresentação obrigatória:

Foram anexados os seguintes documentos:

- Termo de Concordância do Hospital do Trabalhador assinado pelo diretor da instituição;
- Declaração de ciência a respeito do estudo, emitidas pelos responsáveis do Núcleo de Segurança do Paciente das instituições envolvidas;
- Termo de Consentimento Livre e Esclarecido devidamente adequado as sugestões do Comitê de Ética em Pesquisa em Seres Humanos.

Recomendações:

É obrigatório trazer ao CEP/HC uma via do Termo de Consentimento Livre e Esclarecido que foi aprovado, para assinatura e rubrica. Após, xerocar este TCLE em duas vias, uma ficará com o pesquisador e uma para o participante da pesquisa.

Conclusões ou Pendências e Lista de Inadequações:

Todas as pendências elencadas no parecer anterior foram atendidas. Sendo assim, a coleta de dados pode iniciar após emissão do parecer final deste Comitê de Ética em Pesquisa em Seres Humanos.

Considerações Finais a critério do CEP:

Diante do exposto, o Comitê de Ética em Pesquisa em Seres Humanos do HC-UFPR, de acordo com as atribuições definidas na Resolução CNS 466/2012 e na Norma Operacional Nº 001/2013 do CNS, manifesta -se pela aprovação do projeto conforme proposto para início da Pesquisa. Solicitamos que sejam apresentados a este CEP, relatórios semestrais sobre o andamento da pesquisa, bem como informações relativas às modificações do protocolo, cancelamento, encerramento e destino dos conhecimentos obtidos. Manter os documentos da pesquisa arquivado.

É dever do CEP acompanhar o desenvolvimento dos projetos, por meio de relatórios semestrais dos pesquisadores e de outras estratégias de monitoramento, de acordo com o risco inerente à pesquisa.

Endereço: Rua Gal. Carneiro, 181
Bairro: Alto da Glória **CEP:** 80.060-900
UF: PR **Município:** CURITIBA
Telefone: (41)3360-1041 **Fax:** (41)3360-1041 **E-mail:** cep@hc.ufpr.br

Continuação do Parecer: 1.990.760

Este parecer foi elaborado baseado nos documentos abaixo relacionados:

Tipo Documento	Arquivo	Postagem	Autor	Situação
Informações Básicas do Projeto	PB_INFORMAÇÕES_BÁSICAS_DO_PROJETO_823657.pdf	19/02/2017 14:09:17		Aceito
Outros	CARTA_RESPOSTA_CEP_HC.pdf	19/02/2017 14:08:31	ELAINE DREHMER DE ALMEIDA CRUZ	Aceito
Outros	PROJETO_CORRIGIDO.docx	19/02/2017 14:06:35	ELAINE DREHMER DE ALMEIDA CRUZ	Aceito
Outros	DECLARACAO_NSP_HT.pdf	19/02/2017 14:02:36	ELAINE DREHMER DE ALMEIDA CRUZ	Aceito
Outros	DELCARACAO_NSP_EVANGELICO.pdf	19/02/2017 14:01:38	ELAINE DREHMER DE ALMEIDA CRUZ	Aceito
Outros	DECLARACAO_NSP_HC_UFPR.pdf	19/02/2017 13:58:45	ELAINE DREHMER DE ALMEIDA CRUZ	Aceito
Outros	AUTORIZACAO_DIRETOR_HT.pdf	19/02/2017 13:55:15	ELAINE DREHMER DE ALMEIDA CRUZ	Aceito
TCLE / Termos de Assentimento / Justificativa de Ausência	TCLE_CORRIGIDO.pdf	19/02/2017 13:50:07	ELAINE DREHMER DE ALMEIDA CRUZ	Aceito
Outros	ANEXOD_FORMULARIOAVALIACAO.pdf	16/11/2016 15:22:08	ELAINE DREHMER DE ALMEIDA CRUZ	Aceito
Outros	ANEXOC_CRITERIOSRASTREAMENTO.pdf	16/11/2016 15:21:24	ELAINE DREHMER DE ALMEIDA CRUZ	Aceito
Outros	ANEXOB_GLOBALTRIGGERTOOL.pdf	16/11/2016 15:20:30	ELAINE DREHMER DE ALMEIDA CRUZ	Aceito
Outros	ANEXOA_QUESTIONARIOHSOPSC.pdf	16/11/2016 15:19:30	ELAINE DREHMER DE ALMEIDA CRUZ	Aceito
Projeto Detalhado / Brochura Investigador	Projeto_Doutorado.docx	16/11/2016 15:16:50	ELAINE DREHMER DE ALMEIDA CRUZ	Aceito
TCLE / Termos de Assentimento / Justificativa de Ausência	TCLE.pdf	16/11/2016 15:13:01	ELAINE DREHMER DE ALMEIDA CRUZ	Aceito
Outros	QUALIFICACAO_PESQUISADORES.pdf	16/11/2016 15:11:39	ELAINE DREHMER DE ALMEIDA CRUZ	Aceito
Outros	TermoResponsabilidadePesquisa.pdf	16/11/2016 15:09:34	ELAINE DREHMER DE ALMEIDA CRUZ	Aceito
Declaração de Pesquisadores	DECLARCOMPROMISSOPESQUISADORES.pdf	16/11/2016 15:08:22	ELAINE DREHMER DE ALMEIDA CRUZ	Aceito
Outros	TermoCompromissoUtilizacaoDados.pdf	16/11/2016 15:06:37	ELAINE DREHMER DE ALMEIDA CRUZ	Aceito
Outros	DECLARUSODEDADOS.pdf	16/11/2016 15:04:54	ELAINE DREHMER DE ALMEIDA CRUZ	Aceito
Outros	DECLARTORNARPUBLICOOSRESULT	16/11/2016	ELAINE DREHMER	Aceito

Outros	DOS.pdf	15:03:31	DE ALMEIDA CRUZ	Aceito
Outros	TERMODECONFIDENCIALIDADE.pdf	16/11/2016 15:01:51	ELAINE DREHMER DE ALMEIDA CRUZ	Aceito
Outros	ConcordanciaHospitalEvangelico.pdf	16/11/2016 14:57:19	ELAINE DREHMER DE ALMEIDA CRUZ	Aceito
Outros	ConcordanciaHospitalTrabalhador.pdf	16/11/2016 14:54:10	ELAINE DREHMER DE ALMEIDA CRUZ	Aceito
Outros	ConcordanciaHC.pdf	16/11/2016 14:53:06	ELAINE DREHMER DE ALMEIDA CRUZ	Aceito
Outros	DECLARORIENTADORALUNO.pdf	16/11/2016 14:46:55	ELAINE DREHMER DE ALMEIDA CRUZ	Aceito
Outros	CARTAENCAMINHAMENTOPESQUISADORAOCEP.pdf	16/11/2016 14:45:01	ELAINE DREHMER DE ALMEIDA CRUZ	Aceito
Folha de Rosto	FolhaRosto.pdf	16/11/2016 14:42:46	ELAINE DREHMER DE ALMEIDA CRUZ	Aceito

Situação do Parecer:
Aprovado

Necessita Apreciação da CONEP:
Não

CURITIBA, 30 de Março de 2017

Assinado por:
Renato Tambara Filho
(Coordenador)

Endereço: Rua Gal. Carneiro, 181
Bairro: Alto da Glória **CEP:** 80.060-900
UF: PR **Município:** CURITIBA
Telefone: (41)3360-1041 **Fax:** (41)3360-1041 **E-mail:** cep@hc.ufpr.br

ANEXO J – PARECER COMITÊ DE ÉTICA EM PESQUISA HOSPITAL B.

SOCIEDADE EVANGÉLICA BENEFICENTE DE CURITIBA - PR

PARECER CONSUBSTANCIADO DO CEP

Elaborado pela Instituição Coparticipante

DADOS DO PROJETO DE PESQUISA

Título da Pesquisa: AVALIAÇÃO DA CULTURA DE SEGURANÇA E OCORRÊNCIA DE EVENTOS ADVERSOS CIRÚRGICOS EM HOSPITAIS BRASILEIROS

Pesquisador: ELAINE DREHMER DE ALMEIDA CRUZ

Área Temática:

Versão: 2

CAAE: 62101216.7.3001.0103

Instituição Proponente: Hospital de Clínicas da Universidade Federal do Paraná

Patrocinador Principal: Financiamento Próprio

DADOS DO PARECER

Número do Parecer: 2.325.448

Apresentação do Projeto:

Pretende-se desenvolver uma pesquisa para avaliar a relação da cultura de segurança e a ocorrência de Eventos Adversos (EA) cirúrgicos em hospitais na cidade de Curitiba, estado Paraná. Conhecer os EA cirúrgicos notificados na instituição e compará-los com os EA cirúrgicos encontrados por meio da técnica de revisão de prontuários permitirá conhecer sua real prevalência, além de relacionar à cultura de segurança dos trabalhadores de saúde que prestam assistência.Esta pesquisa pretende fornecer subsídios para conhecer os resultados da incidência de EAs cirúrgicos, acompanhar a ocorrência destes até um ano após as cirurgias que utilizam materiais de implante, avaliar a relação dos EAs com mortalidade e identificar fatores de risco para sua ocorrência. Tais dados poderão subsidiar a análise das ações institucionais em prol da segurança do paciente, bem como sua comparação com os EA cirúrgicos notificados por meio do sistema hospitalar, o potencial de prevenção e sua gravidade no decorrer do tempo. Os resultados da cultura de segurança serão mensurados por instrumento específico, por meio de dados gerais e por domínios e poderão subsidiar ações com vistas a corrigir fragilidades. A análise da relação entre a cultura de segurança e a ocorrência de EAs em pacientes cirúrgicos poderá somar-se aos resultados de pesquisas internacionais. A comparação entre EA e cultura de segurança e gestão do hospital, permitirá conhecer sua influência na cultura e ocorrência de Evento Adverso.

Endereço: Rua Padre Anchieta, 2770
Bairro: Bigorrilho **CEP:** 80.730-000
UF: PR **Município:** CURITIBA
Telefone: (41)3240-5570 **Fax:** (41)3240-5584 **E-mail:** comite.etica@fepar.edu.br

Metodologia Proposta:
Pesquisa avaliativa, de abordagem quantitativa, sobre a ocorrência de EAs e de suas possíveis causas em hospitais brasileiros e a relação entre
cultura de segurança, eventos adversos cirúrgicos e gestão hospitalar.Neste estudo será considerado os dados que possibilitem a avaliação da
ocorrência de EAs por meio do método de revisão retrospectiva de prontuários, a partir de um protocolo desenvolvido pelo Canadian Adverse Events
Study (CAES), bem como será considerado os dados dos trabalhadores de saúde que prestam assistência à saúde de modo direto ou indireto em
centro cirúrgico ou unidades de internação cirúrgicas, por meio de entrevistas e instrumento específico para avaliar a cultura de segurança. Optou-se
por essa especialidade haja visto que os procedimentos cirúrgicos estão no ranking das ocorrências de Eventos Adversos (BAKER; NORTON;
FLINTOFT et al., 2004; MENDES; MARTINS; ROZENFELD, et al., 2009).A população do estudo está apresentada em dois grupos, considerando-se
os dois principais fatores a serem investigados, qual sejam, a cultura de segurança – trabalhadores (grupo 1); e a ocorrência de eventos adversos
cirúrgicos – prontuários de pacientes (grupo 2).
Critério de Inclusão:
Os critérios de inclusão serão os profissionais lotados e atuantes nos Centros Cirúrgicos ou Unidades de Internação Cirúrgicas dos hospitais participantes (grupo 1), no período de coleta de dados; com carga horária mínima semanal de trabalho de 20 horas; com contato direto ou interação com pacientes cirúrgicos, e aqueles sem contato direto com o paciente, mas com função de liderança, gerência ou supervisão (AHRQ, 2004).
Critérios de Inclusão:
Para a inclusão da amostra de dados de prontuários dos pacientes cirúrgicos (grupo 2) será considerado o período de (agosto/2014 a setembro 2015) e de (agosto 2016 a setembro 2017) por meio de listagem de pacientes operados emitidas pelos Centros Cirúrgicos dos respectivos hospitais participantes, durante o período de coleta de dados, de pacientes submetidos a procedimentos cirúrgicos índice (primeiro procedimento cirúrgico realizado na internação) independentemente de sexo, idade, tipo ou especialidade cirúrgica e tempo de internamento.
Critério de Exclusão:
Serão excluídos da análise os participantes do grupo 1 cujos questionários tiverem menos de 50%

de perguntas preenchidas (sessões A a G) e/ou pelo menos uma seção com todas as perguntas não preenchidas (AHRQ,2004). Serão excluídos do grupo 2 os prontuários de pacientes psiquiátricos e dados de reoperações de cirurgia índice (primeiro procedimento cirúrgico realizado na internação) das internações ocorridas no período de coleta de dados.

Objetivo da Pesquisa:

Objetivo Primário:

Investigar a relação entre cultura de segurança, eventos adversos cirúrgicos e gestão hospitalar

Objetivo Secundário:

- Mensurar a cultura de segurança.- Estimar a prevalência de eventos adversos cirúrgicos- Verificar a associação do escore geral e por domínios da cultura de segurança com a ocorrência de eventos adversos cirúrgicos - Comparar a frequência de eventos adversos notificados e identificados por busca ativa- Monitorar ocorrência de eventos adversos em pacientes submetidos à cirurgias com implante até um ano de pós operatório- Avaliar a

relação dos eventos adversos com mortalidade - Comparar a cultura de segurança e a incidência de eventos adversos cirúrgicos com a variável gestão organizacional.

Avaliação dos Riscos e Benefícios:

Riscos:

Em relação aos trabalhadores (grupo 1), considera-se que o uso de instrumentos pode intervir na variável psicológica. Contudo, os pesquisadores buscarão minimizar desconfortos ou constrangimentos, por meio da não identificação dos participantes, disponibilizando momento e local apropriado à participação e preenchimento do instrumento; e envelope para a sua devolução de forma confidencial. Os materiais para a pesquisa (formulário, caneta e envelope) serão fornecidos aos participantes pelos pesquisadores. Os pesquisadores orientarão os participantes que em caso de algum item do instrumento ou do processo de coleta de dados lhe causar desconforto o mesmo poderá se abster de responder caso lhe seja conveniente e o pesquisador estará disponível para elucidar dúvidas.

Trata-se de riscos mínimos considerando-se não haver intervenção nas variáveis fisiológicas, psicológicas e sociais dos pacientes, uma vez que parte da busca é documental.

Benefícios:

Entre os benefícios da pesquisa destacam-se que os resultados dessa investigação revelarão o diagnóstico situacional referente à cultura de segurança que poderá contribuir para implementação de estratégias de práticas seguras em ambiente hospitalar, incluindo centros cirúrgicos e clínicas cirúrgicas, relevantes na área da saúde e na promoção da cultura de segurança

Endereço: Rua Padre Anchieta, 2770
Bairro: Bigorrilho **CEP:** 80.730-000
UF: PR **Município:** CURITIBA
Telefone: (41)3240-5570 **Fax:** (41)3240-5584 **E-mail:** comite.etica@fepar.edu.br

e qualidade do cuidado conforme recomendações da OMS e diretrizes nacionais. Os resultados ainda servirão de substrato para futuras análises relacionados a cultura de segurança do paciente em ambiente cirúrgico e eventos adversos em outros estabelecimentos de saúde brasileiro.

Comentários e Considerações sobre a Pesquisa:

Justificativa da Emenda:

Tendo em vista que o referido projeto não foi replicado para o Comitê de Ética do Hospital do Trabalhador (SESA -Pr), devido ao erro no número do CNPJ fornecido no momento da submissão, solicitamos autorização desta emenda, para que o projeto possa ser avaliado pela referida instituição co-participante. Salientamos que os documentos com a autorização do Diretor Médico do Hospital do Trabalhador e do Núcleo de Segurança já estavam anexados desde o início desta submissão, a qual já foi autorizada pelo CEP das demais instituições participantes. Neste sentido, foi incluído na plataforma Brasil, o número correto do CNPJ do Hospital do Trabalhador, conforme orientação do CEP-HT (CNPJ 76416866/0001-40).

Considerações sobre os Termos de apresentação obrigatória:

Adequados.

Recomendações:

Não há.

Conclusões ou Pendências e Lista de Inadequações:

Não há.

Considerações Finais a critério do CEP:

Este parecer foi elaborado baseado nos documentos abaixo relacionados:

Tipo Documento	Arquivo	Postagem	Autor	Situação
Informações Básicas do Projeto	PB_INFORMAÇÕES_BÁSICAS_974453_E1.pdf	16/08/2017 12:15:19		Aceito
Declaração de Pesquisadores	EMENDA_03.pdf	16/08/2017 12:14:27	ELAINE DREHMER DE ALMEIDA CRUZ	Aceito
Outros	CARTA_RESPOSTA_CEP_HC.pdf	19/02/2017 14:08:31	ELAINE DREHMER DE ALMEIDA CRUZ	Aceito
Outros	PROJETO_CORRIGIDO.docx	19/02/2017 14:06:35	ELAINE DREHMER DE ALMEIDA CRUZ	Aceito

SOCIEDADE EVANGÉLICA BENEFICENTE DE CURITIBA - PR

Continuação do Parecer: 2.325.448

Outros	DECLARACAO_NSP_HT.pdf	19/02/2017 14:02:36	ELAINE DREHMER DE ALMEIDA CRUZ	Aceito
Outros	DELCARACAO_NSP_EVANGELICO.pdf	19/02/2017 14:01:38	ELAINE DREHMER DE ALMEIDA CRUZ	Aceito
Outros	DECLARACAO_NSP_HC_UFPR.pdf	19/02/2017 13:58:45	ELAINE DREHMER DE ALMEIDA CRUZ	Aceito
Outros	AUTORIZACAO_DIRETOR_HT.pdf	19/02/2017 13:55:15	ELAINE DREHMER DE ALMEIDA CRUZ	Aceito
TCLE / Termos de Assentimento / Justificativa de Ausência	TCLE_CORRIGIDO.pdf	19/02/2017 13:50:07	ELAINE DREHMER DE ALMEIDA CRUZ	Aceito
Outros	ANEXOD_FORMULARIOAVALIACAO.pdf	16/11/2016 15:22:08	ELAINE DREHMER DE ALMEIDA CRUZ	Aceito
Outros	ANEXOC_CRITERIOSRASTREAMENTO.pdf	16/11/2016 15:21:24	ELAINE DREHMER DE ALMEIDA CRUZ	Aceito
Outros	ANEXOB_GLOBALTRIGGERTOOL.pdf	16/11/2016 15:20:30	ELAINE DREHMER DE ALMEIDA CRUZ	Aceito
Outros	ANEXOA_QUESTIONARIOHSOPSC.pdf	16/11/2016 15:19:30	ELAINE DREHMER DE ALMEIDA CRUZ	Aceito
Projeto Detalhado / Brochura Investigador	Projeto_Doutorado.docx	16/11/2016 15:16:50	ELAINE DREHMER DE ALMEIDA CRUZ	Aceito
TCLE / Termos de Assentimento / Justificativa de Ausência	TCLE.pdf	16/11/2016 15:13:01	ELAINE DREHMER DE ALMEIDA CRUZ	Aceito
Outros	QUALIFICACAO_PESQUISADORES.pdf	16/11/2016 15:11:39	ELAINE DREHMER DE ALMEIDA CRUZ	Aceito
Outros	TermoResponsabilidadePesquisa.pdf	16/11/2016 15:09:34	ELAINE DREHMER DE ALMEIDA CRUZ	Aceito
Declaração de Pesquisadores	DECLARCOMPROMISSOPESQUISADORES.pdf	16/11/2016 15:08:22	ELAINE DREHMER DE ALMEIDA CRUZ	Aceito
Outros	TermoCompromissoUtilizacaoDados.pdf	16/11/2016 15:06:37	ELAINE DREHMER DE ALMEIDA CRUZ	Aceito
Outros	DECLARUSODEDADOS.pdf	16/11/2016 15:04:54	ELAINE DREHMER DE ALMEIDA CRUZ	Aceito
Outros	DECLARTORNARPUBLICOOSRESULTADOS.pdf	16/11/2016 15:03:31	ELAINE DREHMER DE ALMEIDA CRUZ	Aceito
Outros	TERMODECONFIDENCIALIDADE.pdf	16/11/2016 15:01:51	ELAINE DREHMER DE ALMEIDA CRUZ	Aceito
Outros	ConcordanciaHospitalEvangelico.pdf	16/11/2016 14:57:19	ELAINE DREHMER DE ALMEIDA CRUZ	Aceito
Outros	ConcordanciaHospitalTrabalhador.pdf	16/11/2016 14:54:10	ELAINE DREHMER DE ALMEIDA CRUZ	Aceito
Outros	ConcordanciaHC.pdf	16/11/2016 14:53:06	ELAINE DREHMER DE ALMEIDA CRUZ	Aceito

Endereço: Rua Padre Anchieta, 2770
Bairro: Bigorrilho **CEP:** 80.730-000
UF: PR **Município:** CURITIBA
Telefone: (41)3240-5570 **Fax:** (41)3240-5584 **E-mail:** comite.etica@fepar.edu.br

Outros	DECLARORIENTADORALUNO.pdf	16/11/2016 14:46:55	ELAINE DREHMER DE ALMEIDA CRUZ	Aceito
Outros	CARTAENCAMINHAMENTOPESQUISADORAOCEP.pdf	16/11/2016 14:45:01	ELAINE DREHMER DE ALMEIDA CRUZ	Aceito
Folha de Rosto	FolhaRosto.pdf	16/11/2016 14:42:46	ELAINE DREHMER DE ALMEIDA CRUZ	Aceito

Situação do Parecer:
Aprovado

Necessita Apreciação da CONEP:
Não

CURITIBA, 10 de Outubro de 2017

Assinado por:
JURANDIR MARCONDES RIBAS FILHO
(Coordenador)

Endereço: Rua Padre Anchieta, 2770
Bairro: Bigorrilho **CEP:** 80.730-000
UF: PR **Município:** CURITIBA
Telefone: (41)3240-5570 **Fax:** (41)3240-5584 **E-mail:** comite.etica@fepar.edu.br

ANEXO K – PARECER COMITÊ DE ÉTICA EM PESQUISA HOSPITAL C.

PARANÁ
GOVERNO DO ESTADO

HOSPITAL DO
TRABALHADOR/SES/PR

Plataforma Brasil

PARECER CONSUBSTANCIADO DO CEP

Elaborado pela Instituição Coparticipante

DADOS DO PROJETO DE PESQUISA

Título da Pesquisa: AVALIAÇÃO DA CULTURA DE SEGURANÇA E OCORRÊNCIA DE EVENTOS ADVERSOS CIRÚRGICOS EM HOSPITAIS BRASILEIROS

Pesquisador: ELAINE DREHMER DE ALMEIDA CRUZ

Área Temática:

Versão: 1

CAAE: 62101216.7.3002.5225

Instituição Proponente: secretaria de estado da saude do parana

Patrocinador Principal: Financiamento Próprio

DADOS DO PARECER

Número do Parecer: 2.367.592

Apresentação do Projeto:

Apresentação de Emenda para correção do número de Cadastro Nacional de Pessoas Jurídicas de um dos centros coparticipantes.

Objetivo da Pesquisa:

Alteração do número de Cadastro Nacional de Pessoas Jurídicas do Hospital do Trabalhador

Avaliação dos Riscos e Benefícios:

Não apresenta risco

Comentários e Considerações sobre a Pesquisa:

Pesquisa de interesse público que obedece os critérios éticos em pesquisa.

Considerações sobre os Termos de apresentação obrigatória:

Apresenta a declaração de concordância assinada pela Direção Técnica do Hospital do Trabalhador autorizando a pesquisa.

Conclusões ou Pendências e Lista de Inadequações:

Aprovada para continuidade e solicitamos que seja protocolado a conclusão desta pesquisa.

Considerações Finais a critério do CEP:

Endereço: Secretaria da Saúde Rua Piquiri 170 - Rebouças - 80230-140 - Curitiba - PR 41 3330-4300
Bairro: Rebouças **CEP:** 81.050-000
UF: PR **Município:** CURITIBA
Telefone: (41)3212-5871 **E-mail:** cepht@sesa.pr.gov.br

Este parecer foi elaborado baseado nos documentos abaixo relacionados:

Tipo Documento	Arquivo	Postagem	Autor	Situação
Informações Básicas do Projeto	PB_INFORMAÇÕES_BÁSICAS_974453 E1.pdf	16/08/2017 12:15:19		Aceito
Declaração de Pesquisadores	EMENDA_03.pdf	16/08/2017 12:14:27	ELAINE DREHMER DE ALMEIDA CRUZ	Aceito
Informações Básicas do Projeto	PB_INFORMAÇÕES_BÁSICAS_974453 E1.pdf	07/08/2017 15:30:26		Aceito
Outros	CARTA_RESPOSTA_CEP_HC.pdf	19/02/2017 14:08:31	ELAINE DREHMER DE ALMEIDA CRUZ	Aceito
Outros	PROJETO_CORRIGIDO.docx	19/02/2017 14:06:35	ELAINE DREHMER DE ALMEIDA CRUZ	Aceito
Outros	DECLARACAO_NSP_HT.pdf	19/02/2017 14:02:36	ELAINE DREHMER DE ALMEIDA CRUZ	Aceito
Outros	DELCARACAO_NSP_EVANGELICO.pd f	19/02/2017 14:01:38	ELAINE DREHMER DE ALMEIDA CRUZ	Aceito
Outros	DECLARACAO_NSP_HC_UFPR.pdf	19/02/2017 13:58:45	ELAINE DREHMER DE ALMEIDA CRUZ	Aceito
Outros	AUTORIZACAO_DIRETOR_HT.pdf	19/02/2017 13:55:15	ELAINE DREHMER DE ALMEIDA CRUZ	Aceito
TCLE / Termos de Assentimento / Justificativa de Ausência	TCLE_CORRIGIDO.pdf	19/02/2017 13:50:07	ELAINE DREHMER DE ALMEIDA CRUZ	Aceito
Outros	ANEXOD_FORMULARIOAVALIACAO.p df	16/11/2016 15:22:08	ELAINE DREHMER DE ALMEIDA CRUZ	Aceito
Outros	ANEXOC_CRITERIOSRASTREAMENT O.pdf	16/11/2016 15:21:24	ELAINE DREHMER DE ALMEIDA CRUZ	Aceito
Outros	ANEXOB_GLOBALTRIGGERTOOL.pdf	16/11/2016 15:20:30	ELAINE DREHMER DE ALMEIDA CRUZ	Aceito
Outros	ANEXOA_QUESTIONARIOHSOPSC.pd f	16/11/2016 15:19:30	ELAINE DREHMER DE ALMEIDA CRUZ	Aceito
Projeto Detalhado / Brochura Investigador	Projeto_Doutorado.docx	16/11/2016 15:16:50	ELAINE DREHMER DE ALMEIDA CRUZ	Aceito
TCLE / Termos de Assentimento / Justificativa de Ausência	TCLE.pdf	16/11/2016 15:13:01	ELAINE DREHMER DE ALMEIDA CRUZ	Aceito
Outros	QUALIFICACAO_PESQUISADORES.pd f	16/11/2016 15:11:39	ELAINE DREHMER DE ALMEIDA CRUZ	Aceito
Outros	TermoResponsabilidadePesquisa.pdf	16/11/2016 15:09:34	ELAINE DREHMER DE ALMEIDA CRUZ	Aceito
Declaração de Pesquisadores	DECLARCOMPROMISSOPESQUISAD ORES.pdf	16/11/2016 15:08:22	ELAINE DREHMER DE ALMEIDA CRUZ	Aceito
Outros	TermoCompromissoUtilizacaoDados.	16/11/2016	ELAINE DREHMER	Aceito

Outros	pdf	15:06:37	DE ALMEIDA CRUZ	Aceito
Outros	DECLARUSODEDADOS.pdf	16/11/2016 15:04:54	ELAINE DREHMER DE ALMEIDA CRUZ	Aceito
Outros	DECLARTORNARPUBLICOOSRESULTADOS.pdf	16/11/2016 15:03:31	ELAINE DREHMER DE ALMEIDA CRUZ	Aceito
Outros	TERMODECONFIDENCIALIDADE.pdf	16/11/2016 15:01:51	ELAINE DREHMER DE ALMEIDA CRUZ	Aceito
Outros	ConcordanciaHospitalEvangelico.pdf	16/11/2016 14:57:19	ELAINE DREHMER DE ALMEIDA CRUZ	Aceito
Outros	ConcordanciaHospitalTrabalhador.pdf	16/11/2016 14:54:10	ELAINE DREHMER DE ALMEIDA CRUZ	Aceito
Outros	ConcordanciaHC.pdf	16/11/2016 14:53:06	ELAINE DREHMER DE ALMEIDA CRUZ	Aceito
Outros	DECLARORIENTADORALUNO.pdf	16/11/2016 14:46:55	ELAINE DREHMER DE ALMEIDA CRUZ	Aceito
Outros	CARTAENCAMINHAMENTOPESQUISADORAOCEP.pdf	16/11/2016 14:45:01	ELAINE DREHMER DE ALMEIDA CRUZ	Aceito
Folha de Rosto	FolhaRosto.pdf	16/11/2016 14:42:46	ELAINE DREHMER DE ALMEIDA CRUZ	Aceito

Situação do Parecer:
Aprovado

Necessita Apreciação da CONEP:
Não

CURITIBA, 06 de Novembro de 2017

Assinado por:
silvania klug pimentel
(Coordenador)
